# To tether or not to tether? The merits of adding functionality to Li–O_2_ cell redox mediators

**DOI:** 10.1039/d6eb00134c

**Published:** 2026-08-24

**Authors:** Thukshan Samarakoon, Alex R. Neale, Elliot Coulbeck, Daniel J. Saccomando, Tyler Petek, Laurence J. Hardwick

**Affiliations:** a Stephenson Institute for Renewable Energy, Department of Chemistry, University of Liverpool Liverpool L69 7ZF UK hardwick@liverpool.ac.uk; b Lubrizol Limited Blackley Manchester M9 8ES UK; c Lubrizol Limited Hazelwood Derby DE56 4AN UK; d Lubrizol Limited 29400 Lakeland Boulevard Wickliffe Ohio 44092-2201 USA

## Abstract

The cycle life of lithium–oxygen (Li–O_2_) cells is limited by the continual degradation of cell components by both potential-independent and high overpotential-induced parasitic reactions. Extending cell cycle life requires (i) driving electrochemical reactions at low overpotentials with high selectivity, (ii) stabilising the Li|electrolyte interface and (iii) mitigating reactive oxygen species (ROS)-induced electrolyte/electrode degradation. Charge redox mediators (RMs) present a promising strategy to facilitate low overpotential oxidation of Li_2_O_2_. By covalently linking or “tethering” the RM to motifs that can enhance cell discharge, quench ROS, and/or stabilise the Li|electrolyte interface, a single multifunctional species can be realised to improve cell lifetime. In this work, a series of 2,2,6,6-tetramethylpiperidine-1-oxyl (TEMPO)-tethered RMs based on ionic liquids/salts is reported. The effect of these additives on discharge capacities and cell cyclability was explored systematically in Li–O_2_ cells. The tethered RMs gave superior performance relative to TEMPO when using Li_1−*x*_FePO_4_ as the counter electrode. Improved performance in Li metal-based cells with the tethered RMs was attained by substitution of the bis(trifluoromethanesulfonyl)imide ([TFSI]^−^) anion of the RM with nitrate ([NO_3_]^−^), resulting in an exceptionally high Li_2_O_2_ yield close to 100% on discharge, almost 15% greater than for the analogous [TFSI]^−^-containing tethered RM. A marker for cell/RM failure was identified with *operando* gas evolution measurements to track charge mediation losses by the tethered RMs. Furthermore, degradation reactions with Li metal and superoxide, where superoxide-induced decomposition occurs at the TEMPO moiety, were identified as key RM degradation pathways, and the major bottleneck to achieving high cycling performance was established.

## Abstract 2

It's better to tether: Tethered redox mediators enhance Li–O_2_ cell discharge capacities and rechargeability.

## Graphical abstract

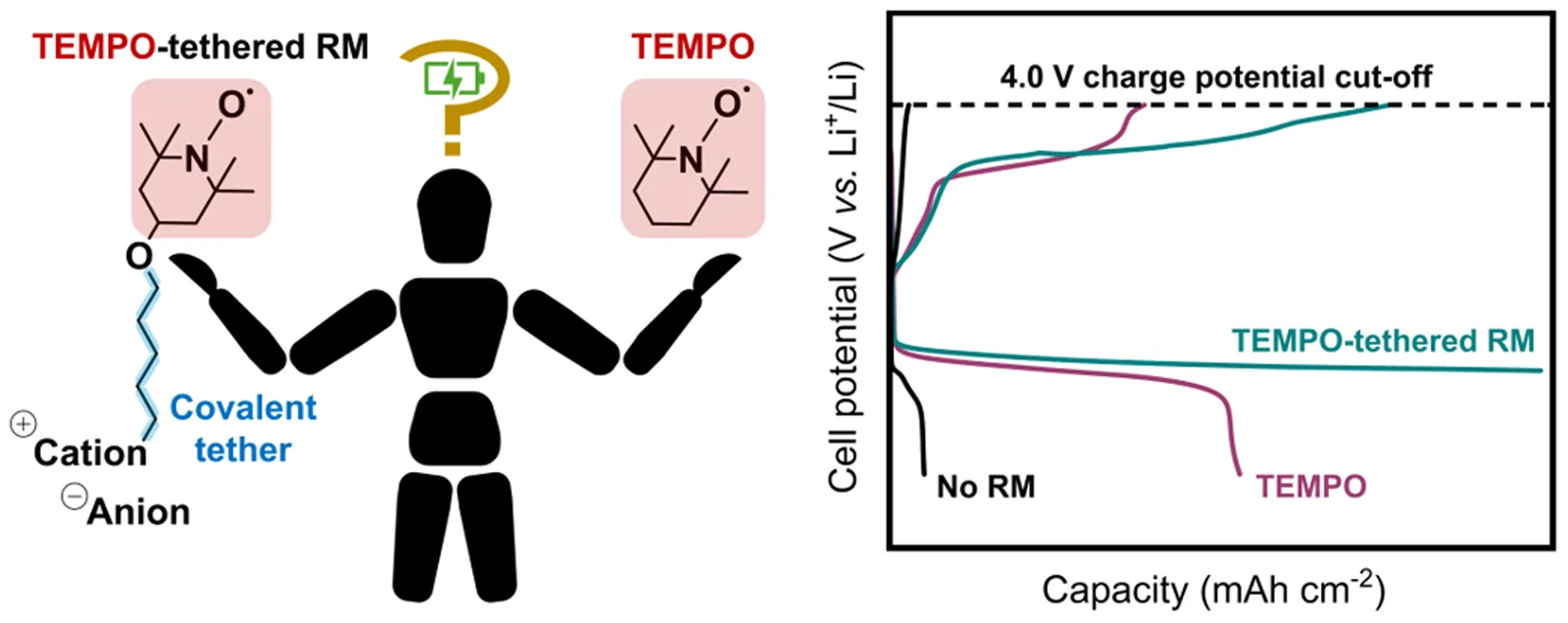

**Broader context**

Higher specific energy batteries, far exceeding the limit of lithium-ion batteries, will be critical to the realisation of long-range electric heavy goods vehicles and electrification of the aviation industry. This has led to significant research on the lithium–oxygen (Li–O_2_) battery, a chemistry with practical specific energies estimated to be 2–3-fold greater than lithium-ion batteries. However, poor cycle life has hampered the realisation of a practical Li–O_2_ cell, due to reactive oxygen species (ROS) generated during cycling, and high charge overpotentials required to oxidise the primary discharge product, lithium peroxide (Li_2_O_2_). Electrolyte-soluble redox mediators (RMs) can decrease charge overpotentials by catalysing the oxidation of Li_2_O_2_, but they suffer from decomposition by Li metal and ROS. Therefore, multifunctional RMs that can mediate cell charging, as well as enhance discharge, resist ROS-attack, and improve Li|electrolyte interfacial stability are crucial to extending cell lifetime. Covalently linking or “tethering” a redox-active moiety to the cation of an ionic liquid (IL)/salt has been shown to yield multifunctional RMs, but a focus on methodical structural variation of such tethered RMs and the corresponding cell performance relative to their untethered counterparts is largely lacking in the field. This is the focus of this work, wherein a series of redox-active ILs/salts were synthesised and employed as RMs in Li–O_2_ cells. It is demonstrated through systematic experimentation that RM tethering can enhance cell performance relative to the untethered equivalent.

## Introduction

Despite the promise of lithium–oxygen (Li–O_2_) batteries to deliver high specific energies (>1000 Wh kg^−1^), this is yet to be realised with high efficiencies and commercially relevant lifetimes.^1^ Practical specific energies are still not clearly defined as various cell configurations, based on liquid, solid and hybrid (solid–liquid) electrolytes, are currently being explored.^2,3^ For most Li–O_2_ systems, cell discharge proceeds *via* a two-electron oxygen reduction reaction (ORR) at the positive electrode (typically carbon-based) and oxidation of Li (to Li^+^) at the Li metal negative electrode. Together, this yields lithium peroxide (Li_2_O_2_) as the discharge product at the carbon electrode. On charge, Li_2_O_2_ is electrochemically oxidised to evolve O_2_ gas and Li^+^ (oxygen evolution reaction, OER), which is balanced by the reduction of Li^+^ to Li^0^ at the negative electrode. High charge overpotentials are required for electrochemical oxidation of Li_2_O_2_ (relative to the thermodynamic formation/decomposition potential for Li_2_O_2_, 2.96 V *vs.* Li^+^/Li), which can also drive parasitic reactions, such as electrolyte oxidation. Therefore, realisation of a practical Li–O_2_ cell necessitates low overpotential charging (and discharging). Another challenge is mitigating potential-independent electrolyte solvent/salt degradation pathways that are initiated by reactive oxygen species (ROS, *e.g.*, singlet oxygen (^1^O_2_), superoxide (O_2_˙^−^), peroxide (O_2_^2−^)) widespread in Li–O_2_ cell chemistry.^4–9^

Solid electrocatalysts have been explored to promote Li_2_O_2_ decomposition.^10,11^ However, such positive electrode-confined species have been shown to catalyse parasitic reactions within the Li–O_2_ cell and are inherently less able to oxidise solid Li_2_O_2_ in poor contact with the electrode and/or catalyst surface.^12^ In contrast, electrolyte soluble catalysts are not limited by this confinement, as they can freely diffuse to where Li_2_O_2_ is located. A widely explored type of electrolyte-soluble catalyst for Li–O_2_ cells are redox mediators (RMs). RMs are electron/hole shuttles that can promote the formation (discharge RMs) and decomposition (charge RMs) of Li_2_O_2_. Discharge/charge RMs operate based on undergoing a reduction/oxidation at the carbon electrode during discharge/charge, followed by the reduced (RM^−^)/oxidised (RM^+^) form carrying out chemical reduction/oxidation of O_2_/Li_2_O_2_. Therefore, in theory, the only electrochemical step is RM reduction/oxidation. For discharge RMs, the redox potential should be as close to, but still below the thermodynamic potential for the O_2_/Li_2_O_2_ couple (2.96 V *vs*. Li^+^/Li). Conversely for charge RMs, the oxidation potential should also be as close to, but slightly above this 2.96 V *vs.* Li^+^/Li value. Cell overpotentials on charge are 2–3-fold greater than the overpotentials on discharge and, as such, utilising charge RMs to decompose Li_2_O_2_ offers a means of significantly reducing charge overpotentials and increasing energy efficiencies. Since the initial reports on organic discharge and charge RMs by Lacey *et al.*^13^ and Chen *et al.*,^14^ respectively, it is now widely accepted that a practical Li–O_2_ cell will require the use of RMs to operate at low overpotentials.^1^

Numerous RMs have been reported which can be categorized into halide-based species,^15–18^ organometallic complexes^19–21^ and organic RMs.^14,22–28^ Most RMs that have been reported to date fall into the organic class due to the synthetic versatility, and thus, tuneability of RM redox potentials. Stable nitroxyl radical-bearing molecules have been shown to be effective organic charge RMs, with Bergner *et al.*^22^ being the first to demonstrate 2,2,6,6-tetramethylpiperidine-1-oxyl (TEMPO) as a charge RM in an Li–O_2_ cell operating with a diglyme-based electrolyte. Compared to identical cells cycled with a halide-based RM (lithium iodide, LiI) and an organic RM (tetrathiafulvalene), a nearly 2-fold increase in cycle life was achieved with the TEMPO-mediated electrolyte.

As for molecular electrolyte solvents where decomposition *via* ROS commonly yields lithium carbonate (Li_2_CO_3_), formate (HCO_2_Li) and acetate (CH_3_CO_2_Li),^4–6,29^ RMs themselves are also susceptible to degradation by ROS-initiated pathways and by reaction with the Li metal negative electrode.^30,31^ Therefore, it is also essential to identify and develop strategies to mitigate RM degradation reactions. Since the introduction of RMs to Li–O_2_ cell chemistry, recent research interest has been directed towards multifunctional RMs that can carry out discharge and/or charge mediation, in addition to other functions such as stabilisation of the Li metal|electrolyte interface and quenching of ROS.^32–36^ Multifunctional RMs also offer the added benefit of simplifying electrolyte formulation, negating the need for multiple additives (each with a singular function) and consideration of the potentially parasitic interactions between each additive.

A variety of multifunctional RMs have been reported for Li–O_2_ cells, which have focused on discharge/charge mediation and facilitating stable SEI formation at Li metal,^32,33,35^ but also more recently included RMs targeting the ability to quench/scavenge O_2_˙^−^.^36,37^ Ionic liquid (IL)-based RMs, wherein a redox-active centre is covalently bound or “tethered” to the IL cation, present an interesting sub-class due to the synthetic versatility of ILs. Zhang *et al.*^32^ reported a TEMPO-functionalised IL bearing an imidazolium-based cation ([Im_TEMPO_][TFSI]) that enhanced discharge capacities and cell rechargeability. The imidazolium moiety was proposed to stabilise O_2_˙^−^, promoting solution-based discharge, as well as stabilising the Li metal|electrolyte interface, which was attributed to electrostatic attraction of the TEMPO-tethered imidazolium cation to the Li metal negative electrode. The authors proposed that this generated a thin SEI and protection layer that mitigated shuttling of the oxidized RM to the negative electrode. In a follow-up study, Tkacheva *et al.*^38^ employed imidazolium-based TEMPO-tethered ILs as RMs and the electrolyte solvent for Li–O_2_ batteries. A similar Li metal stabilisation mechanism was proposed by Yoon *et al.*^33^ for a phenothiazine-tethered IL with an alkyl ammonium cation. In their work, the benefit of the tethering approach was clearly demonstrated in Li–O_2_ cells, as cells operating with the phenothiazine-tethered IL exhibited a two-fold increase in cycle life relative to a cell operating with the free (untethered) phenothiazine RM.

The studies discussed above highlight significant scope for the systematic exploration of tethered RMs based on ILs as multifunctional additives for Li–O_2_ cells. Unlike the work by Yoon *et al.*,^33^ Zhang *et al.*^32^ did not compare the performance of Li–O_2_ cells operating with TEMPO to those cycled with [Im_TEMPO_][TFSI]. This is an important comparison as it highlights the impact of RM diffusivity on charge mediation, as [Im_TEMPO_][TFSI] is a bulkier molecule than TEMPO. Imidazolium-based ILs are known to be more reactive with O_2_˙^−^ and Li metal compared to pyrrolidinium-based ILs,^39–41^ and the latter has been used as an additive to reduce electrolyte volatility and promote RM-mediated charging.^42,43^ Furthermore, ILs bearing 1,4-diazabicyclo[2.2.2]octanium (DABCOnium) as the cation have been shown to quench ^1^O_2_ and reduce parasitic product formation in Li–O_2_ cells.^44^ Therefore, varying cation and anion structure could unlock more chemically robust multifunctional RMs that in turn would result in a greater number of effective mediation cycles. Additionally, comparison of cell performance (discharge capacities, rechargeability, cell lifetime) for Li–O_2_ cells operating with tethered RMs and their untethered equivalents is required to give insight into the true benefit of the tethering strategy as an approach towards multifunctional RMs for Li–O_2_ cells. This is particularly important for multifunctional RMs bearing TEMPO as the redox active centre, as nitroxyl radical-containing RMs exhibit some of the highest apparent rate constants for mediated Li_2_O_2_ oxidation among organic charge RMs reported for Li–O_2_ cells.^45^

In this work, a series of three novel tethered RMs are reported where the cation and anion structure was systematically varied to conclusively answer the question: does the RM tethering strategy impart multifunctionality that benefits Li–O_2_ cell cyclability relative to cells operating with their untethered counterparts? The synthesised RMs incorporate TEMPO as the primary redox-active centre, which is tethered to either an imidazolium-, pyrrolidinium- or DABCOnium-based cation. For the latter two, the anion was [TFSI]^−^, while for the former, a [TFSI]^−^ derivative ([Im_TEMPO_][TFSI], as reported by Zhang *et al.*^32^) and [NO_3_]^−^ derivative were synthesised. The [NO_3_]^−^-containing tethered RM was of particular interest due to the previously reported charge mediating functionality of [NO_3_]^−^.^46,47^ The efficacy and multifunctionality of all tethered RMs were evaluated in diglyme-based electrolytes using galvanostatic Li–O_2_ cell cycling with and without *operando* internal cell pressure measurement, with comparisons made to TEMPO-mediated and unmediated systems. *Ex situ* methods were employed to determine Li_2_O_2_ yield and morphology, and RM degradation was probed by ^1^H NMR and cyclic voltammetry (CV).

## Results and discussion

### Synthesis and structural characterisation of tethered redox mediators

**Fig. 1:**
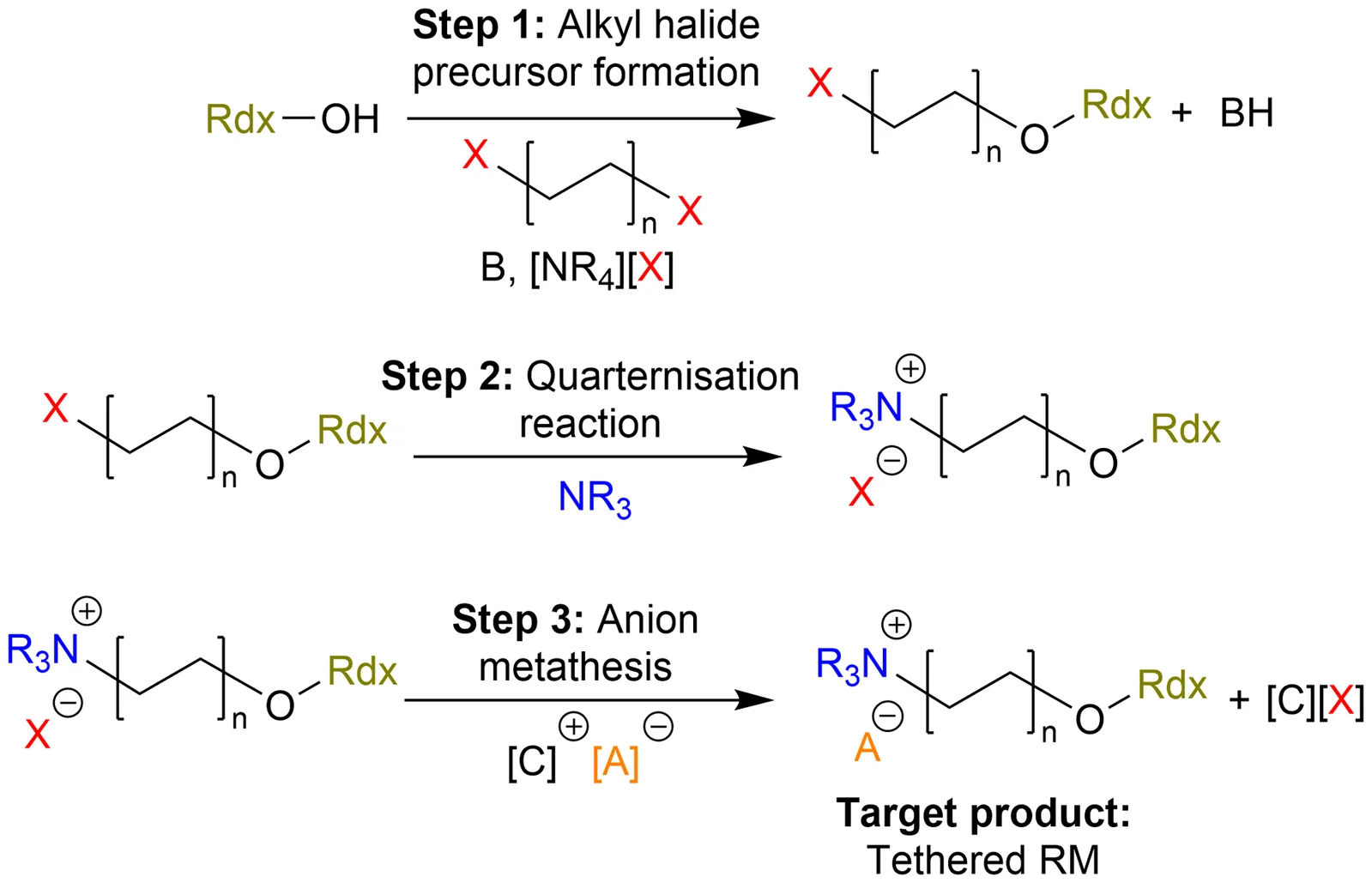
General synthetic route towards tethered redox mediators. Rdx = redox-active moiety, B = base, X = halide (*e.g.*, Br, I), NR_3_ = tertiary amine, C = cation (*e.g.*, Li^+^) and A = anion (*e.g.*, [TFSI]^−^). The Rdx-OH starting material used in step 1 can also be an amine (*i.e.*, Rdx-NH). The length of the alkyl chain (*n*) tethering the redox-active moiety to the cation in the target RM (product of step 3) is determined by the alkyl dihalide used in step 1.

Synthesis of the tethered RMs reported in this work is described in detail in the SI. Briefly, the synthetic route involved (i) synthesis of an alkyl halide precursor bearing the redox active centre, (ii) a quaternization reaction with a tertiary amine to yield the halide salt, and (iii) an anion metathesis reaction to exchange the halide anion for the desired anion in the RM (Fig. 1). Importantly, this synthetic route does not require metal catalysts or pyrophoric reagents, and purification is performed with standard chromatographic techniques, simple washing steps and vacuum drying, making it a readily accessible route towards the RMs reported here.

**Fig. 2:**
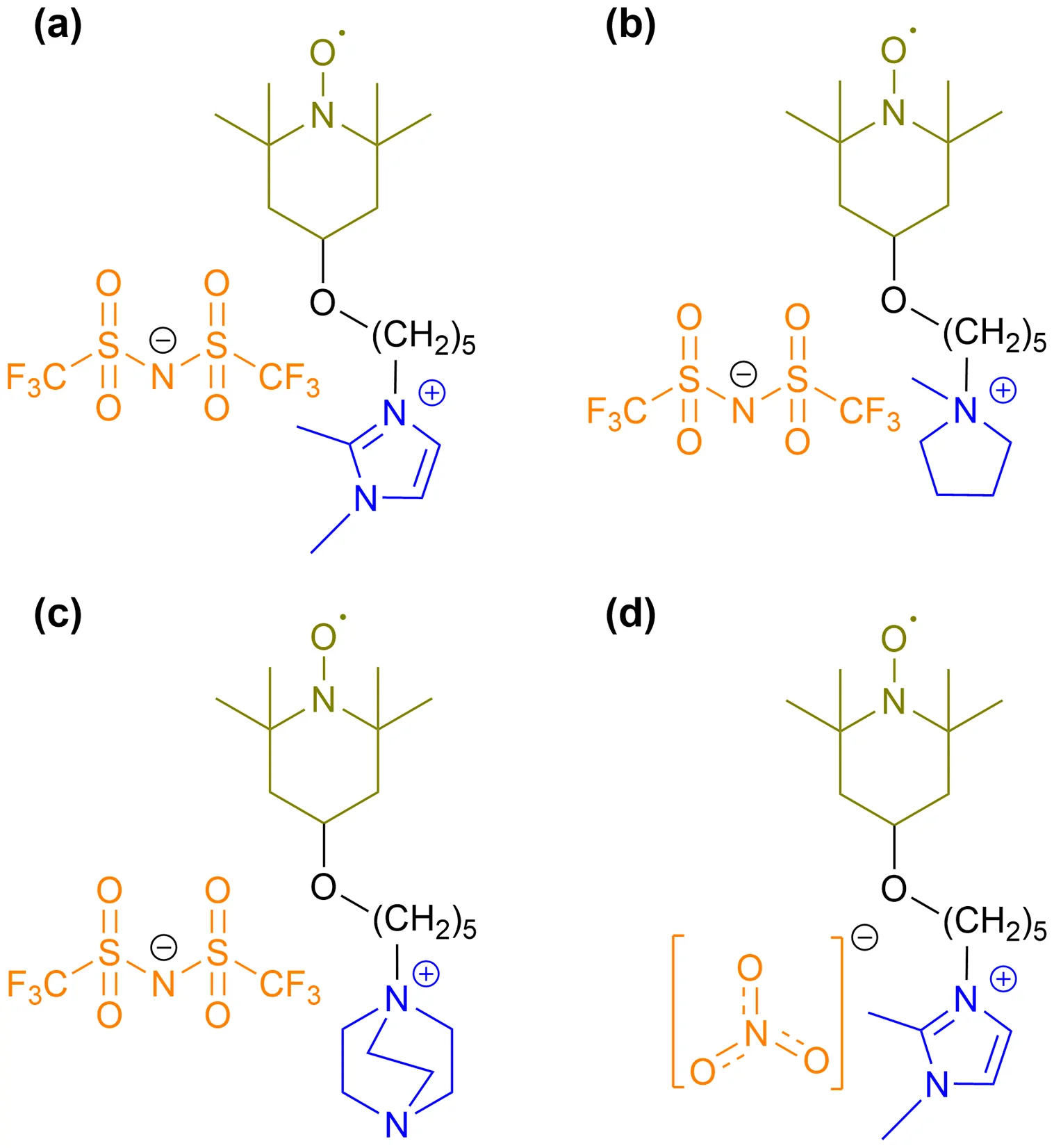
Chemical structures of the four tethered RMs synthesised in this work: (a) 3-(5-((1-oxyl-2,2,6,6-tetramethylpiperidin-4-yl)oxy)pentyl)-1,2-dimethylimidazol-3-ium bis(trifluoromethane)sulfonimide ([Im_TEMPO_][TFSI]). (b) 1-(5-((1-oxyl-2,2,6,6-tetramethylpiperidin-4-yl)oxy)pentyl)-1-methylpyrrolidin-1-ium bis(trifluoromethane)sulfonimide ([Pyrr_TEMPO_][TFSI]). (c) 1-(5-((1-oxyl-2,2,6,6-tetramethylpiperidin-4-yl)oxy)pentyl)-1,4-diazabicyclo[2.2.2]octan-1-ium bis(trifluoromethane)sulfonimide ([D_TEMPO_][TFSI]). (d) 3-(5-((1-oxyl-2,2,6,6-tetramethylpiperidin-4-yl)oxy)pentyl)-1,2-dimethylimidazol-3-ium nitrate ([Im_TEMPO_][NO_3_]).

The structures of the target tethered RMs and their IUPAC chemical names are provided in Fig. 2. Extensive characterization including 1D and 2D NMR focusing on ^1^H and ^13^C nuclei, electrospray ionisation mass spectrometry (ESI-MS), attenuated total reflectance-Fourier transform infrared (ATR-FTIR) spectroscopy, CHN elemental microanalysis, and thermal stability tests (thermogravimetric analysis (TGA) and differential scanning calorimetry (DSC)) was conducted for each of the four RMs. The synthetic routes were scaled up from 0.5 g scale to yield between 5–7 g of [Im_TEMPO_][TFSI], [Pyrr_TEMPO_][TFSI] and [D_TEMPO_][TFSI], and *ca.* 2 g of [Im_TEMPO_][NO_3_] with sufficient purity for further electrochemical testing. All reaction schemes (Schemes S1–S4), synthesis protocols and modifications made to procedures for scaled-up reactions (Table S1), and characterization data (including qualitative confirmation of [NO_3_]^−^ following the anion metathesis to yield [Im_TEMPO_][NO_3_] (Fig. S1), 1D and 2D NMR spectra (Fig. S2–S45), ATR-FTIR spectra (Fig. S46), CHN elemental microanalysis (Table S2), ESI-MS spectra (Fig. S47–S50) and TGA/DSC thermograms (Fig. S51–S53)) are provided in the SI.

### Galvanostatic Li–O_2_ cell cycling and Li_2_O_2_ yield quantification

**Fig. 3:**
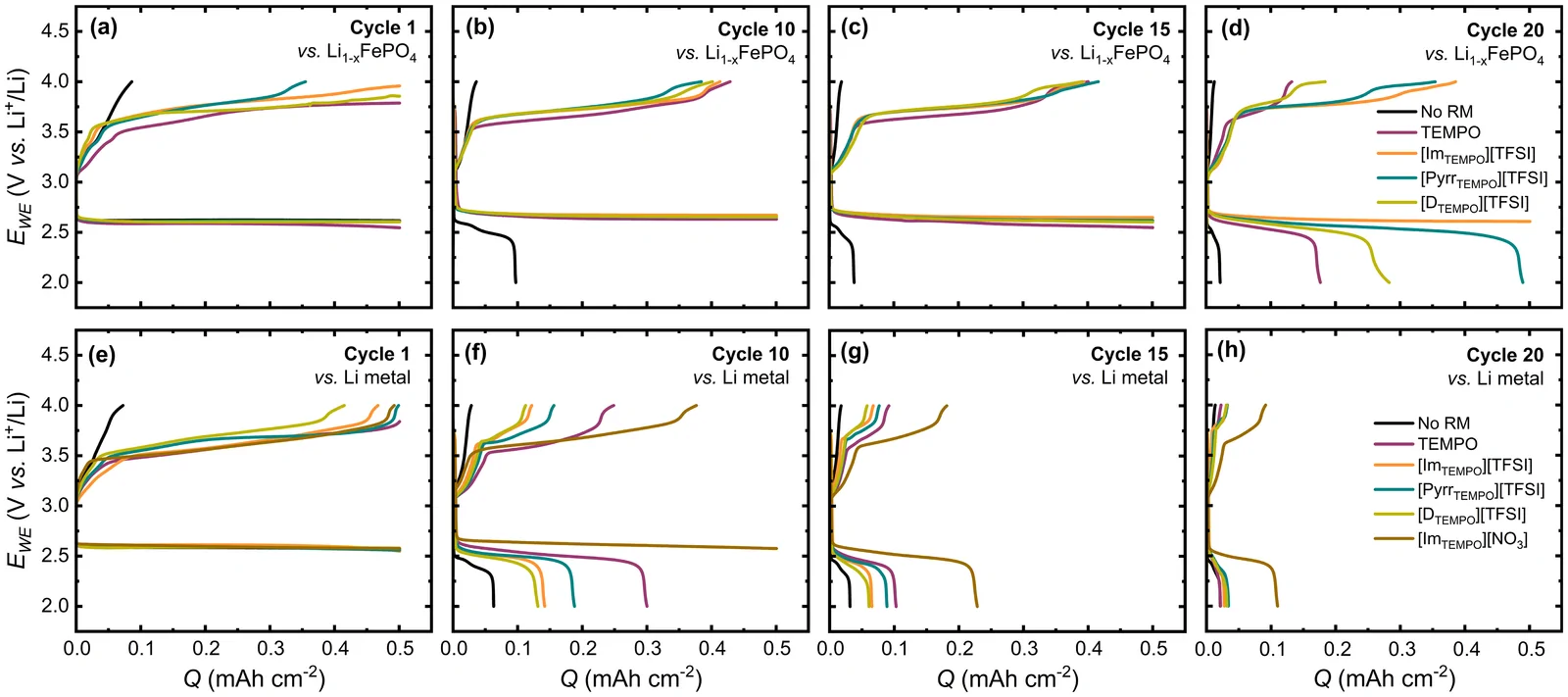
Galvanostatic, capacity-limited cycles (a and e) 1, (b and f) 10, (c and g) 15 and (d and h) 20 of Li–O_2_ cells comprising a carbon paper positive electrode, either (a–d) partially delithiated lithium iron phosphate (Li_1−*x*_FePO_4_) or (e–h) Li metal as the negative electrode and an electrolyte with or without RMs. The base electrolyte was 0.8 mol kg_solvent_^−1^ Li[TFSI] in diglyme and mediated systems contained 25 mmol kg_solvent_^−1^ of either TEMPO, [Im_TEMPO_][TFSI], [Pyrr_TEMPO_][TFSI], [D_TEMPO_][TFSI] or [Im_TEMPO_][NO_3_]. Cells were cycled at a current density of 0.1 mA cm^−2^ to a capacity limit of 0.5 mAh cm^−2^, and discharge and charge potential cut-offs were 2.0 and 4.0 V *vs*. Li^+^/Li, respectively.

The initial galvanostatic cycling experiments were performed in “standard” Li–O_2_ cells (the standard configuration is described in Fig. S54) operating with diglyme-based electrolytes containing either no RM or 25 mmol kg_solvent_^−1^ TEMPO, [Im_TEMPO_][TFSI], [Pyrr_TEMPO_][TFSI] or [D_TEMPO_][TFSI]. Details regarding electrolyte formulation are described in Table S3. In Fig. 3a–d, Li–O_2_ cells were cycled with a carbon paper positive electrode and partially delithiated lithium iron phosphate (Li_1−*x*_FePO_4_) as the negative electrode. Li_1−*x*_FePO_4_ was chosen instead of Li metal as it is a stable, less reactive source of Li^+^ and is widely adopted in the field for mitigating reaction of the RM and/or RM^+^ with the negative electrode.^25,48,49^ Notably, all mediated cells outperform the unmediated cell in terms of rechargeability and discharge capacities. Within the first cycle, the TEMPO-mediated cell exhibits better rechargeability than cells with tethered RMs.

The potential of the charge plateau (*ca.* 3.5–3.7 V *vs*. Li^+^/Li) is consistent with the oxidation of the nitroxyl radical (R_2_N–O˙) in each of the RMs to the oxoammonium cation (R_2_N=O^+^). The R_2_N=O^+^/R_2_N–O˙ redox couple can be readily probed by CV. A detailed description of how CVs were conducted is provided in the Experimental section and SI, including the use of a silver trifluoromethanesulfonate/silver (Ag[OTf]/Ag) reference electrode (RE) for experiments under O_2_. In this case, the procedure for conversion of potentials referenced against Ag[OTf]/Ag to the Li^+^/Li scale is described in Fig. S55. The charge plateaus in Fig. 3 are consistent with the redox potential measured by CV under Ar for the R_2_N=O^+^/R_2_N–O˙ redox couple in each of the RMs (Fig. S56). It should be noted that the accessibility of the R_2_N–O˙/*N*-oxide (R_2_N–O^−^) redox couple, previously reported by Zhang *et al.*^32^ to occur at *ca.* 3.0 V *vs.* Li^+^/Li for [Im_TEMPO_][TFSI], was only observed under certain cell configurations (Fig. S57). This is discussed in detail in the SI (Supporting Discussion 1). Briefly, based on Fig. S57 and the Li–O_2_ cell cycling data (Fig. 3), it is concluded that the R_2_N–O˙/R_2_N–O^−^ redox couple is not accessible for discharge mediation in the RMs studied in this work, as a distinct plateau for reduction of R_2_N–O˙ to R_2_N–O^−^ (at *ca.* 3.0 V *vs*. Li^+^/Li) was not observed during discharge of Li–O_2_ cells, whereas a plateau is observed for the R_2_N=O^+^/R_2_N–O˙ couple during cell charging.

In Fig. 3, initially, charge overpotentials are greater with the tethered RMs relative to TEMPO (at most *ca.* 100 mV difference at 50% of the recharge capacity in cycle 1), irrespective of the negative electrode material used. This is consistent with the *ca.* 40 mV greater redox potential for the tethered RMs compared to TEMPO (see CVs in Fig. S56). This difference in redox potentials is in line with the structures of the tethered mediators; the ether linkage (absent in TEMPO) is electron withdrawing through the inductive effect, and therefore, there is relatively less localised electron density around the R_2_N–O˙ moiety. The higher charge overpotentials in cycle 1 may also be related to the bulkier nature of the tethered RMs, exhibiting diffusion coefficients *ca.* 2.0–2.5-fold lower than TEMPO (as determined by Randles–Sevcik analysis (Fig. S56f and Table S4)). However, the slower diffusivity of the tethered RMs does not limit cell performance. In cells with Li_1-*x*_FePO_4_ negative electrodes, by cycle 20 (Fig. 3d), comparing cells operated with [Im_TEMPO_][TFSI], [Pyrr_TEMPO_][TFSI], or TEMPO, the two tethered RMs deliver almost 3-fold greater capacity on discharge and recharge to between 70–80% of the capacity limit, whereas the TEMPO cell recharges to <30% of this limit. The [D_TEMPO_][TFSI]-containing cell performs comparably to TEMPO. As RM degradation by Li metal can be ruled out due to the use of Li_1−*x*_FePO_4_ counter electrodes in these cells, this data indicates that the DABCOnium-based cation in [D_TEMPO_][TFSI] is more susceptible to ROS-induced degradation compared to the imidazolium- and pyrrolidinium-based cations in [Im_TEMPO_][TFSI] and [Pyrr_TEMPO_][TFSI], respectively.

From the cycling data in Fig. 3a–d, it is demonstrated that the bifunctional benefit of tethered RMs is realised in terms of delivering enhanced discharge capacities and cell rechargeability compared to cell operation with TEMPO (as summarised in Table S5). Although Li_1−*x*_FePO_4_ serves as a useful model electrode to isolate RM effects on positive electrode behaviour, it is not a viable option for a practical negative electrode for Li–O_2_ cells. Ideally, to maximise specific energy, a pure Li metal negative electrode is desirable. Therefore, the above cycling data was repeated with Li metal as the negative electrode (Fig. 3e–h). The cell performances using the TEMPO and tethered-TEMPO electrolytes with a Li metal negative electrode was drastically diminished relative to their counterparts cycled with Li_1−*x*_FePO_4_. This point is further exemplified by the comparable performance between the unmediated cells (black traces, Fig. 3) operating with Li metal and Li_1−*x*_FePO_4_ as the negative electrodes. Comparing mediated cells cycled with Li metal and Li_1−*x*_FePO_4_, it is evident that the loss in cell rechargeability and achievable discharge capacities is more pronounced for cells operating with [Im_TEMPO_][TFSI], [Pyrr_TEMPO_][TFSI] and [D_TEMPO_][TFSI] as compared to TEMPO, suggesting that decomposition of these three tethered RMs by Li metal is significant.

It is important to note that all capacity-limited galvanostatic cycling was performed with a 4.0 V *vs.* Li^+^/Li charge potential cut-off. Although this limit is considerably lower than the limits used in the literature (typically ≥4.2 V *vs.* Li^+^/Li),^22,32,33,35,36,38,50^ it was chosen as all RMs tested in this work have redox potentials below this potential. Therefore, cell cyclability depends to a greater extent on mediated charging, providing conditions better suited to evaluate efficacy of the RM. This contrasts with cases where the charge potential cut-off is ≥4.2 V *vs.* Li^+^/Li and cells charge through a combination of mediated charging and direct electrochemical oxidation. However, even after increasing the charge potential cut-off to 4.5 V *vs.* Li^+^/Li, [Im_TEMPO_][TFSI]- and [Pyrr_TEMPO_][TFSI]-mediated cell discharge capacities and rechargeability were comparable to a cell operating with TEMPO over 25 cycles under the same conditions (Fig. S58). However, consistent with the report by Zhang *et al.*^32^ for [Im_TEMPO_][TFSI], a significant improvement in cell cyclability with Li metal as the negative electrode was achieved for [Im_TEMPO_][TFSI] and [Pyrr_TEMPO_][TFSI] compared to the unmediated cell under a 4.5 V *vs.* Li^+^/Li cut-off.

To improve tethered RM stability with Li metal, an imidazolium-bearing tethered RM was prepared wherein the anion, [TFSI]^−^, was exchanged for [NO_3_]^−^ ([Im_TEMPO_][NO_3_]). This resulted in a significant improvement in cell performance (brown traces, Fig. 3e–h), consistent with the reported Li metal|electrolyte interface stabilising effect of [NO_3_]^−^.^51^ The [NO_3_]^−^ anion has also been shown to promote the ORR due to a high degree of association with Li^+^,^47^ thus allowing stabilised LiO_2_ to diffuse away from the carbon electrode and disproportionate to form platelets of Li_2_O_2_ that grow into toroidal particles.^52^ This promotion of the solution mechanism on discharge leaves more electrochemically active (non-passivated) sites to sustain the discharge reaction, rationalising the enhanced discharge capacities in [Im_TEMPO_][NO_3_]-containing cells operating with a Li metal negative electrode. To understand whether or not the improvement in cycling performance with [Im_TEMPO_][NO_3_] was largely due to stabilisation of the Li metal|electrolyte interface by [NO_3_]^−^, Li–O_2_ cells with Li_1−*x*_FePO_4_ negative electrodes were cycled with [Im_TEMPO_][NO_3_]. With Li metal stabilisation effects eliminated, [Im_TEMPO_][NO_3_]-mediated cells still delivered the best performance (Fig. S59) relative to the other RMs. Therefore, the cell performance enhancement with [Im_TEMPO_][NO_3_] is not purely due to [NO_3_]^−^-derived stabilisation of Li metal.

**Fig. 4:**
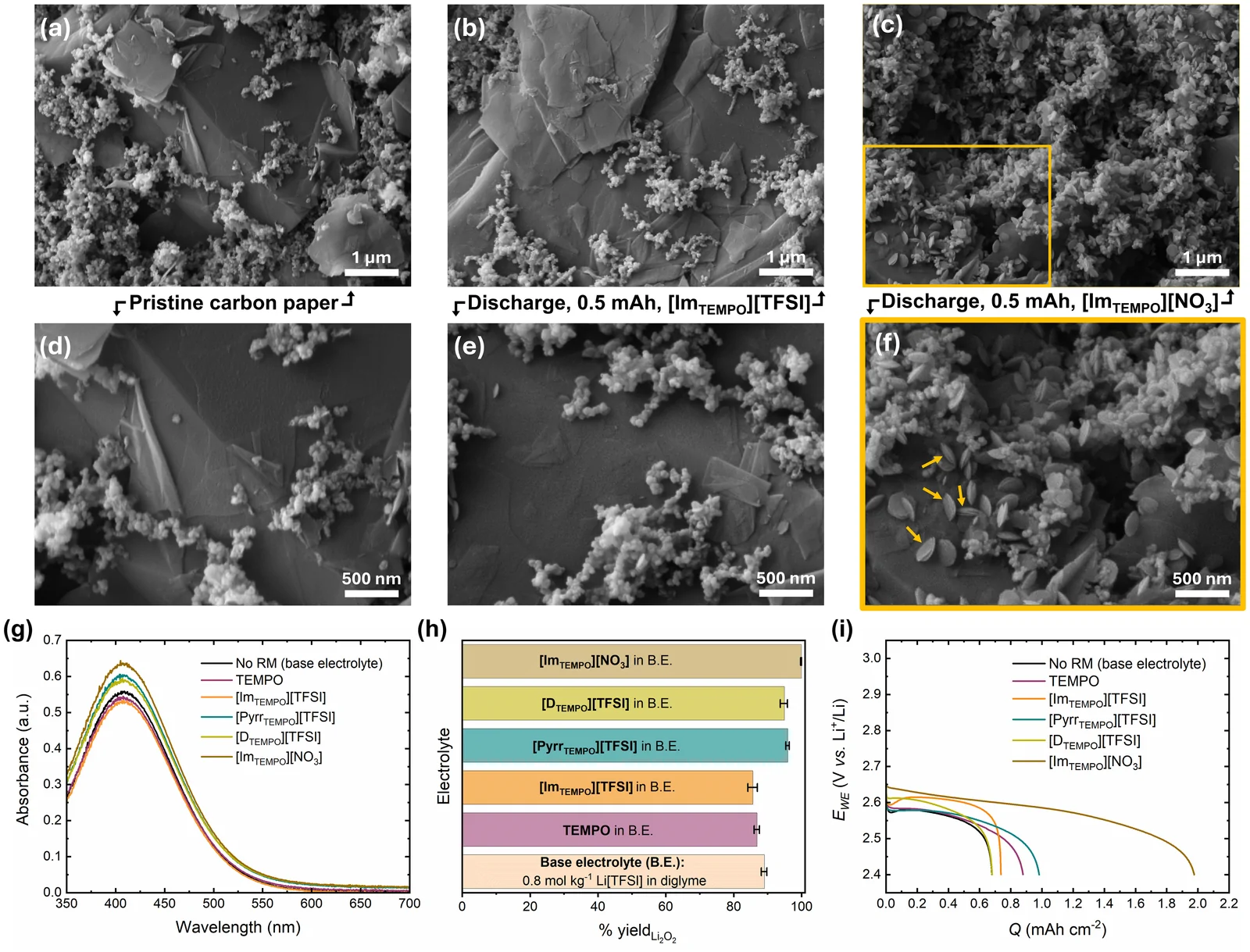
(a–f) SEM micrographs of (a and d) pristine carbon paper and carbon paper after a 0.5 mAh discharge in a base electrolyte (B.E.) comprising 0.8 mol kg_solvent_^−1^ Li[TFSI] in diglyme with (b and e) [Im_TEMPO_][TFSI] and (c and f) [Im_TEMPO_][NO_3_] ((f) is a higher magnification micrograph of the region outlined by the orange rectangle in (c)). The arrows in (f) highlight some regions where stacking of Li_2_O_2_ platelets was observed. Enlarged versions of (c) and (f) are provided in Fig. S60. (g) UV-Vis spectra of aliquots from aqueous solutions containing carbon paper electrodes discharged to 0.5 mAh cm^−2^ (in the B.E. with and without mediators) treated with a 1 : 1 TiOSO_4_ (in H_2_SO_4(aq)_) : H_2_O solution for determination of Li_2_O_2_ yield. (h) Average Li_2_O_2_ yields as a function of electrolyte formulation. Error bars represent absolute uncertainties derived from duplicate measurements in each electrolyte. (i) Full discharge tests (discharge to 2.4 V *vs.* Li^+^/Li) with all RMs investigated in identical electrolytes to those prepared for Li_2_O_2_ yield quantification. All cells were discharged at a current density of 0.1 mA cm^−2^, Li metal was used as the negative electrode and the RM loading was 25 mmol kg_solvent_^−1^.

SEM micrographs of the pristine P50 carbon electrode (Fig. 4a and d) and that discharged with [Im_TEMPO_][TFSI] (Fig. 4b and e) were comparable and indicated no distinct particle deposits. Conversely, for carbon paper positive electrodes discharged with [Im_TEMPO_][NO_3_] (Fig. 4c and f), a high density of platelet-shaped particles with some stacking of platelets was observed. This morphology is characteristic of Li_2_O_2_ formed *via* solution-driven discharge.^52–54^ Enlarged versions of Fig. 4c and f are provided in the SI (Fig. S60). Raman spectroscopic analysis of the carbon electrode discharged with [Im_TEMPO_][NO_3_] confirmed the presence of Li_2_O_2_ (Fig. S61). Quantification of Li_2_O_2_ was performed using the well-established titanium oxysulfate (TiOSO_4_)-based titration method (see Fig. S62 for the calibration curve and the Experimental section for the titration procedure).^25,49^ An exceptionally high Li_2_O_2_ yield was achieved with [Im_TEMPO_][NO_3_] (99.8 ± 0.1%). By comparison, the lower Li_2_O_2_ yields for TEMPO and the other tethered RMs ranged between 85.6 ± 1.4% and 95.8 ± 0.5% (Fig. 4g and h). The difference in yields between [Im_TEMPO_][NO_3_] and the other RMs, as much as nearly 15%, may be related to differences in Li_2_O_2_ reactivity as it grows and/or once it is formed. Chau and co-workers recently demonstrated poor Li_2_O_2_ yields (<90%) despite near-theoretical electron-to-oxygen mole ratios (2e^−^/O_2_, as determined by online electrochemical mass spectrometry) indicating loss of Li_2_O_2_.^9^ In their study, it was concluded that growing Li_2_O_2_ surfaces during cell discharge are prone to reaction with the electrolyte solution. Therefore, additives that can drive Li_2_O_2_ formation selectively, such as [Im_TEMPO_][NO_3_], are crucial for realising high Li_2_O_2_ yield (close to 100%) Li–O_2_ batteries and highlights the need for RMs that could play a role in modulating Li_2_O_2_ surface reactivity.

Consistent with the morphology of Li_2_O_2_ in cells discharged with [Im_TEMPO_][NO_3_], the highest maximum (non-capacity limited) discharge capacity (*ca.* 2 mAh cm^−2^) was achieved with this RM when discharging cells to 2.4 V *vs.* Li^+^/Li (Fig. 4i). Terminal discharge capacities followed the order [Im_TEMPO_][NO_3_] > [Pyrr_TEMPO_][TFSI] > TEMPO > [Im_TEMPO_][TFSI] > [D_TEMPO_][TFSI] ≈ no RM. The 33% increase in discharge capacities with [Pyrr_TEMPO_][TFSI] compared to [Im_TEMPO_][TFSI] is consistent with the reported greater lifetime of O_2_˙^−^ in pyrrolidinium-based ILs compared to imidazolium-based ILs,^39,40^ thus promoting solution-based (O_2_˙^−^ disproportionation-driven) discharge to yield Li_2_O_2_. The ability of TEMPO, [Im_TEMPO_][TFSI], [Pyrr_TEMPO_][TFSI] and [D_TEMPO_][TFSI] to promote solution-driven discharge is also likely to be limited by decomposition of these RMs by Li metal. This reactivity is mitigated with [Im_TEMPO_][NO_3_], further supported by Li|Li symmetric cell cycling data discussed later, demonstrating the benefit of additive-level loadings of [NO_3_]^−^ in both driving platelet-like Li_2_O_2_ formation and stabilising the Li metal|electrolyte interface.

A table comparing various cell performance metrics (discharge/charge overpotentials, cycle life, Li_2_O_2_ yield, maximum discharge capacity and RM diffusion coefficient) for the RMs reported in this work and identical/related RMs reported in the literature is provided in the SI (Table S6). However, it is worth noting that drawing conclusions on performance of various RMs by comparing metrics across a range of studies is complicated by the large variability in Li–O_2_ cell configurations, components and cycling parameters, and underreporting of key experimental information. Therefore, it is recommended that the metal–air battery research community work towards an agreed standardisation of cell components and cell testing protocol to easily identify advancements in RMs for Li–O_2_ cells.

The [NO_3_]^−^ anion has also been reported to exhibit charge mediating functionality by first undergoing reduction at Li metal to nitrite ([NO_2_]^−^), which is subsequently electrochemically oxidised to nitrogen dioxide (NO_2_) at the carbon electrode. NO_2_ can then oxidise Li_2_O_2_, re-forming [NO_2_]^−^ to complete the catalytic cycle.^46^ However, in this work it was observed that charge mediation is primarily achieved through the R_2_N=O^+^/R_2_N–O˙ redox couple (Fig. S63). One of the benefits of incorporating [NO_3_]^−^, even at additive-level loadings, is that it has a marked effect on Li metal|electrolyte interfacial stability, as demonstrated by Li|Li symmetric cell cycling (Fig. S64). Symmetric cells containing TEMPO, [Im_TEMPO_][TFSI], [Pyrr_TEMPO_][TFSI] and [D_TEMPO_][TFSI] all showed a rapid rise in Li stripping/plating overpotential within 300–400 h. In contrast, the symmetric cells operating with [Im_TEMPO_][NO_3_] exhibited significantly lower overpotential cycling over the 1000 h test period, consistent with the stable Li metal SEI formation achieved with nitrate-based salts.^51^ The reactivity of [Im_TEMPO_][TFSI] with Li metal is contrary to work by Zhang *et al.*,^32^ who showed superior Li|Li symmetric cell stability of [Im_TEMPO_][TFSI] relative to a cell cycled with TEMPO over a 600 h test period. However, the instability of imidazolium-based ILs in contact with Li metal has been reported before and shown to be due to decomposition to highly reactive N-heterocyclic carbenes.^41^

The improvement in cell cyclability with [Im_TEMPO_][NO_3_] was further validated in Li–O_2_ cells by increasing the RM loading from 25 to 135 mmol kg_solvent_^−1^ in cells cycled with TEMPO, [Im_TEMPO_][TFSI] and [Im_TEMPO_][NO_3_] (Fig. S65). Cells containing 135 mmol kg_solvent_^−1^ [Im_TEMPO_][TFSI] and [Im_TEMPO_][NO_3_] exhibited enhanced discharge capacities and cell rechargeability across cycles 1, 5 and 10 compared to their 25 mmol kg_solvent_^−1^ RM counterparts. However, by cycle 15, low and high loading [Im_TEMPO_][TFSI]-mediated cells have similar discharge–charge profiles irrespective of the RM concentration. In contrast, the cells cycled with [Im_TEMPO_][NO_3_] gave significantly better performance beyond cycle 5 while the performance of the cell with a higher concentration of [Im_TEMPO_][NO_3_] is enhanced further, delivering the capacity limit (0.5 mAh cm^−2^) on discharge and recharging to >90% of this limit at cycle 15. Additionally, it is worth noting that in cycle 10, cells with both [Im_TEMPO_][TFSI] and [Im_TEMPO_][NO_3_] deliver higher discharge capacities and improved rechargeability at the higher loadings (Fig. S65g) – a distinctly different response compared to cells cycled with TEMPO, wherein higher loading cell performance was poorer compared to the lower loading counterpart (Fig. S65c). For [Im_TEMPO_][NO_3_], the improved performance at the higher loading can be rationalised by [NO_3_]^−^ driving platelet-like Li_2_O_2_ growth and stabilising the Li metal|electrolyte interface. Similarly, higher loadings of [Im_TEMPO_][TFSI] can improve discharge capacities and cell rechargeability. However, this is not sustained, as both lower and higher loading [Im_TEMPO_][TFSI] cells exhibit comparable performance by cycle 15 (Fig. S65h, orange traces). These results highlight the poorer stability of [Im_TEMPO_][TFSI] at higher loadings and the benefit of increasing the [Im_TEMPO_][NO_3_] loading on Li–O_2_ cell cyclability.

From the data discussed thus far, it is evident that RM tethering can enhance positive electrode electrochemistry within Li–O_2_ cells. However, other strategies towards multifunctional RMs have been reported in the literature, largely based on halide based ILs/salts wherein the anion (iodide or bromide) exhibits charge RM functionality and the cation facilitates Li metal protection.^35,55–59^ However, in the tethering strategy there is scope to tune the redox potential of the tethered organic redox moiety through structural variations to the cation component of the tethered RM, whereas such redox potential tuning is not possible in halide-based ILs. This redox potential sensitivity is demonstrated for the tethered RMs, which exhibit a redox potential *ca.* 40 mV greater than free TEMPO (Table S4) due to the ether linkage in the tethered RMs. Moreover, the diffusion coefficient of the RM can be altered by changing the chain length of the covalent tether and the nature of the tethered moiety can be varied as well. In this work, the tethered TEMPO moiety mediates cell charging, but there is also the possibility of tethering discharge RMs, such as some of the amine-containing triarylmethyl-based RMs recently reported by Askins *et al.*^60^ Besides halide-based multifunctional RMs incorporating ammonium-based salts,^61^ organic aromatic molecules^34,62^ and organometallic complexes^19–21^ have also been reported, which also offer significant synthetic versatility.

Comparison of the cell performance improvements from the various multifunctional RMs reported is complicated by the variability in cell configurations, components and cycling parameters, and unreported experimental parameters, as discussed earlier. In many of the above referenced studies, cycle lives on the order of a few tens to hundreds of cycles are reported, but with positive electrodes comprising some form of high specific surface area (SSA) carbon (*e.g.*, carbon black (Ketjenblack and Super P) or carbon nanotubes (CNTs)). Therein, the carbon loading and electrode diameters range from 0.10–0.75 mg and 7–16 mm, respectively. Hence, the pre-determined capacity limit and current density for cycle life assessment do not exceed 0.5 mAh cm^−2^ and 0.26 mA cm^−2^, respectively. It is important to note here that these values are normalised by the geometric area of the electrode, and so for relatively large SSA electrodes (*e.g.*, Super P or Ketjenblack EC-600JD with SSAs exceeding 60 and 1000 m^2^ g^−1^, respectively),^63,64^ the true areal (SSA-normalised) capacity and current density will be considerably smaller than the corresponding values for a carbon paper electrode (*e.g.*, the SSA of Avcarb P50 carbon paper used in this work is *ca.* 9 m^2^ g^−1^).^65^ Therefore, while higher SSA electrodes are considered a more practical positive electrode material for Li–O_2_ cells than carbon paper, when setting capacity limits and current densities for cycle life tests, these should be commensurate with the SSA of the electrode material used. Table S7 summarises the key differences in parameters between the previously mentioned studies and this work.

**Fig. 5:**
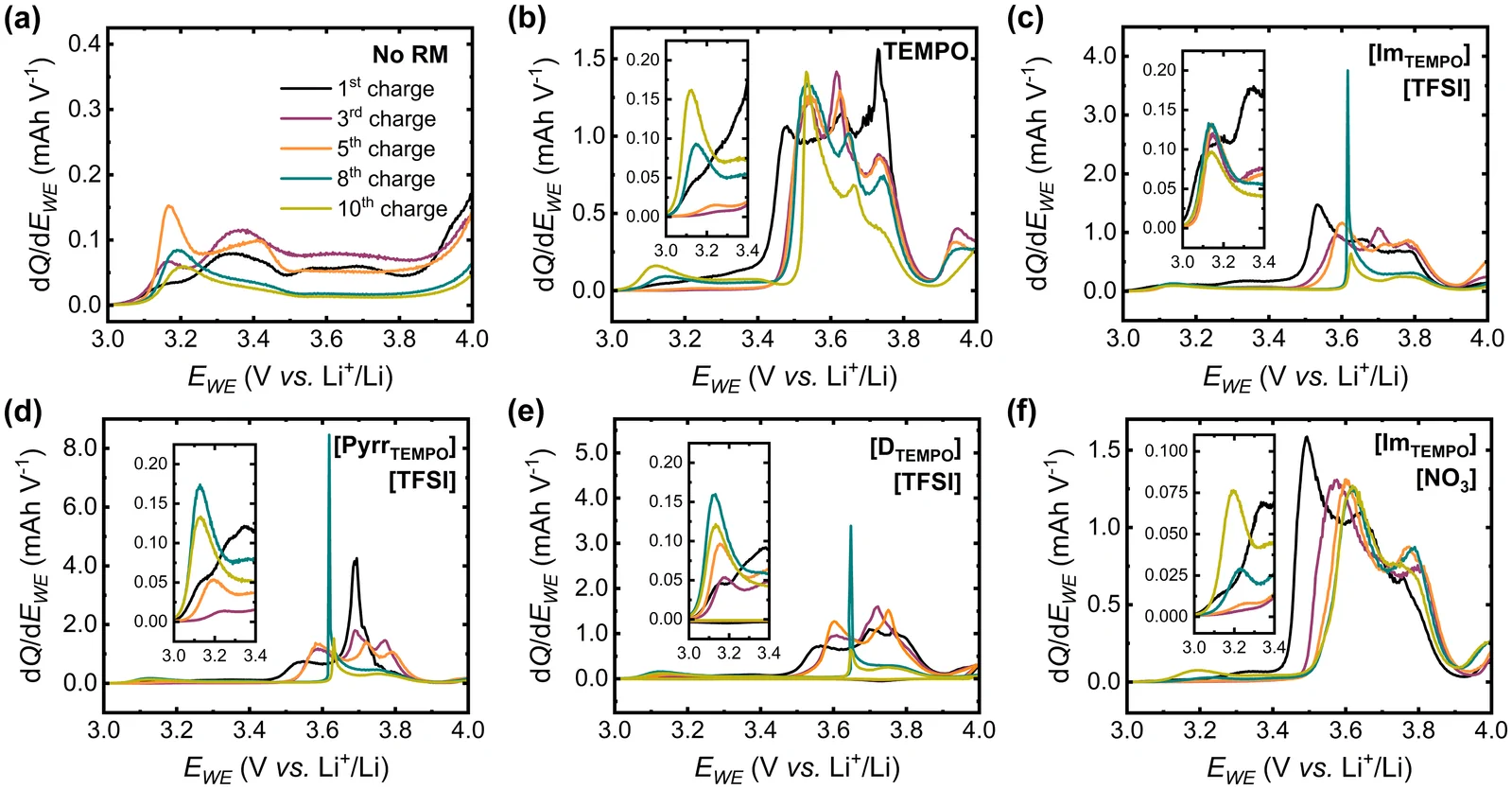
Differential capacity (d*Q*/d*E*_WE_) plots for a series of charge half-cycles in standard Li–O_2_ cells comprising a carbon paper positive electrode, Li metal negative electrode and an electrolyte (a) without a RM and with either (b) TEMPO, (c) [Im_TEMPO_][TFSI], (d) [Pyrr_TEMPO_][TFSI], (e) [D_TEMPO_][TFSI] or (f) [Im_TEMPO_][NO_3_] in 0.8 mol kg_solvent_^−1^ Li[TFSI] in diglyme. The RM loading was 25 mmol kg_solvent_^−1^. The insets zoom into a potential window between 3.0 and 3.4 V *vs.* Li^+^/Li highlighting the evolution of a d*Q*/d*E*_WE_ peak at *ca.* 3.2 V *vs.* Li^+^/Li over a series of charge half-cycles. The capacity was limited to 0.5 mAh cm^−2^, the current density was 0.1 mA cm^−2^ and the potential cut-offs were 2.0 and 4.0 V *vs.* Li^+^/Li on discharge and charge, respectively.

Upon closer inspection of the cycling data presented in Fig. 3, an indicator of cell failure can be discerned; cell failure closely follows once the onset of charge occurs at *ca.* 3.2 *vs*. Li^+^/Li. This trend appears irrespective of the type of negative electrode used (Li metal or Li_1−*x*_FePO_4_) and was also observed in cycling with higher RM loadings (see Fig. S65). Critically, a potential of 3.2 V *vs.* Li^+^/Li is below the potential plateau corresponding to electrochemical RM oxidation, which suggests that charge is being passed to electrochemically oxidise Li_2_O_2_. Given an overarching aim to reduce charging overpotentials, this being an issue may be counterintuitive. However, once this response is observed, cells typically failed to deliver the discharge capacity limit within the next 1–2 cycles. It is proposed that this response can be taken as an indicator of imminent cell failure, defined as the cell no longer being able to deliver the discharge capacity-limit within the pre-determined discharge potential cut-off. The onset of charging at potentials below the RM oxidation potential can be discerned by plotting the first derivative of capacity with respect to potential (d*Q*/d*E*_WE_) as a function of potential over a series of charge half-cycles (Fig. 5).

In the unmediated cell, the d*Q*/d*E*_WE_ plots show the growth of a peak at *ca.* 3.2 V *vs.* Li^+^/Li from the 1^st^ to the 5^th^ charge half-cycle, consistent with electrochemical oxidation of Li_2_O_2_. It has been shown that low overpotential (<400 mV) charging in unmediated cells is associated with formation of a lithium-deficient phase (Li_2−*x*_O_2_) and oxidation of amorphous and/or thin films of Li_2_O_2_, where oxidation of amorphous Li_2_O_2_ is more facile compared to crystalline Li_2_O_2_.^66–68^ As the cell starts to fail, where little to no charge is passed and the cell is primarily polarising, the magnitude of the d*Q*/d*E*_WE_ peak at *ca.* 3.2 V *vs.* Li^+^/Li decreases from the 5^th^ to the 10^th^ charge half-cycle. In cells operated with RMs, initially, d*Q*/d*E*_WE_ peaks corresponding to the potential at which electrochemical RM oxidation occurs dominate the profile. This is consistent with the cell charging primarily *via* RM-driven chemical oxidation of Li_2_O_2_. However, in later charge half-cycles, a similar d*Q*/d*E*_WE_ peak at *ca.* 3.2 V *vs.* Li^+^/Li starts to grow. The growth of this peak coincides with cell failure, suggesting that the cell charging mechanism transitions from primarily RM-driven oxidation of Li_2_O_2_ to mixed (RM-driven and electrochemical) oxidation of Li_2_O_2_. The magnitude of the d*Q*/d*E*_WE_ peak at *ca.* 3.2 V *vs.* Li^+^/Li in the [Im_TEMPO_][NO_3_]-mediated cell is *ca.* 2-fold smaller than in cells operated with the other RMs. This suggests a suppression of the aforementioned mechanistic change in cell charging when [Im_TEMPO_][NO_3_] is employed (*i.e.*, the cell charges for more half-cycles *via* primarily RM-driven oxidation of Li_2_O_2_), further rationalising the enhanced rechargeability with this RM in Li–O_2_ cells with Li metal as the negative electrode.

### Galvanostatic Li–O_2_ cell cycling with *operando* pressure measurement

The galvanostatic cycling profiles enable inferences to be made regarding the dominant electrochemical reactions proceeding on discharge and charge. However, they do not provide explicit evidence for any chemical processes taking place. This is particularly important for mediated charging of Li–O_2_ cells, wherein a coupled chemical process occurs (electrochemical oxidation of the RM to RM^+^ followed by RM^+^-driven chemical oxidation of Li_2_O_2_ to evolve O_2_ and Li^+^ (and regenerating RM)). For this purpose, *operando* pressure measurements can give insight into Li–O_2_ cell operation and provide a readily accessible means of assessing RM efficacy within the Li–O_2_ cell.^69,70^ The differential capacity analysis shown in Fig. 5 can be paired with the cell pressure response to track RM efficacy over a series of charge half-cycles, as we have reported previously for TEMPO-containing sulfolane- and diglyme-based electrolytes.^70^

**Fig. 6:**
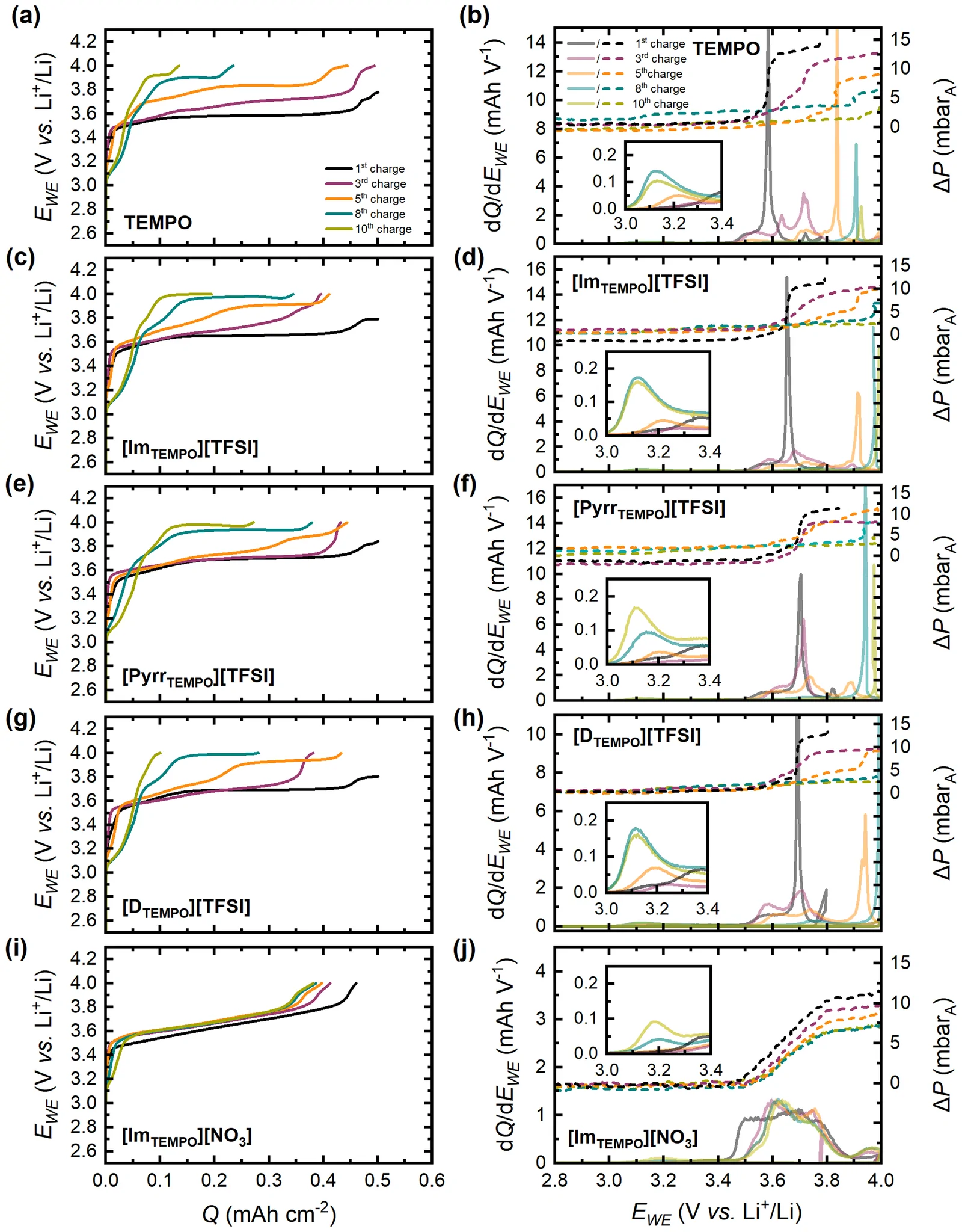
(a, c, e, g and i) Galvanostatic charging profiles and (b, d, f, h and j) differential capacity (d*Q*/d*E*_WE_, solid lines) and change in pressure (Δ*P*, dotted lines) as a function of cell potential during the 1^st^, 3^rd^, 5^th^, 8^th^ and 10^th^ charge *in operando* pressure cells. The insets show the d*Q*/d*E*_WE_ profiles over a narrower potential window to highlight the growth of a d*Q*/d*E*_WE_ peak at *ca.* 3.2 V *vs.* Li^+^/Li. The electrolytes comprised 0.8 mol kg_solvent_^−1^ Li[TFSI] in diglyme with (a and b) TEMPO, (c and d) [Im_TEMPO_][TFSI], (e and f) [Pyrr_TEMPO_][TFSI], (g and h) [D_TEMPO_][TFSI] and (i and j) [Im_TEMPO_][NO_3_]. The RM loading was 25 mmol kg_solvent_^−1^. The pressure at the start of each charge half-cycle is defined as Δ*P* = 0 and the average gas leak rate over a *ca.* 24 h rest period at open circuit potential was (3.4 ± 2.3) × 10^−5^ bar h^−1^. The leak rate for each cell assembled was determined and used to correct the measured pressure response, as described in Fig. S67.

Fig. 6 shows the charge profiles for 5 charge half-cycles and the corresponding cell pressure response (Δ*P*) overlaid with the d*Q*/d*E*_WE_ profile for Li–O_2_*operando* pressure cells cycled with Li metal negative electrodes operating with TEMPO and the tethered RMs. A description of the system is provided in Fig. S66 and the cell volume calibration procedure was detailed in previously published work.^70^ The leak rate in the cell was accounted for by measuring the pressure drop over a *ca.* 24 h rest period at open circuit potential for each experiment prior to galvanostatic cycling. A representative example of how the leak rate was used to correct the measured pressure response is described in Fig. S67. Gas consumption and evolution rates can be determined with knowledge of the cell headspace volume and compared to their theoretical values for the desired two-electron ORR/OER. The potential profile, cell pressure response, and gas consumption/evolution rate (first derivative of the pressure response) as a function of time for all RMs examined in this work is presented in Fig. S68 and S69. Qualitatively, the pressure measurements clearly indicate an increase in cell pressure during the RM oxidation plateau on charge, providing explicit confirmation of a coupled chemical process occurring (Fig. S68). Within a pure O_2_ atmosphere, deviation from the ideal gas consumption/evolution rate (2e^−^/*n*(O_2_) approximated as 2e^−^/*n*_gas_) is an indication of parasitic chemistry taking place within the cell. Considering the gas consumption/evolution profiles in cycles 1–3 (Fig. S68), discharge proceeds at close to the 2e^−^/*n*_gas_ rate, whereas charge occurs at rates consistent with a >2e^−^/*n*_gas_ process, indicating that parasitic chemistry dominates on charge. In cycles 4–6 (Fig. S69), there is a distinct shift in the gas evolution rate closer to the 4e^−^/*n*_gas_ process for all RMs, although this shift is not as significant for the cell cycled with [Im_TEMPO_][NO_3_], further rationalizing the improved rechargeability with this RM compared to the others. In Fig. 6, for all RMs in the first charge half-cycle, a sharp rise in gas evolution (Δ*P*) is observed at the redox potential corresponding to electrochemical RM oxidation (as seen by the overlap between the peak in the d*Q*/d*E*_WE_ profile at the RM oxidation potential and the sharp increase in cell pressure). This further supports the fact that a coupled reaction takes place, as expected for electrochemical RM oxidation followed by RM^+^-mediated chemical oxidation of Li_2_O_2_ to evolve O_2_ gas. However, in all RMs except for [Im_TEMPO_][NO_3_], this sharp rise in gas evolution is not sustained over the 3^rd^, 5^th^, 8^th^ and 10^th^ charge half-cycles. Instead, the charge plateaus are progressively shifted to higher potentials with each cycle, which coincides with gas evolution being spread over a wider potential window and a marked decrease in cell pressure rise during charge steps resulting from incomplete charging due to the upper voltage limit being hit prematurely.

In contrast, with [Im_TEMPO_][NO_3_], although the cell pressure rise decreases each cycle, this decrease is not as significant relative to the other RMs. Furthermore, the sharp rise in gas evolution is sustained from the 1^st^ to the 10^th^ charge half-cycle and is centred over the d*Q*/d*E*_WE_ peak corresponding to [Im_TEMPO_][NO_3_] oxidation. Therefore, RM efficacy is maximised when gas evolution during cell charging is centred around the d*Q*/d*E*_WE_ peak corresponding to mediator oxidation. The broadening of the gas evolution response over a wider potential window, coupled with attenuation of the d*Q*/d*E*_WE_ peak associated with RM oxidation, is an indication of loss of RM efficacy. This criterion for RM efficacy was established in our prior work for a different electrode–electrolyte combination,^70^ which highlights its general applicability as a diagnostic approach for mediated Li–O_2_ cell performance.

Furthermore, the growth of the d*Q*/d*E*_WE_ peak at *ca.* 3.2 V *vs.* Li^+^/Li noted from the differential capacity analysis in standard Li–O_2_ cells (Fig. 5) is also apparent in the pressure cell. The growth of this peak was associated with cell failure in standard cells, and this is corroborated in terms of the gas evolution response, as the growth of this d*Q*/d*E*_WE_ peak coincides with spreading of the cell pressure increase over a wider potential window. It was suggested that the growth of the d*Q*/d*E*_WE_ peak at *ca.* 3.2 V *vs.* Li^+^/Li in standard Li–O_2_ cells was related to a change in the charging mechanism from primarily RM-driven Li_2_O_2_ oxidation to mixed (RM-driven and direct electrochemical) oxidation of Li_2_O_2_. This is further supported by the *operando* pressure data upon examining the change in the Δ*P* and d*Q*/d*E*_WE_ profiles as functions of potential within a potential window of 2.8 to 3.4 V *vs.* Li^+^/Li (Fig. S70). There is a clear increase in Δ*P* that coincides with the potential of *ca.* 3.2 V *vs.* Li^+^/Li at which the d*Q*/d*E*_WE_ peak appears (in cycle 5) and grows (cycles 8 and 10) for all RMs. The observed gas evolution at this potential suggests that it is associated with direct electrochemical oxidation of Li_2_O_2_ to evolve O_2_ gas. Moreover, the increase in Δ*P* as the d*Q*/d*E*_WE_ peak at 3.2 V *vs.* Li^+^/Li grows is considerably smaller for [Im_TEMPO_][NO_3_] compared to the other RMs, supporting the fact that charging *via* RM-driven Li_2_O_2_ oxidation can be sustained for a greater number of cycles, further rationalising the improved cyclability with this RM.

The differential capacity–pressure analysis shown in Fig. 6 can be applied to study any charge mediator. This approach can also be applied to the study of the mediated ORR with discharge RMs, provided that a distinct plateau can be ascribed to discharge RM reduction, which can then be related to a pressure drop as a result of gas consumption within the corresponding potential window. However, it is important to note that *operando* pressure measurements do not offer explicit chemical information on gas composition. Online electrochemical mass spectrometry (OEMS) does offer this capability but is typically only applied to a single cycle or half-cycle for the study of Li–O_2_ cell chemistry and requires bespoke cell design. In contrast, *operando* pressure measurements can be performed for the duration of the cell's lifetime. Furthermore, the pressure cell design reported in this work (Fig. S66) enables the flooding factor, defined as the ratio of electrolyte volume to active electrode area of the positive electrode, to be identical between the standard Li–O_2_ cell and the pressure cell. Therefore, *operando* pressure measurements provide a more accessible means of screening various RMs for their efficacy within the Li–O_2_ cell, after which promising candidate mediators can be further investigated by OEMS.

### Degradation of tethered redox mediators during electrochemical cycling

**Fig. 7:**
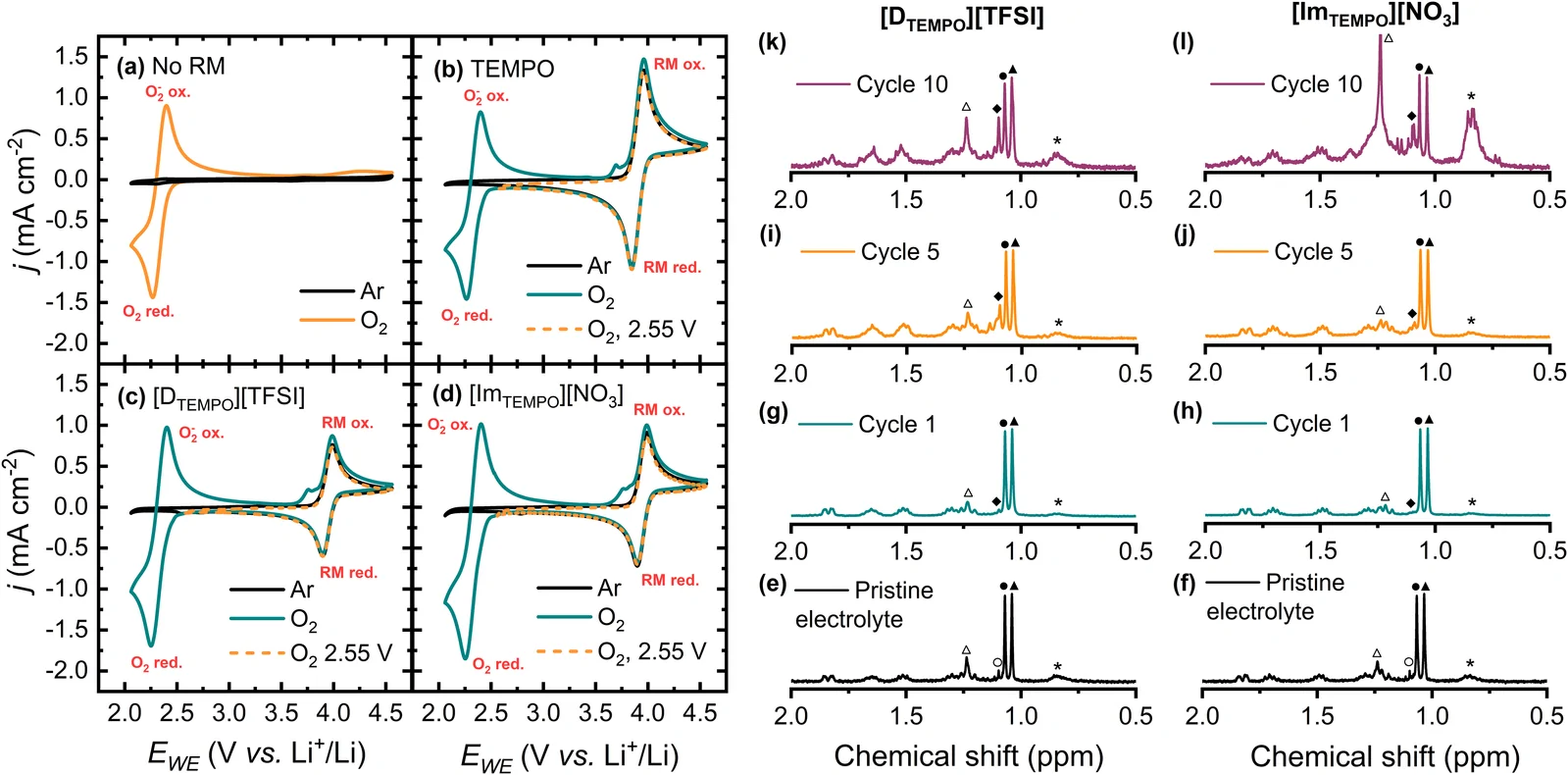
CVs in 1 M [Pyrr_13_][TFSI] in diglyme either (a) without a RM or with 10 mM (b) TEMPO, (c) [D_TEMPO_][TFSI] or (d) [Im_TEMPO_][NO_3_], containing no Li-salt. CVs were conducted under both Ar and O_2_ atmospheres at a scan rate of 50 mV s^−1^ in a three-electrode glass cell comprising a glassy carbon working electrode, Pt counter electrode and Ag[OTf]/Ag reference electrode. (e–l) ^1^H NMR spectra in DMSO-d_6_ of the pristine (e) [D_TEMPO_][TFSI]-containing and (f) [Im_TEMPO_][NO_3_]-containing electrolytes and the same electrolyte compositions after (g and h) 1, (i and j) 5 and (k and l) 10 cycles in Li–O_2_ cells with Li metal as the negative electrode. The electrolyte in (e–l) was 25 mmol kg_solvent_^−1^ RM (either [D_TEMPO_][TFSI] or [Im_TEMPO_][NO_3_]) in 0.8 mol kg_solvent_^−1^ Li[TFSI] in diglyme. Spectra are shown within a 0.5 to 2.0 ppm chemical shift window, highlighting peaks within [D_TEMPO_][TFSI] and [Im_TEMPO_][NO_3_] corresponding to the tethered TEMPO moiety (●, ▲) and the alkyl chain (peaks between 1.2 and 2 ppm). Peak △ and * originate from DMSO-d_6_ and peak ○ (in the pristine electrolytes) is from diglyme based on comparisons with the control ^1^H NMR spectra (Fig. S73). Peak △ overlaps with peaks corresponding to the alkyl chain in the RMs (see control ^1^H NMR spectra (Fig. S73) and those of the pure RMs (Fig. S29 and S37)). The peak at 1.10 ppm (◆) grows on cycling and is associated with RM degradation. Spectra are normalized by the intensity of peak ▲.

A key intermediate in Li–O_2_ cells is lithium superoxide (LiO_2_), wherein O_2_˙^−^ can act as a nucleophile, engaging in hydrogen abstraction of organic solvents used in Li–O_2_ chemistry.^4,5,29^ Such a decomposition mechanism has been proposed for ethers in early work by Freunberger *et al.*,^4^ ultimately leading to the formation of parasitic species such as HCO_2_Li, CH_3_CO_2_Li and Li_2_CO_3_. Nucleophilic attack by O_2_˙^−^ could represent a decomposition pathway for the tethered RMs reported in this work. In this section, the stability of the R_2_N=O^+^/R_2_N–O˙ redox couple was investigated in the presence of O_2_˙^−^. In the presence of a Li salt, upon one-electron reduction of O_2_ to LiO_2_, LiO_2_ will readily disproportionate to give Li_2_O_2_ (and liberate O_2_). It is well known that the O_2_/O_2_˙^−^ redox couple is reasonably chemically reversible in certain organic electrolytes with large organic cations in the conductive salt.^71^ Therefore, to investigate RM stability in the presence of O_2_˙^−^, cyclic voltammetry (CV) in diglyme-based electrolytes without a RM or with either TEMPO, [D_TEMPO_][TFSI] or [Im_TEMPO_][NO_3_] was conducted with an IL, 1-propyl-1-methylpyrrolidinium [TFSI] ([Pyrr_13_][TFSI]), as the supporting electrolyte. [D_TEMPO_][TFSI] and [Im_TEMPO_][NO_3_] were selected as they were the poorest and best performing RMs, respectively, among the tethered RMs used in Li–O_2_ cells cycled with Li_1−*x*_FePO_4_ and Li metal negative electrodes. This enabled the study of R_2_N=O^+^/R_2_N–O˙ redox in the presence of the O_2_/O_2_˙^−^ redox couple (Fig. 7a–d). See the Experimental section and the SI (Fig. S55) for detailed information on the reference electrodes and reference potentials used in these CV experiments.

In the CVs without an RM under an O_2_ atmosphere, a single redox process is apparent, consistent with the O_2_/O_2_˙^−^ redox couple, which is chemically reversible in 1 M [Pyrr_13_][TFSI] in diglyme (redox potential of 2.33 V *vs.* Li^+^/Li, Fig. 7a). No other distinct peaks/features can be observed in the CV. However, after the addition of TEMPO to this system, aside from the peaks corresponding to the TEMPO^+^/TEMPO and O_2_/O_2_˙^−^ redox couples, a small oxidation peak at *ca.* 3.70 V *vs.* Li^+^/Li appears. This oxidation peak is also observed in CVs with [D_TEMPO_][TFSI] and [Im_TEMPO_][NO_3_] at *ca.* 3.76 V *vs.* Li^+^/Li. Importantly, under an Ar atmosphere, within the same potential window as the O_2_ experiment (comparison of black and teal traces in Fig. 7b–d), this small oxidation peak is not present, indicating that it is not a consequence of RM redox in this electrolyte. Additionally, under an O_2_ atmosphere, when the cathodic potential limit is restricted to 2.55 V *vs.* Li^+^/Li, such that the O_2_/O_2_˙^−^ redox couple is not active, this small oxidation peak is also not apparent. Therefore, this peak can be associated with the oxidation of a chemical decomposition reaction product between O_2_˙^−^ and the RM. Importantly, as this additional oxidation peak is apparent in both free (untethered) and tethered TEMPO RMs, this indicates that susceptibility towards O_2_˙^−^ is not due to tethering.

Previous studies have shown that when Li^+^ is present, O_2_˙^−^ (present as LiO_2_) disproportionates to Li_2_O_2_, and this disproportionation step is a major pathway towards ^1^O_2_ in Li–O_2_ cells.^72,73^ However, in these CVs, disproportionation is not possible as there is no Li^+^ present in the electrolyte. Therefore, it is unlikely that ^1^O_2_ is responsible for the small oxidation peak observed in CVs with RMs under O_2_, and direct electrochemical oxidation of O_2_˙^−^ to O_2_ is not known as a major pathway towards ^1^O_2_. Furthermore, the additional oxidation peak is not observed in the absence of an RM under O_2_ (Fig. 7a), indicating that it does not stem from O_2_˙^−^-induced degradation of the supporting electrolyte ([Pyrr_13_][TFSI]).

The CVs in Fig. 7 were conducted at 50 mV s^−1^, which is fast relative to the typical timescale of galvanostatic Li–O_2_ cell cycling. In the CVs with [D_TEMPO_][TFSI] at 50 mV s^−1^ (Fig. 7c), the small oxidation peak at *ca.* 3.76 V *vs.* Li^+^/Li appears as a shoulder peak tied to the RM oxidation peak. Upon decreasing the scan rate to 10 mV s^−1^ (Fig. S71), the oxidation peak at *ca.* 3.74 V *vs.* Li^+^/Li is distinctly separate from the RM oxidation peak and, qualitatively, the peak current ratio of this small oxidation peak to that of the RM oxidation peak has increased compared to the CV at 50 mV s^−1^. This suggests that decreasing the scan rate has allowed more time for the generation of the species that is being oxidised associated with the additional oxidation peak. It is hypothesised that O_2_˙^−^ has more time to chemically react with the RM at the slower scan rate, thus accumulating the decomposition product that is subsequently oxidised. Furthermore, holding at 1.9 V *vs*. Li^+^/Li during the reduction sweep results in growth of this additional oxidation peak on the subsequent oxidation sweep (Fig. S72), reinforcing the hypothesis of RM reaction with O_2_˙^−^.

Tethered RM degradation was further probed by *ex situ* NMR studies on cycled electrolytes within mediated Li–O_2_ cells operating with [D_TEMPO_][TFSI] and [Im_TEMPO_][NO_3_]. Fig. 7e–l shows the NMR spectra of [D_TEMPO_][TFSI]- and [Im_TEMPO_][NO_3_]-containing electrolytes within a chemical shift window of 0.5 to 2.0 ppm. The peaks in this window are primarily related to the alkyl chain of the RM and the tethered TEMPO moiety. However, based on comparison to the control ^1^H NMR spectra (Fig. S73), there is an intensity contribution to peak △ stemming from the NMR solvent, DMSO-d_6_.

Considering first the spectrum of the [D_TEMPO_][TFSI]-containing electrolyte after cycle 1, a small peak (labelled ◆ at 1.10 ppm in Fig. 7g) to the left of the two singlets (labelled ● and ▲) corresponding to the methyl groups on the TEMPO moiety is apparent. A peak at 1.10 ppm is also present in the pristine electrolyte (○), but it is considerably sharper and more consistent with the same peak observed in the control ^1^H NMR spectra containing diglyme (Fig. S73). In the cycled electrolytes, growth of peak ◆ was qualitatively confirmed by comparison of approximate integral peak intensity ratios. For example, from cycle 1 to 5, the ratios of ◆/▲ and ◆/● increase 3.9- and 4.2-fold, respectively. In cycle 5 (Fig. 7i), this peak has a shoulder, and by cycle 10 (Fig. 7k) the peak has split into two distinct peaks. These results point towards structural changes within the RM related to degradation of [D_TEMPO_][TFSI] itself. The peaks observed between 1.0 to 2.0 ppm in Fig. 7e, g, i and k are consistent with the ^1^H NMR spectra reported for neat [D_TEMPO_][TFSI] (Fig. S29). Peak ◆ at 1.10 ppm is also not observed in the unmediated electrolyte (0.8 mol kg^−1^ Li[TFSI] in diglyme) cycled with a Li_1−*x*_FePO_4_ counter electrode (Fig. S74), highlighting that the growth of peak ◆ is indeed due to RM degradation, and not the result of ROS-induced diglyme and/or Li[TFSI] decomposition.

A similar trend is observed for electrolytes cycled with [Im_TEMPO_][NO_3_] (Fig. 7f, h, j and l, see Fig. S37 for the ^1^H NMR spectrum of neat [Im_TEMPO_][NO_3_]). However, comparing the growth of peak ◆ from cycle 1 to cycle 5 in cells cycled with [D_TEMPO_][TFSI] and [Im_TEMPO_][NO_3_], peak growth is larger in the former; integral peak intensity ratios ◆/▲ and ◆/● increase 3.9- and 4.2-fold, respectively with [D_TEMPO_][TFSI] but only 2.8-fold with [Im_TEMPO_][NO_3_]. This is consistent with the enhanced performance of Li–O_2_ cells operating with [Im_TEMPO_][NO_3_] compared to [D_TEMPO_][TFSI] and reinforces that the growth of peak ◆ is associated with RM decomposition. Peak ◆ is at a similar chemical shift to peaks labelled ● and ▲, which correspond to the methyl groups in the TEMPO moiety in [D_TEMPO_][TFSI] and [Im_TEMPO_][NO_3_]. Therefore, decomposition may be associated with structural changes in the TEMPO moiety. It should be noted that the apparent large intensity of peak △ (stemming from DMSO-d_6_, see control spectra (Fig. S73)) in cycle 10 for [D_TEMPO_][TFSI] and [Im_TEMPO_][NO_3_] (Fig. 7k and l, respectively) is due to attenuation of the peaks corresponding to the tethered RMs as a result of RM decomposition.

Cell discharge capacities and rechargeability were improved significantly for cells operating with [D_TEMPO_][TFSI] when the Li metal negative electrode was replaced by Li_1−*x*_FePO_4_. Therefore, ^1^H NMR analysis of the electrolyte was conducted after 5 cycles of a [D_TEMPO_][TFSI]-mediated cell with Li_1−*x*_FePO_4_ as the counter electrode (Fig. S75). Peak ◆ is clearly larger in the electrolyte cycled with Li metal, however, the fact that this peak is also present in the cell cycled with Li_1−*x*_FePO_4_ indicates ROS-induced RM decomposition. [D_TEMPO_][TFSI] decomposition when the Li–O_2_ cell is not galvanostatically cycled was also considered to probe the reactivity of the RM with Li metal and Li_1−*x*_FePO_4_ when no reactive oxygen species (such as O_2_˙^−^, O_2_^2−^ and ^1^O_2_) from the ORR and OER are present. Under these conditions, peak ◆ is either not observed or is of extremely low intensity, highlighting that its growth is reliant upon O_2_ electrochemistry taking place.

Based on the CV study, the nature of the RM degradation reaction is thought to involve the TEMPO moiety itself, as the additional oxidation peak in the CVs is present with free (untethered) TEMPO. This is supported by the NMR study, wherein peak ◆ at 1.10 ppm (ascribed to RM decomposition) which grows on cycling in [D_TEMPO_][TFSI]- and [Im_TEMPO_][NO_3_]-containing electrolytes is at a similar chemical shift to the methyl protons on the TEMPO moiety.

### Systematic replacement of lithium–oxygen cell components

**Fig. 8:**
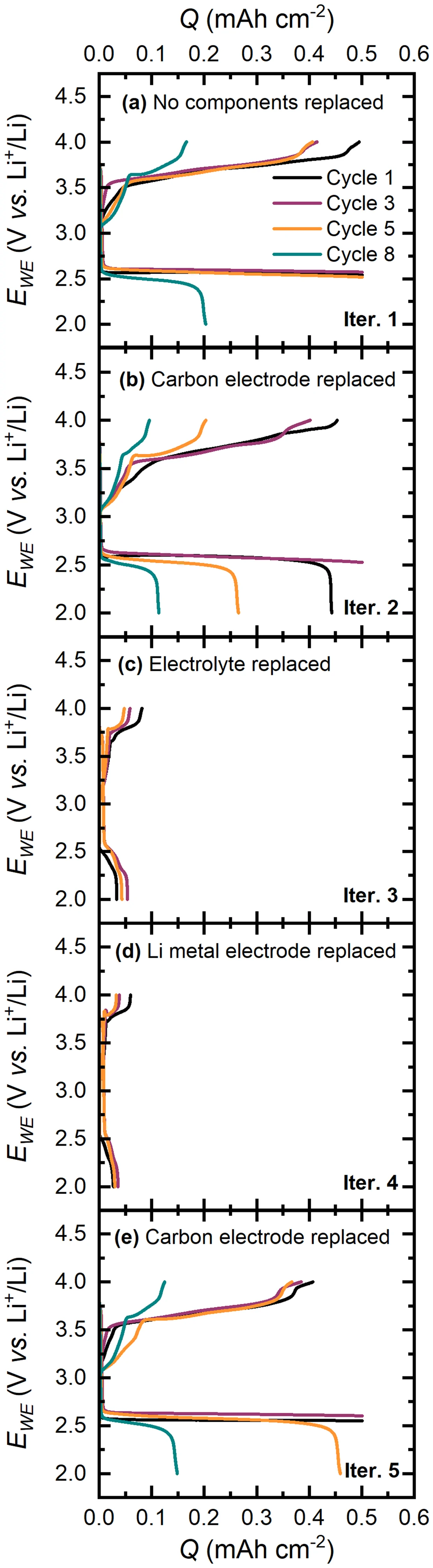
Galvanostatic cycles of a standard Li–O_2_ cell comprising a carbon paper positive electrode, Li metal negative electrode and an electrolyte consisting of 25 mmol kg_solvent_^−1^ [D_TEMPO_][TFSI] in 0.8 mol kg_solvent_^−1^ Li[TFSI] in diglyme. The same, single cell was used throughout, and the carbon paper positive electrode, Li metal negative electrode and electrolyte were systematically replaced after each iteration (Iter.) of cell failure: (a) no components replaced, (b) carbon electrode replaced, (c) electrolyte replaced, (d) Li metal replaced and (e) carbon electrode replaced again. For each iteration, the capacity was limited to 0.5 mAh cm^−2^ and the cell was cycled at a current density of 0.1 mA cm^−2^ with discharge and charge potential cut-offs of 2.0 and 4.0 V *vs*. Li^+^/Li, respectively.

Failure of Li–O_2_ cells is rarely due to decomposition associated with a single component within the cell in isolation. To understand the practical effects of RM degradation on the cell components bottlenecking performance, systematic replacement of the carbon positive electrode, electrolyte, and Li metal negative electrode was carried out in Li–O_2_ cells cycled with [D_TEMPO_][TFSI]. Fig. 8 shows the corresponding galvanostatic cycling profiles.

Following the first instance of cell failure (Fig. 8a, iteration 1), defined as the cell not being able to deliver the discharge capacity limit (0.5 mAh cm^−2^), the carbon electrode was replaced, the cell re-purged with fresh high purity O_2_, rested for 8 h and galvanostatically cycled again. The same procedure was followed every time a component was replaced. Replacement of the carbon electrode recovered cell performance (Fig. 8b, iteration 2). Interestingly, in iteration 2, the cell fails to deliver the discharge capacity limit in cycle 1 but can do so in cycle 3. This may be due to the electrolyte not being replaced between iterations 1 and 2, and therefore, the cell requires more time to wet the replaced carbon electrode. Following failure of iteration 2, the electrolyte was replaced (wherein the separator was removed and replaced, and 80 µL of fresh electrolyte was added), which resulted in no recovery of cell performance (Fig. 8c, iteration 3). The plateau corresponding to RM oxidation in iterations 1 and 2 was between *ca.* 3.5–3.7 V *vs*. Li^+^/Li, which is below the redox potential of 3.77 V *vs.* Li^+^/Li for [D_TEMPO_][TFSI] under an Ar atmosphere (Fig. S56d). This is consistent with the coupled chemical process described previously. In contrast, in iteration 3, where the electrolyte has been replaced but the carbon electrode was unchanged (*i.e.*, previously cycled to failure in iteration 2), the RM oxidation potential is consistent with the redox potential of [D_TEMPO_][TFSI] under an Ar atmosphere. This observation, combined with the significantly reduced recharge capacity in iteration 3, suggests that the coupled chemical process described earlier does not occur or is substantially mitigated, possibly by coverage of the carbon electrode by parasitic products.

It is important to consider that iterations 2 and 3 were cycled with the same Li electrode used in iteration 1. Therefore, failure in iterations 2 and 3 may also be associated with the interface of the Li electrode. Following electrolyte replacement (iteration 3), the Li electrode was replaced (Fig. 8d, iteration 4) and, similar to the case following electrolyte replacement, no recovery of cell performance was observed. To confirm that the carbon electrode is the bottleneck for cell performance, it was replaced again (Fig. 8e, iteration 5). Following all iterations of changing various cell components, re-replacing the carbon positive electrode resulted in the same recovery response as observed in iteration 2. Therefore, despite the relative instability of the Li|electrolyte interface when [D_TEMPO_][TFSI] is present, the component dominating cell failure in this instance is the carbon electrode. The lack of a distinct RM oxidation plateau when the electrolyte is replaced (iteration 3), suggests that passivation of the carbon electrode leads to cell failure. This component replacement study highlights that an understanding of the primary failure mechanisms is needed before drawing conclusions on the effect of new components in Li–O_2_ cells.

## Conclusions

The merits of RM tethering were explored in this work for a series of TEMPO-tethered mediators. Beneficial properties from RM tethering have been demonstrated in terms of enhancing discharge and charge processes relative to their untethered equivalent when Li_1−*x*_FePO_4_ was used as the negative electrode. Under this condition, cells with [Im_TEMPO_][TFSI] and [Pyrr_TEMPO_][TFSI] outperformed untethered (free) TEMPO-mediated cells, while [D_TEMPO_][TFSI]-containing cells performed comparably. However, replacing Li_1−*x*_FePO_4_ with Li metal resulted in poorer cell cyclability with the tethered RMs compared to TEMPO, indicating that Li metal reactivity was a significant source of tethered RM degradation. The introduction of [NO_3_]^−^ as the counterion instead of [TFSI]^−^ to yield the imidazolium-based nitrate-bearing RM, [Im_TEMPO_][NO_3_], resulted in a marked improvement in cell discharge capacities and rechargeability compared to TEMPO in Li metal-based cells. However, [Im_TEMPO_][NO_3_] efficacy was superior compared to all other RMs tested even when Li_1−*x*_FePO_4_ negative electrodes were used, indicating that the performance enhancement with [Im_TEMPO_][NO_3_] was not solely due to nitrate-derived stabilisation of the Li|electrolyte interface, as is widely known for nitrate salts. It was shown that additive-level loadings of [Im_TEMPO_][NO_3_] resulted in an exceptionally high Li_2_O_2_ yield of 99.8 ± 0.1% and drove platelet-like Li_2_O_2_ formation. The enhancement in cell performance may also be related to Li_2_O_2_ morphology, as this platelet-like morphology was not observed with the [TFSI]^−^-based analogue ([Im_TEMPO_][TFSI]), which exhibited poorer cell performance characteristics. Therefore, the relative reactivity of Li_2_O_2_ morphologies and RMs that can modulate this reactivity should be explored further.

The improved performance with [Im_TEMPO_][NO_3_] was further rationalized by *operando* pressure measurements, from which a general criterion for RM efficacy was verified. RM efficacy is maximised when the majority of gas evolution occurs at the RM oxidation potential. Therefore, cell lifetime is maximised when this type of pressure response can be sustained for as many charge half-cycles as possible. The approach and analysis methodology can be readily and accessibly used to screen various charge RMs for their efficacy in Li–O_2_ cells under conditions identical to the standard Li–O_2_ cell used in this work. Furthermore, as all capacity-limited galvanostatic cycling was performed with a 4.0 V *vs.* Li^+^/Li charge potential cut-off, this work highlights the achievable cycle life under conditions where cells with unmodified carbon paper positive electrodes are forced to be reliant on RM-driven charging rather than a combination of mediated charging and electrochemical oxidation of the carbon surface, as is the case at higher potential cut-offs (*e.g.*, 4.5 V *vs*. Li^+^/Li).

Cyclic voltammetry under conditions where O_2_/O_2_˙^−^ redox is chemically reversible revealed that the TEMPO moiety in [D_TEMPO_][TFSI] and [Im_TEMPO_][NO_3_] is susceptible to O_2_˙^−^-induced decomposition. Importantly, as no Li^+^ was present, disproportionation of LiO_2_ to yield Li_2_O_2_ and O_2_ (where a significant fraction has been shown to be ^1^O_2_) is not possible, thus ruling out ^1^O_2_ and O_2_^2−^ as drivers of this decomposition. ^1^H NMR of cycled electrolytes containing [Im_TEMPO_][NO_3_] and [D_TEMPO_][TFSI] both show the growth of a peak on cycling of comparable chemical shift to the methyl protons in the TEMPO moiety, reinforcing that superoxide-induced decomposition occurs on the TEMPO group of the tethered RMs. Crucially, this peak was also present in cells cycled with Li_1−*x*_FePO_4_ and absent in the corresponding unmediated electrolyte, indicating that it is related to ROS-induced degradation of the RM. Therefore, there should be a focus on the design of tethered RMs with redox moieties that are resistant to superoxide-induced decomposition. As it has been demonstrated that the tethering strategy can enhance the desired electrochemical reactions at the positive electrode relative to the untethered counterpart, pairing tethered mediators with an appropriate Li metal protection strategy alongside practical consideration of the key bottleneck components will enable enhanced mediated Li–O_2_ cell cyclability.

## Experimental

### Electrolyte preparation

All electrolytes used in this work were prepared in an Ar-filled glovebox (O_2_ and H_2_O < 0.1 ppm). Electrolytes for CVs comprised of either 1 M Li[TFSI] or 1 M 1-propyl-1-methylpyrrolidinium [TFSI] ([Pyrr_13_][TFSI]) in diglyme with or without RMs. The RM concentration was 10 mM. For Li–O_2_ cells, electrolytes were prepared by mass and used within two months from preparation. These electrolytes comprised of 0.8 mol kg_solvent_^−1^ Li[TFSI] in diglyme with or without RM. The RM loading was 25 mmol kg_solvent_^−1^ and select mediated electrolytes were prepared at a higher loading of 135 mmol kg_solvent_^−1^. Table S3 summarizes all electrolytes prepared for Li–O_2_ cells and relevant component molalities. Diglyme was dried successively over freshly activated molecular sieves (3 Å) and Li[TFSI] was dried under vacuum at 10^−2^ mbar at 100 °C for 24 h, followed by further drying at 10^−5^ mbar for 48–72 h at the same temperature. The synthesised RMs were dried under vacuum following a similar procedure (24 h at 10^−2^ mbar at 100 °C and then 24 h at 10^−5^ mbar at the same temperature). The water content in all electrolytes used in this work was found to be <15 ppm by coulometric Karl Fischer titration (C20 Compact KF Coulometer, Mettler Toledo, Switzerland).

### Li–O_2_ cell assembly and cycling protocol

All stainless-steel Swagelok-type Li–O_2_ cells were assembled and sealed in an Ar filled glovebox (O_2_ and H_2_O < 0.1 ppm), wherein the component stack was housed in a polyether ether ketone (PEEK)-lined cell body (see Fig. S54 for an image of the “standard” cell). The component stack comprised a stainless-steel mesh (∅ 12 mm, aperture size 0.085 mm), a carbon paper (∅ 10 mm) positive electrode, two glass fibre separators (∅ 12.7 mm, GF/F) soaked with electrolyte (80 µL) and either Li metal (∅ 12 mm) or freestanding Li_1−*x*_FePO_4_ (∅ 12 mm) as the negative electrode. The latter provides a stable reference potential (3.45 V *vs*. Li^+^/Li) when partially delithiated and is a useful alternative negative electrode when RMs exhibit reactivity with Li metal.^48,74^ Preparation/fabrication of all cell components is described in detail in the SI. Li–O_2_ cells in which pressure within the internal headspace was measured during cycling contained the same electrode stack (see Fig. S66 for images of the *operando* pressure cell).

All Li–O_2_ cells were cycled under a galvanostatic, capacity-limited (0.5 mAh cm^−2^) cycling regime with a discharge potential cut-off of 2.0 V *vs.* Li^+^/Li and a charge potential cut-off of either 4.0 or 4.5 V *vs.* Li^+^/Li at a current density of 0.1 mA cm^−2^. For full discharge tests, Li–O_2_ cells were discharged to 2.4 V *vs.* Li^+^/Li at the same current density. Capacities and current densities are expressed per unit geometric area of the carbon paper positive electrode. Where Li_1−*x*_FePO_4_ was used, these electrodes exhibited *ca.* 7-fold greater Li excess relative to the capacity limit (0.5 mAh cm^−2^) applied to discharging and charging of the carbon electrode. The assembled cells were purged for 30 min with high purity O_2_ (N5.5, BOC, UK) and rested for 8 h prior to cycling. *Operando* pressure cells were rested for *ca.* 24 h to ascertain a leak rate in the system, which was then used to correct the measured pressure response. An example of the leak rate correction is described in Fig. S67. A detailed description of the volume calibration procedure for the *operando* pressure cell and data processing methods have been described in a previous study.^70^ All Li–O_2_ cells were cycled on Bio-Logic (France) potentiostats: standard Li–O_2_ cells containing Li metal as the negative electrode were cycled on a BCS-805 cycler, while those containing Li_1−*x*_FePO_4_ as the negative electrode were cycled on an MPG-2 potentiostat. *Operando* pressure cells were cycled on a SP-300 potentiostat.

### Cyclic voltammetry

Cyclic voltammetry (CV) was conducted in a three-electrode glass cell on a SP-300 potentiostat (Bio-Logic, France) under Ar and O_2_ atmospheres. The working electrode (WE) was a planar glassy carbon macrodisk electrode (geometric surface area: 7.07 mm^2^) and the counter electrode (CE) was a coiled platinum wire. The reference electrode (RE) was a silver wire immersed in a reference electrolyte comprising 100 mM Ag[OTf] (≥99%) in [Pyrr_13_][TFSI] (99.9%). The reference electrolyte was separated from the analyte electrolyte by a porous glass frit. Preparation of the WE, CE and RE, and conversion of potentials from the Ag[OTf]/Ag scale to the Li^+^/Li scale (Fig. S55) are described in detail in the SI.

### Li|Li symmetric cells

Li|Li symmetric cells were assembled in an Ar-filled glovebox in CR2025-type coin cells. The cell stack comprised Li metal disc electrodes (∅ 12 mm) separated by a glass fibre separator wetted with electrolyte (same formulations as were used for Li–O_2_ cells). The symmetric cells were galvanostatically cycled on a S4000 battery cycler (Maccor, US) at 0.1 mA cm^−2^ with potential cut-offs of −1.8 and 1.8 V *vs.* Li^+^/Li on discharge and charge, respectively. Cells were charged and discharged for 1 h each equating to a stripping/plating capacity of 0.1 mAh cm^−2^ per half-cycle.

### ^1^H NMR analysis of cycled electrolytes

^1^H NMR was used to analyse electrolytes post-cycling with a focus on peaks corresponding to [D_TEMPO_][TFSI] and [Im_TEMPO_][NO_3_] in diglyme-based electrolytes. Li–O_2_ cells were galvanostatically cycled with a 0.5 mAh cm^−2^ capacity limit at 0.1 mA cm^−2^ with Li metal or Li_1−*x*_FePO_4_ as the negative electrode. Cells were purged with Ar, disassembled and separators extracted after 1, 5 and 10 cycles. Some electrolyte analysis was also performed after cycling in the base electrolyte (no RM) with Li_1−*x*_FePO_4_ as the negative electrode to verify that evolution of ^1^H NMR spectra of cycled RM-containing electrolytes was indeed due to RM degradation. Additionally, ^1^H NMR electrolyte analysis was also conducted after resting cells (without galvanostatic cycling) under an O_2_ atmosphere to understand RM reactivity in the absence of Li–O_2_ cell electrochemical reactions with either Li_1−*x*_FePO_4_ or Li metal as the negative electrode. In all cases, separators from cycled cells were extracted inside an Ar-filled glovebox and dried in the glovebox antechamber for 1 h to remove residual diglyme. The dried separators were taken back into the Ar-filled glovebox washed with a minimal volume (0.7 mL) of high purity deuterated DMSO-d_6_ (99.96%, dried over molecular sieves (H_2_O < 15 ppm, determined by Karl Fischer titration)) to dissolve the RM and Li[TFSI] salt and *ca.* 5 µL of phenylhydrazine (97%, also dried over molecular sieves, H_2_O < 15 ppm) was added to reduce the nitroxyl radical (as was done prior to acquisition of ^1^H NMR spectra of the synthesised RMs). The DMSO-d_6_ wash was then sealed inside an NMR tube and ^1^H NMR spectra were acquired using an Avance III spectrometer (Bruker, USA).

### Scanning electron microscopy of cycled carbon electrodes

Li–O_2_ cells containing carbon paper and Li metal as the positive and negative electrodes, respectively, were discharged to 0.5 mAh at 0.1 mA cm^−2^ in select mediated diglyme-based electrolytes. After cycling, cells were purged with Ar and then taken into an Ar-filled glovebox. The carbon electrode was extracted, washed with diglyme and dried in the glovebox antechamber under vacuum for 1 h at room temperature. For each electrolyte formulation, a wedge from the corresponding discharged circular carbon electrode was cut and pasted onto a conductive carbon tab stuck to a SEM sledge. The sledge was transferred into the SEM chamber without exposure to air *via* a hermetically sealed sample holder. Micrographs were acquired on a S8000G focused ion beam (FIB)-SEM (Tescan, Czech Republic) at a beam accelerating voltage of 5 keV, a beam current of 35 pA and a working distance of 4.5 mm. To ensure micrographs were representative of the sample, images were taken at 2–3 spots on each carbon electrode wedge.

### Determination of Li_2_O_2_ yield

Li_2_O_2_ yield was determined using a widely reported titration method using titanium oxysulfate (TiOSO_4_).^49,74^ Li–O_2_ cells were first discharged to 0.5 mAh cm^−2^ using P50 carbon paper as the positive electrode and Li metal as the negative electrode. After discharge, the cell was purged with Ar and the carbon electrode extracted inside an Ar-filled glovebox. Following a drying step inside the glovebox antechamber, the electrode was placed into a septum sealed vial, taken out of the glovebox and treated with a solution of 2 wt% TiOSO_4_ in H_2_O/H_2_SO_4_ : H_2_O (1 : 1 by volume). Water reacts with Li_2_O_2_ forming LiOH and H_2_O_2_. In the presence of H_2_O_2_, the titanium complex reacts to form a [TiO_2_]^2+^ species, which results in a colour change from colourless to yellow. After a reaction time of 30 min, an aliquot of this solution was diluted with water to avoid saturation of the UV-Vis detector and analysed by UV-Vis spectroscopy. The [TiO_2_]^+^ species exhibits a maximum absorbance at 407 nm, and it was this peak absorbance that was used to quantify the Li_2_O_2_ yield (taking into account the dilution factor) using a calibration curve prepared with concentration standards of commercial Li_2_O_2_ (95%).

### Raman spectroscopy analysis of carbon electrodes

Raman spectroscopy was used to detect Li_2_O_2_ formed on discharged P50 carbon paper electrodes. The characteristic Raman bands associated with Li_2_O_2_ are at *ca.* 250 cm^−1^ (Li–O_2_ coupled vibrational mode) and *ca.* 790 cm^−1^ (O–O stretching mode).^75^ Both are weak bands, although the latter is more intense than the former. Li–O_2_ cells were first discharged to 0.5 mAh at a current density of 0.1 mA cm^−2^ with P50 carbon paper positive electrodes in diglyme-based electrolytes with [Im_TEMPO_][NO_3_] as the RM and Li metal as the negative electrode. The cell was then purged with Ar gas and taken into an Ar-filled glovebox before cell disassembly and electrode extraction. The extracted electrode was dried for 2 h in the glovebox antechamber under a dynamic vacuum to remove excess diglyme and placed into a hermitically sealed *ex situ* cell with a borosilicate optical window. An optical microscope was used to focus the 532 nm Raman laser spot onto the carbon surface and at least 5 spots (20 accumulations per spot) on the carbon electrode were analysed per electrode. Raman spectra were acquired on an *In via* spectrometer with an inverted optical microscope (Renishaw, UK).

## Author contributions

TS: conceptualisation, formal analysis, investigation, supervision, methodology, writing original draft manuscript, review and editing, and visualisation. ARN: conceptualisation, investigation, supervision, methodology, writing manuscript, review, and editing, and visualisation. EC: review and editing of manuscript. DJS: review and editing of manuscript. TP: review and editing of manuscript, LJH: conceptualisation, funding acquisition, project administration, supervision, writing manuscript, review, and editing. All authors have given approval to the final version of the manuscript.

## Conflicts of interest

A patent application has been filed by Lubrizol Ltd. for parts of the work in this article, wherein all authors are co-inventors.

## Supplementary Material

EB-002-D6EB00134C-s001

## Data Availability

The data supporting this article have been included as part of the supplementary information (SI). Supplementary information is available. See DOI: https://doi.org/10.1039/d6eb00134c. The supplementary information includes the following: (i) details on materials used, (ii) additional experimental information, (iii) synthetic protocol and characterization data (including 1D and 2D ^1^H and ^13^C NMR, ATR-FTIR and ESI-MS spectra, TGA and DSC thermograms, and elemental microanalysis), (iv) additional electrochemical data (cyclic voltammograms and galvanostatic cycling profiles), and (v) *ex situ* characterization data (calibration curves, Raman spectra and SEM micrographs of cycled electrodes and ^1^H NMR spectra of cycled electrolytes).
