# Supplementary material for: To tether or not to tether? The merits of adding functionality to Li–O2 cell redox mediators
Source: EES Batter. 2026 Aug 24;2(5):1703–20. doi: 10.1039/d6eb00134c (PMC13551449; doi:10.1039/d6eb00134c)
Supplement: Supplementary file 1 [file EB-002-D6EB00134C-s001.pdf]

Supplementary Information (SI) for EES Batteries.
This journal is © The Royal Society of Chemistry 2026

Supplementary Information

To tether or not to tether? The merits of adding functionality to Li-O_2 cell redox mediators

Thukshan Samarakoon,[1] Alex R. Neale,[1] Elliot Coulbeck,[2] Dan Saccomando,[3] Tyler Petek[4] and Laurence J. Hardwick[1,*]

[1]*Stephenson Institute for Renewable Energy, Department of Chemistry, University of Liverpool, Liverpool, L69 7ZF, UK*
[2]*Lubrizol Limited, Blackley, Manchester, M9 8ES, UK*
[3]*Lubrizol Limited, Hazelwood, Derby, DE56 4AN, UK*
[4]*Lubrizol Limited, 29400 Lakeland Boulevard, Wickliffe, Ohio 44092-2201, USA*

*Corresponding author: hardwick@liverpool.ac.uk

Table of Contents

Materials **3**
Synthesis and characterisation of tethered redox mediators **4**
Reaction schemes 4
Synthesis protocols 8
Scaled-up synthesis of imidazolium- and pyrrolidinium-based TEMPO-tethered redox mediators 10
1D and 2D NMR characterisation 13
ATR-FTIR spectroscopy 58
CHN elemental microanalysis 60
Electrospray ionisation mass spectrometry 61
Thermal stability 65
Standard lithium-oxygen cell and preparation of cell components **68**
Cyclic voltammetry **71**
Electrochemical and spectroscopic characterisation of tethered RMs **73**
Supporting Discussion 1 75
^{1}H NMR analysis of cycled electrolytes **98**
References **101**

Materials

Potassium hydroxide (KOH, 99.98%, trace metal basis), tetra-*n*-butylammonium bromide ($[NBu_4][Br]$, 99+%), dichloromethane (DCM, 99%), hexane (≥95%), ethyl acetate (EtOAc, 99%), isopropanol (99%, IPA), hydrogen peroxide (30%), glacial acetic acid (99.7%), ethanol (GPR), lithium peroxide (95%) and diethyl ether (99%) were purchased from Fisher Scientific UK. 4-hydroxy-2,2,6,6-tetramethylpiperidin-1-oxyl (TEMPO-OH, 97%), toluene (99.8%), 1,5-dibromopentane (97%), acetonitrile (99.8%), phenylhydrazine (97%), 1,2-dimethylimidazole (97%), 1,4-diazabicyclo[2.2.2]octane (≥99%), diglyme (>99.5%, anhydrous), lithium metal (99.9%, ribbon, 0.38 mm thickness, 23 mm width), polytetrafluoroethylene (PTFE, 60 wt% dispersion in water), 1.9–2.1% titanium(IV) oxysulfate solution (prepared according to DIN 38 409, part 15, DEV-18), silver trifluoromethanesulfonate (Ag[OTf], ≥99%), ferrocene (98%), iron (II) sulfate hydrate (99.999%, trace metals basis), sulfuric acid (99.999%), lithium ribbon (99.9%, 0.38 mm thickness) and silver nitrate ($Ag[NO_3]$, ≥99.0%) were purchased from Merck Life Science (UK). Silver wire, the glass capillary and glass frits to assemble an Ag[OTf]/Ag reference electrode were purchased from IJ Cambria Scientific Ltd. (UK). 1-methylpyrrolidine (98%) was purchased from Alfa Aesar (UK). Silica gel (40-63 µm) was purchased from VWR Chemicals. Lithium bis(trifluoromethane)sulfonylimide (Li[TFSI], 99%+) and 1-propyl-1-methylpyrrolidinium [TFSI] ($[Pyrr_{13}][TFSI]$, 99.9%) were purchased from Solvionic (France). 2,2,6,6-tetramethylpiperidin-1-oxyl (TEMPO, 99%) and dimethylsulfoxide-d_6 (DMSO-d_6, 99.96%) were purchased from Fluorochem (UK). Alumina powder of varying particle sizes (1.0, 0.3 and 0.05 µm), purchased from Buehler (US), was used to prepare aqueous slurries of alumina for polishing of the glassy carbon macrodisk electrode used for cyclic voltammetry experiments. All components used to build the standard Li-O_2 cells were purchased from Swagelok (UK) except for the PEEK-lined cell bodies and the stainless-steel tubing (⌀ 12.7 mm) used as the O_2 reservoir, which were supplied by Microplas Mouldings (UK). P50 carbon paper (Avcarb) served as the positive electrode material and was purchased from Fuel Cell Store (US). Glass fibre separators (GF/F, Whatman) were purchased from Scientific Laboratory Supplies (UK). Lithium iron phosphate ($LiFePO_4$, particle size: 1.5 µm) powder was purchased from MSE Materials (US). Stainless-steel mesh (opening: 0.085 mm, 180 mesh per inch, wire diameter: 0.05 mm) was purchased from Advent Research Materials Ltd. (UK). Super C65 carbon black was purchased from TIMCAL (Belgium). High purity O_2 gas (N5.5) for purging Li-O_2 cells was purchased from the British Oxygen Company (UK).

Synthesis and characterisation of tethered redox mediators

<u>Reaction schemes</u>

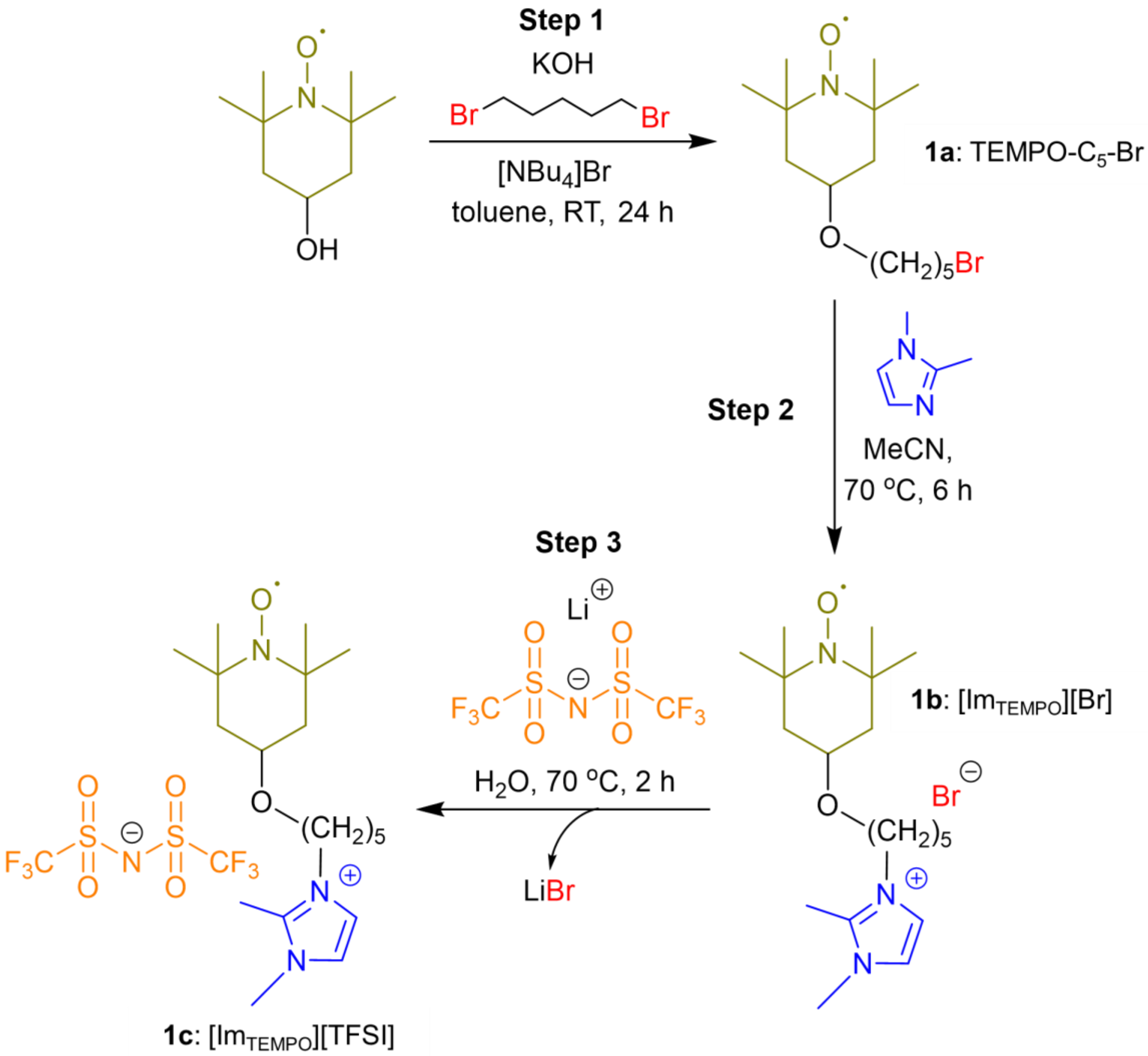

Scheme S1 | Low-scale synthetic route towards [Im$_{TEMPO}$][TFSI] (modifications made to reaction times and temperatures for the scaled-up synthesis are detailed in **Table S1**). Step 1 involves synthesis of the TEMPO-alkyl halide precursor (TEMPO-C$_5$-Br, **1a**), which is subsequently treated with 1,2-dimethylimidazole in the quaternization reaction (Step 2) to give the bromide salt ([Im$_{TEMPO}$][Br], **1b**). [Im$_{TEMPO}$][Br] is then used in the anion metathesis reaction (Step 3) to yield the target RM ([Im$_{TEMPO}$][TFSI], **1c**).

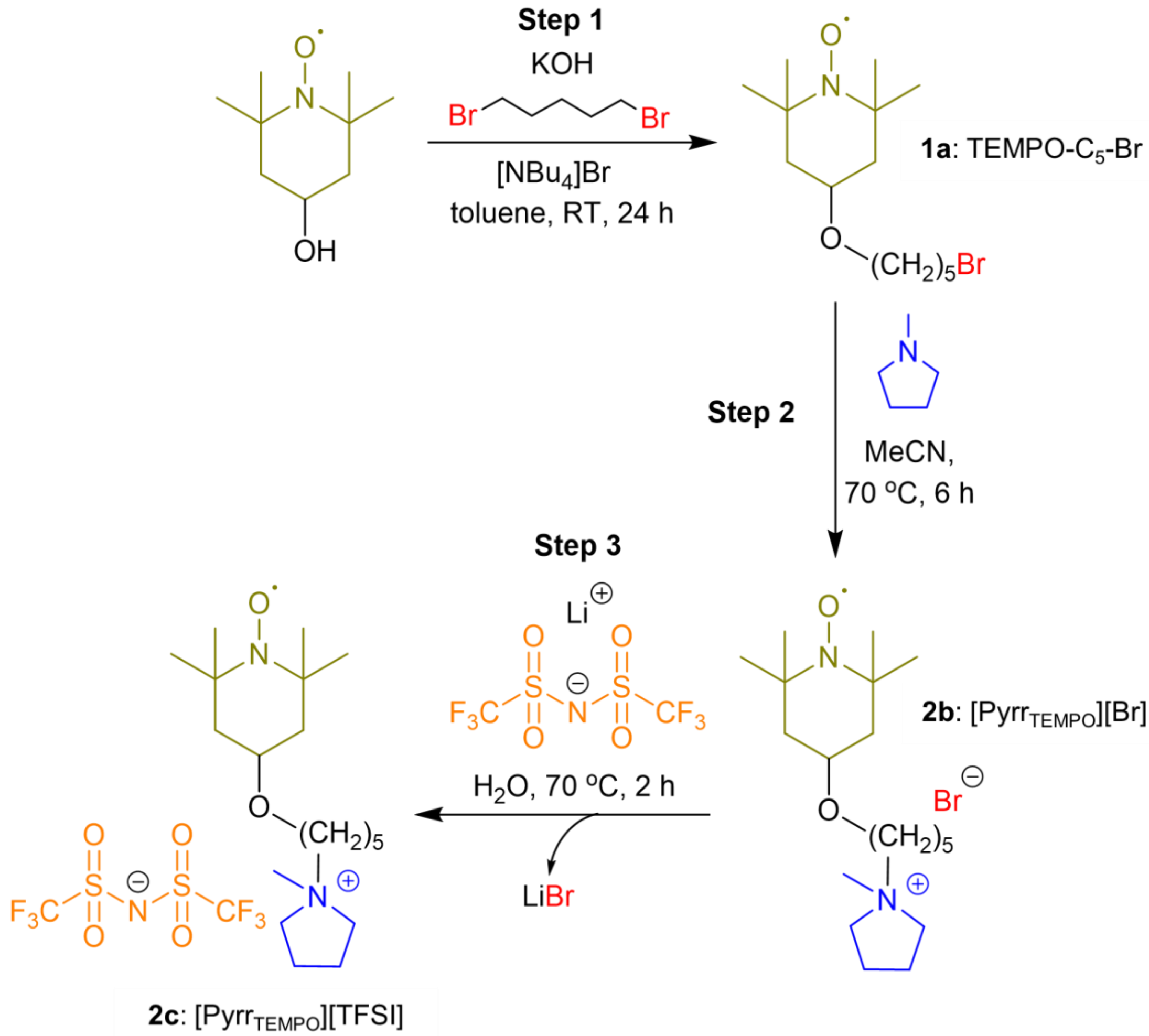

Scheme S2 | Low scale synthetic route towards [Pyrr$_{TEMPO}$][TFSI] (modifications made to reaction times and temperatures for the scaled-up synthesis are detailed in **Table S1**). Step 1 involves synthesis of the TEMPO-alkyl halide precursor (TEMPO-C$_5$-Br, **1a**), which is subsequently treated with 1-methylpyrrolidine in the quaternization reaction (Step 2) to give [Pyrr$_{TEMPO}$][Br] (**2b**). [Pyrr$_{TEMPO}$][Br] is then used in the anion metathesis reaction (Step 3) to yield the target RM ([Pyrr$_{TEMPO}$][TFSI], **2c**).

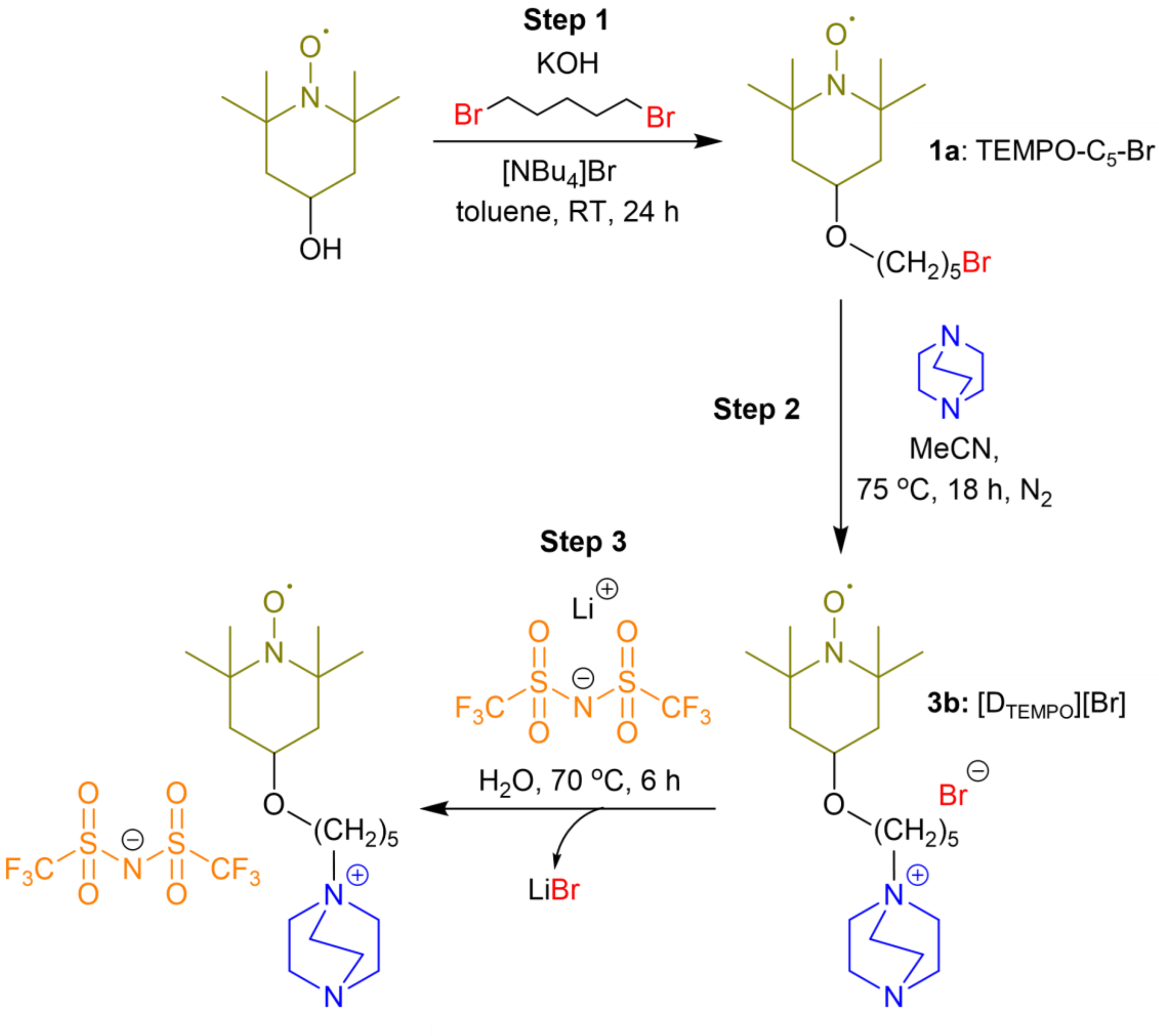

Scheme S3 | Synthetic route towards D_{TEMPO}. Step 1 involves synthesis of the TEMPO-alkyl halide precursor (TEMPO-C_5-Br, **1a**), which is subsequently treated with 1,4-diazabicyclo[2.2.2]octane (DABCO) in the quaternization reaction (Step 2) to give the bromide salt ([D_{TEMPO}][Br], **3b**). [D_{TEMPO}][Br] is then used in the anion metathesis reaction (Step 3) to yield the target RM ([D_{TEMPO}][TFSI], **3c**).

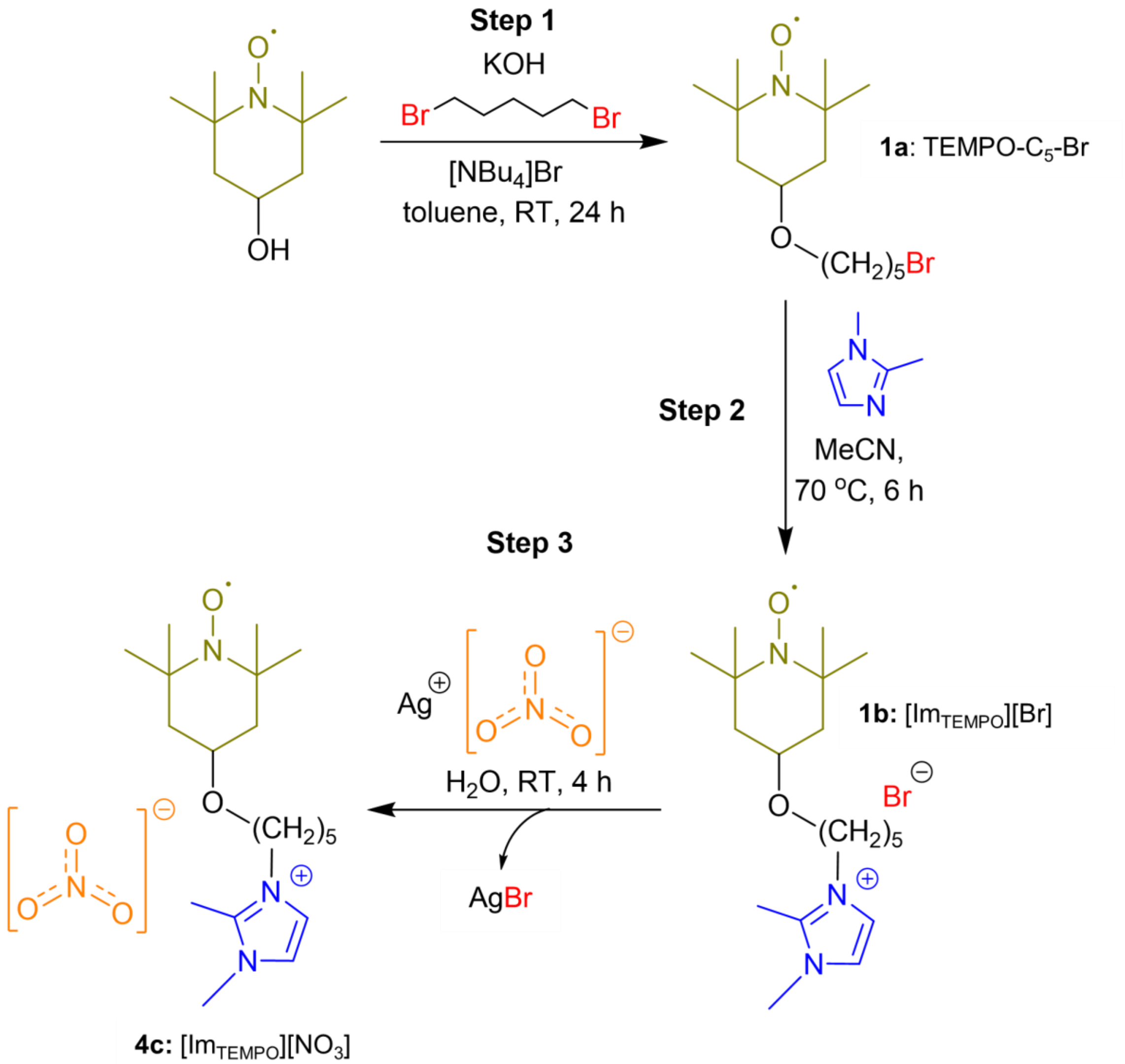

Scheme S4 | Synthetic route towards [Im_{TEMPO}][NO_3]. Step 1 involves synthesis of the TEMPO-alkyl halide precursor (TEMPO-C_5-Br, **1a**), which is subsequently treated with 1,2-dimethylimidazole in the quaternization reaction (Step 2) to give the bromide salt ([Im_{TEMPO}][Br], 1**b**). [Im_{TEMPO}][Br] is then used in the anion metathesis reaction with silver nitrate (Ag[NO_3]) (Step 3) to yield the target RM ([Im_{TEMPO}][NO_3], 4**c**).

Synthesis protocols

Synthesis of 4-((5-bromopentyl)oxy)-2,2,6,6-tetramethylpiperidin-1-oxyl (TEMPO-C_5-Br, **1a**)

To a flask containing TEMPO-OH (1.50 g, 0.0087 mol, 1 eq.) in toluene (15 mL), KOH (1.95 g, 0.0348 mol, 4 eq.) and [NBu_4][Br] (0.28 g, 0.00087 mol, 0.1 eq.) were added. The mixture was stirred for 15 minutes to allow complete dissolution of all solutes, resulting in a red solution, to which 1,5-dibromopentane (2.45 mL, 0.018 mol, 2.1 eq.) was added. The solution was then stirred vigorously at room temperature for 24 h. KOH was used as a base to deprotonate the hydroxyl group in TEMPO-OH and [NBu_4][Br] was used as a phase transfer catalyst. Following reaction, deionised water (2 mL) was added to dissolve unreacted KOH, followed by solvent removal by rotary evaporation. The remaining red liquid was further treated with deionised water (10 mL) and subsequently extracted with dichloromethane (5 x 10 mL). The combined organic extracts were concentrated by rotary evaporation to yield a red viscous liquid as the crude product. Column chromatography was employed to purify the crude product using a semi-automated Isolera One (Biotage, Sweden) purification instrument. The column consisted of a silica stationary phase and a hexane : ethyl acetate mobile phase. A gradient elution was performed starting from 100:0 and ending with 40:60 by volume. The crude product was first adsorbed onto silica gel by dissolving it in dichloromethane and then removing the solvent by rotary evaporation in the presence of the silica gel to give a crude product-adsorbed silica powder. This was then dry-loaded onto the column. Thin layer chromatography (TLC) plates were used to determine the optimal final ratio of hexane:ethyl acetate comprising the mobile phase prior to running the column. When running the column, an initial pure hexane wash was performed to remove any residual toluene and unreacted alkyl reagents, and ethyl acetate was used to move the target product, which does not move with the pure hexane mobile phase. The purified product was dried under vacuum at room temperature at 10^{-2} mbar overnight to give TEMPO-C_5-Br (**1a**) as a viscous (oil-like) red liquid (1.72 g, 62%).

Synthesis of 3-(5-((1-oxyl-2,2,6,6-tetramethylpiperidin-4-yl)oxy)pentyl)-1,2-dimethylimidazol-3-ium bromide ([Im_{TEMPO}][Br], **1b**)

A solution of 1,2-dimethylimidazole (218 µL, 2.46 mmol, 1.2 eq.) in acetonitrile (3 mL) was added to a solution of TEMPO-C_5-Br (0.66 g, 2.05 mmol, 1 eq.) in acetonitrile (3 mL). The resulting red solution was stirred for 6 h at 70 °C. The reaction solvent was removed by rotary evaporation and the remaining red viscous liquid was purified by trituration in diethyl ether (5 x 5 mL). Following each addition of diethyl ether, the mixture was subjected to sonication at room temperature for 2 min. The addition of diethyl ether induced precipitation of an orange solid, which was isolated by vacuum filtration. The solid was dried at 10^{-2} mbar overnight at room temperature to give [Im_{TEMPO}][Br] (**1b**) as a coarse orange powder (0.60 g, 70%).

Synthesis of 3-(5-((1-oxyl-2,2,6,6-tetramethylpiperidin-4-yl)oxy)pentyl)-1,2-dimethylimidazol-3-ium bis(trifluoromethane)sulfonimide ([Im_{TEMPO}][TFSI], **1c**)

Solutions of Li[TFSI] (0.32 g, 1.11 mmol, 1 eq.) in water (1.6 mL) and [Im_{TEMPO}][Br] (0.46 g, 1.11 mmol, 1 eq.) in water (1.6 mL) were separately heated to 70 °C. The solutions were subsequently combined, resulting in the formation of red globules due to the anion metathesis reaction producing the $[TFSI]^-$-bearing equivalent of [Im_{TEMPO}][Br], which is poorly soluble in water. The mixture was stirred for 2 h at 70 °C and then extracted with dichloromethane (5 x 5 mL). The organic extracts were combined and purified by washing in water (10 x 10 mL) to remove any excess lithium bromide formed during the anion metathesis. The aqueous washes were collected and subjected to a silver nitrate test to confirm the absence of bromide in the target product. Aqueous acidified solutions of silver nitrate can be used to detect chloride, bromide and iodide anions due to formation of the corresponding insoluble silver halide precipitate. The aqueous washes were performed until no silver bromide precipitate was formed. From this point, a further three washes were performed. The washed organic extracts were then combined and the solvent (dichloromethane) was removed by rotary evaporation to give a red viscous liquid. The liquid was subjected to a two-stage vacuum drying process, first at 10^{-2} mbar for 24 h at 100 °C, and then at 10^{-5} mbar for 24 h at the same temperature, to give [Im_{TEMPO}][TFSI] (**1c**) as a viscous red liquid (0.55 g, 81%). After the drying process, [Im_{TEMPO}][TFSI] was stored in an Ar-filled glovebox.

Synthesis of 1-(5-((1-oxyl-2,2,6,6-tetramethylpiperidin-4-yl)oxy)pentyl)-1-methylpyrrolidin-1-ium bromide ([$Pyrr_{TEMPO}$][Br], **2b**)

The same procedure for the synthesis of [Im_{TEMPO}][Br] (**1b**) was followed for the synthesis of [$Pyrr_{TEMPO}$][Br] with the following changes: A solution of 1-methylpyrrolidine (87.3 μL, 0.820 mmol, 1.2 eq.) in acetonitrile (1 mL) was added to a solution of TEMPO-C_5-Br (0.22 g, 0.683 mmol, 1 eq.) in acetonitrile (1 mL). [$Pyrr_{TEMPO}$][Br] (**2b**) was isolated as a coarse orange powder (0.20 g, 72%).

Synthesis of 1-(5-((1-oxyl-2,2,6,6-tetramethylpiperidin-4-yl)oxy)pentyl)-1-methylpyrrolidin-1-ium bis(trifluoromethane)sulfonimide ([$Pyrr_{TEMPO}$][TFSI], **2c**)

The anion metathesis was performed as described for the synthesis of [Im_{TEMPO}][TFSI], with [$Pyrr_{TEMPO}$][Br] instead of [Im_{TEMPO}][Br]. For this step, a solution of [$Pyrr_{TEMPO}$][Br] (0.12 g, 0.31 mmol, 1 eq.) in water (436 μL) at 70 °C was added to a solution of Li[TFSI] (0.088 g, 0.31 mmol, 1 eq.) in water (436 μL) at the same temperature. Reaction times, temperatures, work-ups and the drying procedure across all steps were as described for the synthetic steps carried out to yield [Im_{TEMPO}][TFSI]. This gave $Pyrr_{TEMPO}$][TFSI] (**2c**) as a red viscous liquid (0.12 g, 64%). After the drying process, it was stored in an Ar-filled glovebox.

Synthesis of 1-(5-((1-oxyl-2,2,6,6-tetramethylpiperidin-4-yl)oxy)pentyl)-1,4-diazabicyclo[2.2.2]octan-1-ium bromide ([D_{TEMPO}][Br], **3b**)

To synthesise the bromide salt, 1,4-diazabicyclo[2.2.2]octane (DABCO, 2.96 g, 0.026 mol, 2 eq.) was used as the tertiary amine and reacted with TEMPO-C_5-Br (4.15 g, 0.013 mol, 1 eq.) in acetonitrile (25 mL) at 75 °C. Due to the hygroscopic nature of DABCO, this reaction was performed under N_2 and for a longer reaction time (18 h) as compared to the synthesis of [Im_{TEMPO}][Br] and [$Pyrr_{TEMPO}$][Br]. The work-up and drying procedures were as described for the synthesis of [Im_{TEMPO}][Br]. The reaction yielded [D_{TEMPO}][Br] (5.27 g, 94%) as a coarse orange powder.

Synthesis of 1-(5-((1-oxyl-2,2,6,6-tetramethylpiperidin-4-yl)oxy)pentyl)-1,4-diazabicyclo[2.2.2]octan-1-ium bis(trifluoromethane)sulfonimide ([D_{TEMPO}][TFSI], **3c**)

[D_{TEMPO}][Br] (5.22 g, 0.012 mol, 1 eq.) was used in the anion metathesis reaction together with Li[TFSI] (3.46 g, 0.012 mol, 1 eq.). The reaction was run for 6 h, and the work-up and drying procedure was performed as described for [Im_{TEMPO}][TFSI] and [$Pyrr_{TEMPO}$][TFSI]. The isolated red liquid was subjected to a two-stage vacuum drying process, first at 10^{-2} mbar for 24 h at 100 °C, and then at 10^{-5} mbar for 24 h at the same temperature. This gave [D_{TEMPO}][TFSI] as a red liquid (6.90 g, 90%) that was noticeably more viscous than [Im_{TEMPO}][TFSI] and [$Pyrr_{TEMPO}$][TFSI]. After the drying process, it was stored in an Ar-filled glovebox.

Synthesis of 3-(5-((1-oxyl-2,2,6,6-tetramethylpiperidin-4-yl)oxy)pentyl)-1,2-dimethylimidazol-3-ium nitrate ([Im_{TEMPO}][NO_3], **4c**)

To synthesise [Im_{TEMPO}][NO_3], a solution of Ag[NO_3] (0.88 g, 0.005 mol, 1 eq.) in water (12 mL) was added to a solution of [Im_{TEMPO}][Br] (2.16 g, 0.005 mol, 1 eq.) in water (12 mL). Upon mixing of the two solutions, a white-yellow precipitate formed, which was attributed to the formation of silver bromide (AgBr). The mixture was stirred at room temperature for 4 h and AgBr was removed by vacuum filtration. After washing the aqueous filtrate with diethyl ether (4 x 20 mL), it was concentrated by rotary evaporation and subjected to a two-stage vacuum drying process, first at 10^{-2} mbar for 24 h at 100 °C, and then at 10^{-5} mbar for 24 h at the same temperature. This gave [Im_{TEMPO}][NO_3] as a coarse orange powder (1.71 g, 83%).

Qualitative confirmation of nitrate being present in the final product was attained by performing a nitrate test known as the brown ring test. In this test the nitrate containing compound is dissolved in an aqueous solution of iron (II) sulfate (Fe[SO_4]) and then treated with concentrated sulfuric acid (H_2SO_4). As the acid is denser, it sits below the aqueous phase. At the junction of the two phases, a brown ring is formed

if nitrate is present due to the formation of a nitrosyl ferrous sulfate complex. **Figure S1** illustrates the brown ring formed upon carrying out this qualitative test on [Im_{TEMPO}][NO_3] and the associated reactions taking place. The presence of nitrate was also quantitatively confirmed by electrospray ionisation mass spectrometry.

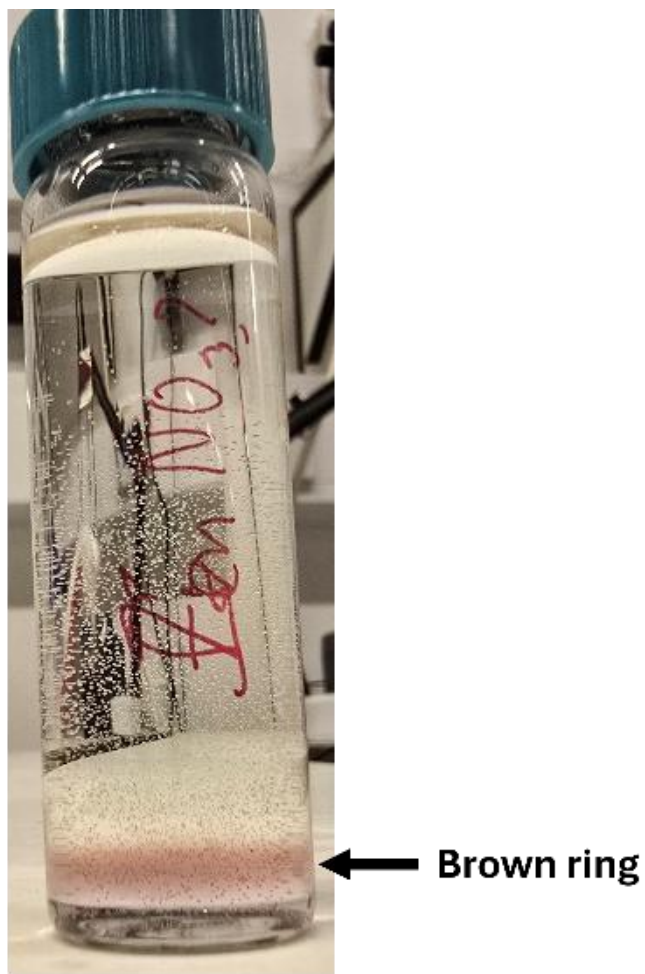

$$2\ \mathbf{H[NO_3]} + 3\ H_2SO_4 + 6\ Fe[SO_4] \rightarrow 3\ Fe_2[SO_4]_3 + 2\ NO + 4\ H_2O$$

$$[Fe(H_2O)_6][SO_4] + NO \rightarrow \mathbf{[Fe(H_2O)_5(NO)][SO_4]} + H_2O$$

Figure S1 | An image of the brown ring formed after treating an aqueous solution of Fe[SO_4] containing [Im_{TEMPO}][NO_3] with concentrated sulfuric acid due to the formation of nitrosyl ferrous sulfate complex (brown), as described by the chemical equations. In these example equations, nitric acid (HNO_3) serves as the nitrate source.

Scaled-up synthesis of imidazolium- and pyrrolidinium-based TEMPO-tethered redox mediators

For the purposes of scale-up, the modifications made to the synthetic procedures described above are summarised in **Table S1**. The bottleneck step in terms of maximising yield of the target RMs was the first step; formation of the TEMPO-C_5-Br precursor. The yield was typically reduced from ~60% to ~50% upon scale-up.

Table S1: A summary of modifications made to synthetic procedures with respect to reaction time, reaction temperature and limiting reagent amounts for the scaled-up syntheses of [Im_{TEMPO}][TFSI] and [$Pyrr_{TEMPO}$][TFSI].

Target product	Step no.	Time (h) / temp. (°C)	Limiting reagent amount (g)					Mass (g) and % yield
			1	2	2	3	4	
TEMPO-C_5-Br	1	24 / **24**<br>RT / **RT**	1.50 / **5.00**	-	-	-	-	1.72 / **4.65**<br>62 / **50**
[Im_{TEMPO}] [Br]	2	6 / **6**<br>60 / **70**	-	0.66 / **5.38**	-	-	-	0.60 / **4.39**<br>70 **/ 63**
[$Pyrr_{TEMPO}$] [Br]	2	6 / **15**<br>60 / **80**	-	-	0.22 / **4.64**	-	-	0.20 / **5.38**<br>72 / **92**
[Im_{TEMPO}] [TFSI]	3	2 / **6**<br>70 / **80**	-	-	-	0.46 / **4.28**	-	0.55 / **5.77**<br>81 / **91**
[$Pyrr_{TEMPO}$] [TFSI]	3	2 / **6**<br>70 / **80**	-	-	-	-	0.12 / **5.22**	0.12 / **7.25**<br>64 / **93**

1: TEMPO-OH, 2: TEMPO-C_5-Br, 3: [Im_{TEMPO}][Br] and 4: [$Pyrr_{TEMPO}$][Br]. Scaled-up reaction parameters are shown in bold font. The stoichiometric equivalents of other (non-limiting) reagents were kept the same between the low and higher scale syntheses (*e.g.*, if 2.1 eq. of 1,5-dibromopentane was used in the low-scale synthesis of TEMPO-C_5-Br, then 2.1 eq. was also used in the scaled-up synthesis). The step numbers 1, 2 and 3 refer to the steps outlined in **Schemes S1** and **S2** for the synthesis of [Im_{TEMPO}][TFSI] and [$Pyrr_{TEMPO}$][TFSI], respectively. Quoted yields are based on the final mass of purified target product in each step.

1D and 2D NMR characterisation

In this work, a range of 1D and 2D NMR experiments were performed for structural characterisation of all the synthesised RMs on an Avance III spectrometer (Bruker, US). The 1D experiments included ^{1}H and ^{13}C {^{1}H} NMR for initial structural assignment of synthesised RMs. These assignments were then confirmed using 2D methods included ^{1}H-^{1}H correlation spectroscopy (COSY) and ^{1}H-^{13}C edited heteronuclear single quantum coherence (HSQC). COSY was employed to study correlation between coupled proton environments and edited HSQC was used for assignment of ^{1}H-^{13}C single bond correlations and distinguishing between secondary carbon (CH_2) and tertiary/primary (CH_3/CH) carbon environments. All samples for NMR analysis were dissolved in deuterated dimethylsulfoxide (DMSO-d_6). For all analytes containing a radical in the structure, this was reduced by the addition of ~10 μL of phenylhydrazine (97%, Merck, UK), according to a published procedure.[1] This was done as the unpaired electron can interfere with NMR relaxation times, which is detrimental to peak resolution in the NMR spectrum. For sample preparation, the solvent to analyte ratio was approximately 0.065 and 0.0026 $mL_{solvent}\ mg_{analyte}^{-1}$ for ^{1}H and ^{13}C experiments, respectively.

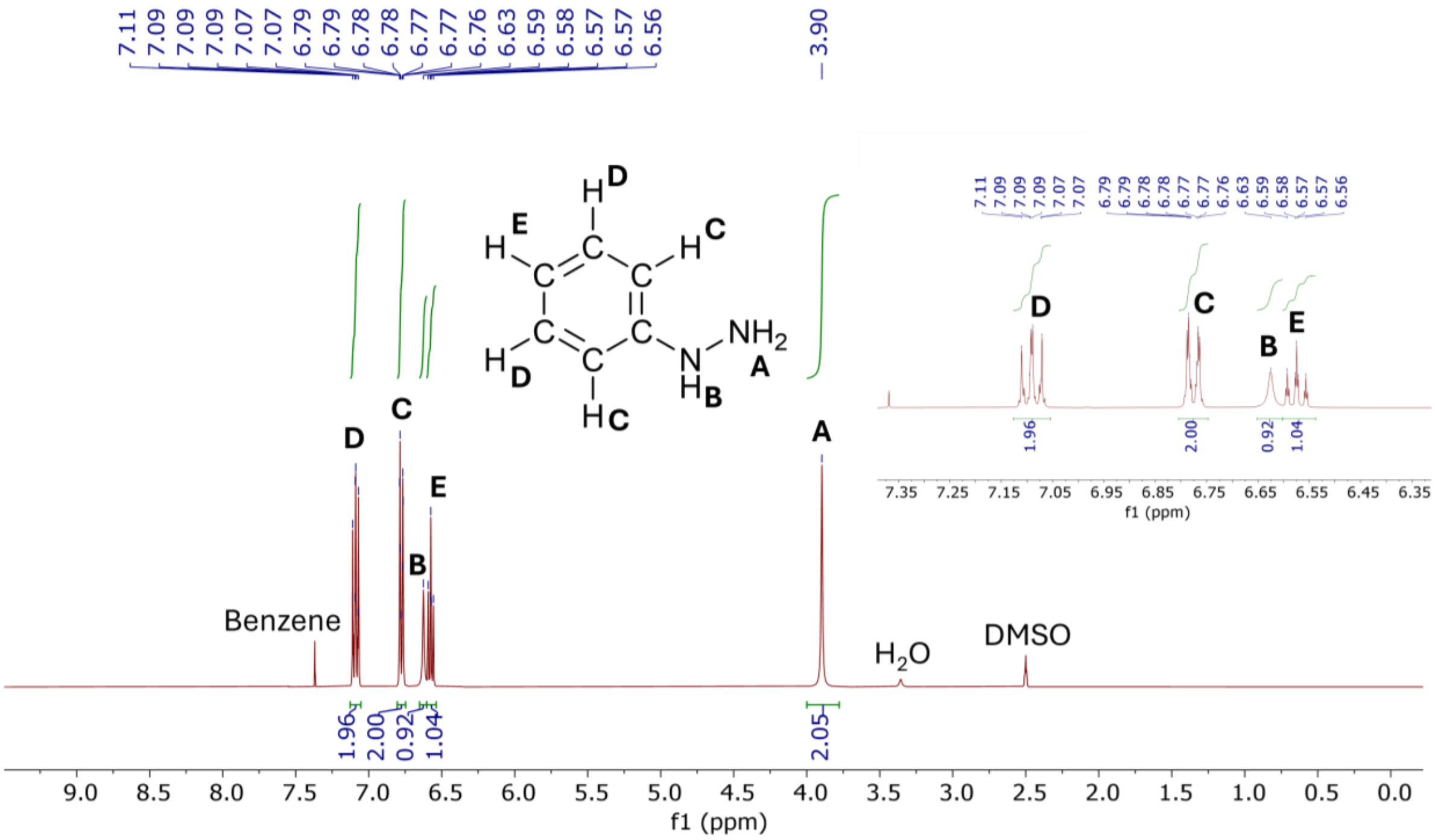

Figure S2 | ^{1}H NMR of phenylhydrazine ($PhNHNH_2$) in DMSO-d_6.

^{1}H NMR (400 MHz, DMSO-d_6): δ 3.90 (s, 2H, H_A), 6.56-6.59 (m, 1H, H_E), 6.63 (s, 1H, H_B), 6.76-6.79 (m, 2H, H_C), 7.07-7.11 (m, 2H, H_D).

Benzene is used in the synthesis of $PhNHNH_2$. Specifically, it is used in the purification of $PhNHNH_2$, after which benzene is removed by distillation.[2] However, it can exist as a trace contaminant in commercially prepared $PhNHNH_2$, which in this case had a quoted purity of 97%.

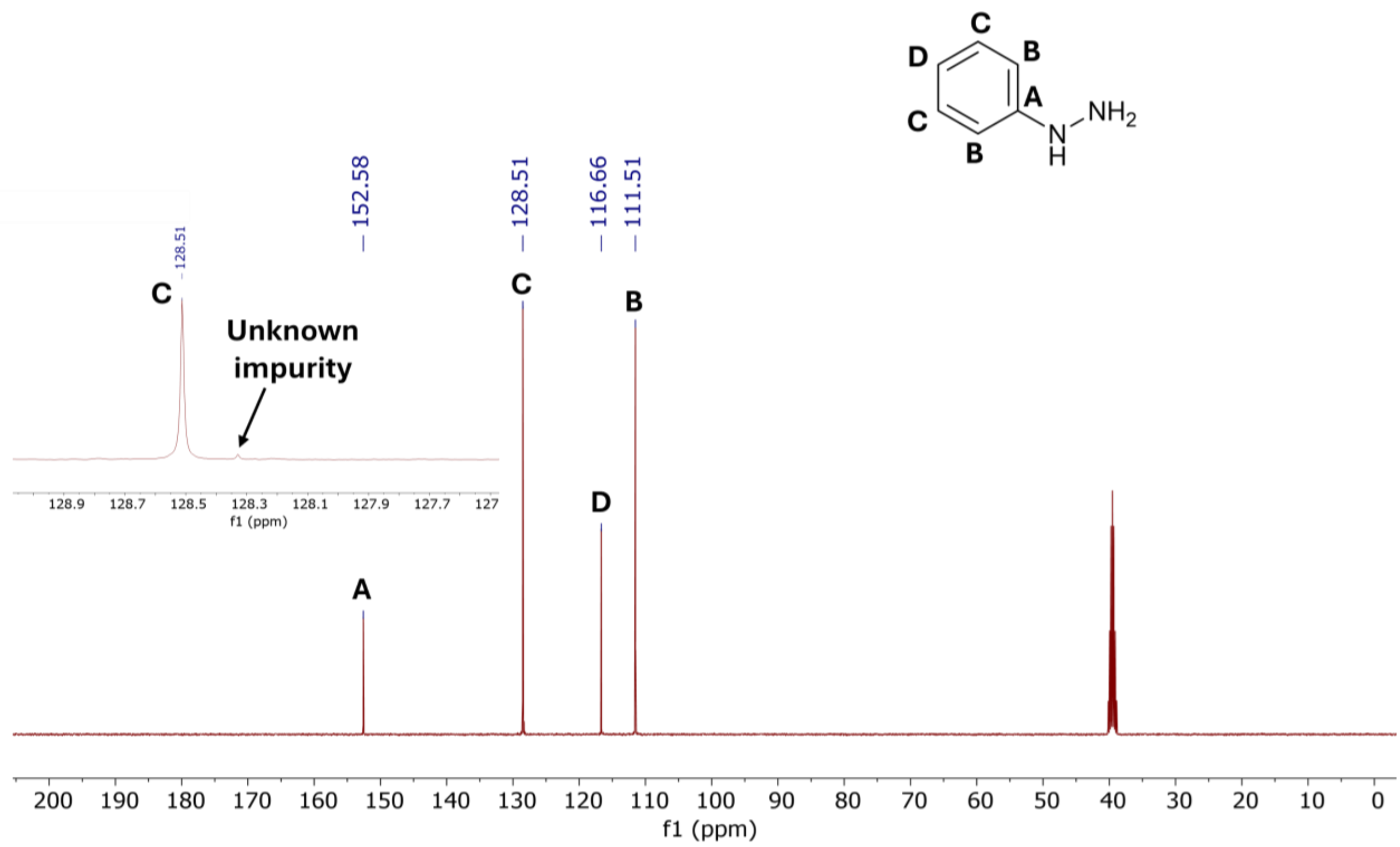

Figure S3 | ^{13}C {^{1}H} NMR of $PhNHNH_2$ in DMSO-d_6.

13C {^{1}H} NMR (100 MHz, DMSO-d_6): δ 111.51 (C_B), 116.66 (C_D), 128.51 (C_C), 152.58 (C_A).

The unknown impurity was not found to affect the reduction of the nitroxyl radical by $PhNHNH_2$ in the TEMPO-tethered RMs.

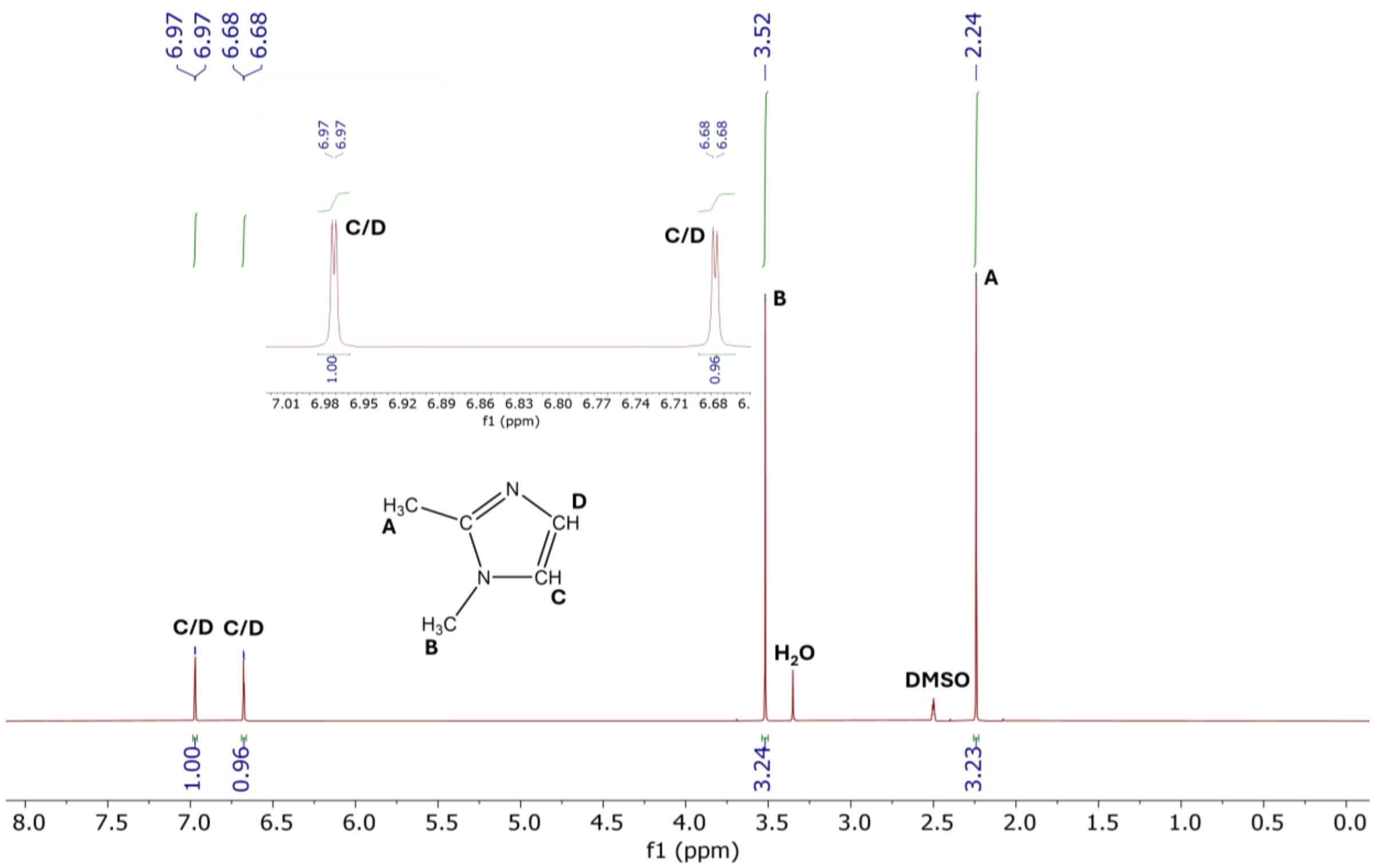

Figure S4 | ^{1}H NMR of 1,2-dimethylimidazole in DMSO-d_6.

^{1}H NMR (400 MHz, DMSO-d_6): δ 2.24 (s, 3H, H_A), 3.52 (s, 3H, H_B), 6.68 (d, 1H, H_C/H_D), 6.97 (d, 1H, H_C/H_D).

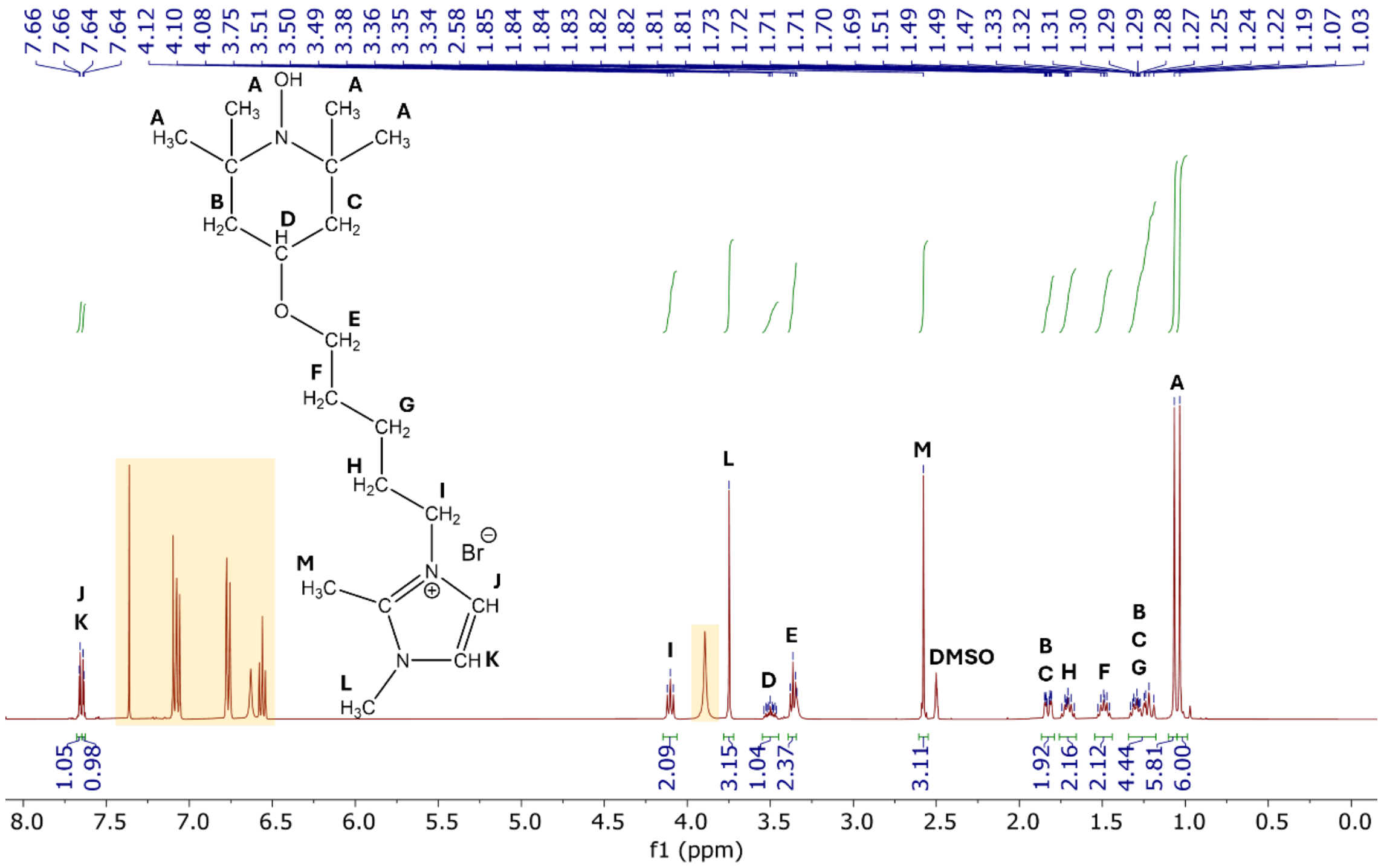

Figure S5 | ^{1}H NMR of [Im$_{TEMPO}$][Br] (**1b**), prepared *via* the low scale synthetic route, in DMSO-d$_6$.

^{1}H NMR (400 MHz, DMSO-d$_6$): δ 1.03 (s, 6H, H$_A$), 1.07 (s, 6H, H$_A$), 1.19-1.33 (m, 4H, H$_B$, H$_C$, H$_G$), 1.47-1.51 (m, 2H, H$_F$), 1.69-1.73 (m, 2H, H$_H$), 1.81-1.85 (m, 2H, H$_B$, H$_C$), 2.58 (s, 3H, H$_M$), 3.36 (t, 2H, H$_E$), 3.49-3.51 (m, 1H, H$_D$), 3.75 (s, 3H, H$_L$), 4.10 (t, 2H, H$_I$), 7.64 (d, 1H, H$_J$/H$_K$), 7.66 (d, 1H, H$_J$/H$_K$).

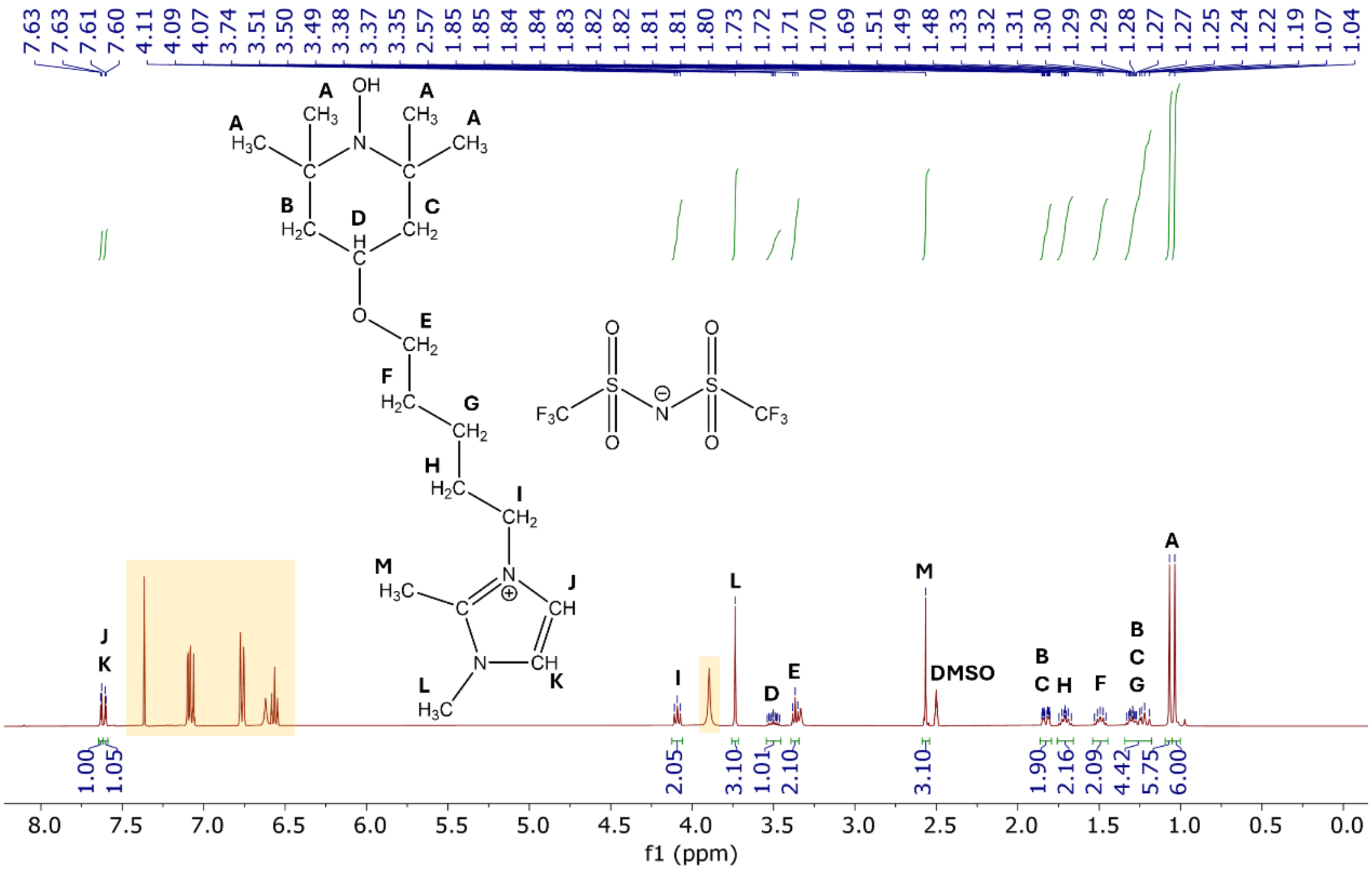

Figure S6 | ^{1}H NMR of [Im$_{TEMPO}$][TFSI] (**1c**), prepared *via* the low scale synthetic route, dissolved in DMSO-d$_6$.

^{1}H NMR (400 MHz, DMSO-d$_6$): δ 1.04 (s, 6H, H_A), 1.07 (s, 6H, H_A), 1.19-1.33 (m, 4H, H_B, H_C, H_G), 1.48-1.51 (m, 2H, H_F), 1.69-1.73 (m, 2H, H_H), 1.80-1.85 (m, 2H, H_B, H_C), 2.57 (s, 3H, H_M), 3.37 (t, 2H, H_E), 3.49-3.51 (m, 1H, H_D), 3.74 (s, 3H, H_L), 4.09 (t, 2H, H_I), 7.61 (d, 1H, H_J/H_K), 7.63 (d, 1H, H_J/H_K).

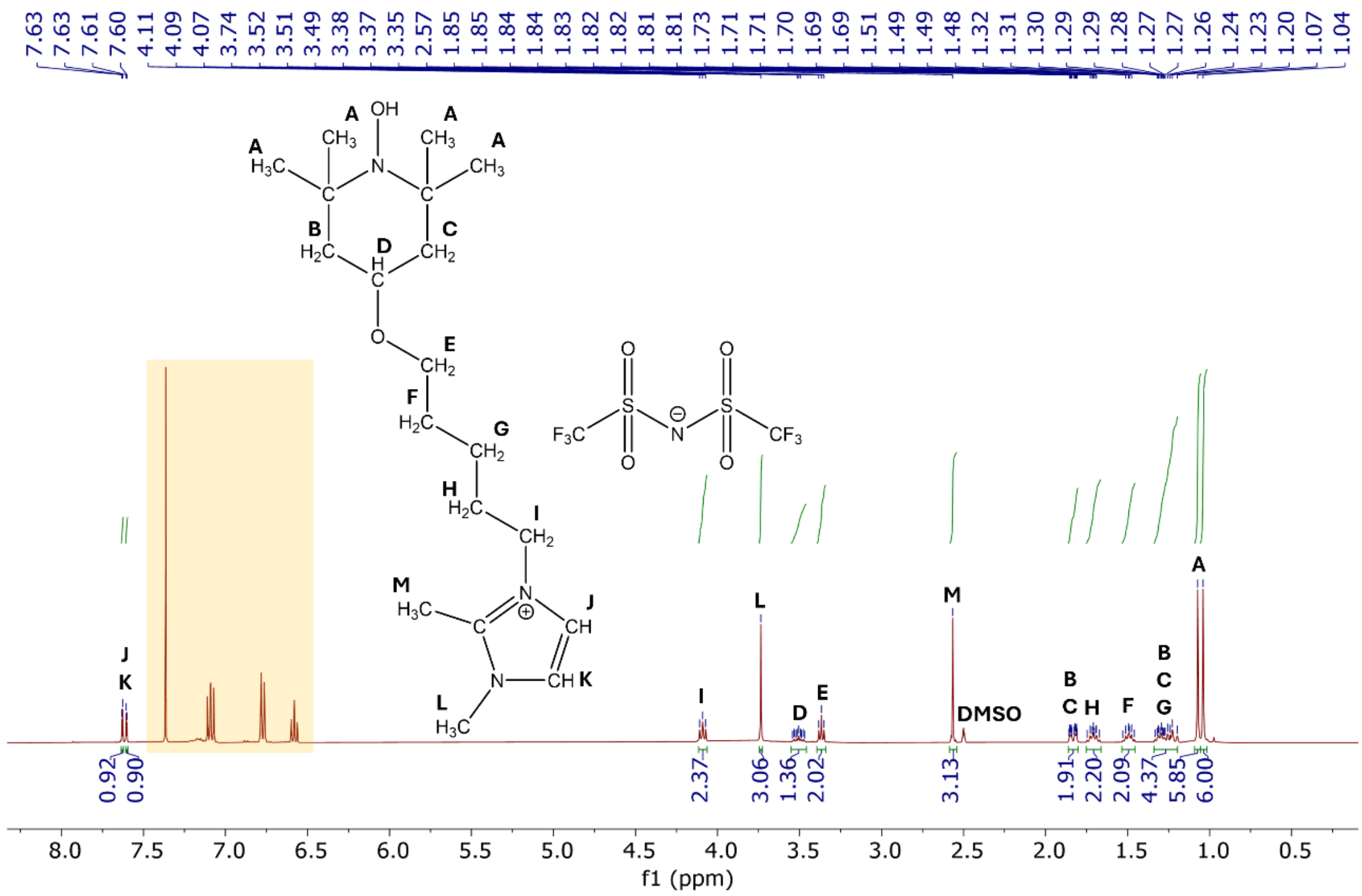

Figure S7 | ^{1}H NMR of [Im$_{TEMPO}$][TFSI] (**1c**), prepared *via* the scaled-up synthetic route, dissolved in DMSO-d_6.

^{1}H NMR (400 MHz, DMSO-d_6): δ 1.04 (s, 6H, H_A), 1.07 (s, 6H, H_A), 1.20-1.32 (m, 4H, H_B, H_C, H_G), 1.48-1.51 (m, 2H, H_F), 1.69-1.73 (m, 2H, H_H), 1.81-1.85 (m, 2H, H_B, H_C), 2.57 (s, 3H, H_M), 3.37 (t, 2H, H_E), 3.49-3.52 (m, 1H, H_D), 3.74 (s, 3H, H_L), 4.09 (t, 2H, H_I), 7.61 (d, 1H, H_J/H_K), 7.63 (d, 1H, H_J/H_K).

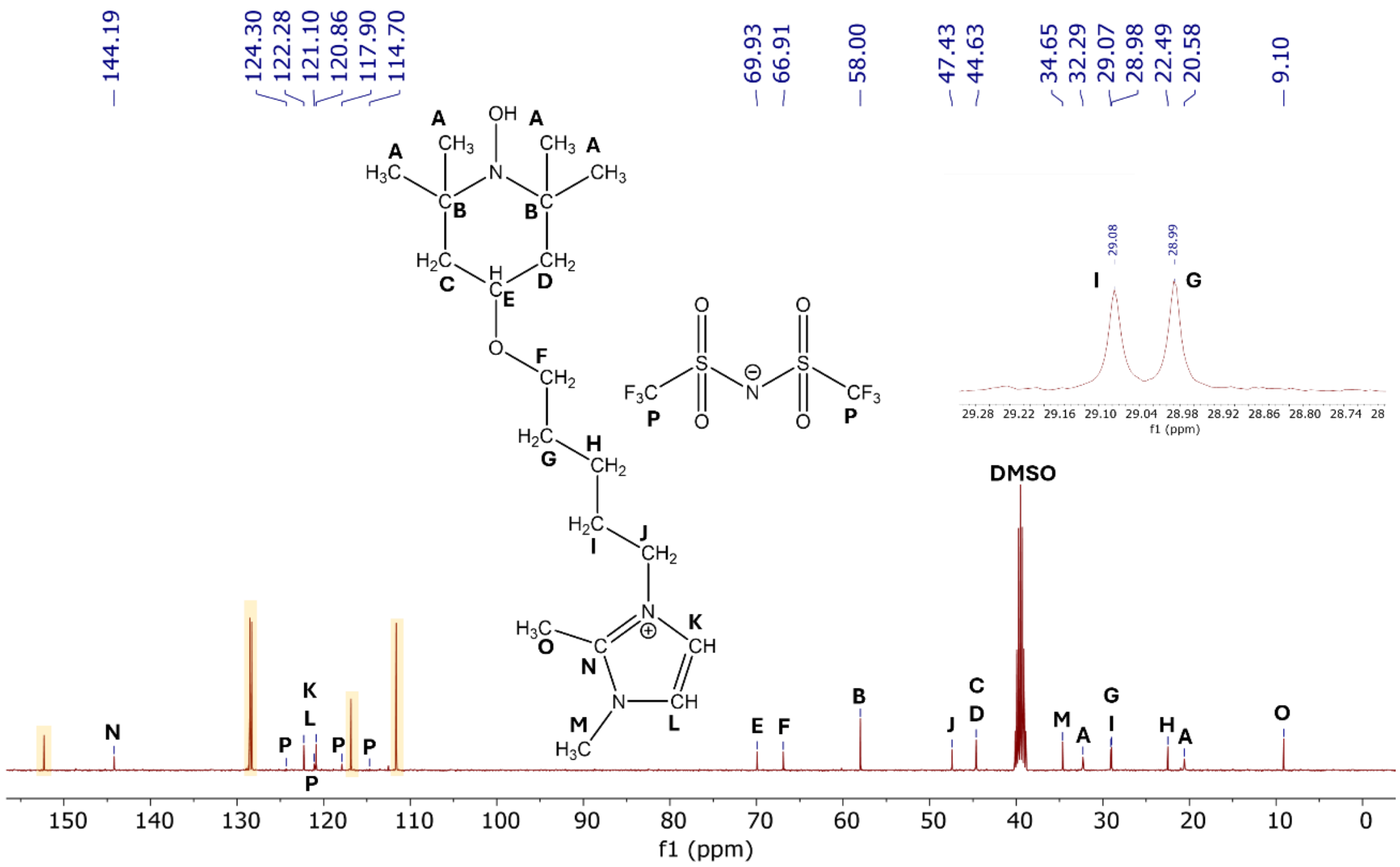

Figure S8 | ^{13}C {^{1}H} NMR of [Im$_{TEMPO}$][TFSI] (**1c**), prepared *via* the scaled-up synthetic route, in DMSO-d$_6$.

^{13}C {^{1}H} NMR (100 MHz, DMSO-d$_6$): δ 9.10 (C$_O$), 20.58 (C$_A$), 22.49 (C$_H$), 28.98 (C$_G$), 29.07 (C$_I$), 32.29 (C$_A$), 34.65 (C$_M$), 44.63 (C$_C$, C$_D$), 47.43 (C$_J$), 58.00 (C$_B$), 66.91 (C$_F$), 69.93 (C$_E$), 119.5 (q, C$_P$, J = 322 Hz), 120.86 (C$_K$/C$_L$), 122.28 (C$_K$/C$_L$), 144.19 (C$_N$).

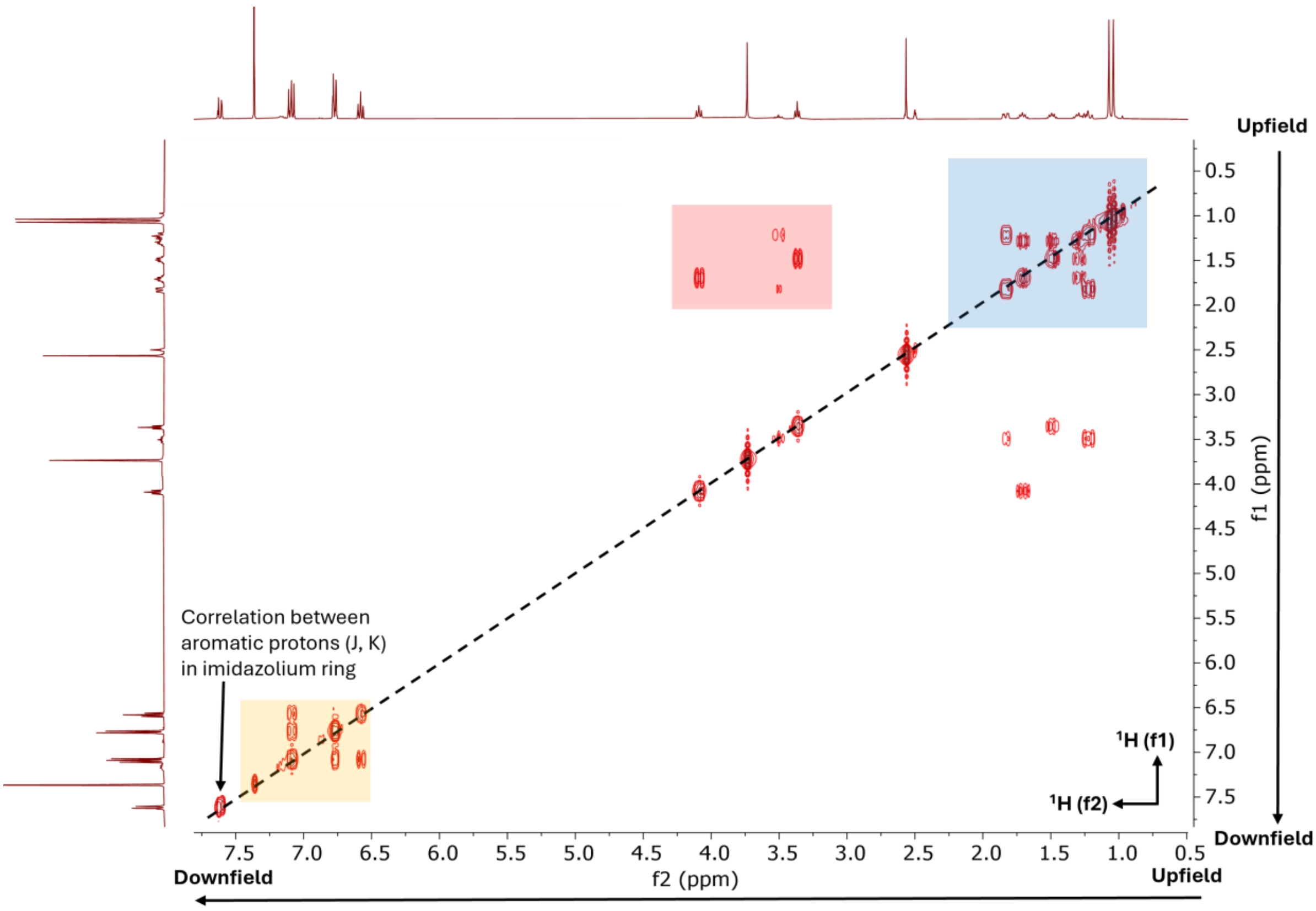

Figure S9 | Full-scale 2D ^{1}H-^{1}H COSY NMR spectrum of [Im$_{TEMPO}$][TFSI] (**1c**), prepared *via* the scaled-up synthetic route, dissolved in DMSO-d$_6$. The cross peaks highlighted in yellow correspond to $PhNHNH_2$. Cross peaks on the dotted line indicate self-correlation cross peaks. The blue and red shaded regions represent upfield and downfield regions of the 2D spectrum that are presented as zoomed-in windows in **Figures S10** and **S11**, respectively.

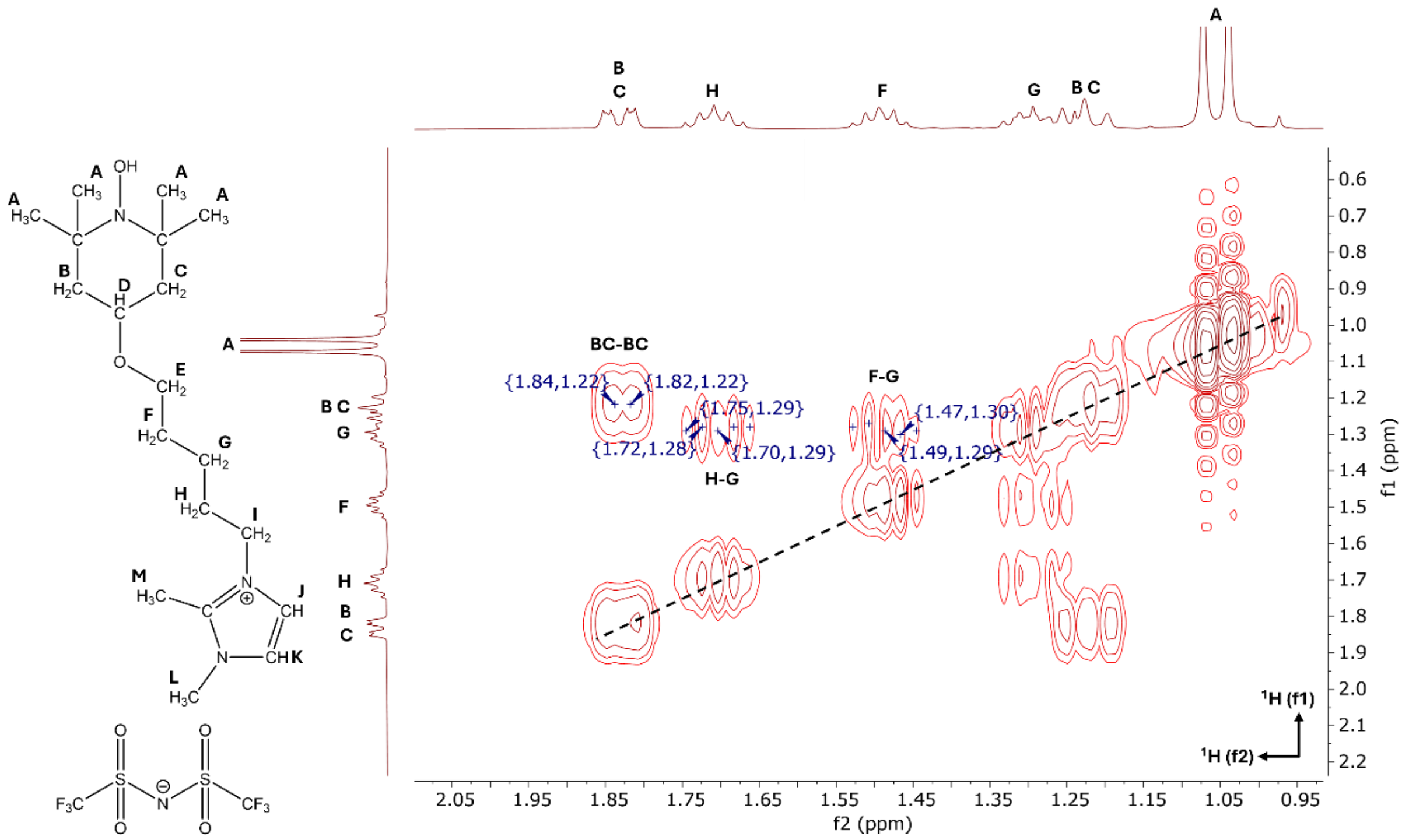

Figure S10 | Zoomed-in view of the upfield region (shaded in blue in **Figure S9**) of the 2D ^{1}H-^{1}H COSY NMR spectrum of [Im$_{TEMPO}$][TFSI] (**1c**) presented in **Figure S9**.

2D ^{1}H-^{1}H COSY NMR (400 MHz, DMSO-d$_6$):

**f$_2$ (ppm)**	**f$_1$ (ppm)**	**^{1}H-^{1}H correlation**
1.49	1.29	H$_F$-H$_G$
1.47	1.30	
1.70	1.29	H$_H$-H$_G$
1.75	1.29	
1.72	1.28	
1.84	1.22	H$_B$H$_C$-H$_B$H$_C$
1.82	1.22	

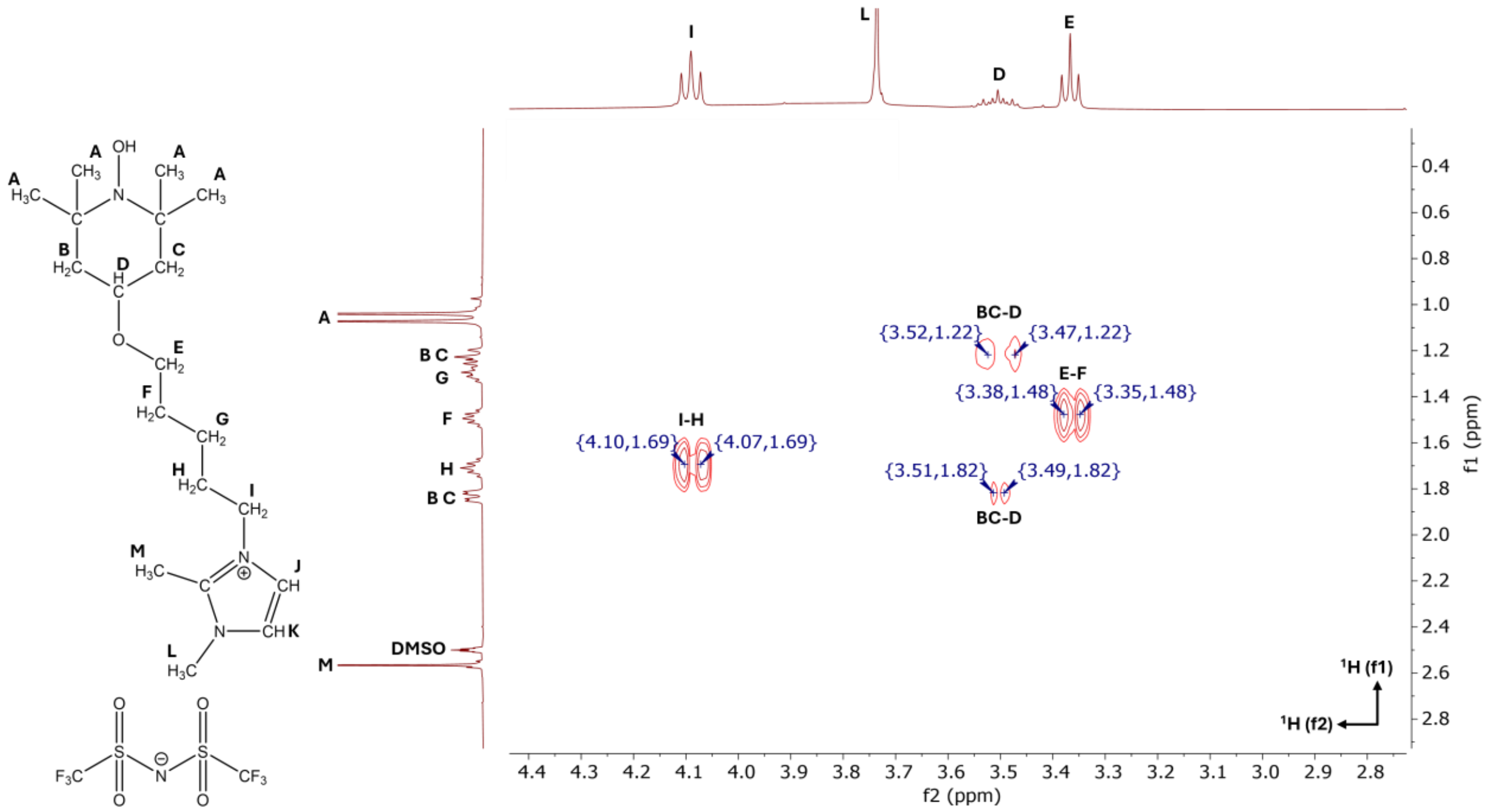

Figure S11 | Zoomed-in view of the downfield region (shaded in red in **Figure S9**) of the 2D ^1H-^1H COSY NMR spectrum of $[Im_{TEMPO}]$[TFSI] (**1c**) presented in **Figure S9**.

2D ^1H-^1H COSY NMR (400 MHz, DMSO-d_6):

f_2 (ppm)	f_1 (ppm)	^1H-^1H correlation
3.38	1.48	H_E-H_F
3.35	1.48	
3.52	1.22	H_BH_C-H_D
3.57	1.22	
3.51	1.82	
3.49	1.82	
4.10	1.69	H_H-H_I
4.07	1.69	

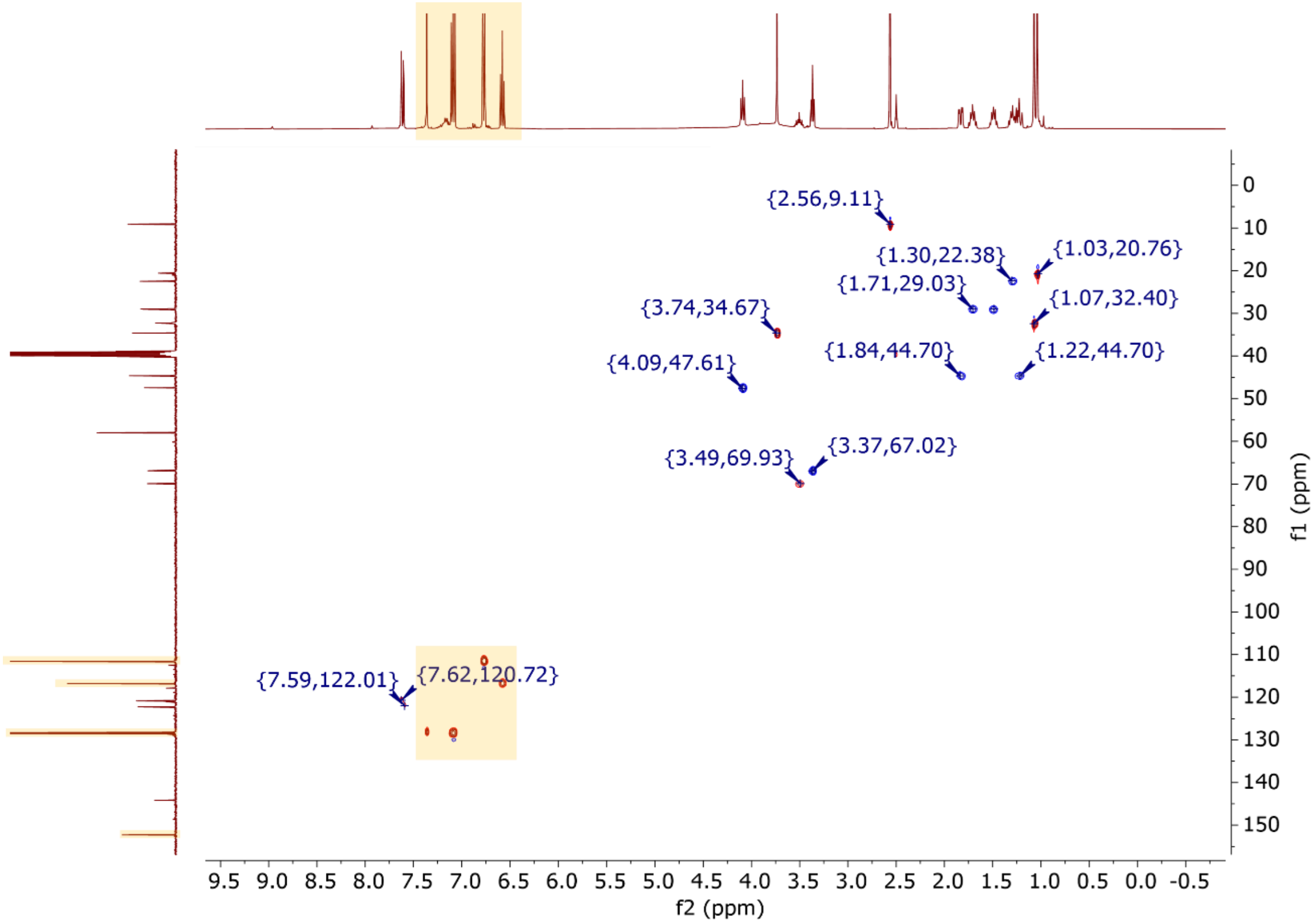

Figure S12 | Full-scale 2D ^{1}H-^{13}C edited HSQC NMR spectrum of [Im_{TEMPO}][TFSI] (**1c**), prepared *via* the scaled-up synthetic route, in DMSO-d_6. The shaded yellow regions represent peaks/cross peaks related to $PhNHNH_2$. Blue cross peaks indicate that these correlations stem from CH_2 (secondary carbon) units and red cross peaks indicate that the carbon is either tertiary (CH) or primary (CH_3).

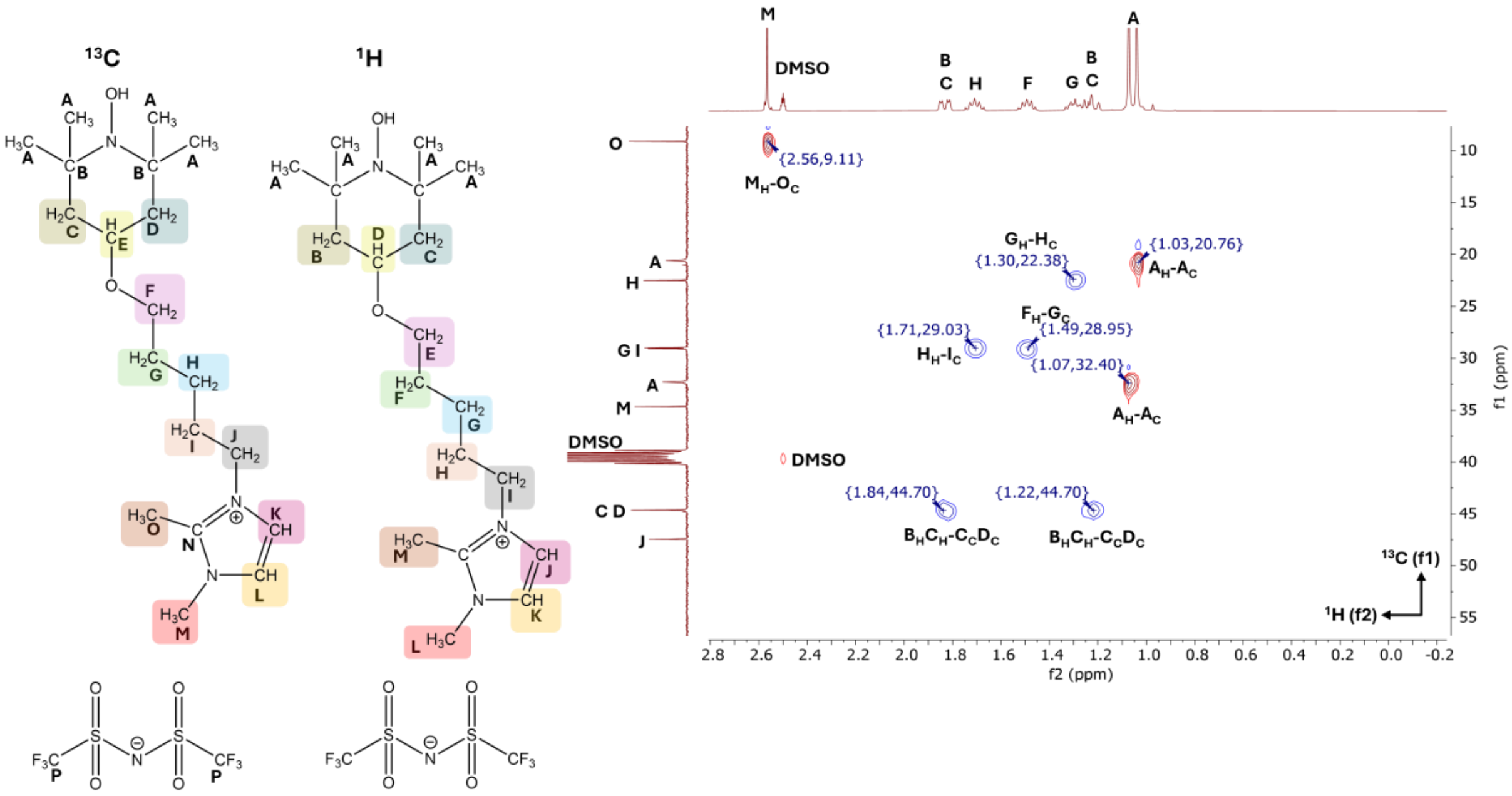

Figure S13 | Zoomed-in region 1 of the 2D ^1H-^{13}C edited HSQC NMR spectrum of [Im$_{TEMPO}$][TFSI] (**1c**) in DMSO-d$_6$. Blue cross peaks indicate that these correlations stem from CH_2 (secondary carbon) units and red cross peaks indicate that the carbon is either tertiary (CH) or primary (CH_3).

2D ^1H-^{13}C edited HSQC NMR (400 MHz (^1H), 100 MHz (^{13}C), DMSO-d$_6$):

f$_2$, ^1H (ppm)	f$_1$, ^{13}C (ppm)	^1H-^{13}C correlation
1.03	20.76	A$_H$-A$_C$
1.07	32.40	
1.30	22.38	G$_H$-H$_C$
1.49	28.95	F$_H$-G$_C$
1.71	29.03	H$_H$-I$_C$
1.22	44.70	B$_H$C$_H$-C$_C$D$_C$
1.84	44.70	
2.56	9.11	M$_H$-O$_C$

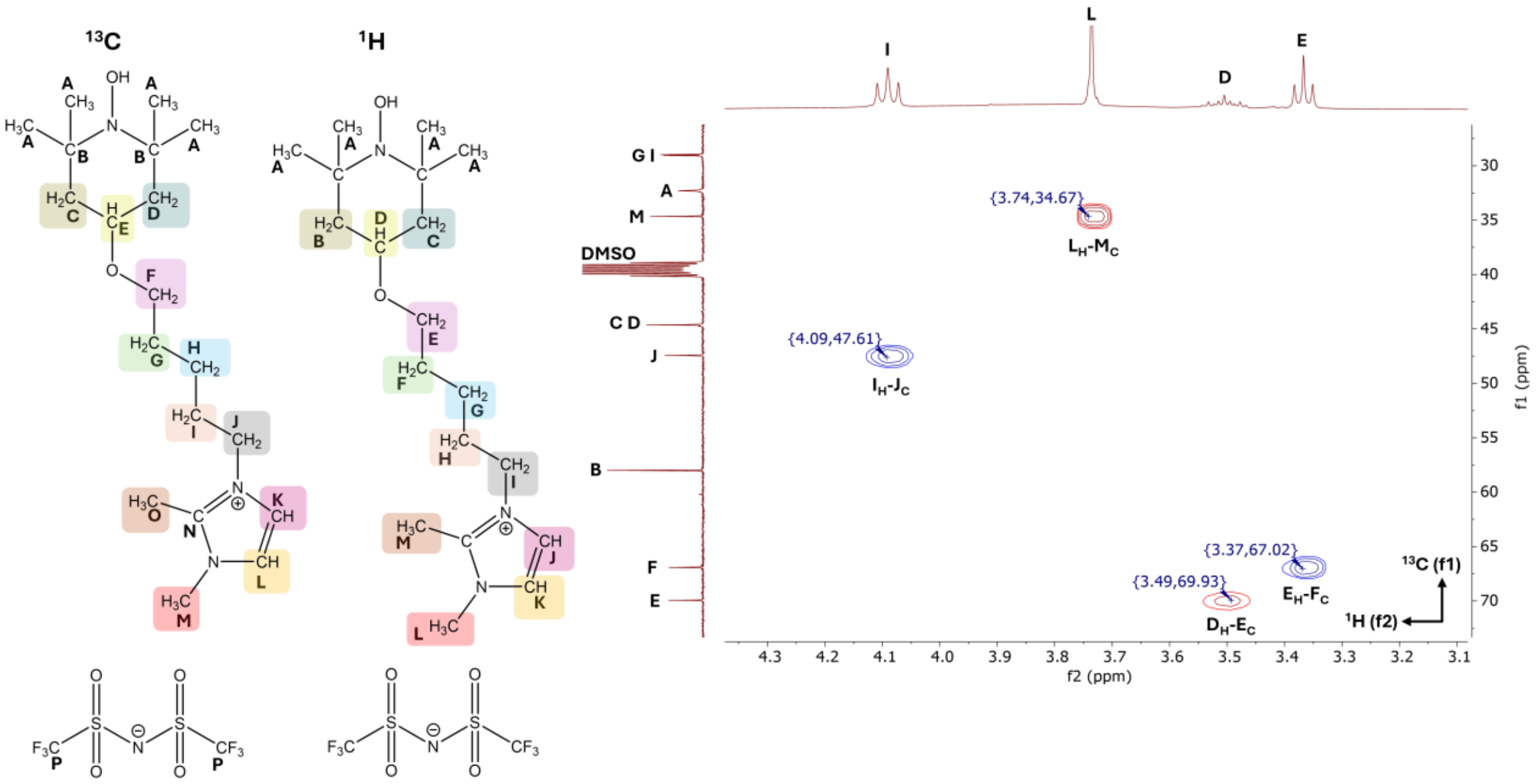

Figure S14 | Zoomed-in region 2 of the 2D ^{1}H-^{13}C edited HSQC NMR spectrum of [Im$_{TEMPO}$][TFSI] (**1c**) in DMSO-d$_6$. Blue cross peaks indicate that these correlations stem from CH_2 (secondary carbon) units and red cross peaks indicate that the carbon is either tertiary (CH) or primary (CH_3).

2D ^{1}H-^{13}C edited HSQC NMR (400 MHz (^{1}H), 100 MHz (^{13}C), DMSO-d$_6$):

**f$_2$, ^{1}H (ppm)**	**f$_1$, ^{13}C (ppm)**	**^{1}H-^{13}C correlation**
3.37	67.02	E_H-F_C
3.49	69.93	D_H-E_C
3.74	34.67	L_H-M_C
4.09	47.61	I_H-J_C

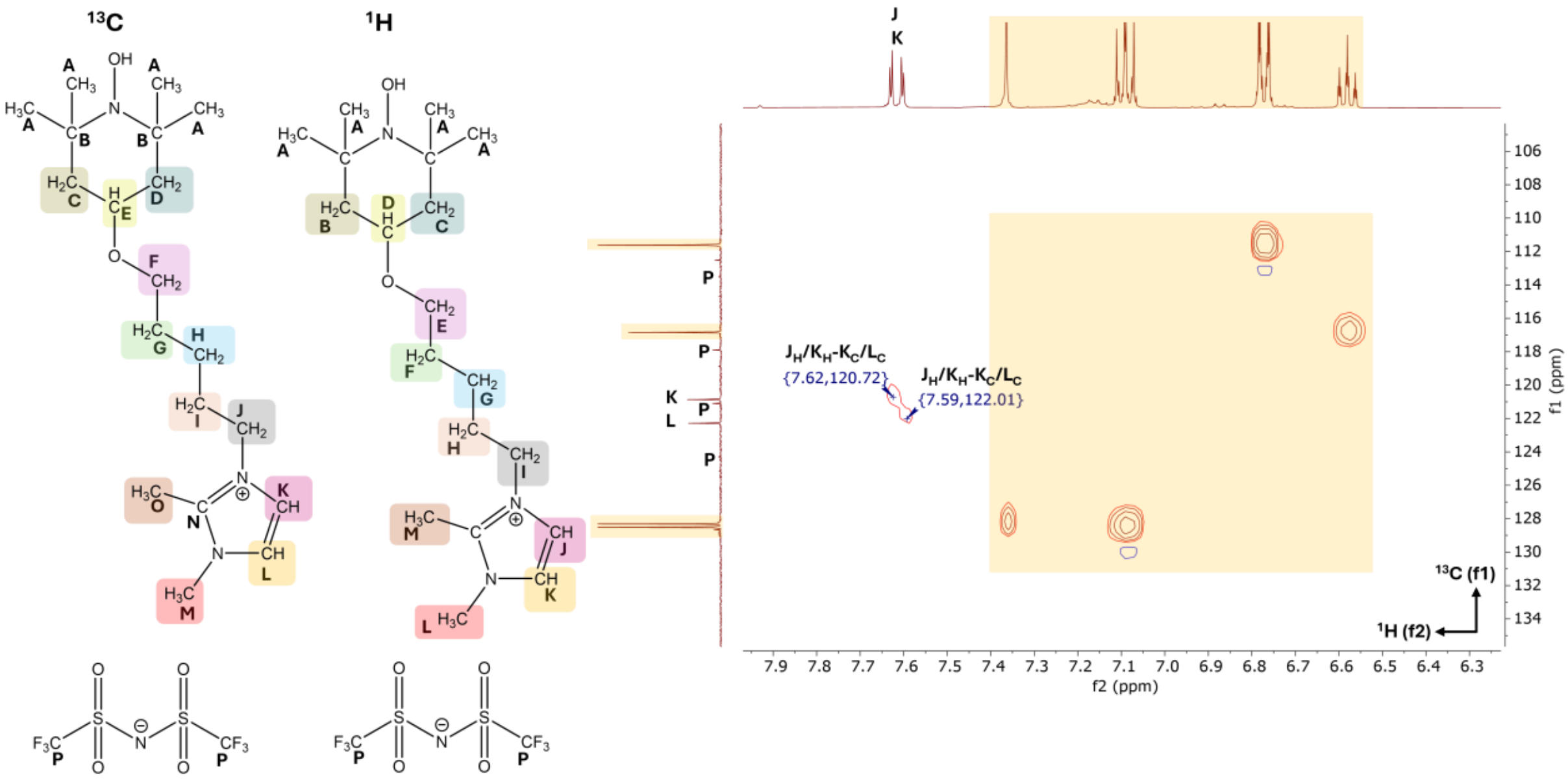

Figure S15 | Zoomed-in region 3 of the 2D ^{1}H-^{13}C edited HSQC NMR spectrum of [Im$_{TEMPO}$][TFSI] (**1c**) in DMSO-d$_6$. The shaded yellow regions represent peaks/cross peaks related to $PhNHNH_2$. Blue cross peaks indicate that these correlations stem from CH_2 (secondary carbon) units and red cross peaks indicate that the carbon is either tertiary (CH) or primary (CH_3).

2D ^{1}H-^{13}C edited HSQC NMR (400 MHz (^{1}H), 100 MHz (^{13}C), DMSO-d$_6$):

**f$_2$, ^{1}H (ppm)**	**f$_1$, ^{13}C (ppm)**	**^{1}H-^{13}C correlation**
7.62	120.72	J_H/K_H-K_C/L_C
7.59	122.01	

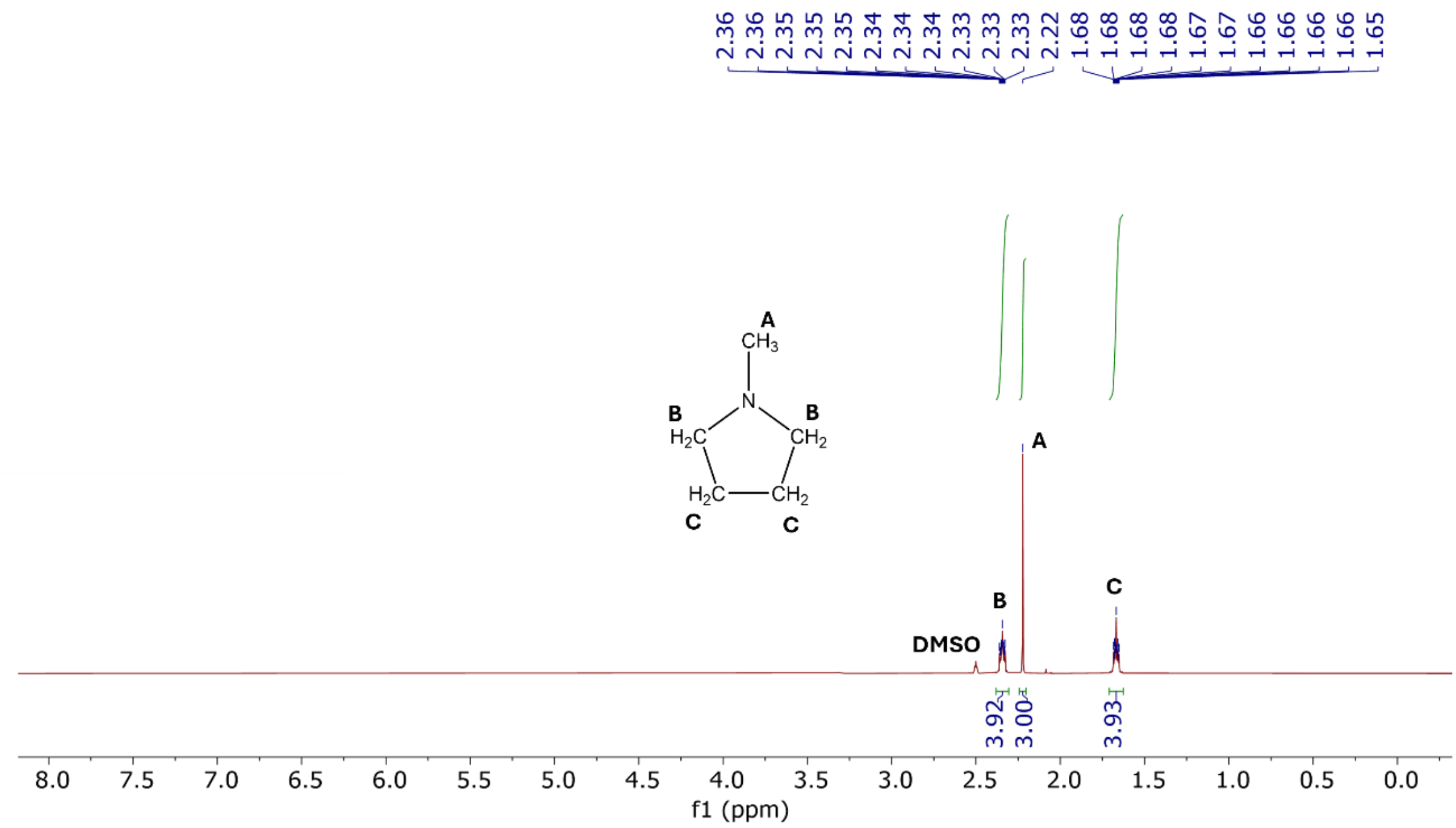

Figure S16 | ^{1}H NMR spectrum of 1-methylpyrrolidine in DMSO-d$_6$.

^{1}H NMR (400 MHz, DMSO-d$_6$): δ 1.65-1.68 (m, 4H, H$_C$), 2.22 (s, 3H, H$_A$), 2.33-2.36 (m, 4H, H$_B$).

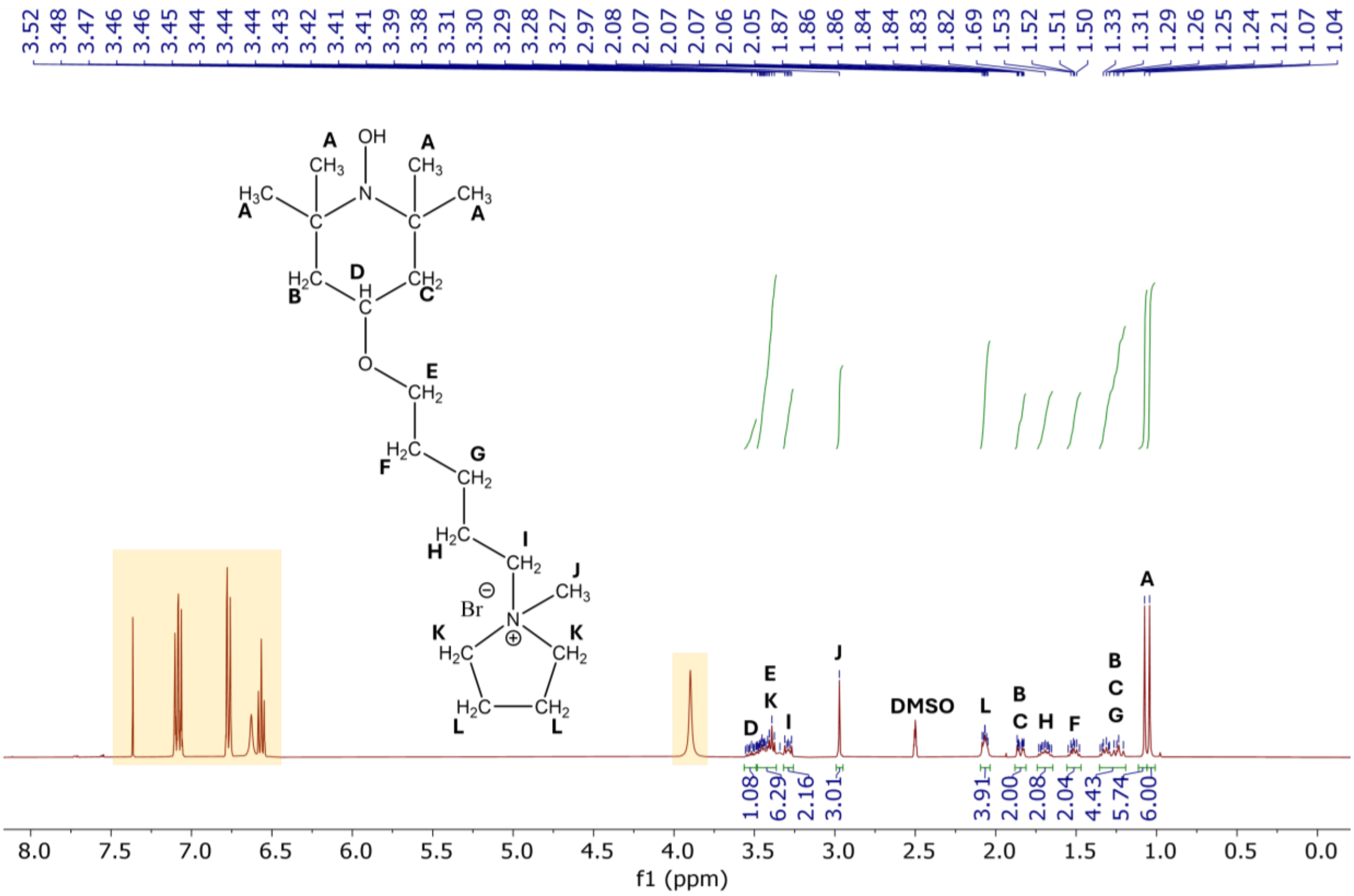

Figure S17 | ^{1}H NMR of [Pyrr$_{TEMPO}$][Br] (**2b**), prepared *via* the low scale synthetic route, in DMSO-d$_6$. Peaks shaded in yellow correspond to $PhNHNH_2$.

^{1}H NMR (400 MHz, DMSO-d$_6$): δ 1.04 (s, 6H, H_A), 1.07 (s, 6H, H_A), 1.21-1.35 (m, 4H, H_B, H_C, H_G), 1.48-1.55 (m, 2H, H_F), 1.65-1.73 (m, 2H, H_H), 1.82-1.87 (m, 2H, H_B, H_C), 2.05-2.08 (m, 4H, H_L), 2.97 (s, 3H, H_J), 3.27-3.31 (m, 2H, H_I), 3.38-3.48 (m, 6H, H_E, H_K), 3.50-3.56 (m, 1H, H_D).

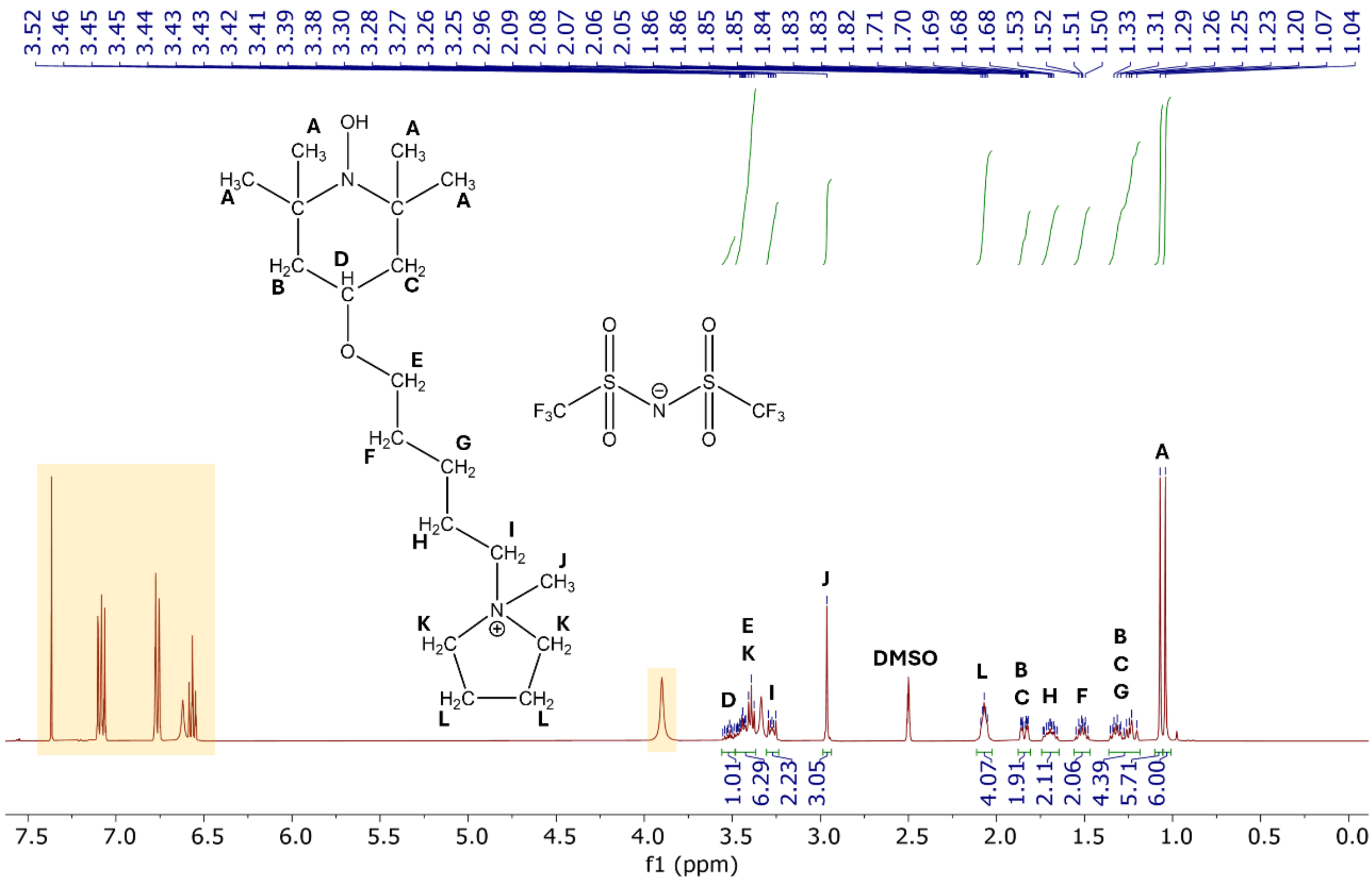

Figure S18 | ^{1}H NMR of [Pyrr$_{TEMPO}$][TFSI] (**2c**), prepared *via* the low scale synthetic route, dissolved in DMSO-d$_6$. Peaks shaded in yellow correspond to PhNHNH$_2$.

^{1}H NMR (400 MHz, DMSO-d$_6$): δ 1.04 (s, 6H, H$_A$), 1.07 (s, 6H, H$_A$), 1.20-1.33 (m, 4H, H$_B$, H$_C$, H$_G$), 1.50-1.53 (m, 2H, H$_F$), 1.68-1.71 (m, 2H, H$_H$), 1.82-1.86 (m, 2H, H$_B$, H$_C$), 2.05-2.09 (m, 4H, H$_L$), 2.96 (s, 3H, H$_J$), 3.25-3.30 (m, 2H, H$_I$), 3.37-3.47 (m, 6H, H$_E$, H$_K$), 3.49-3.55 (m, 1H, H$_D$).

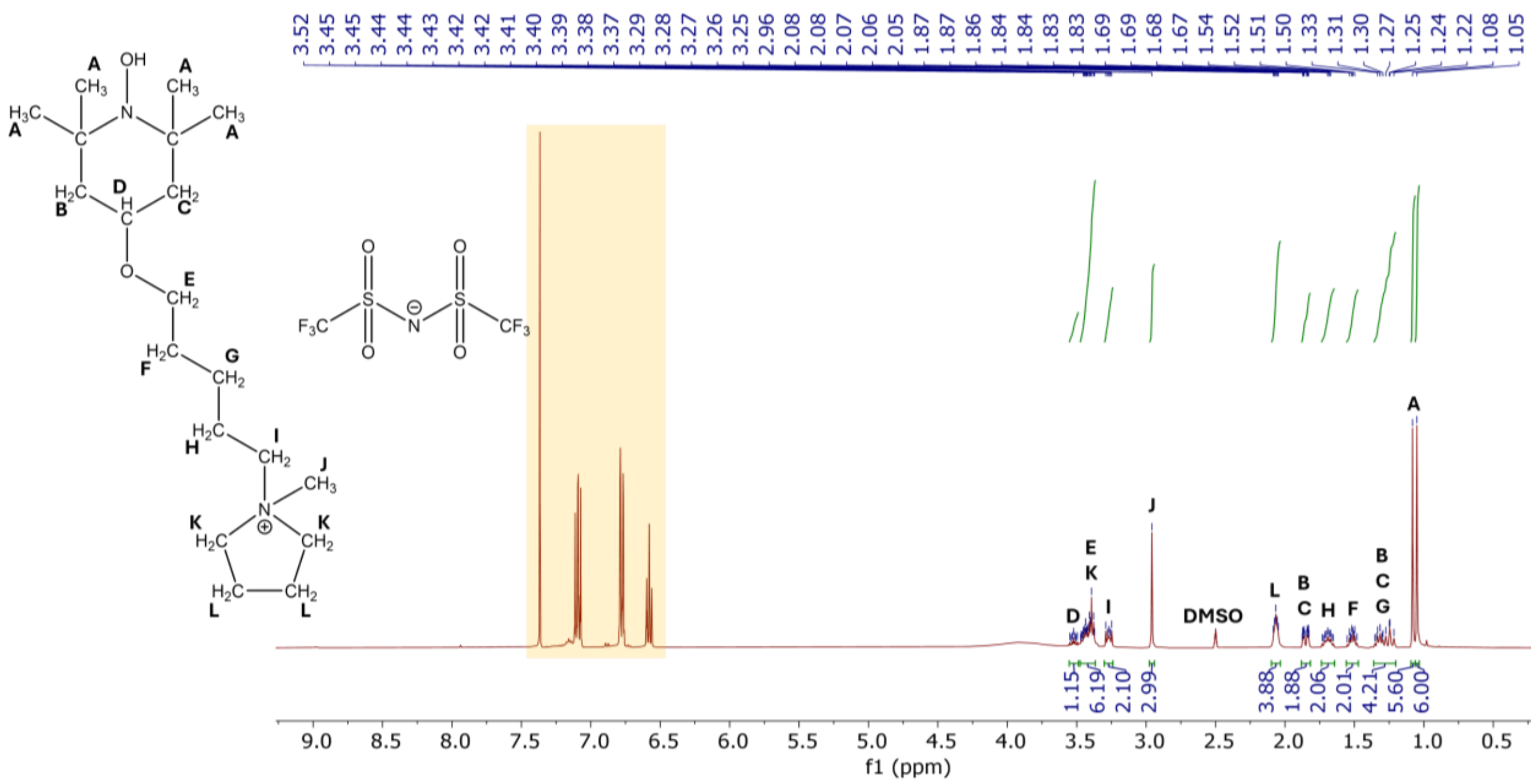

Figure S19 | ^{1}H NMR of [Pyrr$_{TEMPO}$][TFSI] (**2c**), prepared *via* the scaled-up synthetic route, dissolved in DMSO-d$_6$. Peaks shaded in yellow correspond to PhNHNH$_2$.

^{1}H NMR (400 MHz, DMSO-d$_6$): δ 1.05 (s, 6H, H$_A$), 1.08 (s, 6H, H$_A$), 1.22-1.35 (m, 4H, H$_B$, H$_C$, H$_G$), 1.50-1.55 (m, 2H, H$_F$), 1.65-1.73 (m, 2H, H$_H$), 1.83-1.88 (m, 2H, H$_B$, H$_C$), 2.05-2.08 (m, 4H, H$_L$), 2.96 (s, 3H, H$_J$), 3.25-3.29 (m, 2H, H$_I$), 3.37-3.47 (m, 6H, H$_E$, H$_K$), 3.49-3.55 (m, 1H, H$_D$).

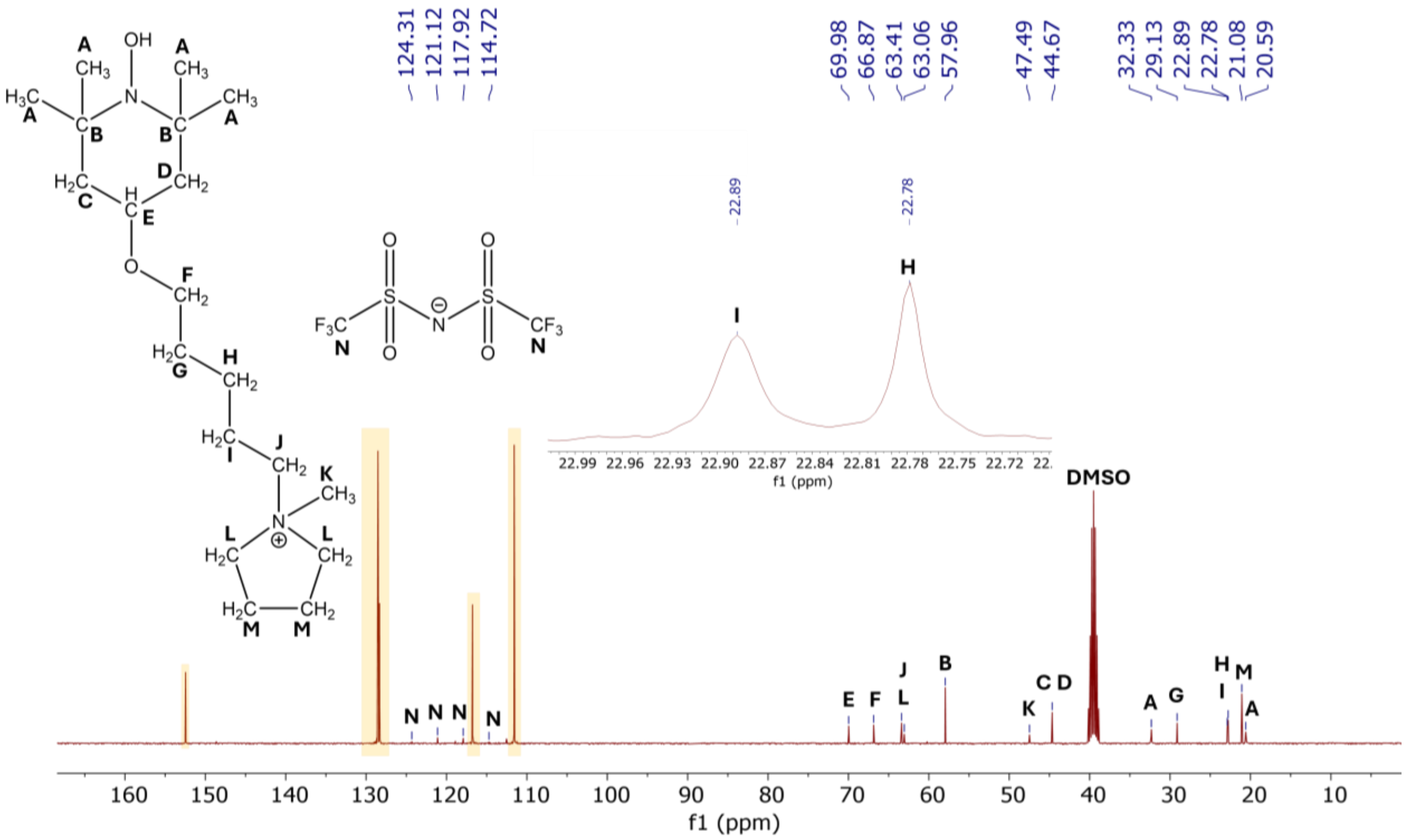

Figure S20 | ^{13}C {^{1}H} NMR of [Pyrr$_{TEMPO}$][TFSI] (**2c**), prepared *via* the scaled-up synthetic route, in DMSO-d$_6$. Peaks shaded in yellow correspond to PhNHNH$_2$.

13C {^{1}H} NMR (100 MHz, DMSO-d$_6$): δ 20.59 (C_A), 21.08 (C_M), 22.78 (C_H), 22.89 (C_I), 29.13 (C_G), 32.33 (C_A), 44.67 (C_C, C_D), 47.49 (C_K), 57.96 (C_B), 63.06 (C_J), 63.41 (C_L), 66.87 (C_F), 69.98 (C_E), 119.5 (q, C_N, J = 322 Hz).

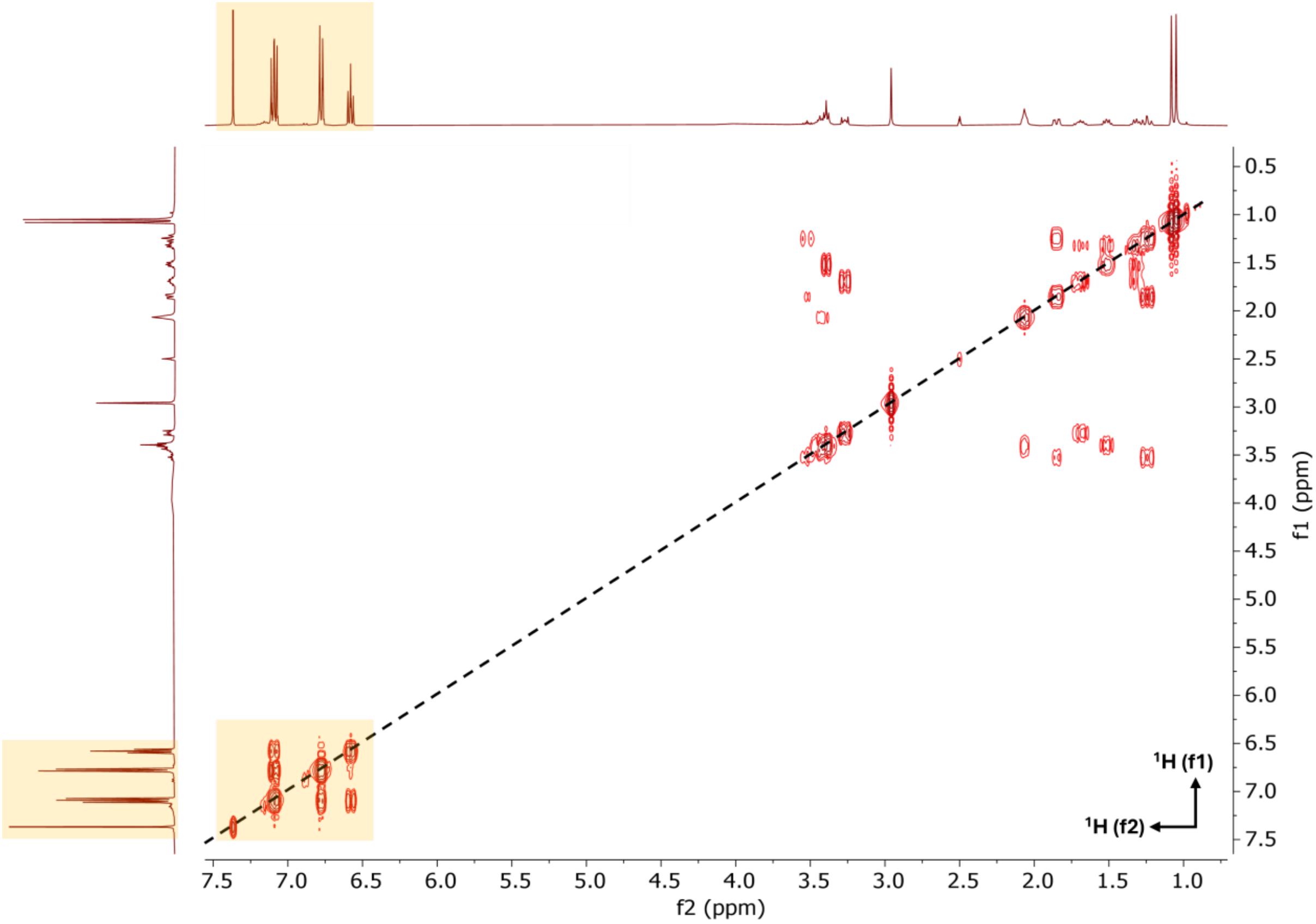

Figure S21 | Full-scale 2D ^{1}H-^{1}H COSY NMR spectrum of [$Pyrr_{TEMPO}$][TFSI] (**2c**), prepared *via* the scaled-up synthetic route, dissolved in DMSO-d_6. The peaks/cross peaks highlighted in yellow correspond to $PhNHNH_2$. Cross peaks on the dotted line indicate self-correlation cross peaks.

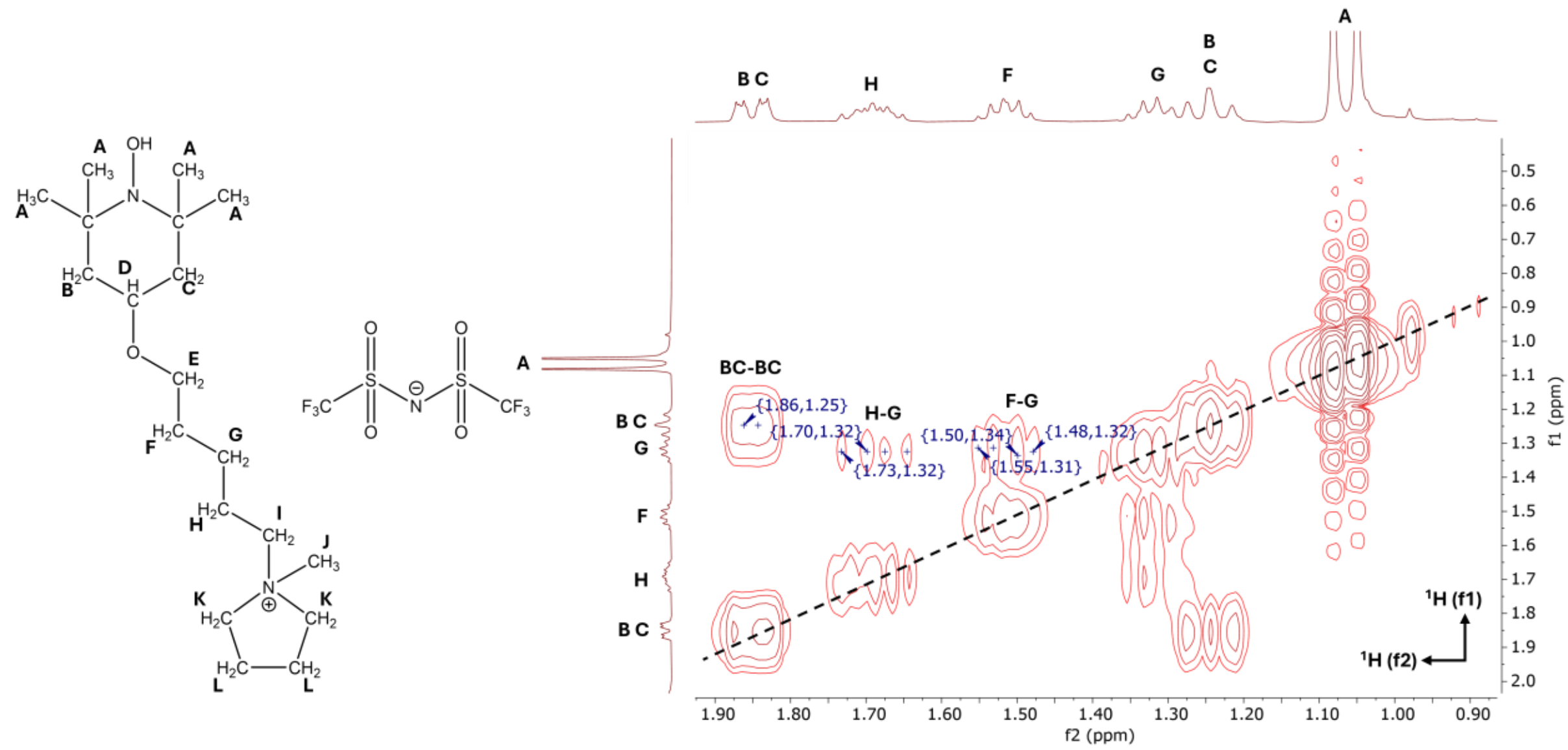

Figure S22 | Zoomed-in region 1 of the 2D ^{1}H-^{1}H COSY NMR spectrum of [Pyrr$_{TEMPO}$][TFSI] (**2c**) dissolved in DMSO-d_6. Cross peaks on the dotted line indicate self-correlation cross peaks.

2D ^{1}H-^{1}H COSY NMR (400 MHz, DMSO-d_6):

f_2 (ppm)	f_1 (ppm)	^{1}H-^{1}H correlation
1.48	1.32	
1.50	1.34	H_F-H_G
1.55	1.31	
1.70	1.32	H_H-H_G
1.73	1.32	
1.86	1.25	H_BH_C-H_BH_C

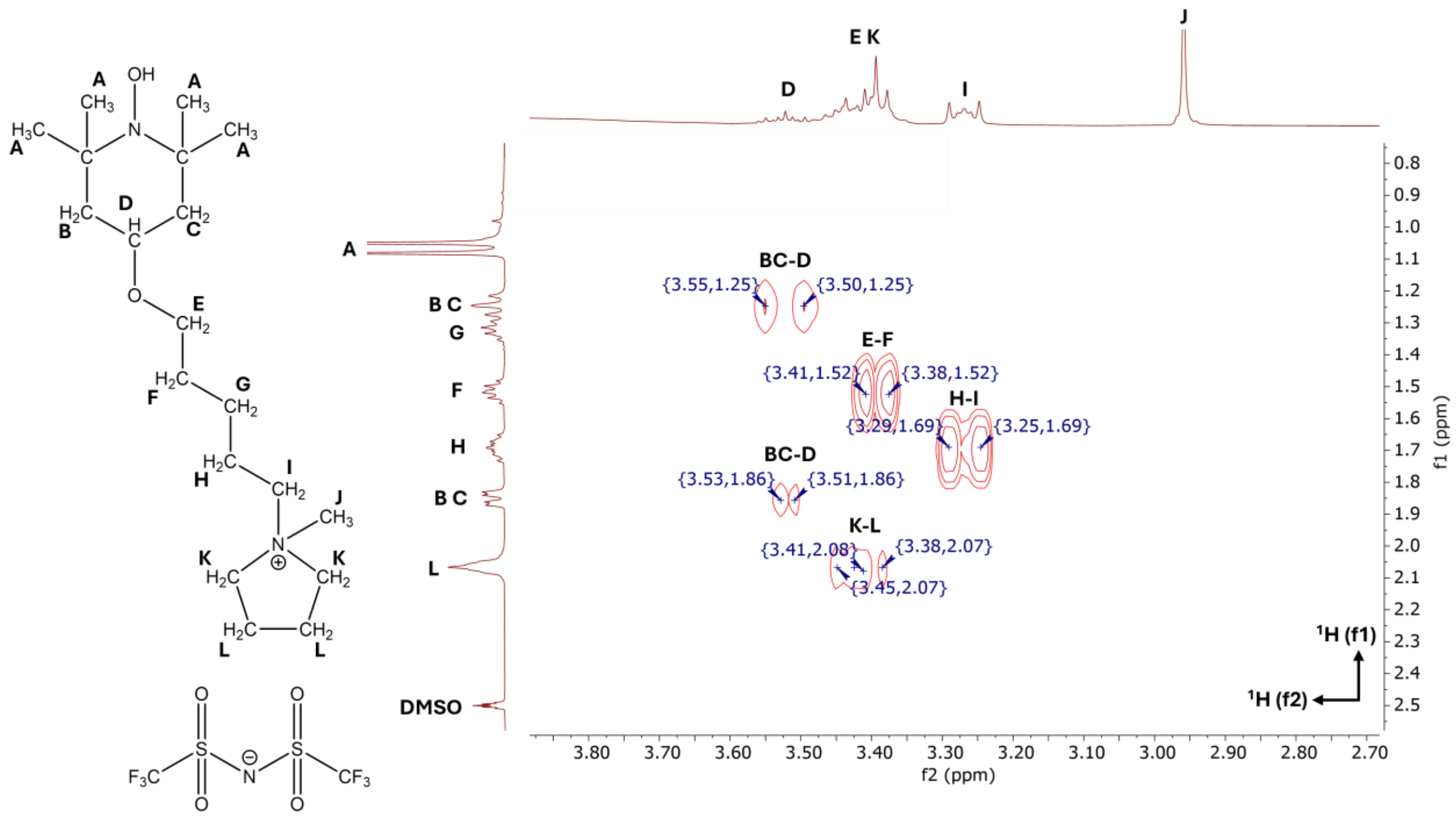

Figure S23 | Zoomed-in region 2 of the 2D ^1H-^1H COSY NMR spectrum of [$Pyrr_{TEMPO}$][TFSI] (**2c**) dissolved in DMSO-d_6. Cross peaks on the dotted line indicate self-correlation cross peaks.

2D ^1H-^1H COSY NMR (400 MHz, DMSO-d_6):

f_2 (ppm)	f_1 (ppm)	^1H-^1H correlation
3.25	1.69	H_H-H_I
3.29	1.69	
3.41	1.52	H_E-H_F
3.38	1.52	
3.45	2.07	H_K-H_L
3.41	2.08	
3.38	2.07	
3.55	1.25	H_BH_C-H_D
3.50	1.25	
3.53	1.86	
3.51	1.86	

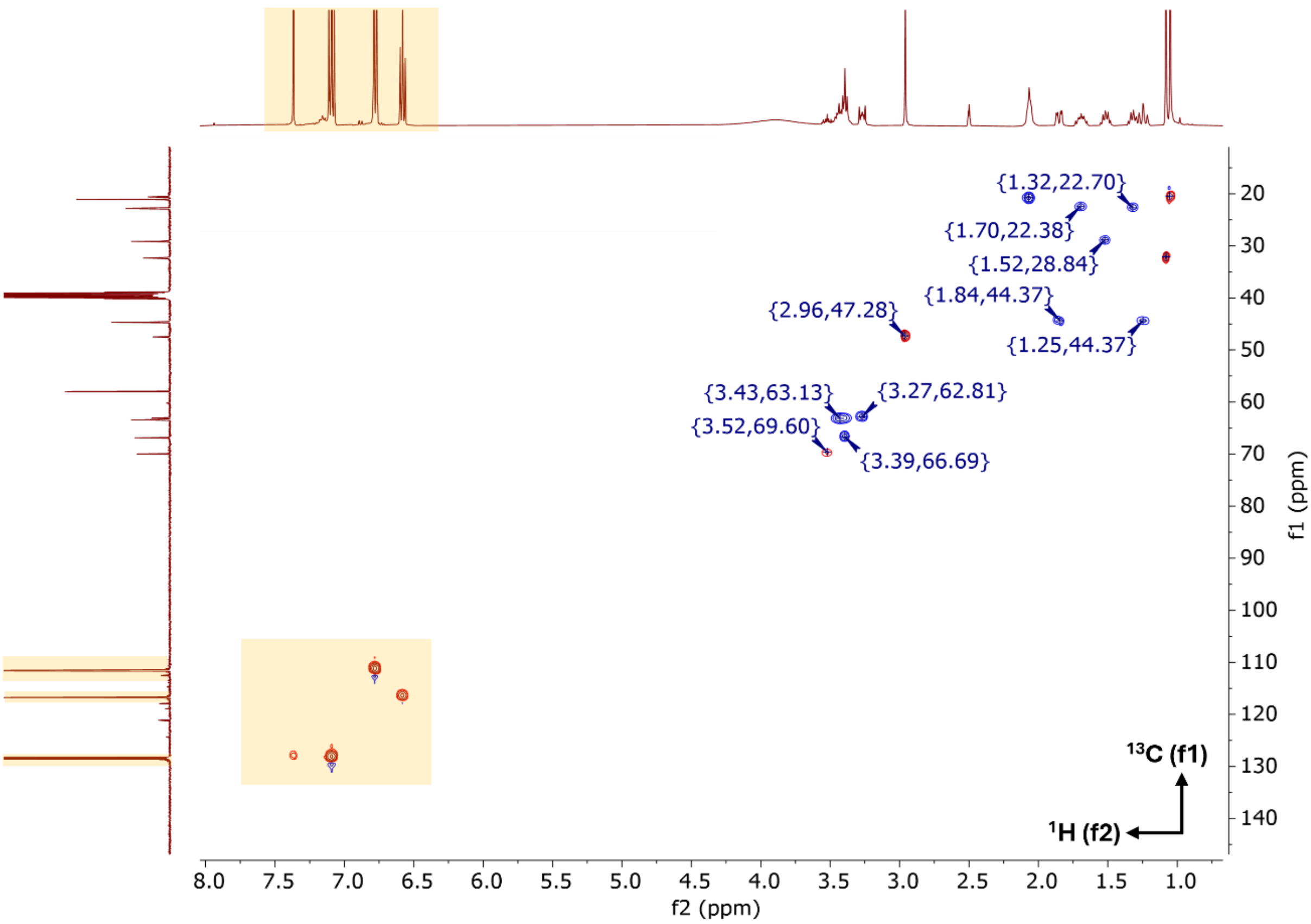

Figure S24 | Full-scale 2D ^{1}H-^{13}C edited HSQC NMR spectrum of [Pyrr$_{TEMPO}$][TFSI] (**2c**), prepared *via* the scaled-up synthetic route, in DMSO-d$_6$. The shaded yellow regions represent peaks/cross peaks related to $PhNHNH_2$. Blue cross peaks indicate that these correlations stem from CH_2 (secondary carbon) units and red cross peaks indicate that the carbon is either tertiary (CH) or primary (CH_3).

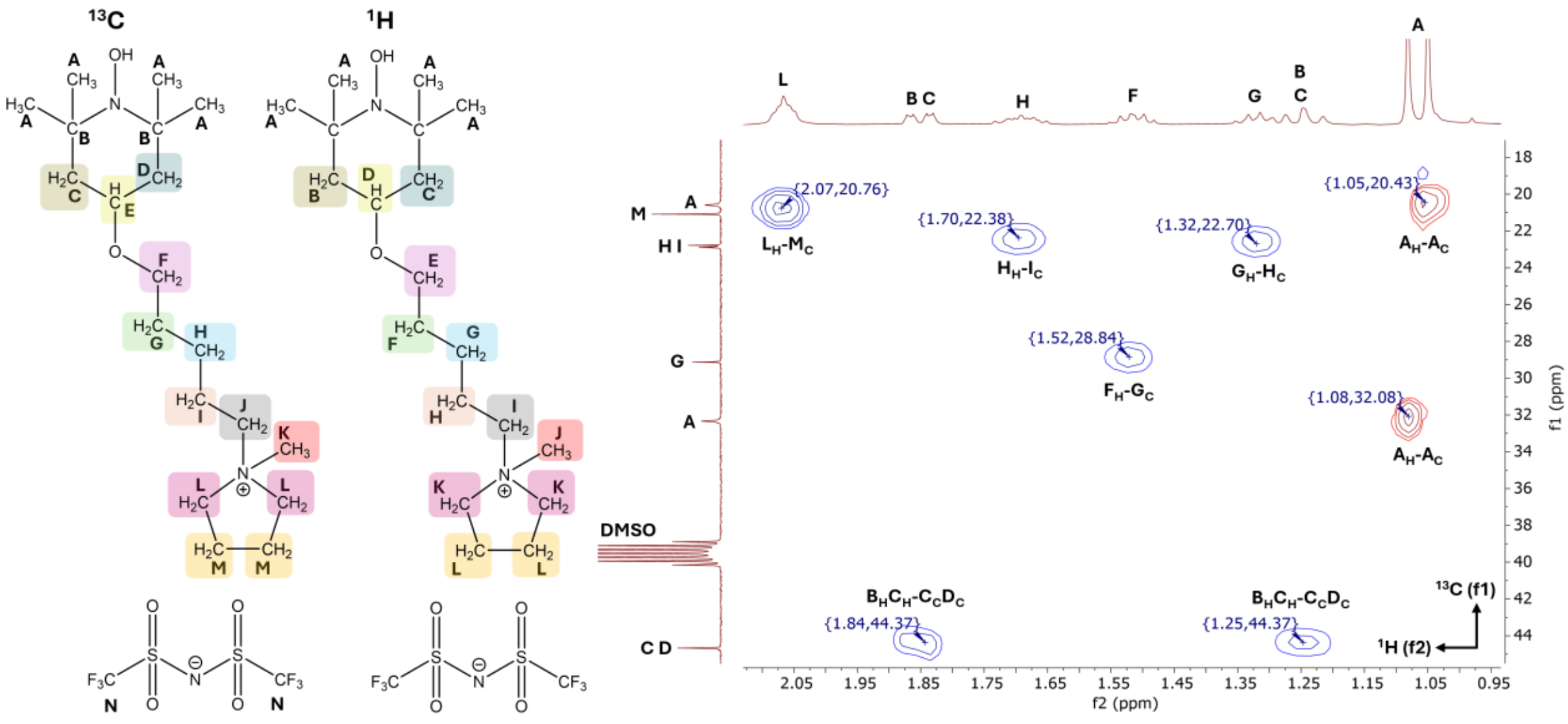

Figure S25 | Zoomed-in region 1 of the 2D ^{1}H-^{13}C edited HSQC NMR spectrum of [$Pyrr_{TEMPO}$][TFSI] (**2c**) in DMSO-d_6. Blue cross peaks indicate that these correlations stem from CH_2 (secondary carbon) units and red cross peaks indicate that the carbon is either tertiary (CH) or primary (CH_3).

2D ^{1}H-^{13}C edited HSQC NMR (400 MHz (^{1}H), 100 MHz (^{13}C), DMSO-d_6):

f_2, ^{1}H (ppm)	f_1, ^{13}C (ppm)	^{1}H-^{13}C correlation
1.05	20.43	A_H-A_C
1.08	32.08	
1.32	22.70	G_H-H_C
1.52	28.84	F_H-G_C
1.70	22.38	H_H-I_C
1.25	44.37	B_HC_H-C_CD_C
1.84	44.37	
2.07	20.76	L_H-M_C

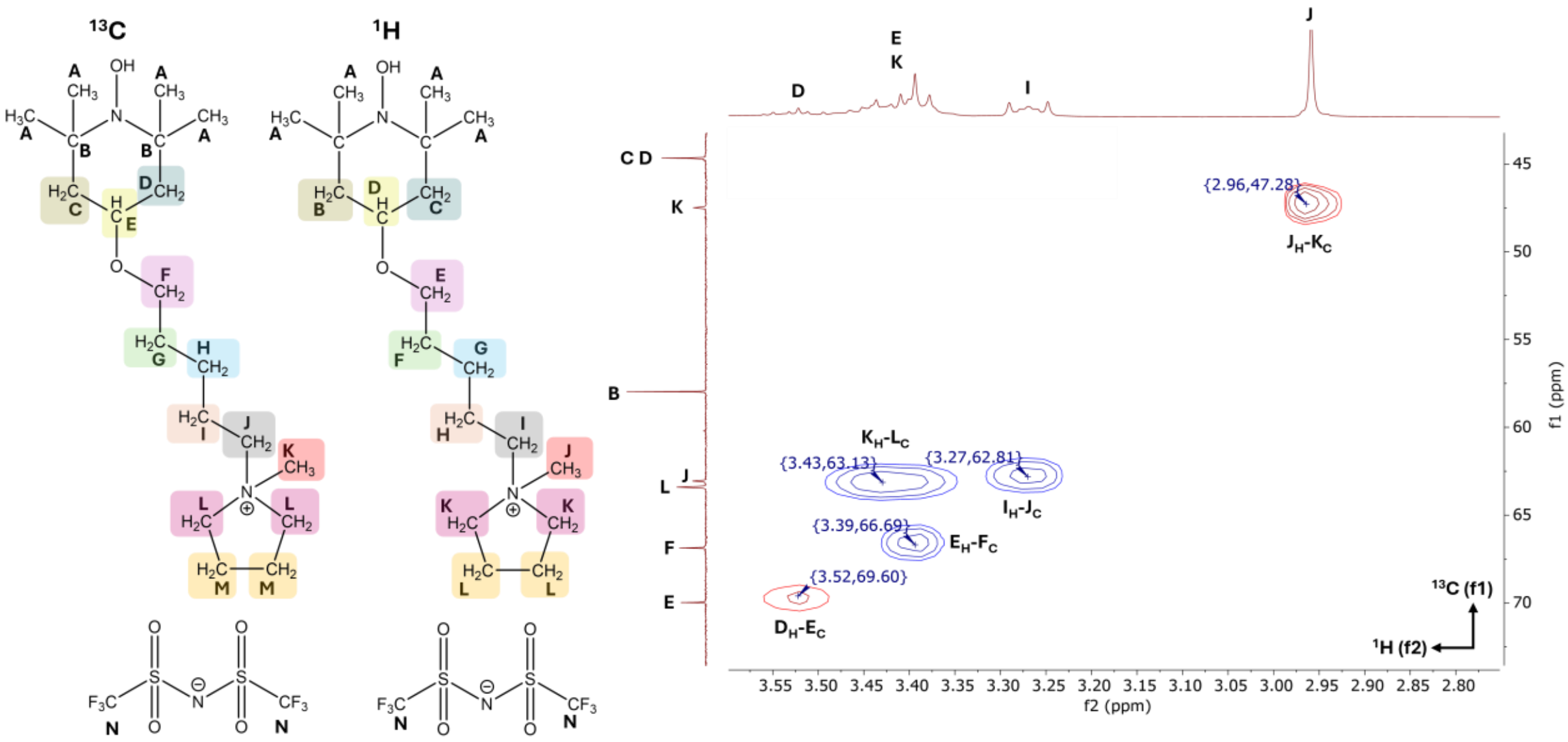

Figure S26 | Zoomed-in region 2 of the 2D ^{1}H-^{13}C edited HSQC NMR spectrum of [Pyrr$_{TEMPO}$][TFSI] (**2c**) in DMSO-d$_6$. Blue cross peaks indicate that these correlations stem from CH_2 (secondary carbon) units and red cross peaks indicate that the carbon is either tertiary (CH) or primary (CH_3).

2D ^{1}H-^{13}C edited HSQC NMR (400 MHz (^{1}H), 100 MHz (^{13}C), DMSO-d$_6$):

**f_2, ^{1}H (ppm)**	**f_1, ^{13}C (ppm)**	**^{1}H-^{13}C correlation**
2.96	47.28	J_H-K_C
3.27	62.81	I_H-J_C
3.39	66.69	E_H-F_C
3.43	63.13	K_H-L_C
3.52	69.60	D_H-E_C

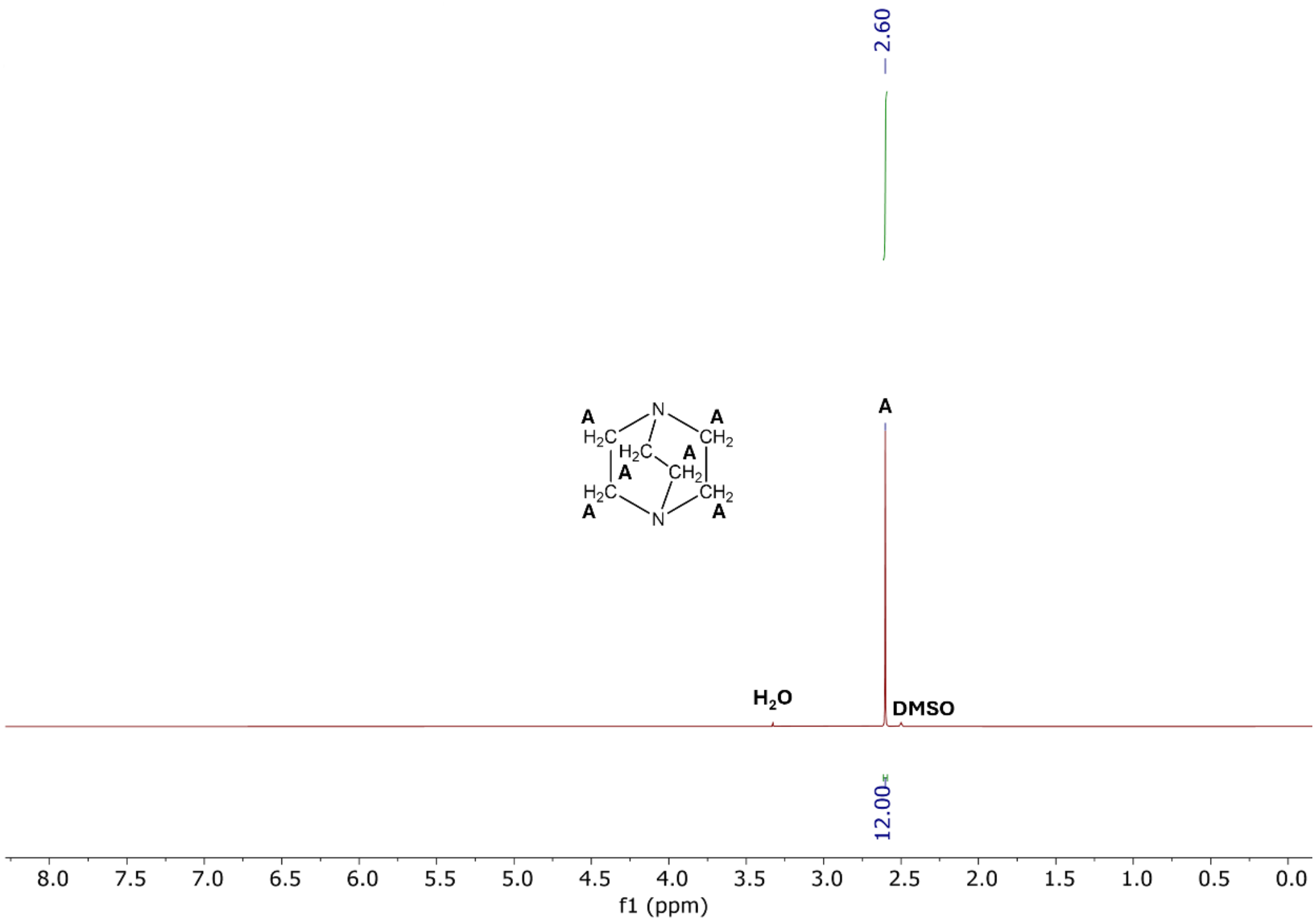

Figure S27 | ^{1}H NMR of DABCO starting material in DMSO-d_6.

^{1}H NMR (400 MHz, DMSO-d_6): δ 2.60 (s, 12H, H_A)

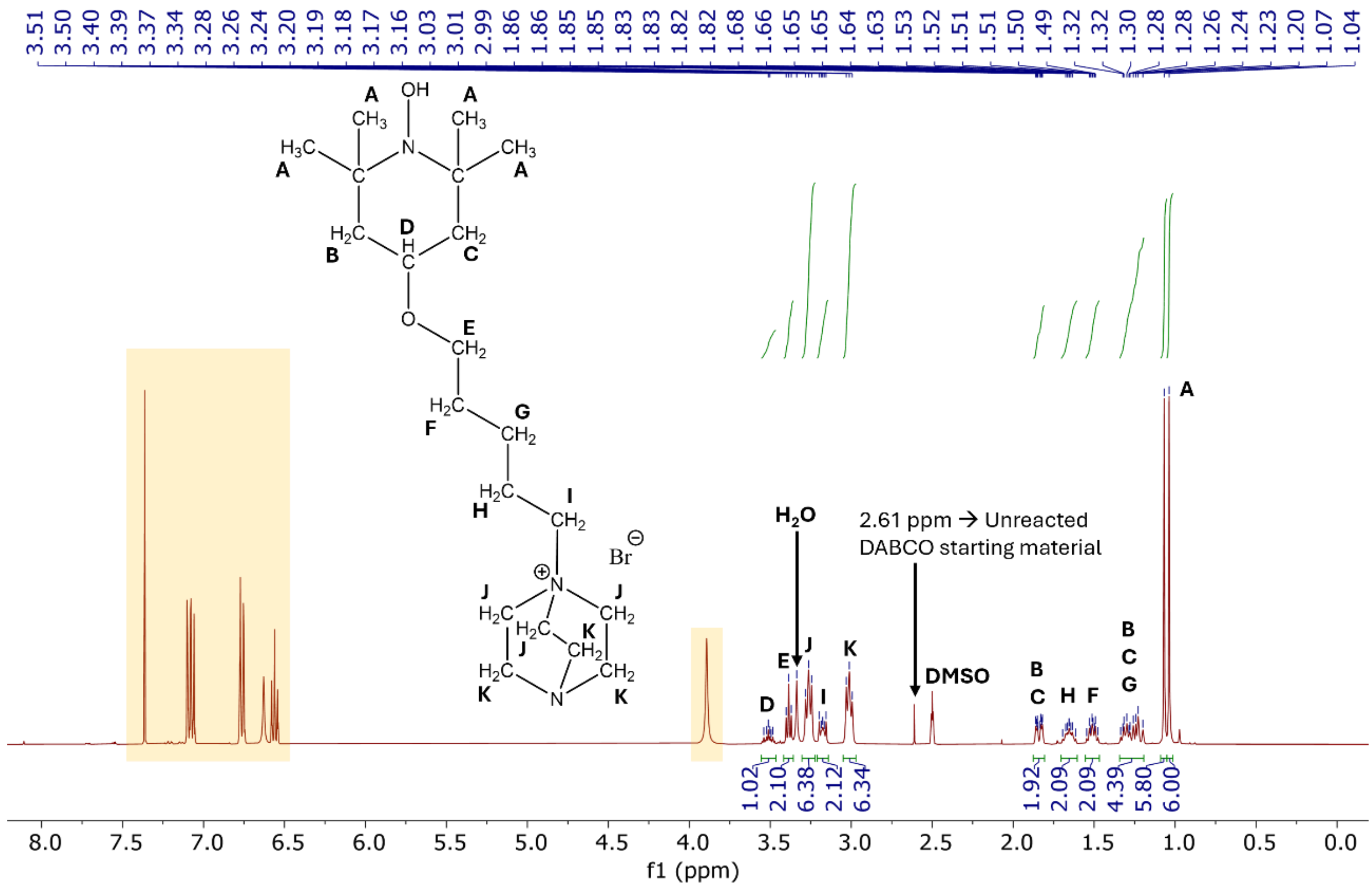

Figure S28 | ^{1}H NMR of [D$_{TEMPO}$][Br] (**3b**) in DMSO-d$_6$. Peaks shaded in yellow correspond to $PhNHNH_2$.

^{1}H NMR (400 MHz, DMSO-d$_6$): δ 1.04 (s, 6H, H$_A$), 1.07 (s, 6H, H$_A$), 1.20-1.34 (m, 4H, H$_B$, H$_C$, H$_G$), 1.48-1.55 (m, 2H, H$_F$), 1.61-1.69 (m, 2H, H$_H$), 1.82-1.86 (m, 2H, H$_B$, H$_C$), 3.01 (t, 6H, H$_K$), 3.16-3.20 (m, 2H, H$_I$), 3.26 (t, 6H, H$_J$), 3.39 (t, 2H, H$_E$), 3.48-3.54 (m, 1H, H$_D$).

The unreacted DABCO present in [D$_{TEMPO}$][Br] was removed in the work-up following the anion metathesis reaction to give [D$_{TEMPO}$][TFSI]. [D$_{TEMPO}$][TFSI] was purified by thoroughly washing the organic phase with water, which removed DABCO as well, as it is preferentially solubilised in the aqueous phase. This is why the peak at 2.61 ppm corresponding to DABCO is absent in the ^{1}H NMR of [D$_{TEMPO}$][TFSI] (**Figure S29**).

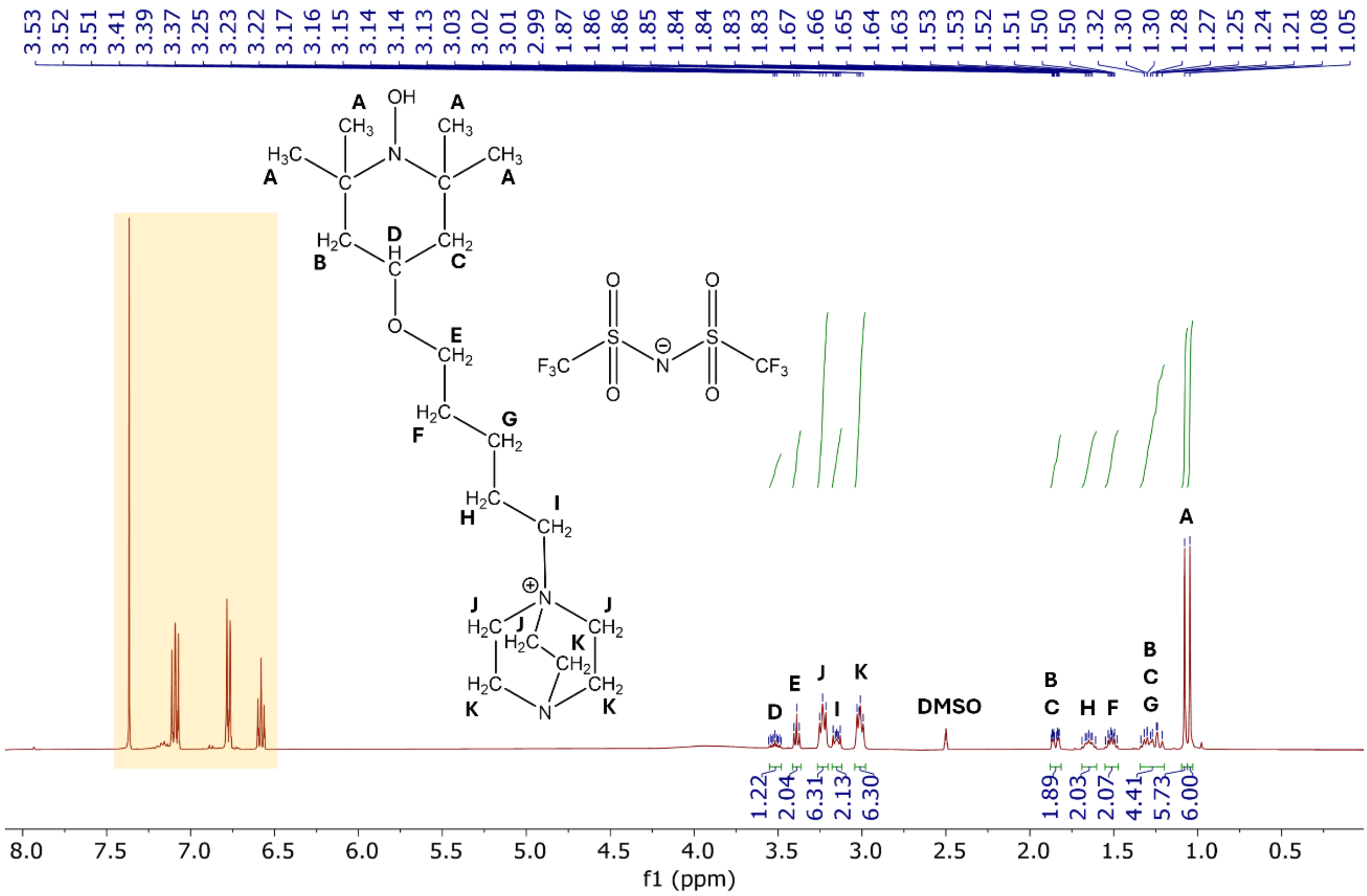

Figure S29 | ^{1}H NMR of [D_{TEMPO}][TFSI] (**3c**) in DMSO-d_6. Peaks shaded in yellow correspond to $PhNHNH_2$.

^{1}H NMR (400 MHz, DMSO-d_6): δ 1.05 (s, 6H, H_A), 1.08 (s, 6H, H_A), 1.21-1.34 (m, 4H, H_B, H_C, H_G), 1.48-1.55 (m, 2H, H_F), 1.61-1.69 (m, 2H, H_H), 1.83-1.87 (m, 2H, H_B, H_C), 3.01 (t, 6H, H_K), 3.13-3.17 (m, 2H, H_I), 3.23 (t, 6H, H_J), 3.39 (t, 2H, H_E), 3.48-3.56 (m, 1H, H_D).

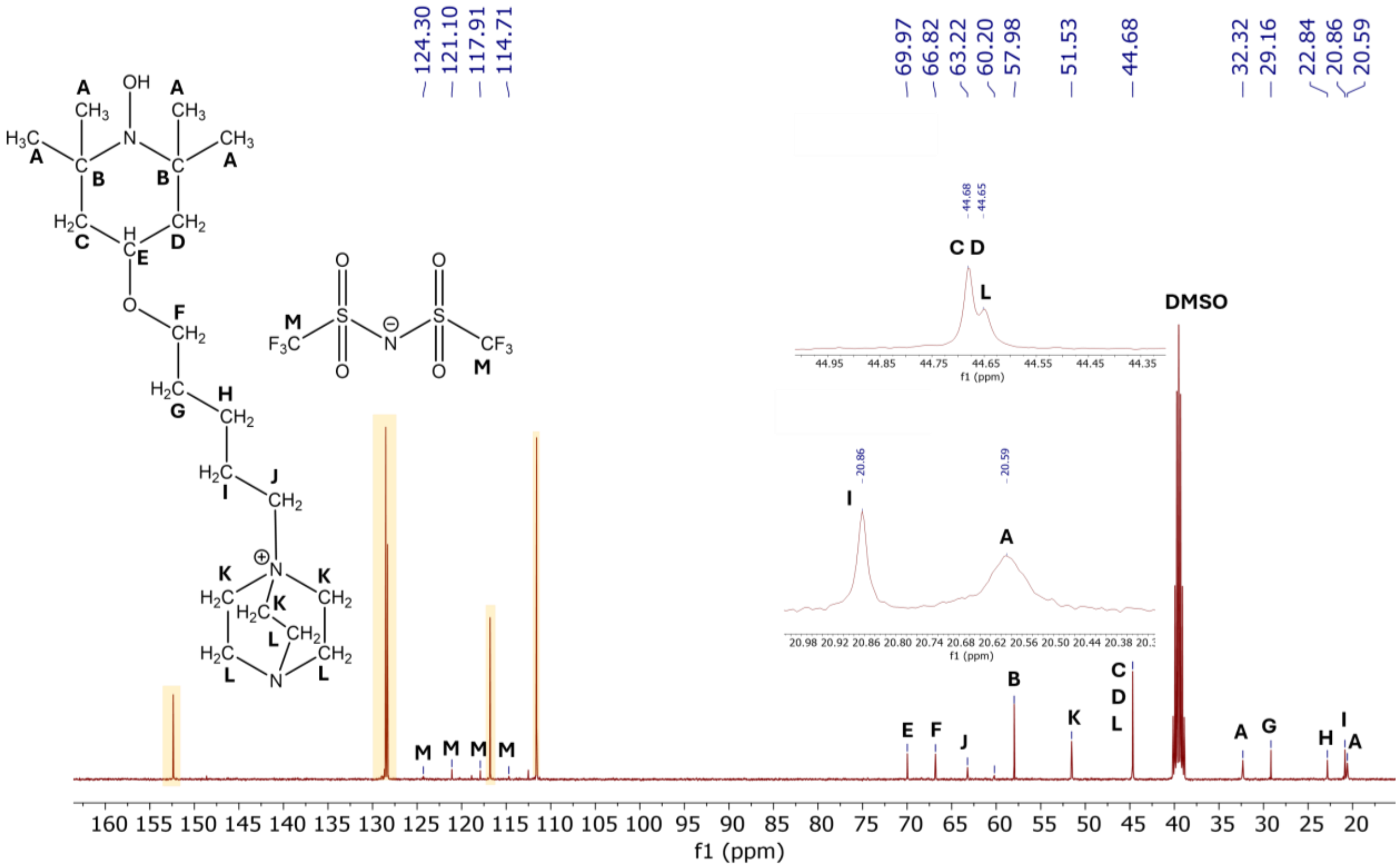

Figure S30 | ^{13}C {^{1}H} NMR of [D$_{TEMPO}$][TFSI] (**3c**) in DMSO-d$_6$. Peaks shaded in yellow correspond to PhNHNH$_2$.

13C {^{1}H} NMR (100 MHz, DMSO-d$_6$): δ 20.59 (C$_A$), 20.86 (C$_I$), 22.84 (C$_H$), 29.16 (C$_G$), 32.32 (C$_A$), 44.65 (C$_L$), 44.68 (C$_C$, C$_D$), 51.53 (C$_K$), 57.98 (C$_B$), 63.22 (C$_J$), 66.82 (C$_F$), 69.97 (C$_E$), 119.5 (q, C$_M$, *J* = 322 Hz).

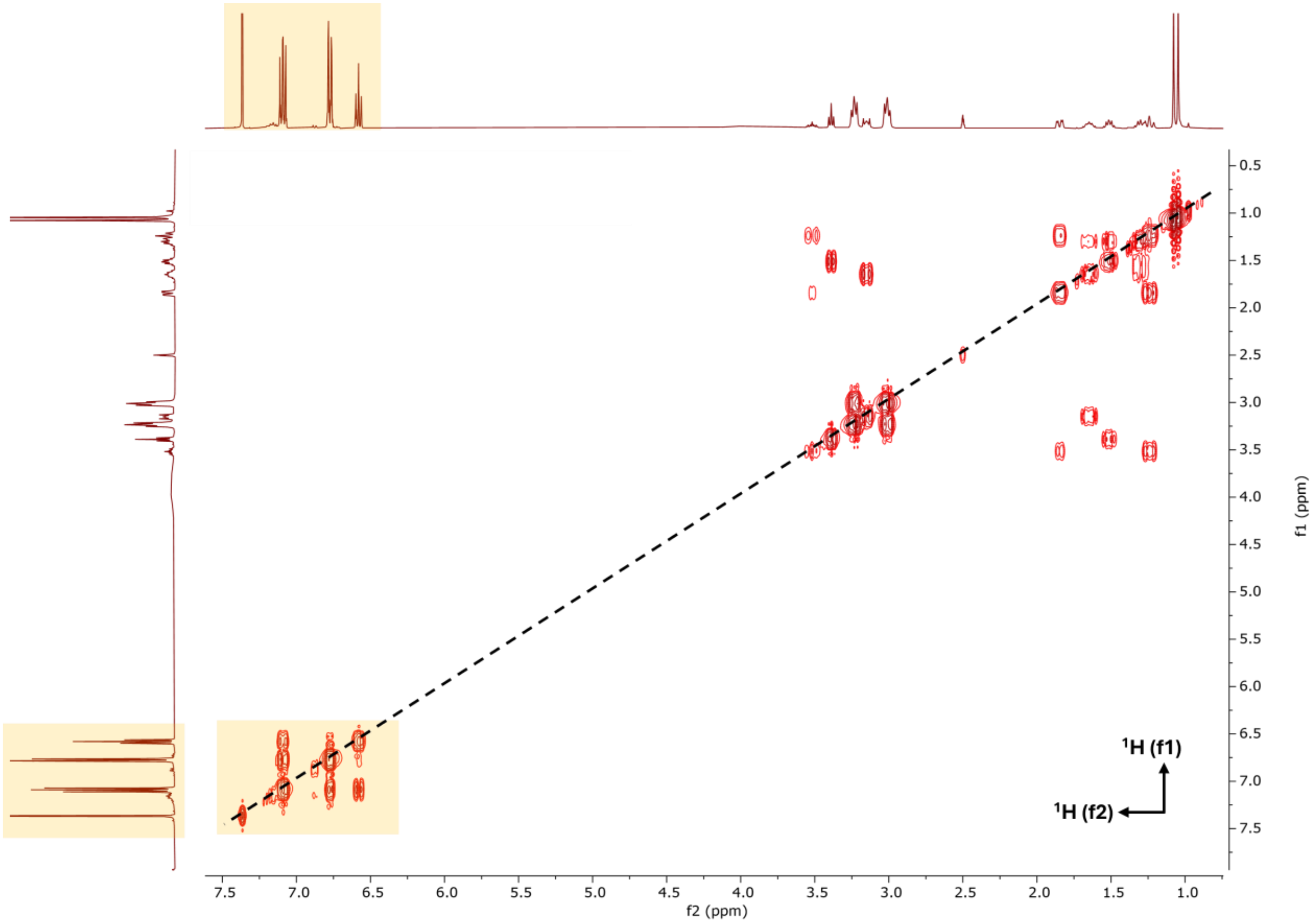

Figure S31 | Full-scale 2D ^{1}H-^{1}H COSY NMR spectrum of [D$_{TEMPO}$][TFSI] (**3c**) dissolved in DMSO-d$_6$. The peaks/cross peaks highlighted in yellow correspond to PhNHNH$_2$. Cross peaks on the dotted line indicate self-correlation cross peaks.

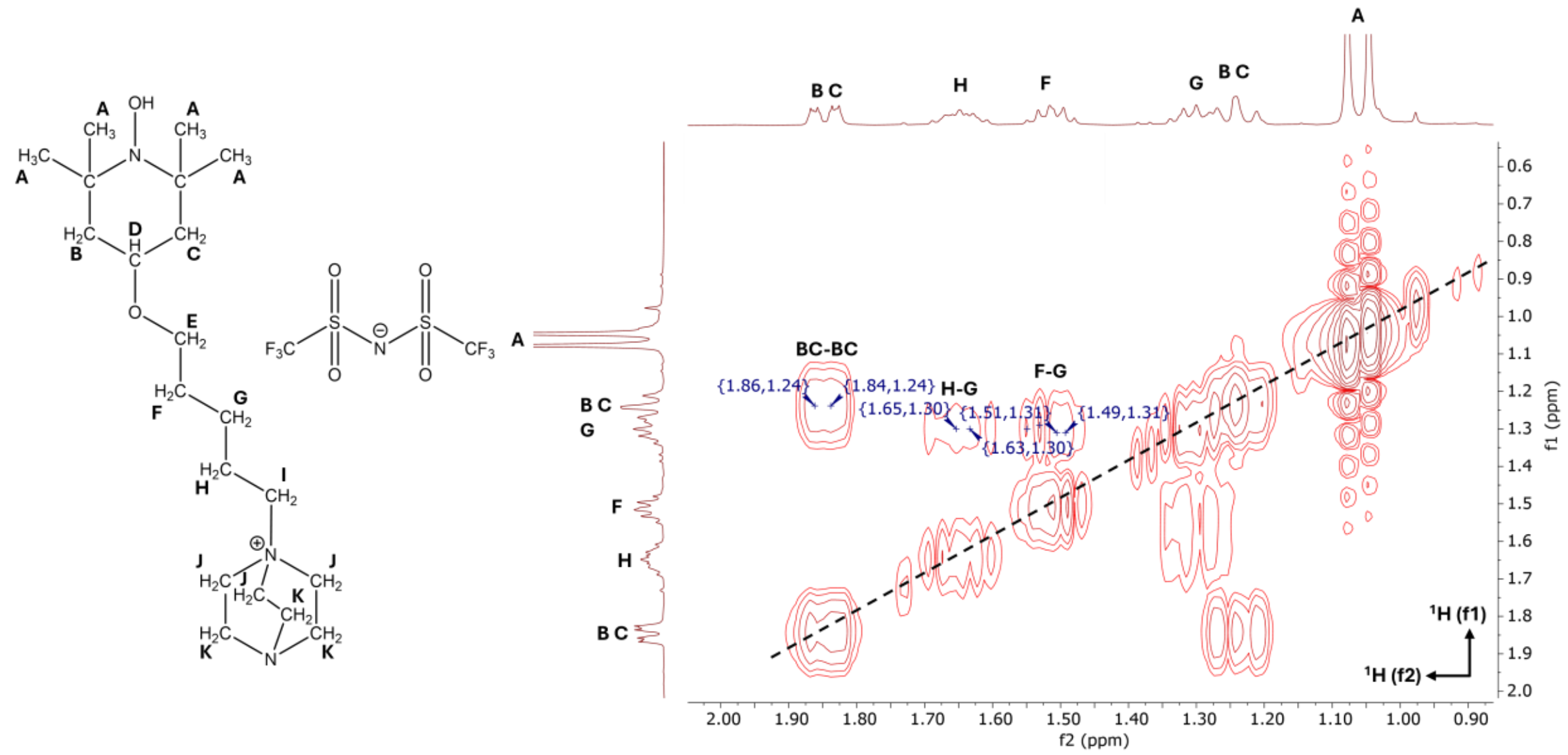

Figure S32 | Zoomed-in region 1 of the 2D ^{1}H-^{1}H COSY NMR spectrum of [D$_{TEMPO}$][TFSI] (**3c**) dissolved in DMSO-d$_6$. Cross peaks on the dotted line indicate self-correlation cross peaks.

2D ^{1}H-^{1}H COSY NMR (400 MHz, DMSO-d$_6$):

**f_2 (ppm)**	**f_1 (ppm)**	**^{1}H-^{1}H correlation**
1.49	1.31	H_F-H_G
1.51	1.31	
1.63	1.30	H_H-H_G
1.65	1.30	
1.84	1.24	H_BH_C-H_BH_C
1.86	1.24	

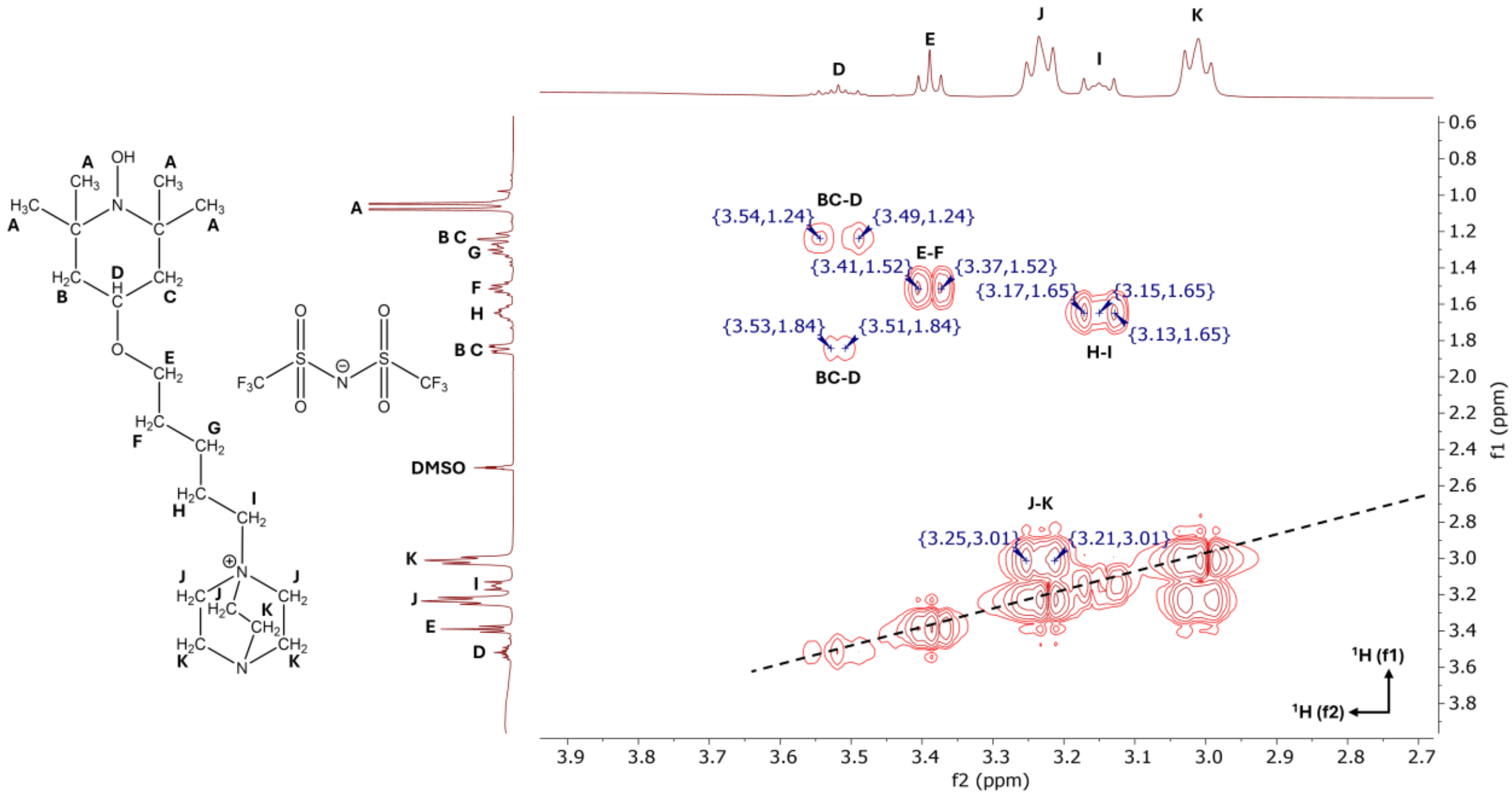

Figure S33 | Zoomed-in region 2 of the 2D ^{1}H-^{1}H COSY NMR spectrum of [D_{TEMPO}][TFSI] (**3c**) dissolved in DMSO-d_6. Cross peaks on the dotted line indicate self-correlation cross peaks.

2D ^{1}H-^{1}H COSY NMR (400 MHz, DMSO-d_6):

f_2 (ppm)	f_1 (ppm)	^{1}H-^{1}H correlation
3.37	1.52	H_E-H_F
3.41	1.52	
3.54	1.24	H_BH_C-H_D
3.49	1.24	
3.53	1.84	
3.51	1.84	
3.17	1.65	H_H-H_I
3.15	1.65	
3.13	1.65	
3.25	3.01	H_J-H_K
3.21	3.01	

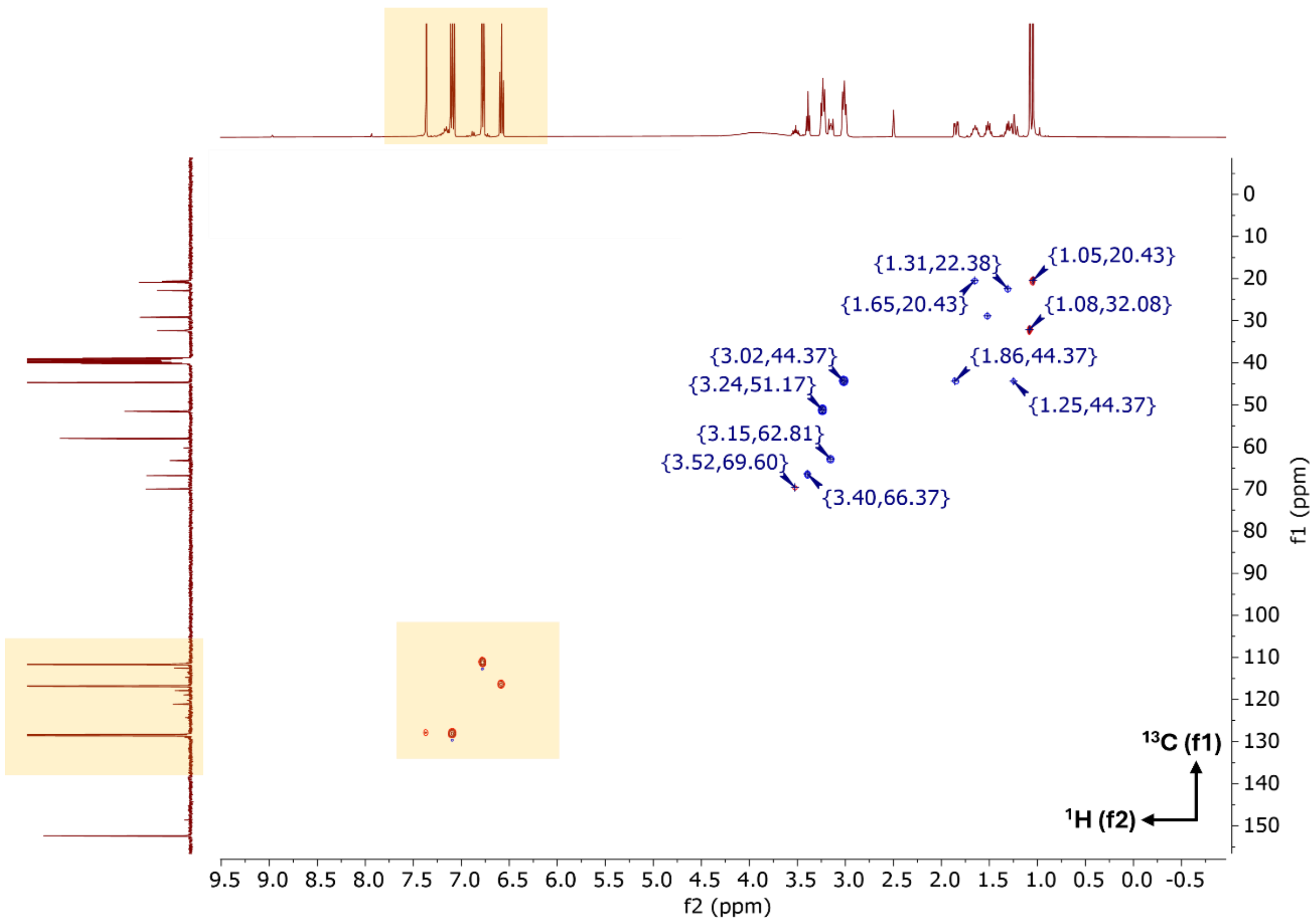

Figure S34 | Full-scale 2D ^{1}H-^{13}C edited HSQC NMR spectrum of [D_{TEMPO}][TFSI] (**3c**) in DMSO-d_6. The shaded yellow regions represent peaks/cross peaks related to $PhNHNH_2$. Blue cross peaks indicate that these correlations stem from CH_2 (secondary carbon) units and red cross peaks indicate that the carbon is either tertiary (CH) or primary (CH_3).

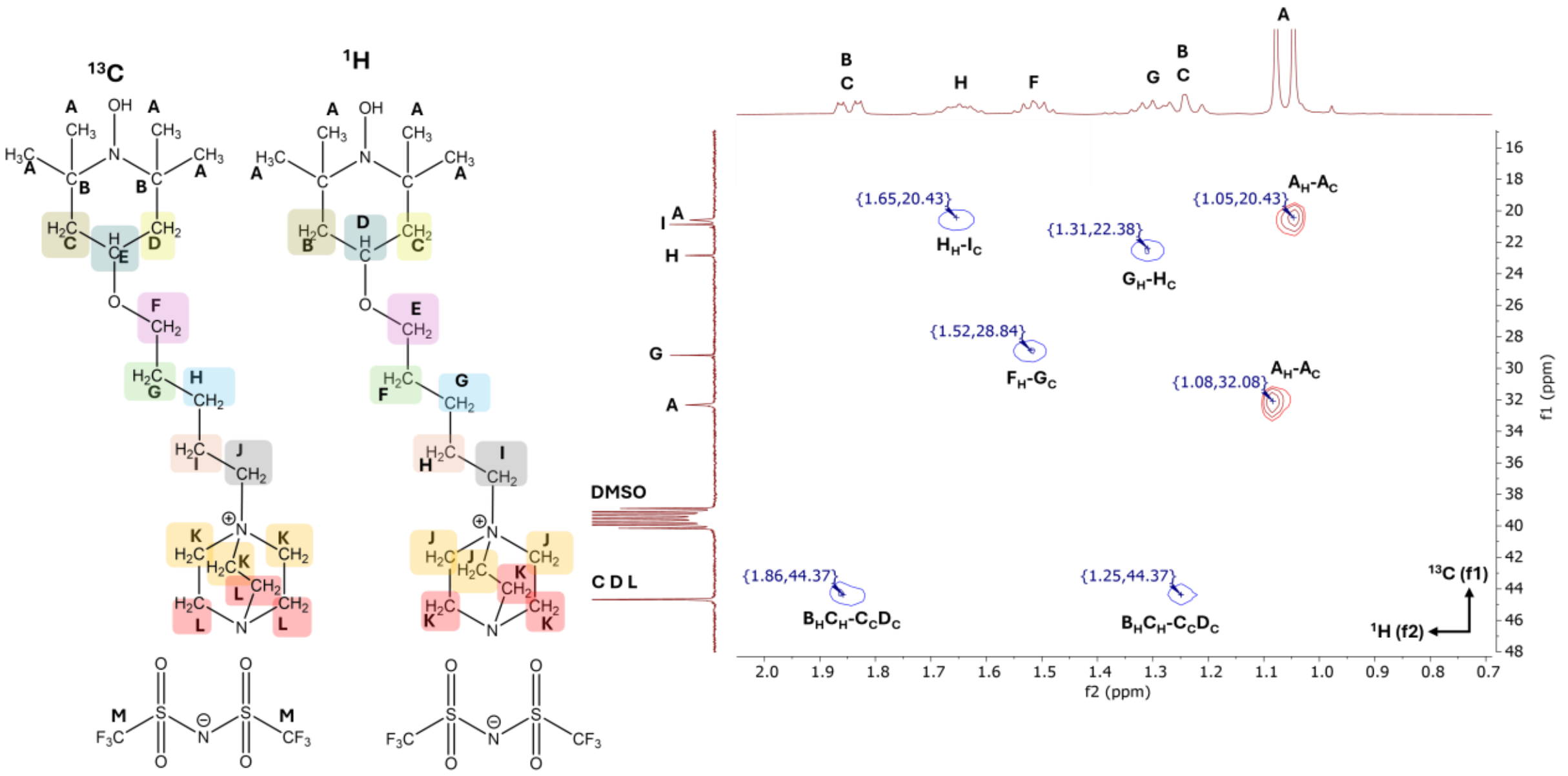

Figure S35 | Zoomed-in region 1 of the 2D ^{1}H-^{13}C edited HSQC NMR spectrum of [D_{TEMPO}][TFSI] (**3c**) in DMSO-d_6. Blue cross peaks indicate that these correlations stem from CH_2 (secondary carbon) units and red cross peaks indicate that the carbon is either tertiary (CH) or primary (CH_3).

2D ^{1}H-^{13}C edited HSQC NMR (400 MHz (^{1}H), 100 MHz (^{13}C), DMSO-d_6):

f_2, ^{1}H (ppm)	f_1, ^{13}C (ppm)	^{1}H-^{13}C correlation
1.05	20.43	A_H-A_C
1.08	32.08	
1.31	22.38	G_H-H_C
1.52	28.84	F_H-G_C
1.65	20.43	H_H-I_C
1.25	44.37	B_HC_H-C_CD_C
1.86	44.37	

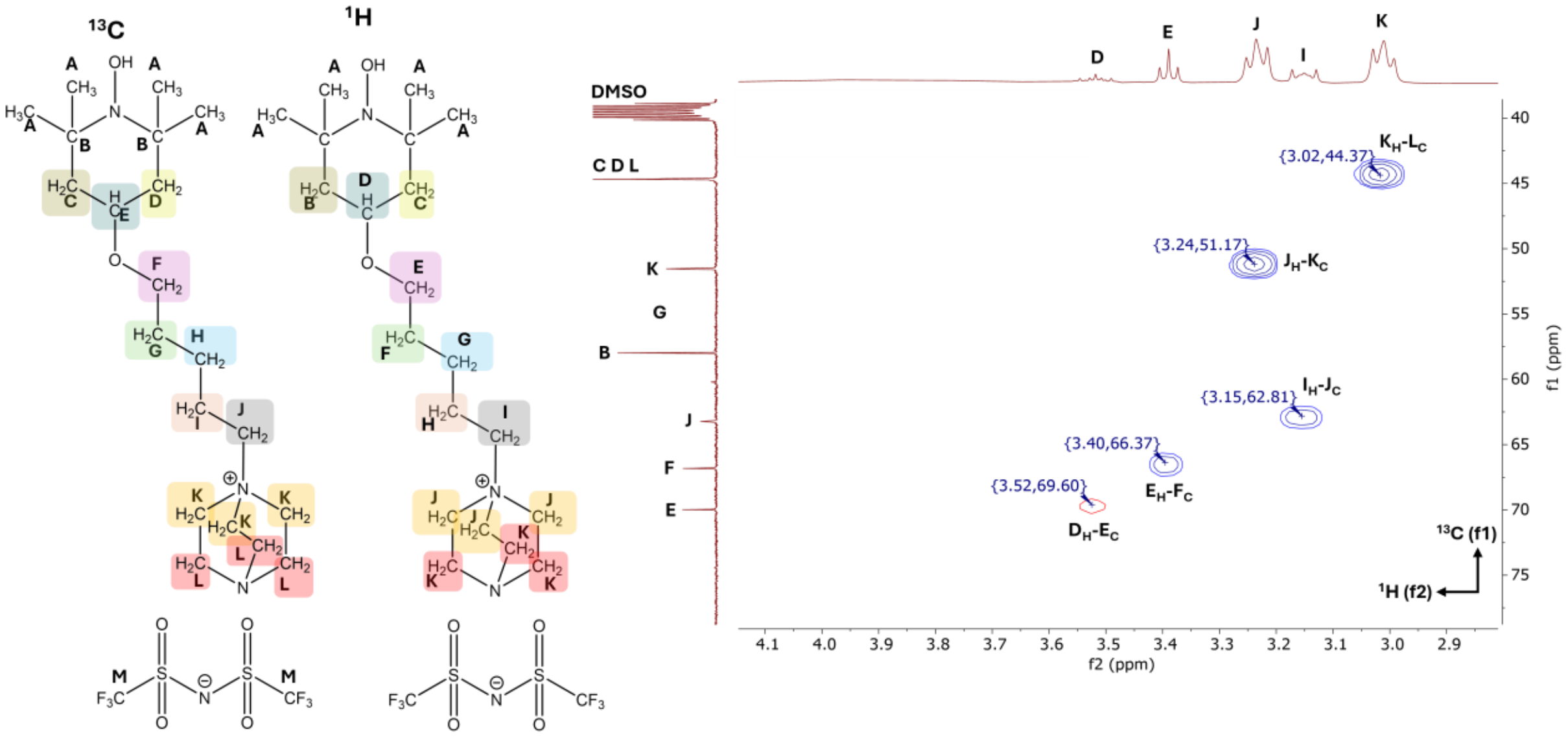

Figure S36 | Zoomed-in region 2 of the 2D ^{1}H-^{13}C HSQC NMR spectrum of [D$_{TEMPO}$][TFSI] (**3c**) in DMSO-d$_6$. Blue cross peaks indicate that these correlations stem from CH_2 (secondary carbon) units and red cross peaks indicate that the carbon is either tertiary (CH) or primary (CH_3).

2D ^{1}H-^{13}C edited HSQC NMR (400 MHz (^{1}H), 100 MHz (^{13}C), DMSO-d$_6$):

**f$_2$, ^{1}H (ppm)**	**f$_1$, ^{13}C (ppm)**	**^{1}H-^{13}C correlation**
3.40	66.37	E_H-F_C
3.52	69.60	D_H-E_C
3.15	62.81	I_H-J_C
3.24	51.17	J_H-K_C
3.02	44.37	K_H-L_C

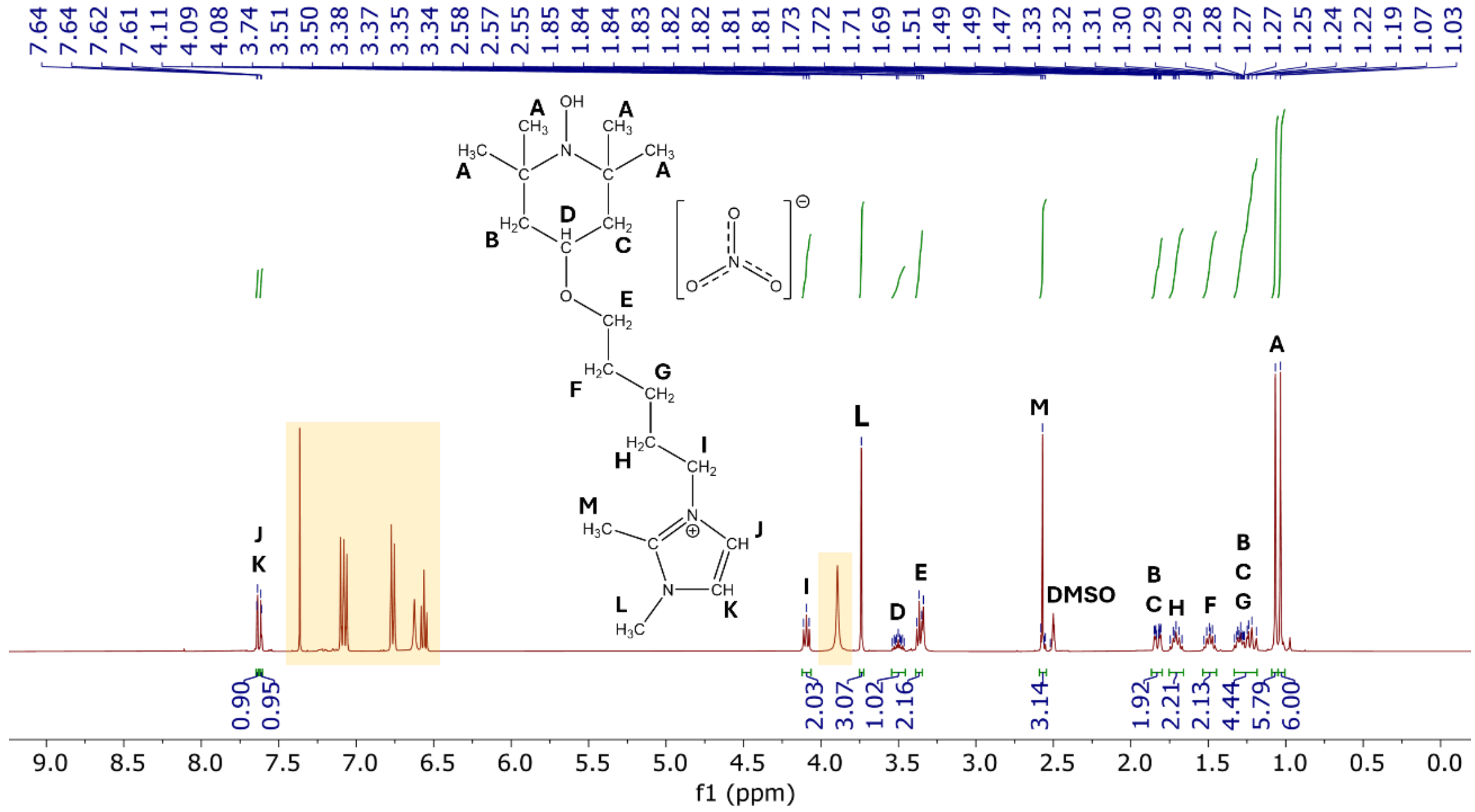

Figure S37 | ^{1}H NMR of $[Im_{TEMPO}][NO_3]$ (**4c**) in DMSO-d_6. Peaks shaded in yellow correspond to $PhNHNH_2$.

^{1}H NMR (400 MHz, DMSO-d_6): δ 1.03 (s, 6H, H_A), 1.07 (s, 6H, H_A), 1.19-1.33 (m, 4H, H_B, H_C, H_G), 1.46-1.53 (m, 2H, H_F), 1.67-1.75 (m, 2H, H_H), 1.81-1.85 (m, 2H, H_B, H_C), 2.57 (s, 3H, H_M), 3.37 (t, 2H, H_E), 3.46-3.54 (m, 1H, H_D), 3.74 (s, 3H, H_L), 4.09 (t, 2H, H_I), 7.61 (d, 1H, H_J/H_K), 7.64 (d, 1H, H_J/H_K).

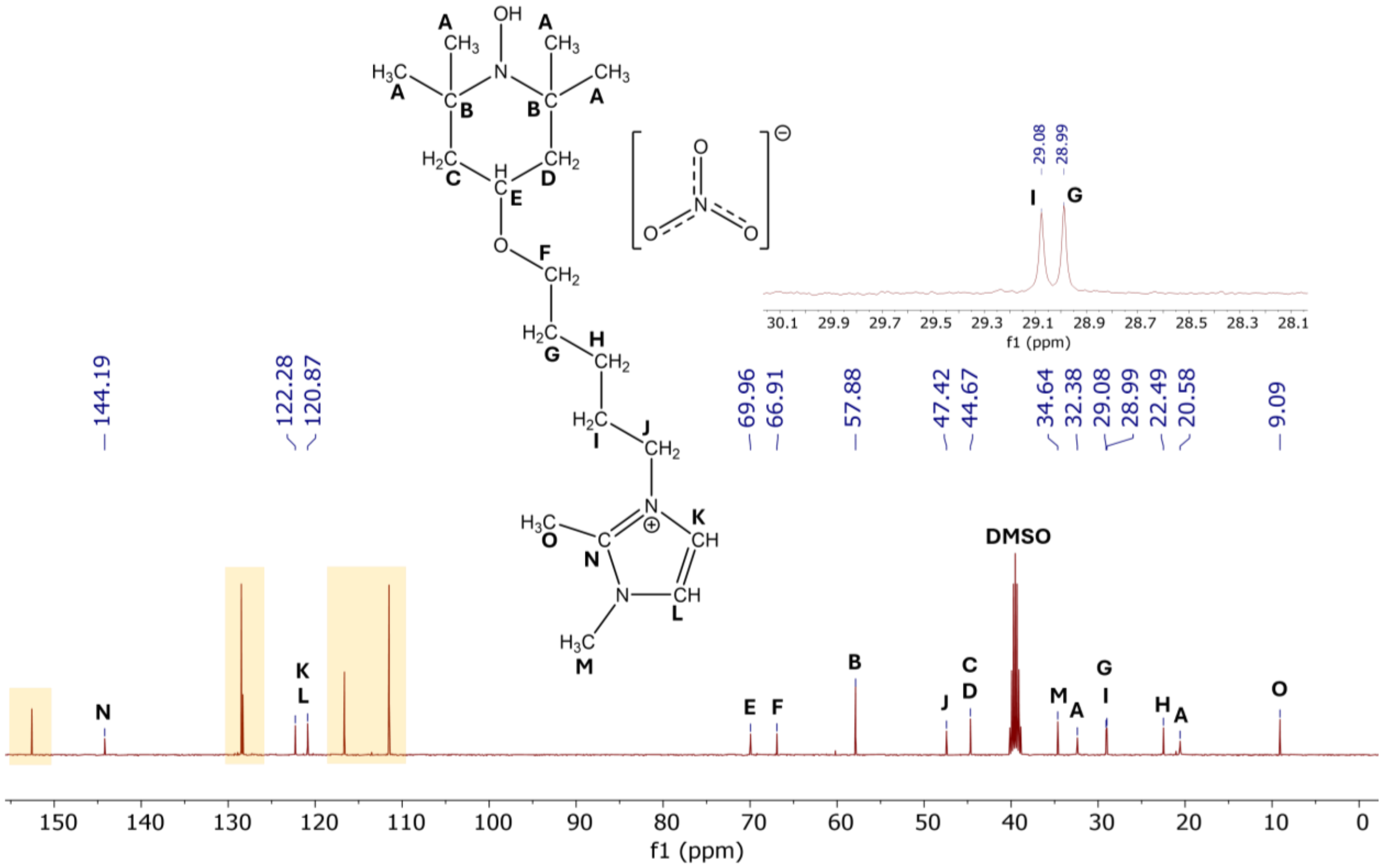

Figure S38 | ^{13}C {^{1}H} NMR of [Im$_{TEMPO}$][NO_3] (**4c**) in DMSO-d$_6$. Peaks shaded in yellow correspond to $PhNHNH_2$.

^{13}C {^{1}H} NMR (100 MHz, DMSO-d$_6$): δ 9.09 (C_O), 20.58 (C_A), 22.49 (C_H), 28.99 (C_G), 29.08 (C_I), 32.38 (C_A), 34.64 (C_M), 44.67 (C_C, C_D), 47.42 (C_J), 57.88 (C_B), 66.91 (C_F), 69.96 (C_E), 120.87 (C_K/C_L), 122.28 (C_K/C_L), 144.19 (C_N).

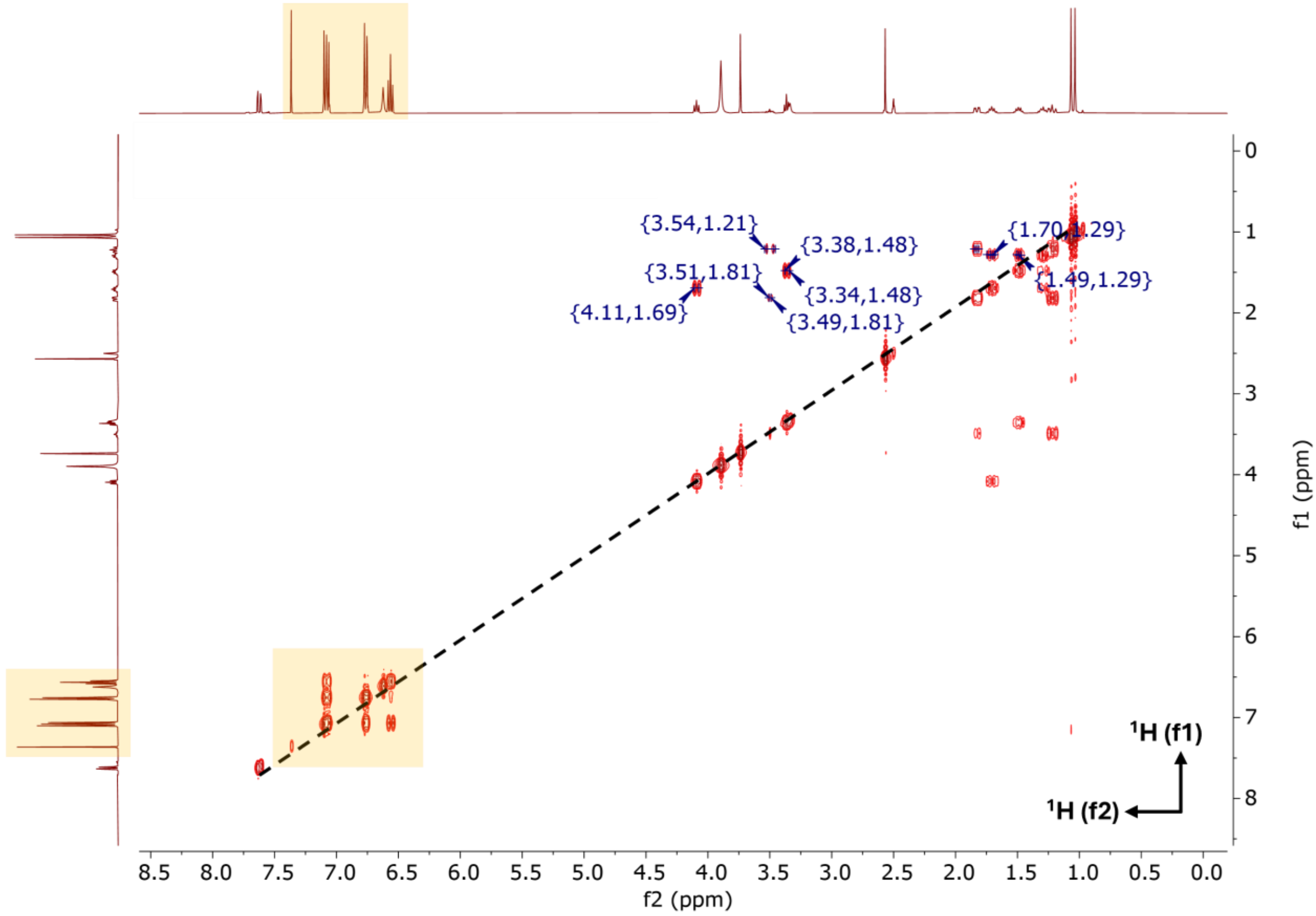

Figure S39 | Full-scale 2D ¹H-¹H COSY NMR spectrum of [Im_{TEMPO}][NO_3] (**4c**) dissolved in DMSO-d_6. The peaks/cross peaks highlighted in yellow correspond to $PhNHNH_2$. Cross peaks on the dotted line indicate self-correlation cross peaks.

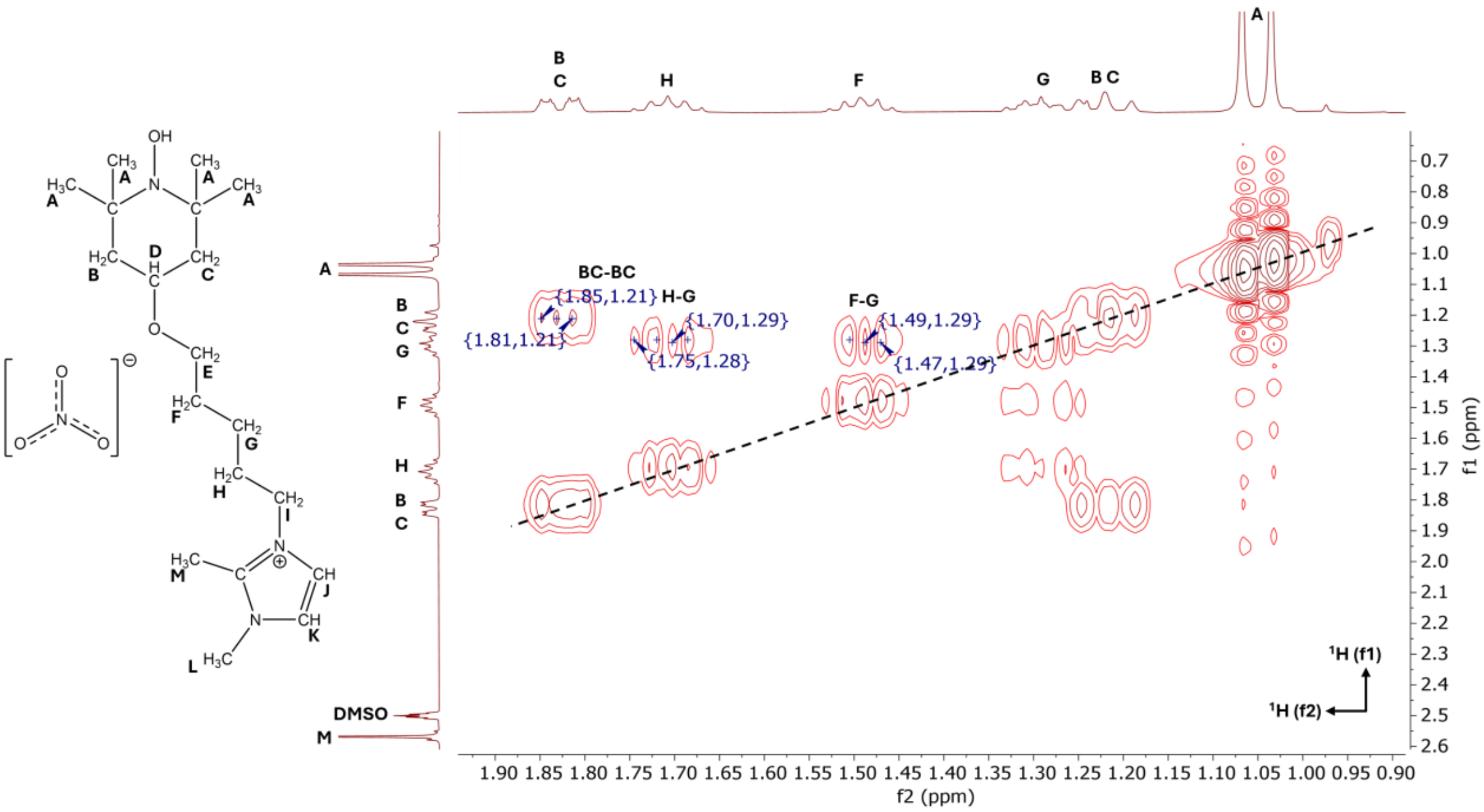

Figure S40 | Zoomed-in region 1 of the 2D ^1H-^1H COSY NMR spectrum of [Im_{TEMPO}][NO_3] (**4c**) dissolved in DMSO-d_6. Cross peaks on the dotted line indicate self-correlation cross peaks.

2D ^1H-^1H COSY NMR (400 MHz, DMSO-d_6):

**f_2 (ppm)**	**f_1 (ppm)**	**^1H-^1H correlation**
1.49	1.29	H_F-H_G
1.47	1.29	
1.70	1.29	H_H-H_G
1.75	1.28	
1.85	1.21	H_BH_C-H_BH_C
1.81	1.21	

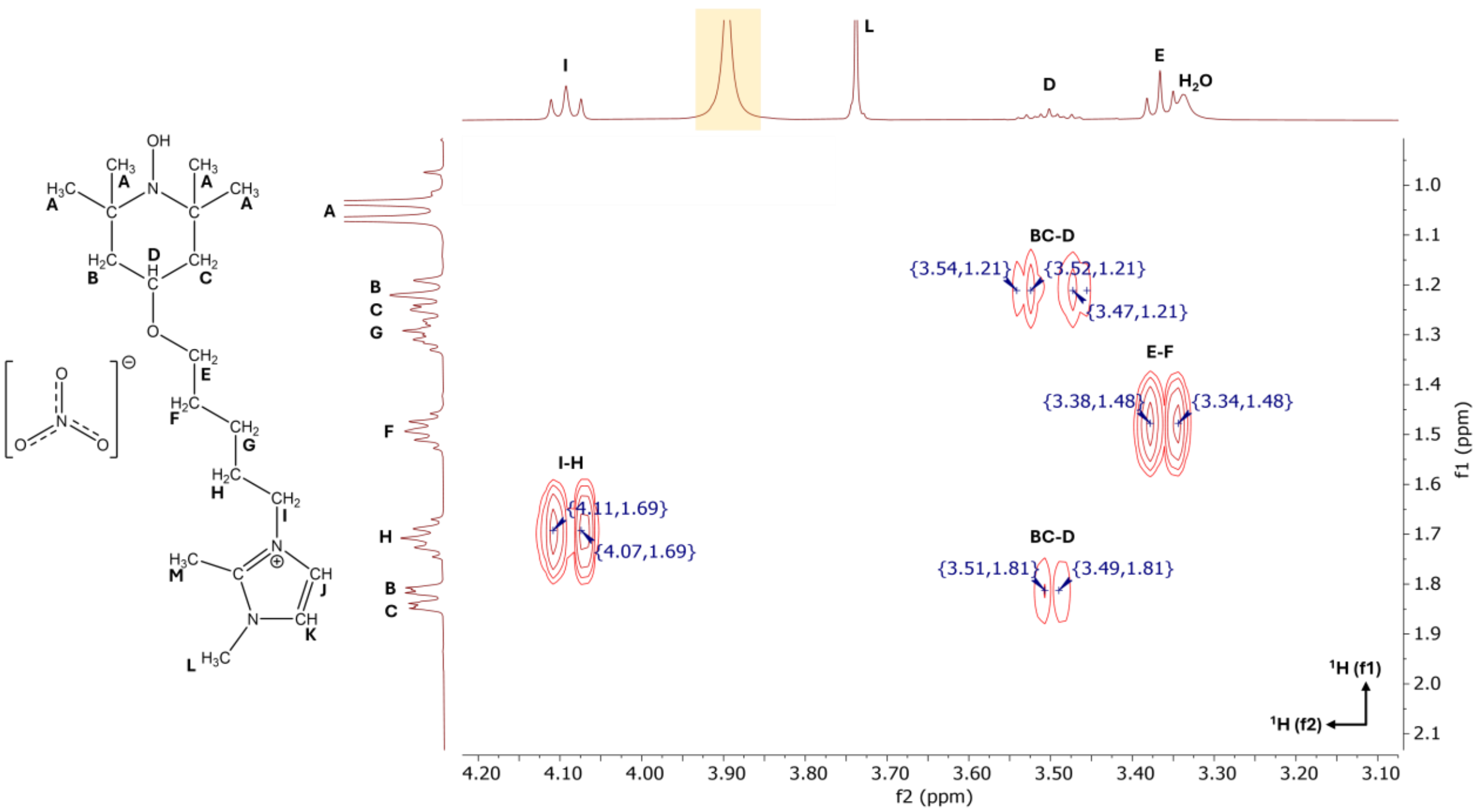

Figure S41 | Zoomed-in region 2 of the 2D ^1H-^1H COSY NMR spectrum of [Im_{TEMPO}][NO_3] (**4c**) dissolved in DMSO-d_6. Cross peaks on the dotted line indicate self-correlation cross peaks. The peaks/cross peaks highlighted in yellow correspond to $PhNHNH_2$.

2D ^1H-^1H COSY NMR (400 MHz, DMSO-d_6):

f_2 (ppm)	f_1 (ppm)	^1H-^1H correlation
3.38	1.48	H_E-H_F
3.34	1.48	
3.54	1.21	H_BH_C-H_D
3.52	1.21	
3.47	1.21	
3.51	1.81	
3.49	1.81	
4.11	1.69	H_H-H_I
4.07	1.69	

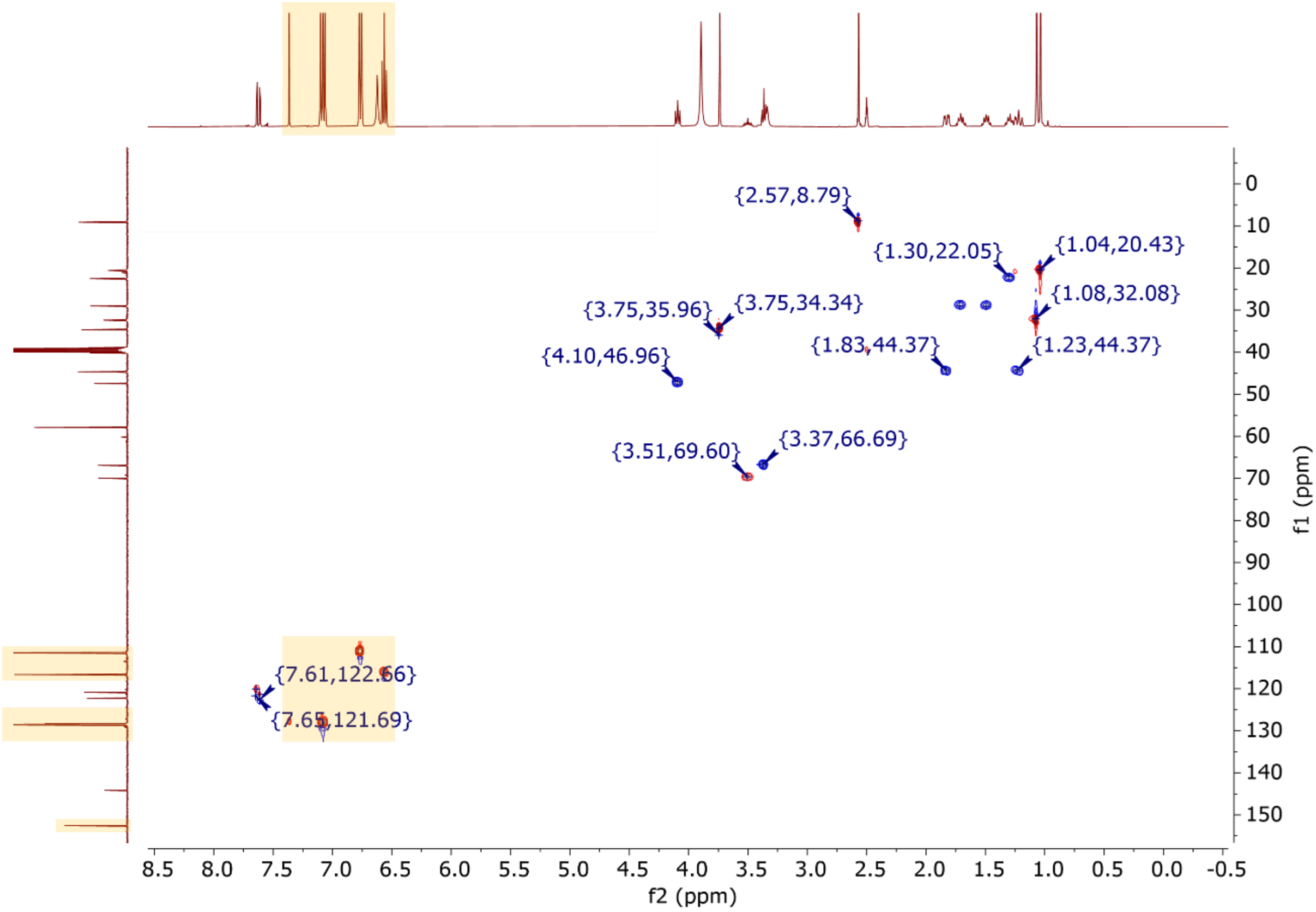

Figure S42 | Full-scale 2D ^{1}H-^{13}C edited HSQC NMR spectrum of $[Im_{TEMPO}][NO_3]$ (**4c**) in DMSO-d_6. The shaded yellow regions represent peaks/cross peaks related to $PhNHNH_2$. Blue cross peaks indicate that these correlations stem from CH_2 (secondary carbon) units and red cross peaks indicate that the carbon is either tertiary (CH) or primary (CH_3).

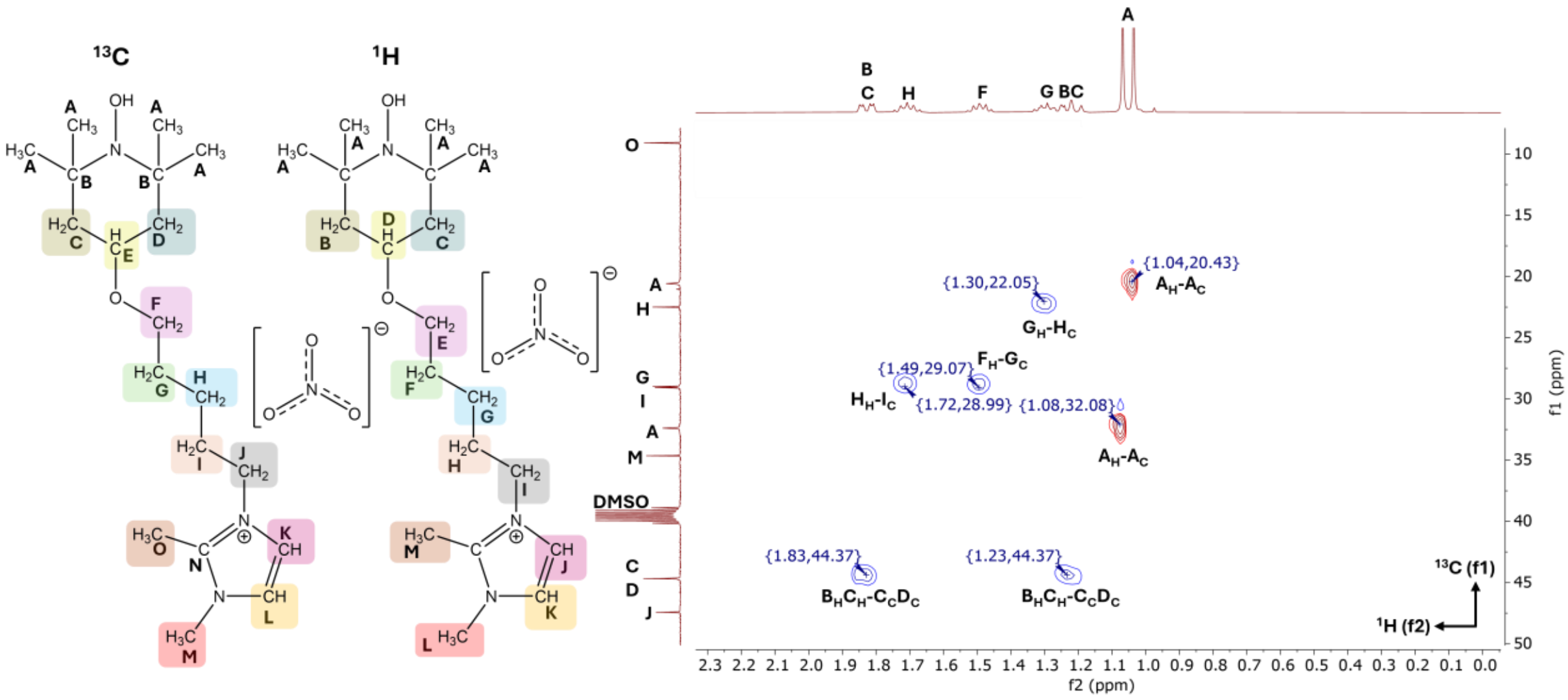

Figure S43 | Zoomed-in region 1 of the 2D ^{1}H-^{13}C edited HSQC NMR spectrum of $[Im_{TEMPO}][NO_3]$ (**4c**) in DMSO-d_6. Blue cross peaks indicate that these correlations stem from CH_2 (secondary carbon) units and red cross peaks indicate that the carbon is either tertiary (CH) or primary (CH_3).

2D ^{1}H-^{13}C edited HSQC NMR (400 MHz (^{1}H), 100 MHz (^{13}C), DMSO-d_6):

f_2, ^{1}H (ppm)	f_1, ^{13}C (ppm)	^{1}H-^{13}C correlation
1.04	20.43	A_H-A_C
1.08	32.08	
1.30	22.05	G_H-H_C
1.49	29.07	F_H-G_C
1.72	28.99	H_H-I_C
1.23	44.37	B_HC_H-C_CD_C
1.83	44.37	

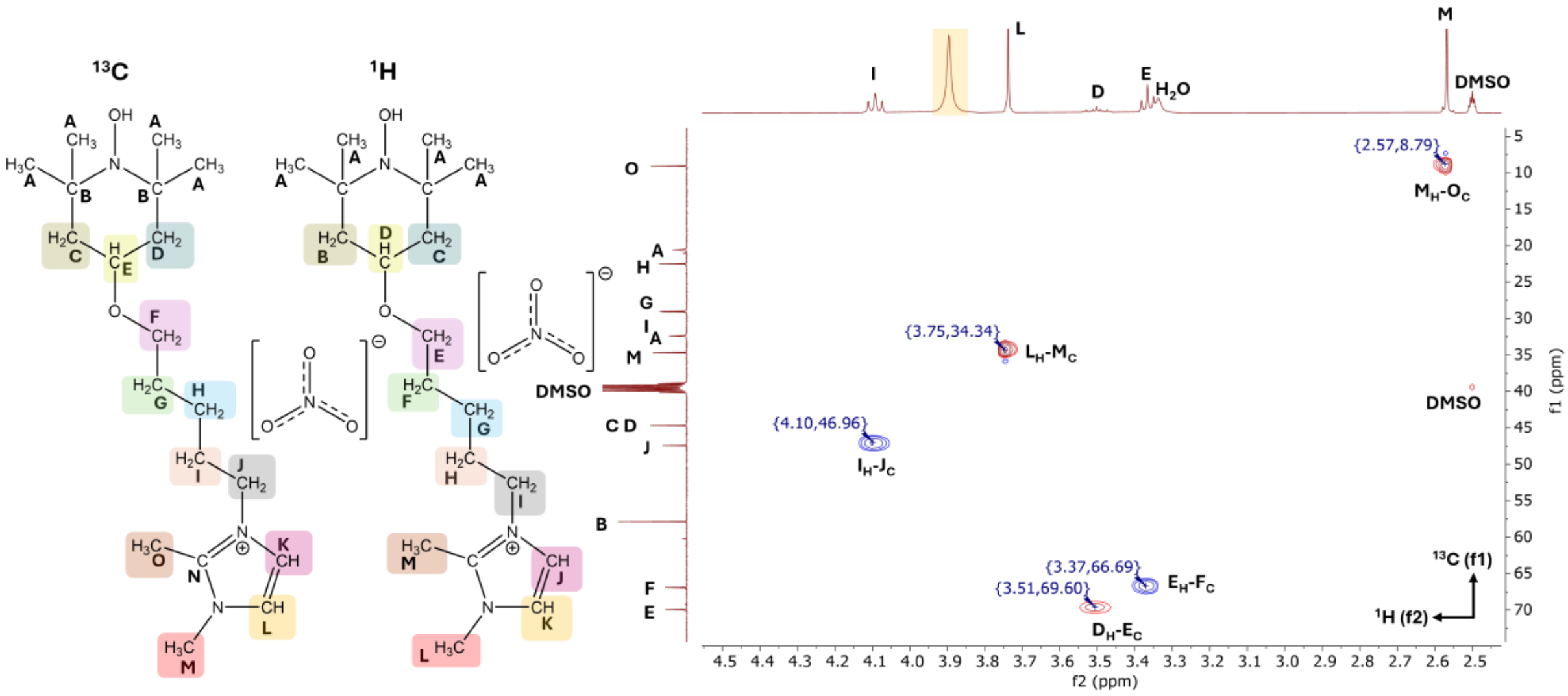

Figure S44 | Zoomed-in region 2 of the 2D ^1H-^{13}C edited HSQC NMR spectrum of [Im$_{TEMPO}$][NO_3] (**4c**) in DMSO-d_6. Peaks highlighted in yellow are associated with $PhNHNH_2$. Blue cross peaks indicate that these correlations stem from CH_2 (secondary carbon) units and red cross peaks indicate that the carbon is either tertiary (CH) or primary (CH_3).

2D ^1H-^{13}C edited HSQC NMR (400 MHz (^1H), 100 MHz (^{13}C), DMSO-d_6):

f_2, ^1H (ppm)	f_1, ^{13}C (ppm)	^1H-^{13}C correlation
3.37	66.69	E_H-F_C
3.51	69.60	D_H-E_C
3.75	34.34	L_H-M_C
4.10	46.96	I_H-J_C
2.57	8.79	M_H-O_C

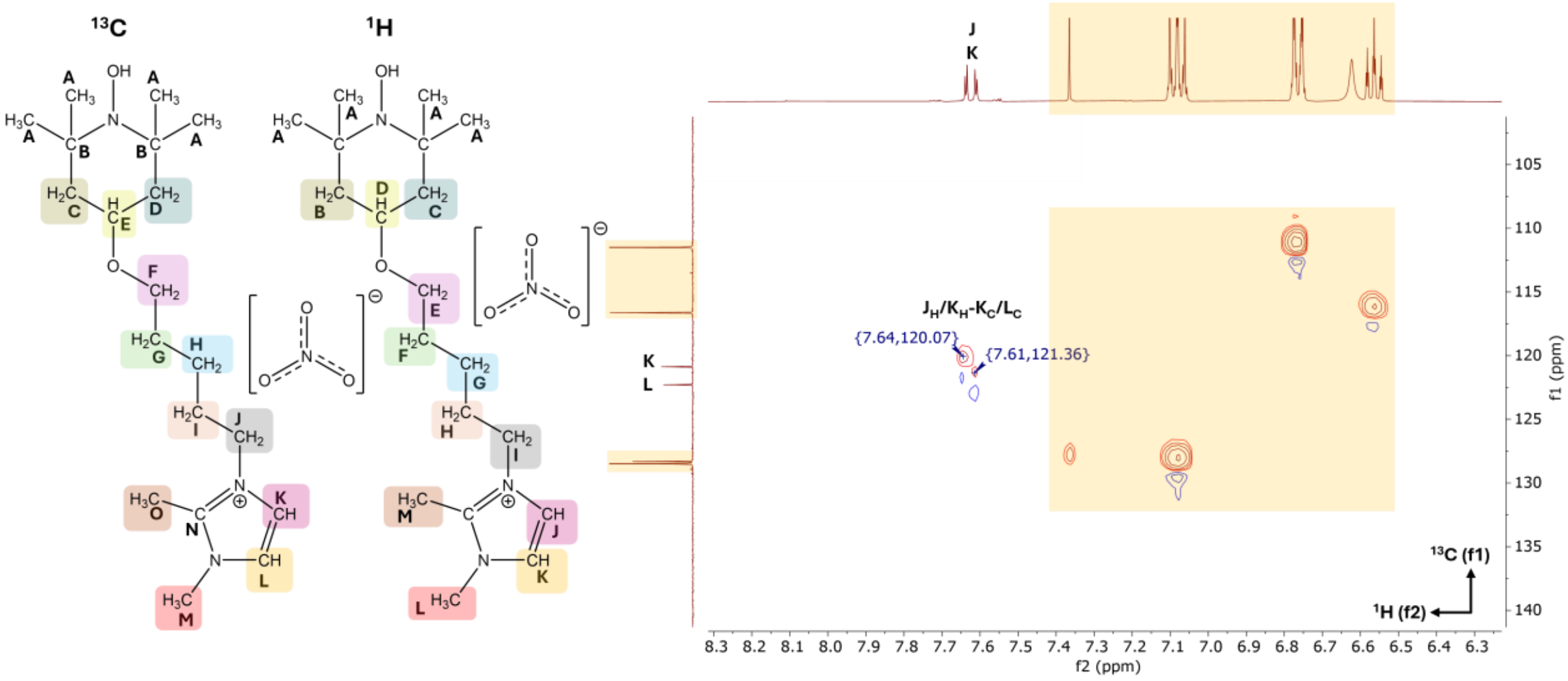

Figure S45 | Zoomed-in region 3 of the 2D ^{1}H-^{13}C edited HSQC NMR spectrum of [Im$_{TEMPO}$][NO_3] (**4c**) in DMSO-d_6. The shaded yellow regions represent peaks/cross peaks related to $PhNHNH_2$. Blue cross peaks indicate that these correlations stem from CH_2 (secondary carbon) units and red cross peaks indicate that the carbon is either tertiary (CH) or primary (CH_3).

2D ^{1}H-^{13}C edited HSQC NMR (400 MHz (^{1}H), 100 MHz (^{13}C), DMSO-d_6):

f_2, ^{1}H (ppm)	f_1, ^{13}C (ppm)	^{1}H-^{13}C correlation
7.61	121.36	J_H/K_H-K_C/L_C
7.64	120.07	

ATR-FTIR spectroscopy

ATR-FTIR was used to characterise the functional groups within the synthesised RMs. This was performed inside an N_2-filled glovebox (H_2O and O_2 < 0.1 ppm) on a Nicolet iS50 ATR-FTIR spectrometer (Thermo Scientific, US).

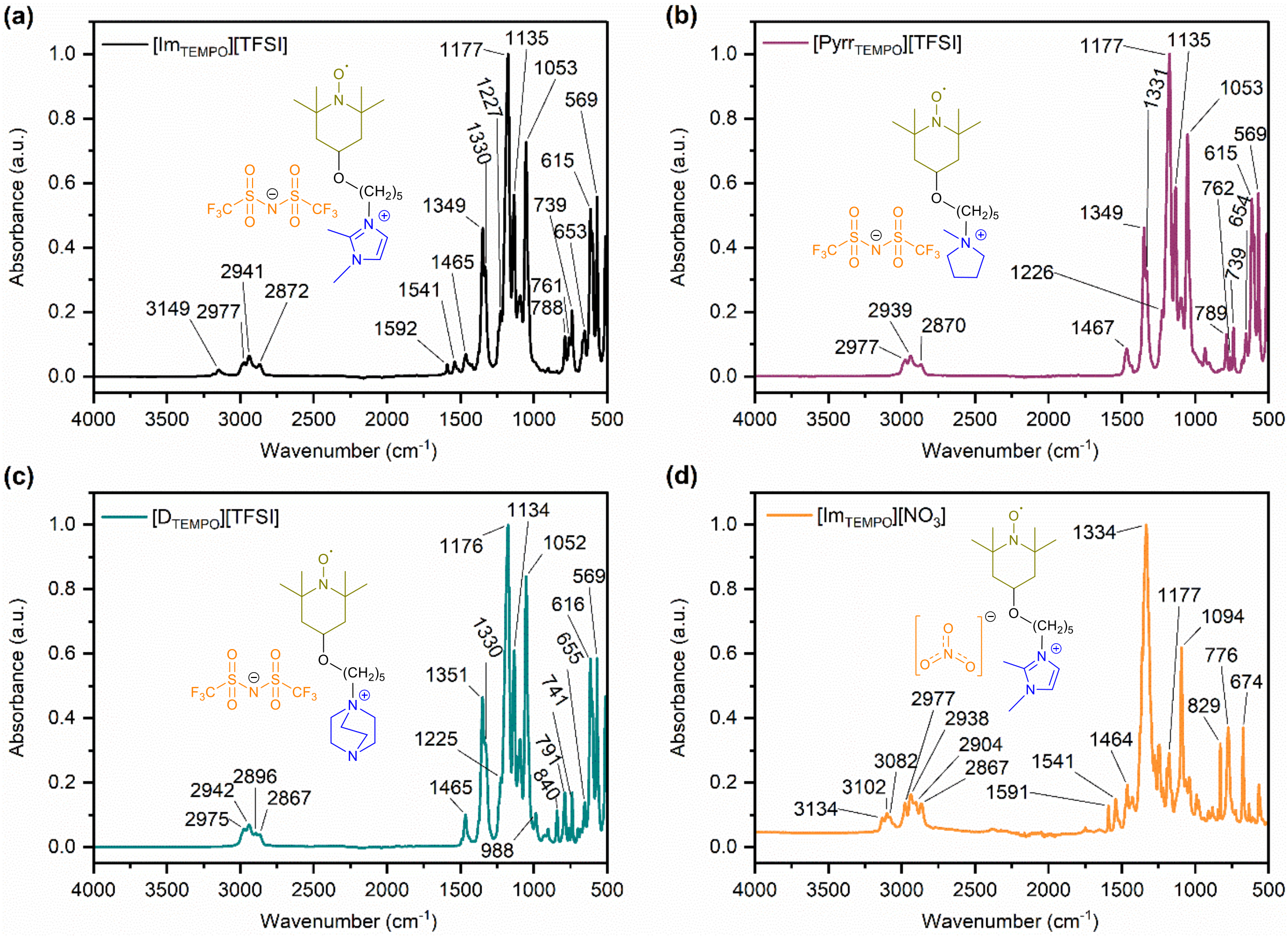

Figure S46 | ATR-FTIR spectra of **(a)** [Im_{TEMPO}][TFSI], **(b)** [$Pyrr_{TEMPO}$][TFSI], **(c)** [D_{TEMPO}][TFSI] and **(d)** [Im_{TEMPO}][NO_3].

The assignments below have been made based on work by Zhang *et al.*[3], Babushkina[4], Kovalenko *et al.*[5] and Zulkifli *et al.*[6]

[Im_{TEMPO}][TFSI]

ATR-FTIR (ṽ, cm^{-1}): 3149 (w, C-H (sp^2)), 2977, 2941, 2872 (m, C-H (sp^3)), 1592, 1541, 1465 (w-m, skeletal str. of Im ring), 1349, 1330 (s, SO_2 asym. str.), 1227 (m, CF_3 sym. str.), 1177 (s, N-CH_2 and N-CH_3 C-N str., C-C str., CF_3 asym. str.), 1135 (s, SO_2 sym. str.), 1053 (s, C-O, ether), 788, 761 (m, skeletal asym. bend of Im ring), 739 (m, CF_3 sym. bend), 653 (m, S-N-S bend), 615 (m, SO_2 asym. bend), 569 (s, CF_3 asym. bend).

[$Pyrr_{TEMPO}$][TFSI]

ATR-FTIR (ṽ, cm^{-1}): 2977, 2939, 2870 (m, C-H (sp^3)), 1467 (m, N-CH_3 sciss.), 1349, 1331 (s, SO_2 asym. str.), 1226 (m, CF_3 sym. str.), 1177 (s, N-CH_2, C-N, C-C, CF_3 asym. str.), 1135 (s, SO_2 sym. str.), 1053 (s, C-O, ether), 789, 762 (w-m, skeletal asym. bend of Pyrr ring), 739 (m, CF_3 sym. bend), 654 (m, S-N-S bend), 615 (m, SO_2 asym. bend), 569 (s, CF_3 asym. bend).

[D_{TEMPO}][TFSI]

ATR-FTIR (ṽ, cm^{-1}): 2975, 2942, 2896, 2867 (m, C-H (sp^3)), 1465 (m, CH_2 sciss. in DABCO), 1351, 1330 (s, SO_2 asym. str.), 1225 (m, CF_3 sym. str.), 1176 (s, N-CH_2, C-N, C-C, CF_3 asym. str.), 1134 (s, SO_2 sym. str.), 1052 (s, C-O, ether), 988 (w, NC_3 sym. bend in DABCOnium), 840 (m, CH_2 rock. and C-N-C bend. in DABCOnium), 791 (w-m, skeletal asym. bend of Pyrr ring), 741 (m, CF_3 sym. bend), 655 (m, S-N-S bend), 616 (m, SO_2 asym. bend), 569 (s, CF_3 asym. bend).

[Im_{TEMPO}][NO_3]

ATR-FTIR (ṽ, cm^{-1}): 3134, 3102, 3082 (m, C-H (sp^3)), 2977, 2938, 2904, 2867 (m, C-H (sp^3)), 1591, 1541, 1465 (w-m, skeletal str. of Im ring), 1334 (s, $[NO_3]^-$ asym. str.), 1177 (s, N-CH_2 and N-CH_3 C-N str., C-C str., CF_3 asym. str.), 1094 (s, C-O, ether), 829 (s, $[NO_3]^-$ twist/wag.) 776 (s, skeletal asym. bend of Im ring).

CHN elemental microanalysis

CHN elemental microanalysis was employed to quantify purity of the synthesised mediators using a FlashSmart Elemental Analyser (Thermo Fisher Scientific, US). The analysis was conducted by Analytical Services (Department of Chemistry, University of Liverpool, UK) and quoted CHN content in **Table S2** was based on an average of two runs per sample.

Table S2: A summary of CHN content for each synthesised RM.

Redox mediator	Element	Theoretical CHN content	Experimentally found CHN content	% Difference
$[Im_{TEMPO}][TFSI]$	C	40.84	40.86	+0.02
	H	5.71	5.68	-0.03
	N	9.07	9.04	-0.03
$[Pyrr_{TEMPO}][TFSI]$	C	41.58	41.60	+0.02
	H	6.31	6.26	-0.05
	N	6.93	6.89	-0.04
$[D_{TEMPO}][TFSI]$	C	41.70	41.64	-0.06
	H	6.20	6.16	-0.04
	N	8.84	8.92	+0.08
$[Im_{TEMPO}][NO_3]$	C	57.12	57.03	-0.09
	H	8.83	8.68	-0.15
	N	14.02	14.23	+0.21

Electrospray ionisation mass spectrometry

ESI-MS was used for accurate molecular weight determination of the synthesised RMs using a high-resolution ESI-MS time-of-flight (TOF) spectrometer (MicroMass, UK). Samples were run by Analytical Services (Department of Chemistry, University of Liverpool, UK) and were first dissolved in acetonitrile (~10 µg mL^{-1}) and analysed *via* direct injection into the MS ion source in both positive and negative ionisation mode.

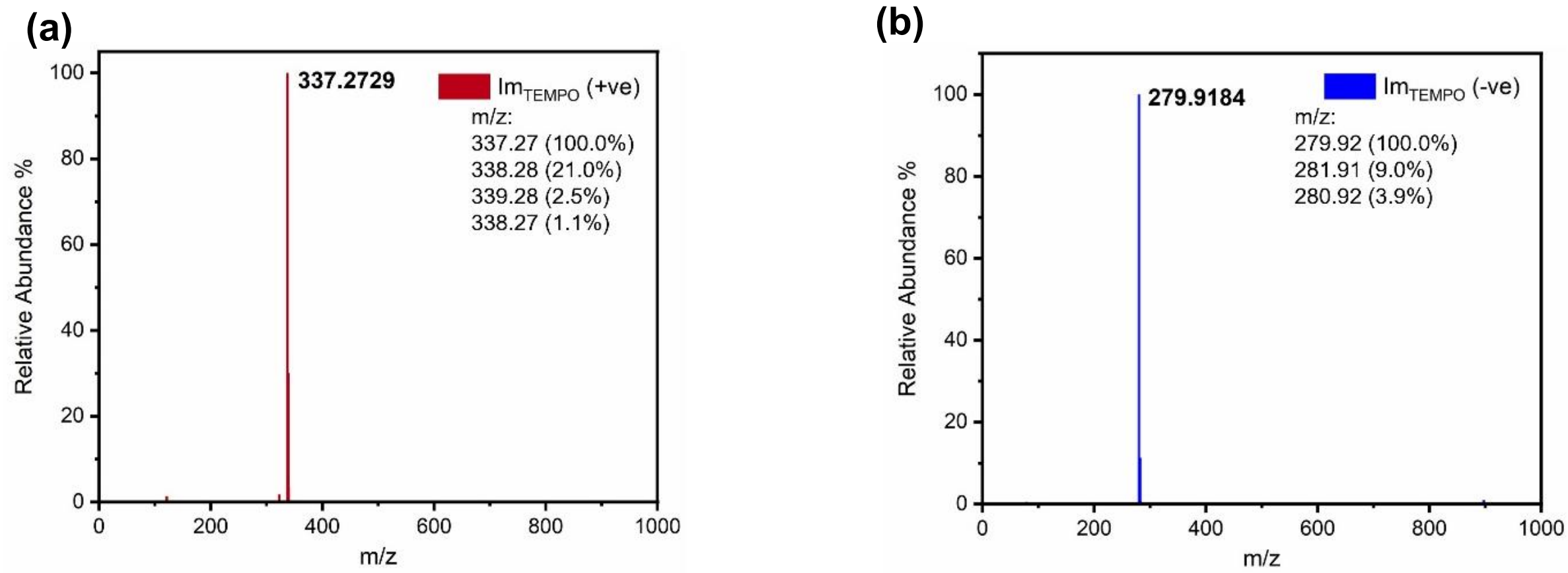

Figure S47 | ESI-MS spectra in **(a)** positive (+ve) and **(b)** negative (-ve) ionisation modes of [Im_{TEMPO}][TFSI]. The m/z values and relative abundances predicted by Chemdraw Professional software for $[Im_{TEMPO}]^+$ and $[TFSI]^-$ are included as well. The absolute abundance is normalised with respect to the highest abundance peak (base peak) to give the relative abundance.

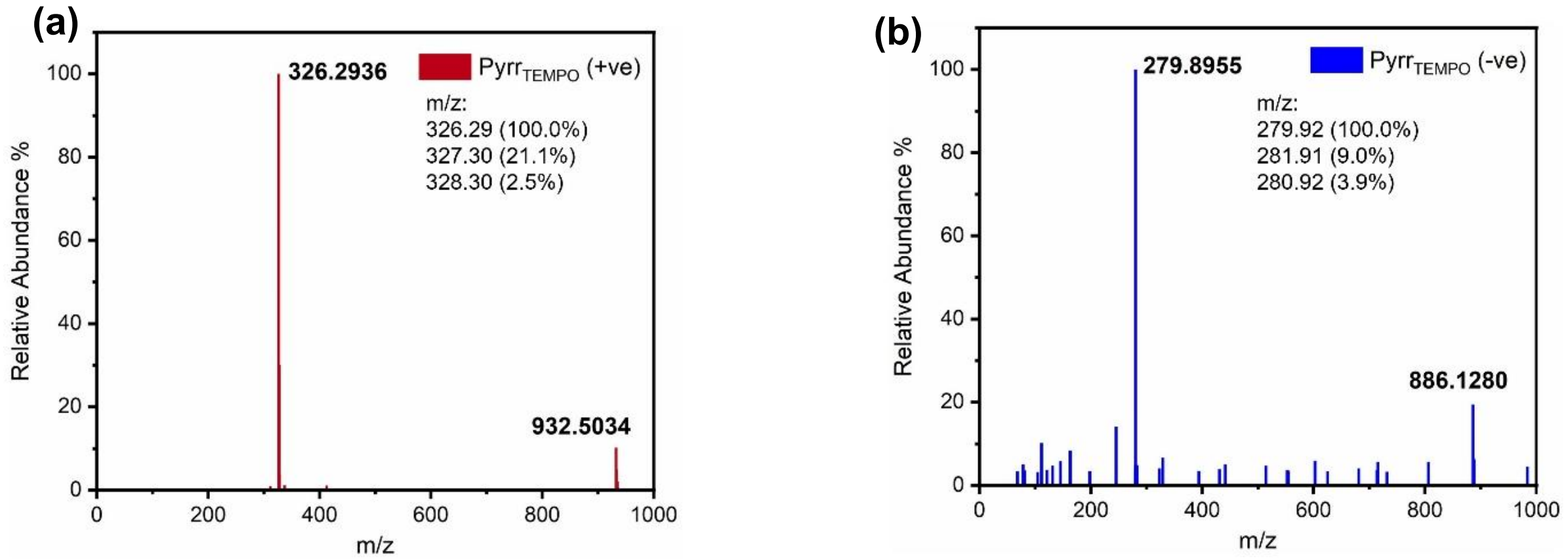

Figure S48 | ESI-MS spectra in **(a)** positive (+ve) and **(b)** negative (-ve) ionisation modes of [Pyrr$_{TEMPO}$][TFSI]. The m/z values and relative abundances predicted by Chemdraw Professional software for [Pyrr$_{TEMPO}$]$^+$ and [TFSI]$^-$ are included as well. The absolute abundance is normalised with respect to the highest abundance peak (base peak) to give the relative abundance.

In **Figure S48**, for both mass spectra, peaks at m/z values far exceeding the molecular weight of the cation and anion are present. This is a common phenomenon previously reported for ESI-MS of ionic liquids and is explained by the formation of cation-anion clusters.[7] The clusters possess either a net negative or positive charge for negative and positive ionisation modes, respectively. For example, the peak at m/z 932.5034 in the positive ionisation mode spectrum (**Figure S48a**) is consistent with a [(cation-anion)(cation)]$^+$ cluster. Similarly, in the negative ionisation mode (**Figure S48b**), a peak at m/z 886.1280 is apparent. This corresponds to a cluster consisting of two anions and one cation, i.e. [(cation-anion)(anion)]$^-$. There appears to be significantly more fragmentation in the negative ionisation scan for [Pyrr$_{TEMPO}$][TFSI] (**Figure S48b**) than for [Im$_{TEMPO}$][TFSI] (**Figure S47b**) given the large number of peaks with relative abundance <20%. On closer inspection, the absolute abundance of these peaks in -ve mass spectrum of [Pyrr$_{TEMPO}$][TFSI] is comparable to that of [Im$_{TEMPO}$][TFSI], if not lower. However, the absolute abundance of the base peak corresponding to the [TFSI]$^-$ anion is two orders of magnitude lower in [Pyrr$_{TEMPO}$][TFSI] relative to [Im$_{TEMPO}$][TFSI]. This may be related to the formation of cation-anion clusters in the negative ionisation mode, as detailed above, which was not observed for [Im$_{TEMPO}$][TFSI].

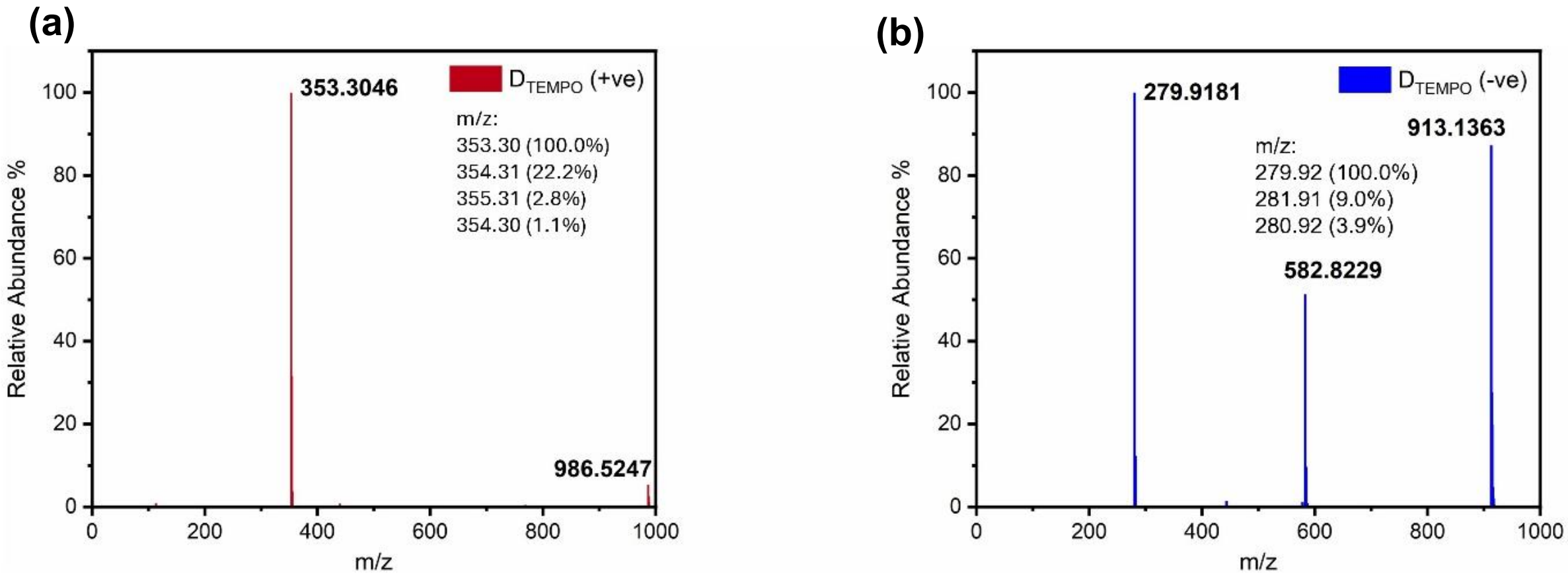

Figure S49 | ESI-MS spectra in **(a)** positive (+ve) and **(b)** negative (-ve) ionisation modes of [D$_{TEMPO}$][TFSI]. The m/z values and relative abundances predicted by Chemdraw Professional software for [D$_{TEMPO}$]$^+$ and [TFSI]$^-$ are included as well. The absolute abundance is normalised with respect to the highest abundance peak (base peak) to give the relative abundance.

As was observed in the mass spectra of [Pyrr$_{TEMPO}$][TFSI], cation-anion cluster peaks are also apparent. The peaks at m/z 986.5247 and m/z 913.1363 correspond to clusters of the type [(cation-anion)(cation)]$^+$ and [(cation-anion)(anion)]$^-$ in the positive and negative ionisation spectra, respectively. However, the peak at m/z 582.8229 in the negative ionisation mass spectrum, assuming a singly charged ion, is not of sufficient mass to form a cluster with net negative charge that incorporates the cation. The m/z of two [TFSI]$^-$ anions would correspond to 559.8362, which is exactly 23 units from the peak at m/z 582.8229. This difference corresponds to the m/z of Na^+, which is a common contaminant in organic solvents stemming from glassware and solvent impurities and readily forms adducts in ESI-MS measurements.[7] Therefore, a possible origin of the peak at m/z 582.8229 is a cation-anion cluster of the type [(cation-anion)(anion)]$^-$, where the cation is Na^+.

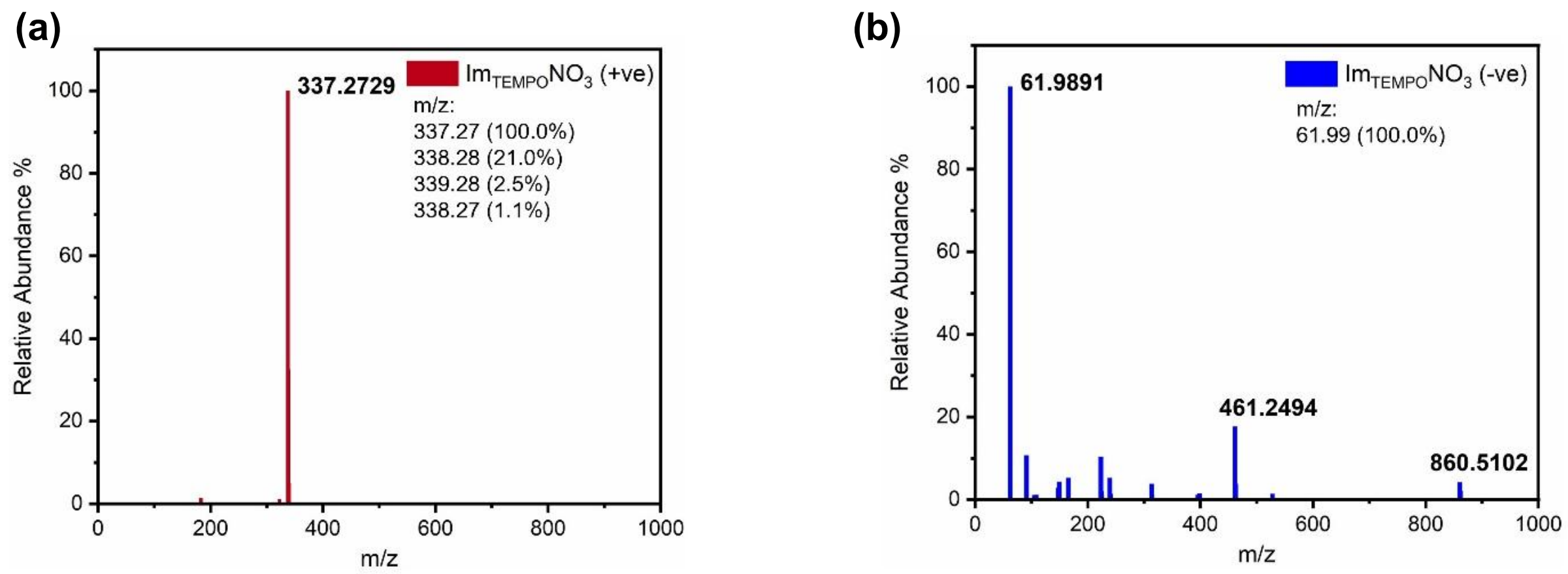

Figure S50 | ESI-MS spectra in **(a)** positive (+ve) and **(b)** negative (-ve) ionisation modes of [Im_{TEMPO}][NO_3]. The m/z values and relative abundances predicted by Chemdraw Professional software for [Im_{TEMPO}]$^+$ and [NO_3]$^-$ are included as well. The absolute abundance is normalised with respect to the highest abundance peak (base peak) to give the relative abundance.

The peak at m/z 61.9891 in the negative ionisation scan corresponds to the nitrate anion. Importantly, there is no peak at m/z 79 and m/z 81, which would indicate the presence of bromide. This suggests that the anion metathesis with [Im_{TEMPO}][Br] and Ag[NO_3] was successful and is further corroborated by CHN elemental microanalysis (**Table S2**). The higher nitrogen content (relative to [Im_{TEMPO}][TFSI], [$Pyrr_{TEMPO}$][TFSI] and [D_{TEMPO}][TFSI]) found experimentally due to the presence of nitrate is in line with the theoretical value.

Thermal stability

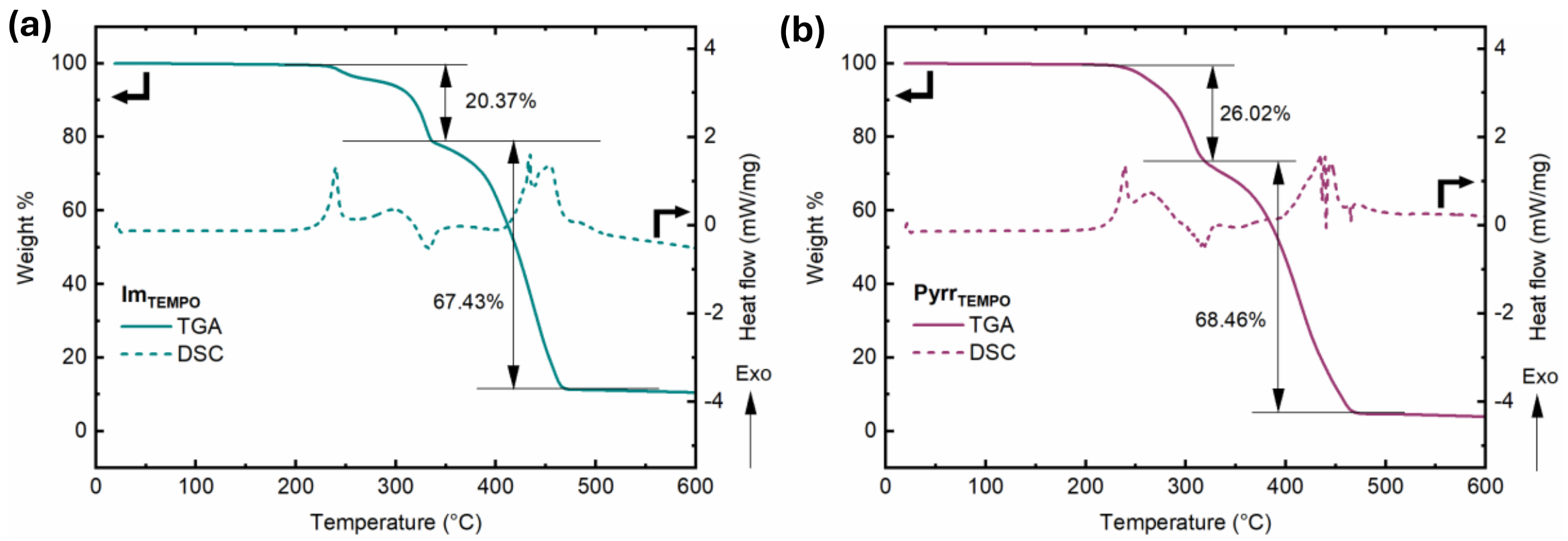

Figure S51 | TGA and DSC thermograms of **(a)** [Im_{TEMPO}][TFSI] and **(b)** [$Pyrr_{TEMPO}$][TFSI]. For DSC thermograms, positive peaks represent exothermic events, while negative peaks indicate endothermic processes.

The TGA and DSC thermograms for [Im_{TEMPO}][TFSI] and [$Pyrr_{TEMPO}$][TFSI] are presented in **Figure S51**. For both RMs, the TGA thermograms indicate there is no significant mass loss between 20 to ~230 °C. This is followed by a 20% mass loss event occurring between 230 to ~340 °C for [Im_{TEMPO}][TFSI], and a 26% mass loss between 230 to ~320 °C for [$Pyrr_{TEMPO}$][TFSI]. In both cases, these mass losses were exothermic as indicated by the positive peaks in the DSC thermogram. In the case of [Im_{TEMPO}][TFSI], the above mass loss is consistent with measurements by Tkacheva *et al.*[1] and was attributed to the loss of the TEMPO moiety based on the percent mass loss. Both RMs are fully decomposed at ~470 °C. The lack of any significant mass loss below 200 °C supports the fact that no volatile organics and/or water were present in the samples, which is in agreement with the high purity in terms of carbon, hydrogen and nitrogen content determined by elemental microanalysis (**Table S2**). A similar TGA/DSC response with two mass loss events was observed for [D_{TEMPO}][TFSI]. The associated thermograms are shown in **Figure S52**.

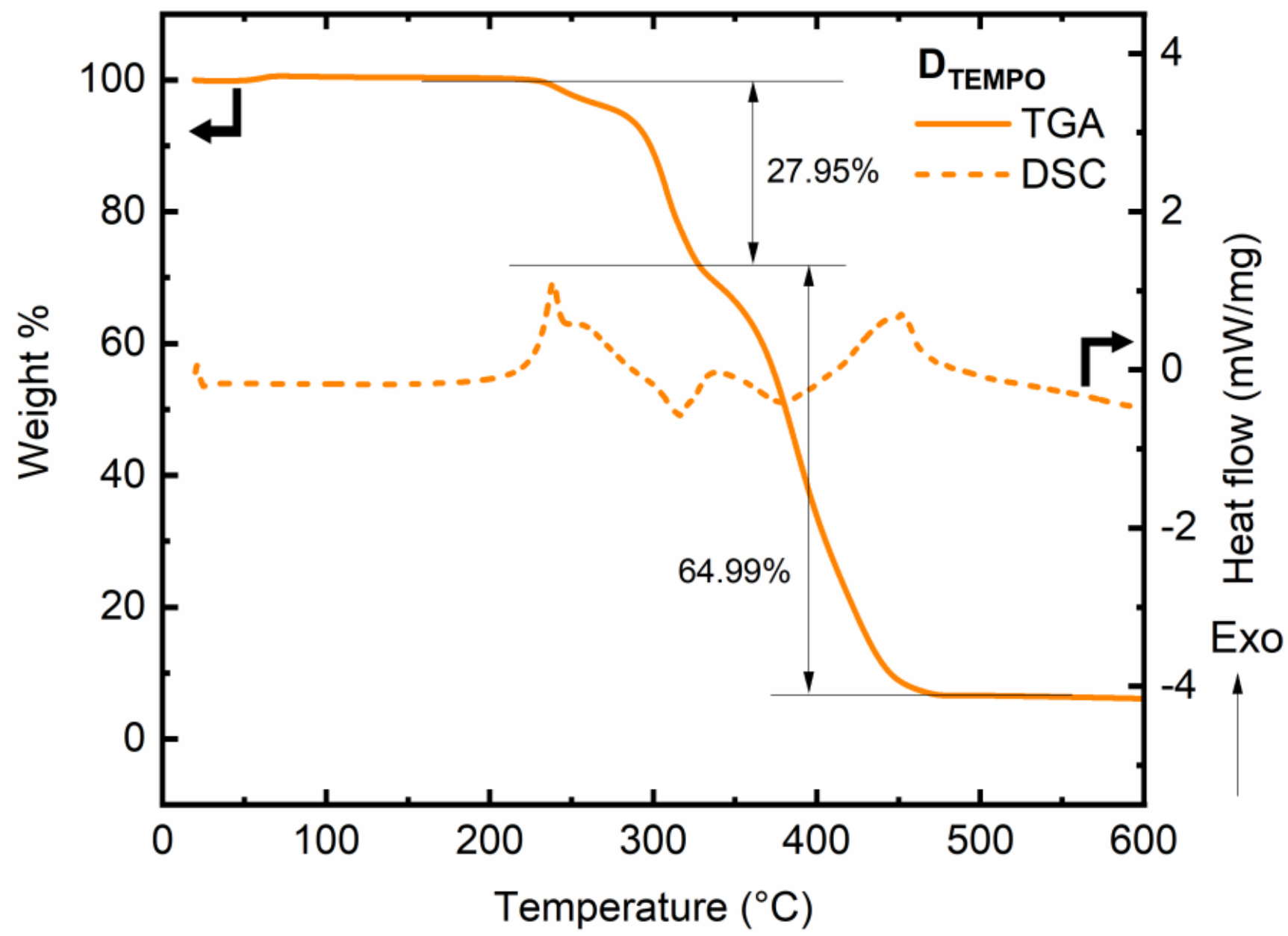

Figure S52 | TGA and DSC thermograms of [D_{TEMPO}][TFSI]. For DSC thermograms, positive peaks represent exothermic events, while negative peaks indicate endothermic processes.

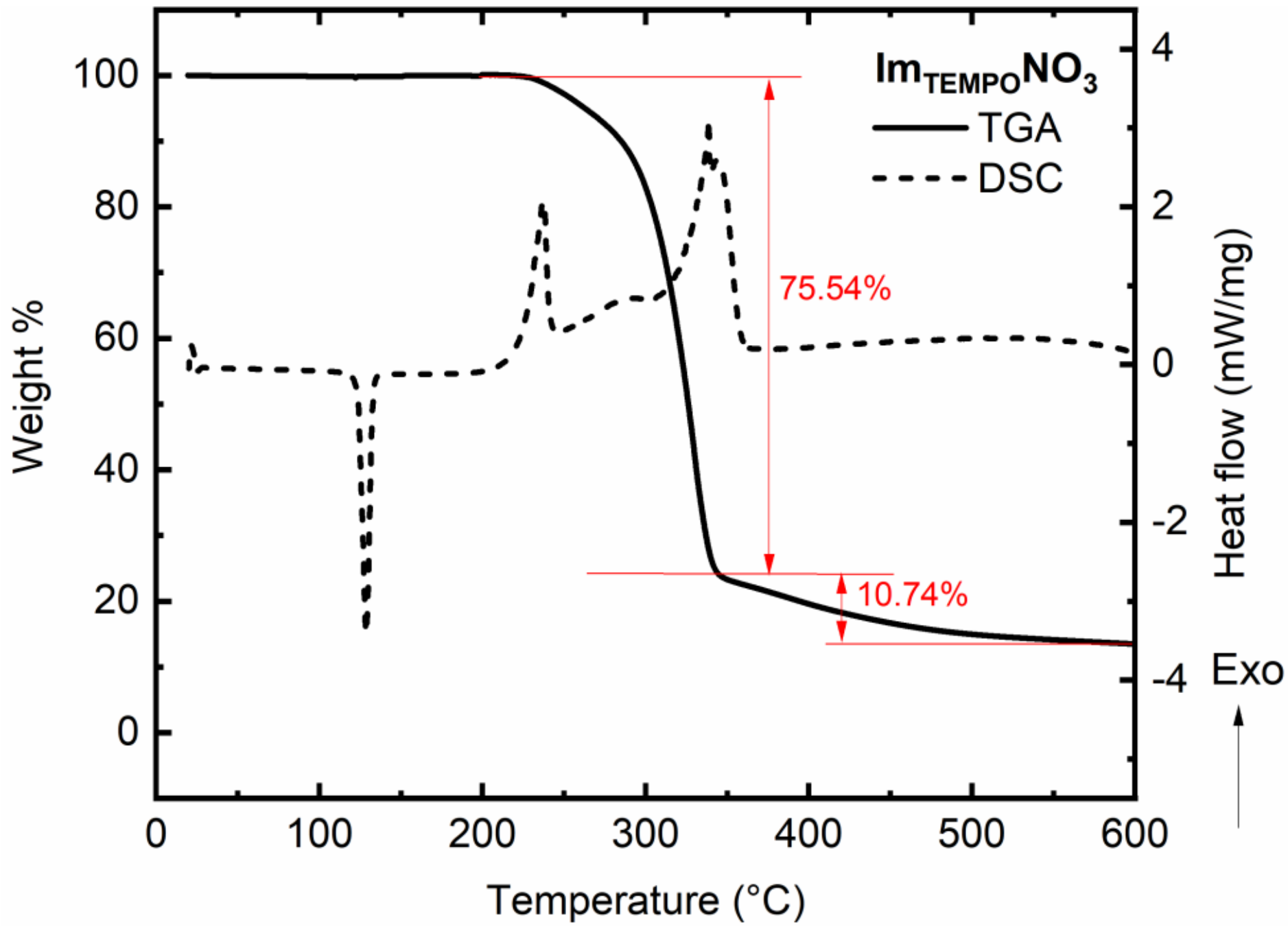

Figure S53 | TGA and DSC thermograms of [Im$_{TEMPO}$][NO_3]. For DSC thermograms, positive peaks represent exothermic events, while negative peaks indicate endothermic processes.

[Im$_{TEMPO}$][NO_3] exhibits a large exothermic mass loss event (76% mass loss) between 225 – 345 °C followed by an 11% loss from 345 – 600 °C (**Figure 3.29**). The significant mass loss in the first step suggests concerted total decomposition of [Im$_{TEMPO}$][NO_3]. Similar to the previous three tethered RMs, no significant mass loss occurred below 200 °C, indicating the absence of moisture or volatile organics in the sample. The sharp endothermic peak at 130 °C in the DSC thermogram does not coincide with any mass loss and is characteristic of melting of [Im$_{TEMPO}$][NO_3], as is also the case for Li[NO_3], albeit at 255 °C.[8]

Standard lithium-oxygen cell and preparation of cell components

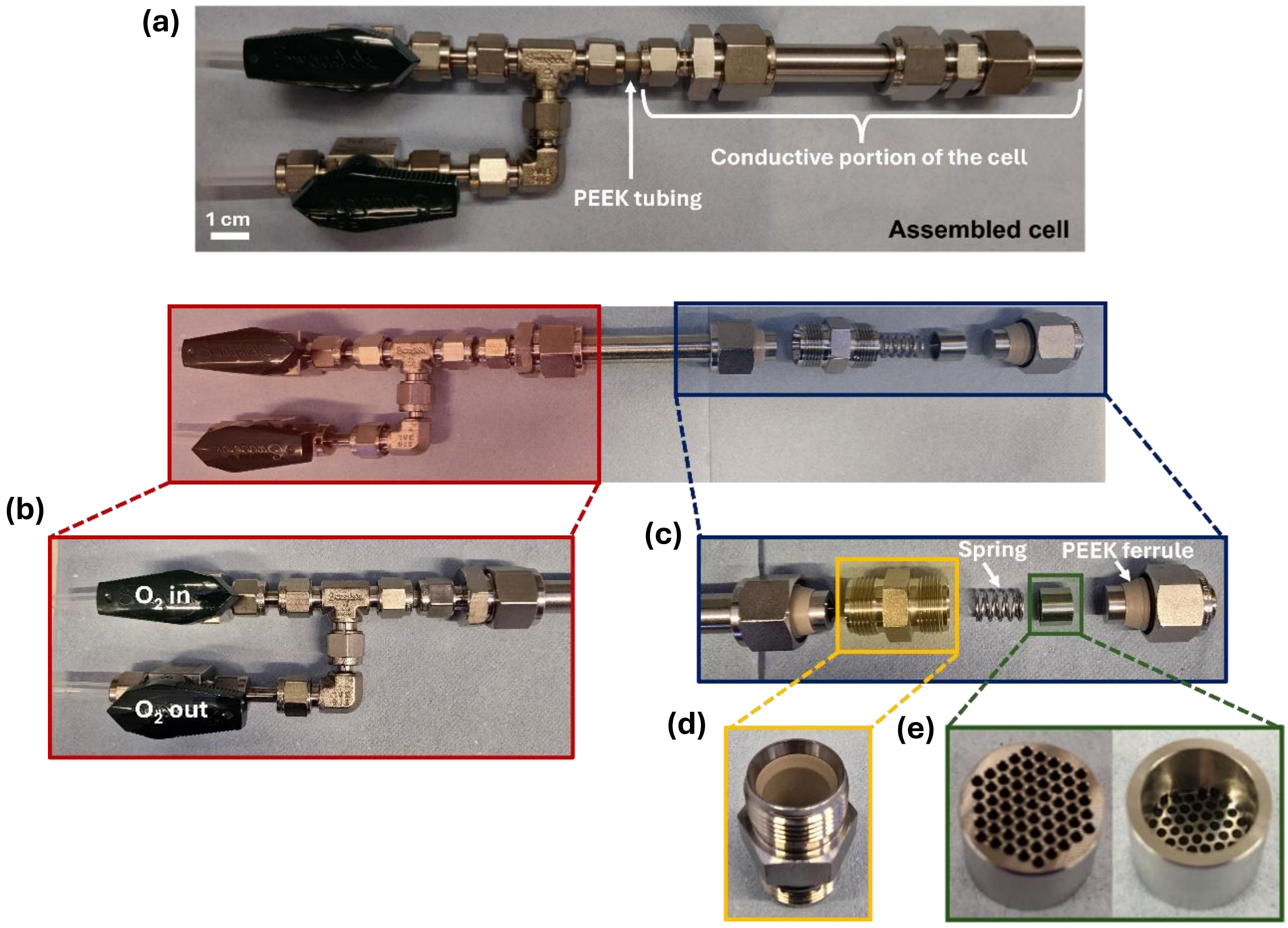

Figure S54 | Images of the standard Li-O_2 cell showing **(a)** the assembled cell, highlighting a piece of polyether ether ketone (PEEK) tubing, internally reinforced with stainless-steel, which was used to reduce the conductive portion of the cell, **(b)** quarter-turn valves with tubing to connect the cell to a high purity oxygen line for cell purging, **(c)** PEEK-lined cell body housing the stack (stainless steel mesh, carbon paper positive electrode, one or two glass fibre separators soaked with electrolyte and a Li metal or partially delithiated lithium iron phosphate counter electrode), which is under spring-loaded compression when the cell is sealed, **(d)** a top-down view of the cell body highlighting the PEEK lining, and **(e)** a stainless-steel cup with machined holes to aid uniform distribution of O_2 over the carbon electrode surface.

Carbon paper (P50), stainless-steel mesh and glass fibre separators were punched to the required diameter (⌀ 10 mm) and then washed successively with ethanol. They were then dried at 110 °C in a tube oven (Buchi, Switzerland) for 15 h and then taken into an Ar-filled glovebox without exposure to air. Stainless-steel mesh (⌀ 12 mm), which sits between the machined stainless-steel cup and carbon electrode to improve O_2 distribution across the electrode, were also washed and dried in the same manner. Lithium metal was first brushed and polished to give a mirror-like surface finish and then punched to the required diameter (⌀ 12 mm).

Partially delithiated lithium iron phosphate ($Li_{1-x}FePO_4$) electrodes were prepared by first fabricating freestanding dough-like $LiFePO_4$ sheet. For this, $LiFePO_4$ powder, Super C65 carbon black and PTFE (60 wt% dispersion in water) in an 8:1:1 ratio were hand-mixed with the gradual addition of isopropanol until a dough-like consistency was achieved. This was then hand-rolled to a thickness of 0.3 mm and punched into ⌀ 12 mm discs. Chemical delithiation of the $LiFePO_4$ discs was achieved by immersing the discs in a solution of hydrogen peroxide (30%, 3.6 mL) and glacial acetic acid (99.7%, 1.5 mL) in deionised water (250 mL) for 30 minutes with gentle stirring. The $Li_{1-x}FePO_4$ discs were then washed successively with deionised water and ethanol, dried under vacuum at 120 °C for 15 h in a tube oven (Buchi, Switzerland) and transferred into an Ar-filled glovebox without exposure to air.

Table S3: Electrolyte formulations for Li-O_2 cells

Entry	Electrolyte components	$x_{solvent}$: $x_{Li\ salt}$	Molality		
			$mol_{solutes}$ $kg_{solvent}^{-1}$	$mol_{Li\ salt}$ $kg_{solvent}^{-1}$	$mol_{additives}$ $kg_{solvent}^{-1}$
1	Li[TFSI] Diglyme	9:1	0.828	0.828	-
2[a]	TEMPO/[Im_{TEMPO}][TFSI]/ [$Pyrr_{TEMPO}$][TFSI]/ [D_{TEMPO}][TFSI]/ [Im_{TEMPO}][NO_3]/Li[NO_3] Li[TFSI] Diglyme	9:1	0.853	0.828	0.0250
3[a]	TEMPO/[Im_{TEMPO}][TFSI]/ [$Pyrr_{TEMPO}$][TFSI]/ [D_{TEMPO}][TFSI]/ [Im_{TEMPO}][NO_3] Li[TFSI] Diglyme	9:1	0.963	0.828	0.135
4[b]	TEMPO + Li[NO_3] Li[TFSI] Diglyme	9:1	0.878	0.828	0.0250

x denotes the mole fraction. [a]One RM (either TEMPO, [Im_{TEMPO}][NO_3], [$Pyrr_{TEMPO}$][TFSI], [D_{TEMPO}][TFSI] **or** [Im_{TEMPO}][NO_3]) used per electrolyte. [b]For TEMPO + Li[NO_3] containing electrolytes, the $mol_{additives}$ $kg_{solvent}^{-1}$ refers to the molality of *each* additive (*i.e.*, 25 $mmol_{TEMPO}$ $kg_{solvent}^{-1}$ and 25 $mmol_{LiNO_3}$ $kg_{solvent}^{-1}$).

Cyclic voltammetry

The WE was polished on Emery microfibre cloth (Buehler, US) using slurries of alumina (particle sizes: 1.0, 0.3 and 0.05 μm) in deionised water. Polishing was done with alumina slurries in order of decreasing particle size. Between particle sizes, the electrode was rinsed several times with ethanol and ultrapure water (Mili-Q, Merck, UK) and sonicated for 1-2 min before polishing with an alumina slurry of smaller particle size. The polishing procedure was performed for every new CV experiment carried out. The Pt counter electrode (CE) was washed with ethanol and deionised water, dried with N_2 gas, and then annealed using a propane torch. The working electrode (WE) and CE electrode were oven-dried overnight at 80 °C before being moved into the glovebox for cell assembly.

The Ag[OTf]/Ag reference electrode (RE) was based on a design by Snook *et al.*[9] The RE consisted of a silver wire immersed in a glass capillary filled with 100 mM Ag[OTf] in 1-propyl-1-methylpyrrolidinium [TFSI] ([$Pyrr_{13}$][TFSI]) behind a porous glass frit. The design of this RE is shown in **Figure S55a**. Conversion to the Li^+/Li reference potential scale was achieved by measuring the redox potential of 10 mM ferrocene (Fc) in the electrolyte of interest using the Ag[OTf]/Ag and Li metal REs. The Li metal RE was prepared inside an Ar-filled glovebox and involved tightly wrapping a strip of polished Li-metal around a Cu rod (⌀ 3 mm). To ensure good metallic contact, the Cu rod was polished on sandpaper (grit: P400, RS Components, UK) and washed with ethanol and water prior to moving into the glovebox. With Li metal wrapped around the Cu rod, additional PTFE tape was applied to improve Li adhesion onto Cu. Care was taken here to ensure that PTFE tape did not come into contact with the electrolyte when assembling the CV cell. Following alignment of Fc redox potentials *vs.* Ag[OTf]/Ag and *vs.* Li^+/Li, it was found that 0 V *vs.* Ag[OTf]/Ag corresponded to 3.89 V *vs.* Li^+/Li with 1 M Li[TFSI] in diglyme as the electrolyte (**Figure S55b**). As this conversion factor is specific to this electrolyte, a separate calibration was performed when the supporting electrolyte was changed from Li[TFSI] to [$Pyrr_{13}$][TFSI], where it was determined that 0 V *vs.* Ag[OTf]/Ag = 4.06 V *vs.* Li^+/Li (relevant for CVs presented in **Figure 7** of the article, wherein the base electrolyte was 1 M [$Pyrr_{13}$][TFSI] in diglyme). An image of the cell setup for O_2 bubbling within an Ar-filled glovebox is shown in **Figure S55c**. For CVs conducted under O_2, electrolytes were bubbled with O_2 gas (pre-dried to <5 ppm H_2O) inside an Ar-filled glovebox for 30 min.

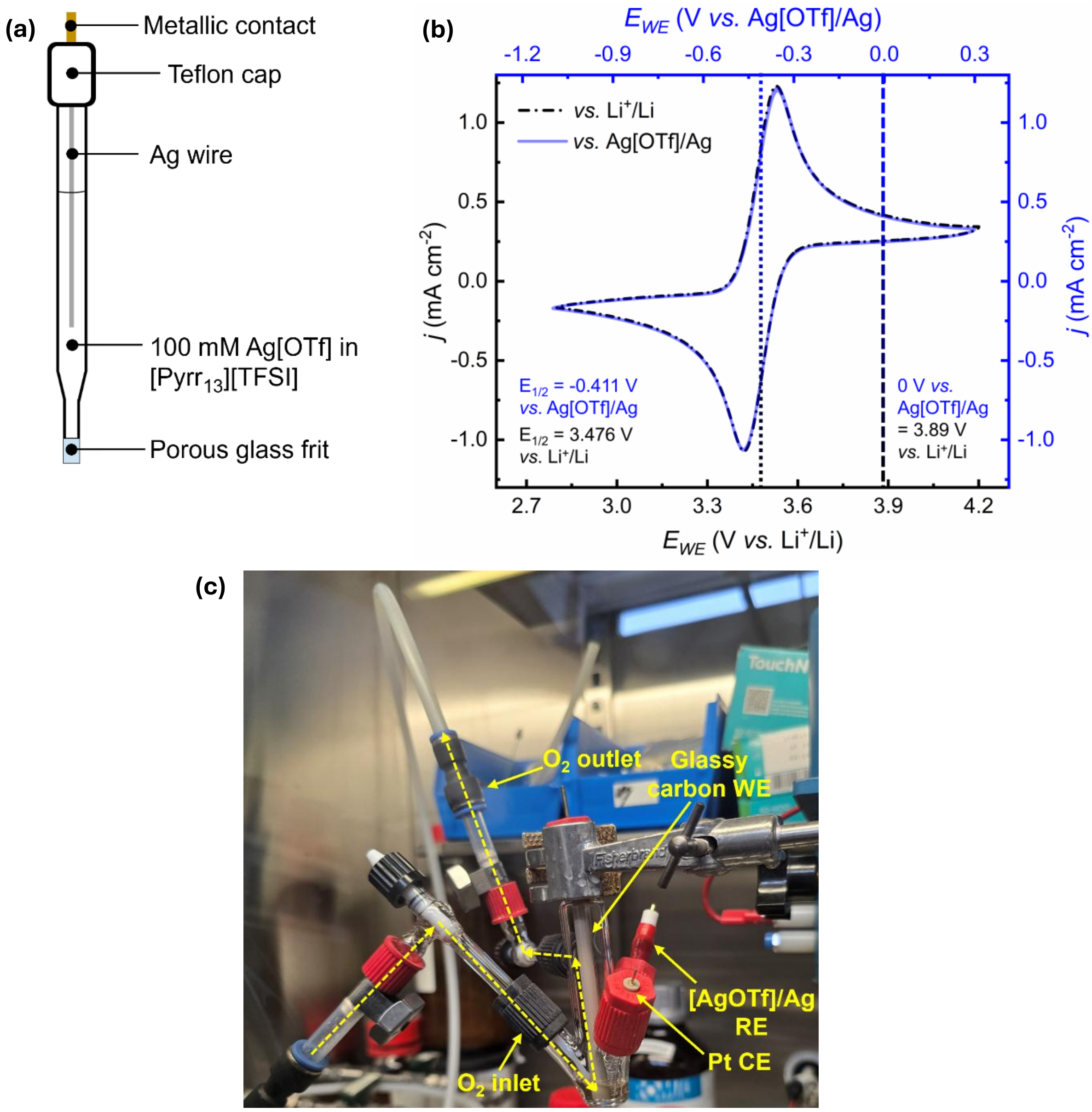

Figure S55 | (a) A schematic of the Ag[OTf]/Ag RE used for CV experiments involving O_2. The potentials measured relative to the Ag[OTf]/Ag RE were converted to potentials *vs.* Li^+/Li by acquiring **(b)** CVs of 10 mM Fc in 1 M Li[TFSI] in diglyme using Ag[OTf]/Ag and Li metal REs (CVs conducted at 50 mV s^{-1}). The redox potentials of Fc relative to both REs were aligned to derive a conversion factor between the reference scales. The dotted line indicates the redox potential of Fc *vs*. both REs and the dashed line shows the conversion factor for this electrolyte. **(c)** Image of the three-electrode bulk cell setup for O_2 bubbling within an Ar-filled glovebox, wherein the dashed arrows indicate the flow of O_2 gas into, and out of, the glass cell.

Electrochemical and spectroscopic characterisation of tethered RMs

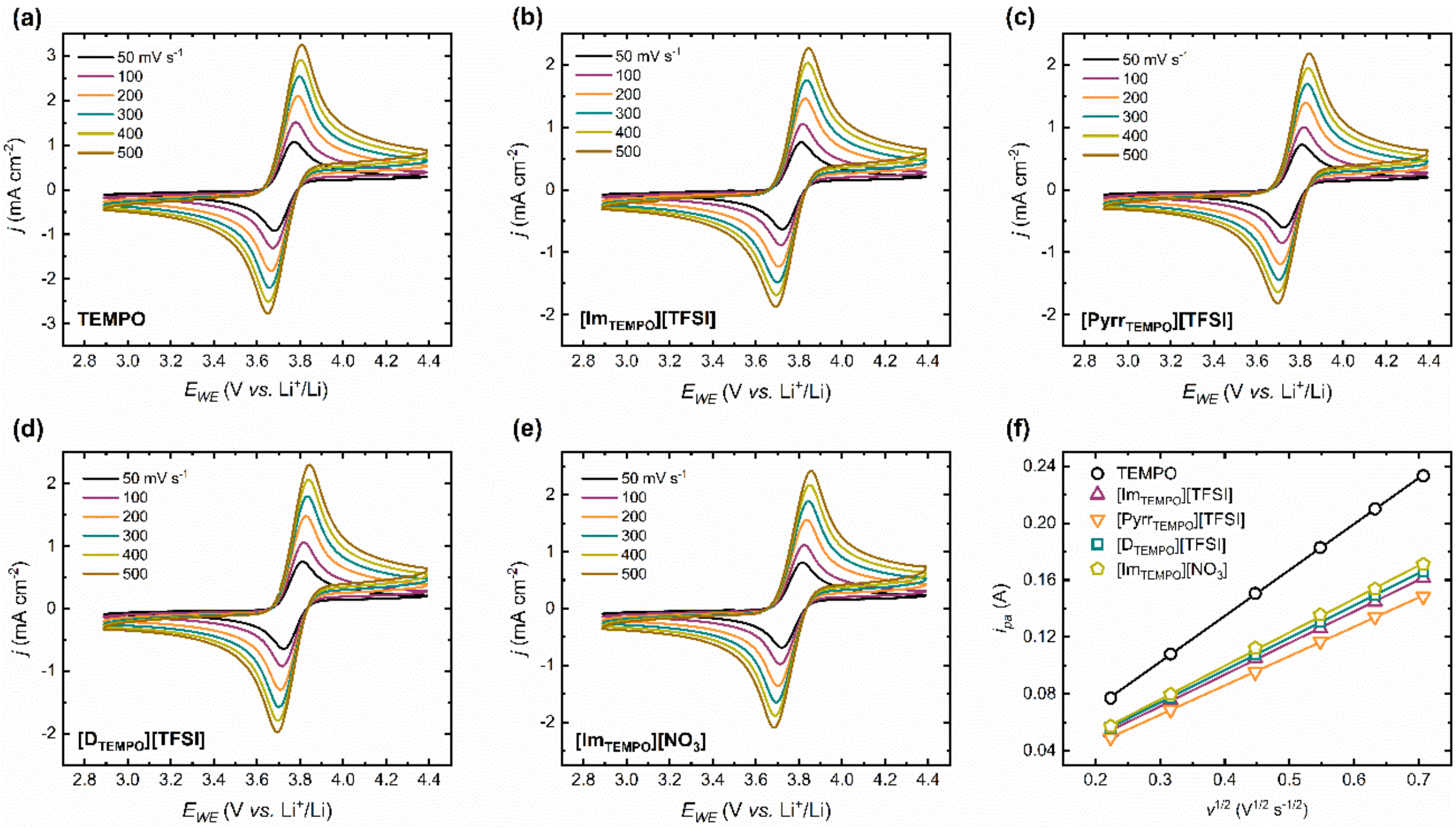

Figure S56 | CVs of 10 mM **(a)** TEMPO, **(b)** [Im_{TEMPO}][TFSI], **(c)** [$Pyrr_{TEMPO}$][TFSI], **(d)** [D_{TEMPO}][TFSI] and **(e)** [Im_{TEMPO}][NO_3] in 1 M Li[TFSI] in diglyme. **(f)** The relationship between peak anodic current and square root of the scan rate (R^2 >0.99 for all linear fits) to determine diffusion coefficients using the Randles-Sevcik equation (**Eq. 1**). The CVs were conducted at scan rates between 50 and 500 mV s^{-1} under Ar in a three-electrode glass cell comprising a glassy carbon WE, Pt CE and Ag[OTf]/Ag RE. Cell assembly and conversion of potentials from the Ag[OTf]/Ag scale to the Li^+/Li scale are described in the Experimental section.

$$i_p = 0.446\mathrm{n}^{\frac{3}{2}}\mathrm{F}^{\frac{3}{2}}\mathrm{AC_A}\left(\frac{v\mathrm{D_A}}{\mathrm{RT}}\right)^{\frac{1}{2}} \qquad \textbf{Eq. 1}$$

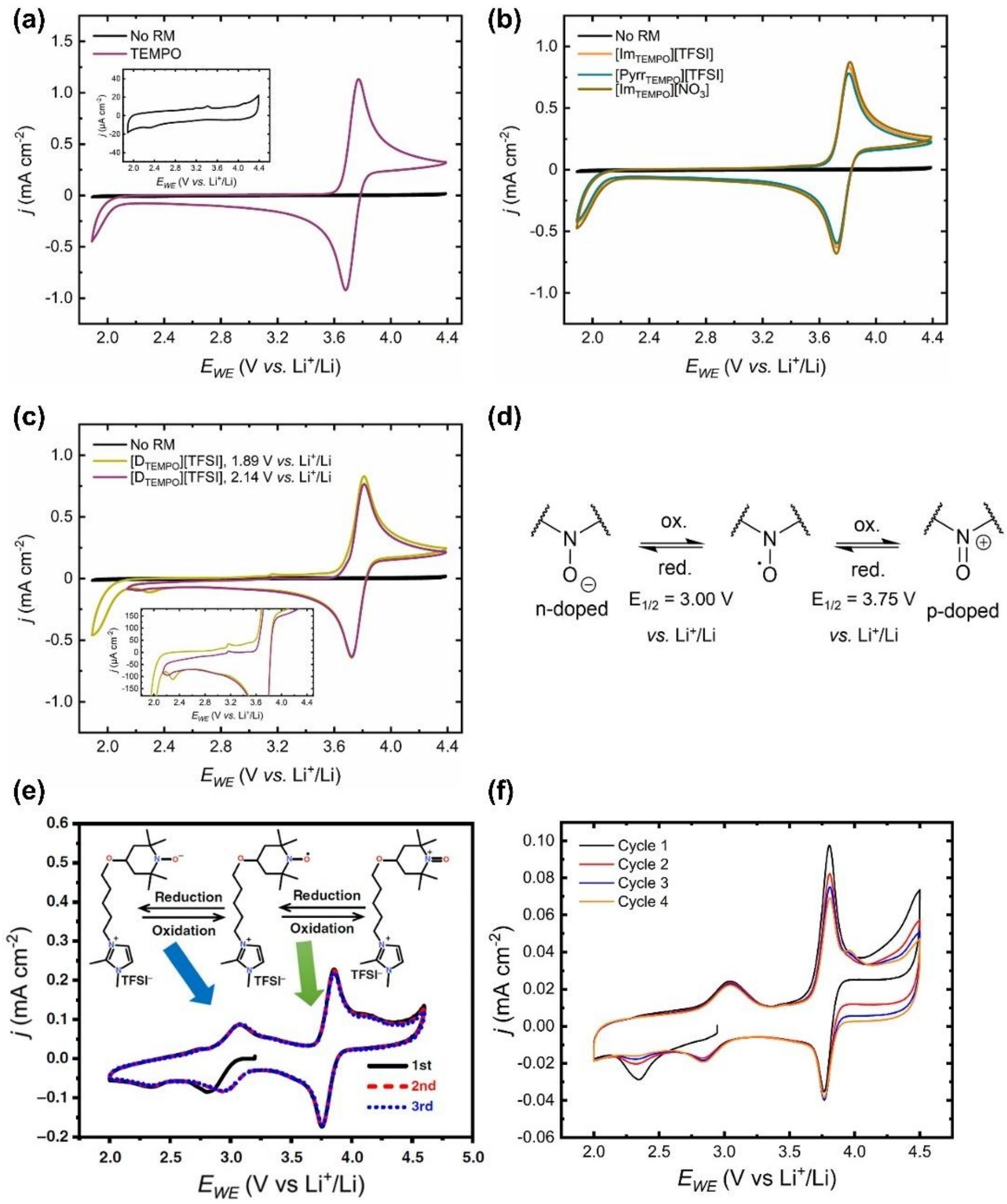

Figure S57 | CVs of 10 mM **(a)** TEMPO, **(b)** [Im$_{TEMPO}$][TFSI], [Pyrr$_{TEMPO}$][TFSI], [Im$_{TEMPO}$][NO$_3$] and **(c)** [D$_{TEMPO}$][TFSI] in 1 M Li[TFSI] in diglyme within a potential window of 1.9 to 4.4 V *vs.* Li^+/Li for **(a)** – **(c)**, and an additional window of 2.1 to 4.4 V *vs.* Li^+/Li for **(c)**. An inset CV of the base electrolyte without a RM (1 M Li[TFSI] in diglyme) is shown in black in **(a)** – **(c)**. A zoomed in view of this CV is provided in the inset in **(a)**, and a zoomed in view of the peaks in the CV of [D$_{TEMPO}$][TFSI] is shown as an inset in **(c)**. **(d)** Redox chemistry of the nitroxyl radical where Zhang *et al.*[3] showed that the reduction of nitroxyl radical to the aminoxy anion is reversible in [Im$_{TEMPO}$][TFSI]. The CVs were conducted at scan rate of 50 mV s^{-1} under Ar in a three-electrode glass cell comprising a glassy carbon WE, Pt CE and Ag[OTf]/Ag RE. Cell assembly and conversion of potentials from the Ag[OTf]/Ag scale to the Li^+/Li scale are as in the ''Cyclic Voltammetry'' section of the supplementary information. **(e, f)** CVs of 10 mM [Im$_{TEMPO}$][TFSI] in 0.5 M Li[TFSI] in diglyme at 0.5 mV s^{-1} conducted in a two-electrode Swagelok-type cell with a carbon paper WE and Li metal RE and CE electrode **(e)** as reported by Zhang *et al.*[3] and **(f)** replicated in this work. The potential window was 2.0 to 4.5 V *vs.* Li^+/Li.

Supporting Discussion 1

There are distinct differences in cell setup between the CVs shown in **Figure S57a-c** and those carried out by Zhang *et al.*[3] (**Figure S57e**). In the latter, CVs were performed in a two-electrode Swagelok-type cell with carbon paper as the WE and Li metal as the CE and RE. Therefore, this CV cell configuration was replicated in this work for [Im_{TEMPO}][TFSI] and the results are presented in **Figure S57f**. In this two-electrode Swagelok configuration the CVs of [Im_{TEMPO}][TFSI] reported by Zhang *et al.*[3] and those in this work exhibit the same peaks. They reported the TEMPO/$TEMPO^-$ redox couple at 3.00 V *vs.* Li^+/Li (**Figure S57e**), which is consistent with a redox process with $E_{1/2}$ = 2.94 V *vs.* Li^+/Li in this work (**Figure S57f**). There are additional reduction and oxidation peaks at ~2.3 and ~3.9 V *vs.* Li^+/Li, respectively, which are also present in the CV reported by Zhang and co-workers. There are a number of differences between the three-electrode glass cell and the two-electrode Swagelok cell experiments; (i) Li[TFSI] concentrations (1.0 M (glass cell), 0.5 M (Swagelok cell)), (ii) scan rate (50 mV s^{-1} (glass cell), 0.5 mV s^{-1} (Swagelok cell)) and (iii) different RE and WE. However, the accessibility of this lower potential redox process (TEMPO/$TEMPO^-$) in Li-O_2 cells is questionable. A potential plateau during galvanostatic cell charging is observed at the potential corresponding to TEMPO oxidation to $TEMPO^+$, but such a plateau is not observed on discharge for the reduction of TEMPO to $TEMPO^-$. Furthermore, In the study by Bergner *et al.*[10] that initially reported the use of TEMPO as a charge RM, they observed that CVs of TEMPO under Ar exhibited a reduction peak at ≤2.2 V *vs.* Li^+/Li and ascribed this peak to the irreversible reduction of TEMPO to $TEMPO^-$. Therefore, the reduction tail at ~1.9 V *vs.* Li^+/Li observed for all RMs in the glass cell in this work (**Figure S57(a)-(d)**) may be related to the irreversible reduction of TEMPO to $TEMPO^-$.

In the CVs of [D_{TEMPO}][TFSI] (**Figure S57c**), a reduction peak between 2.2 – 2.3 V *vs*. Li^+/Li is apparent. In the subsequent oxidation scan, a peak at ~3.2 V *vs.* Li^+/Li can be observed. However, this oxidation peak may be related to the reduction tail at 1.9 V *vs*. Li^+/Li, which is observed in CVs of all RMs. Therefore, the cathodic limit was set to 2.1 V *vs.* Li^+/Li (*i.e.*, just after the reduction peak at 2.2 – 2.3 V *vs*. Li^+/Li). Although the reduction peak has shifted slightly with this higher cathodic limit, the oxidation peak is still apparent, suggesting that these two peaks may be tied to the same process. Nonetheless, the peak separation is nearing 1 V and does not exhibit the degree of reversibility measured for the $TEMPO^+$/TEMPO couple. Therefore, it is concluded that the TEMPO/$TEMPO^-$ couple is also not accessible in [D_{TEMPO}][TFSI].

Table S4: Redox potentials ($E_{1/2}$) and diffusion coefficients (D) for TEMPO and the tethered RMs as determined by CV (Randles-Sevcik analysis), and the ratio of the diffusion coefficient of TEMPO (D_{TEMPO}) and the corresponding tethered RM ($D_{tethered}$).

	**TEMPO**	**[Im_{TEMPO}] [TFSI]**	**[$Pyrr_{TEMPO}$] [TFSI]**	**[D_{TEMPO}] [TFSI]**	**[Im_{TEMPO}] [NO_3]**
**$E_{1/2}$ (V)**	3.73<br>3.73[a]	3.77<br>3.75[b]	3.77	3.77	3.77
**D ($\times 10^{-6}$ cm^2 s^{-1})**	2.95<br>2.9[c]	1.39	1.20	1.47	1.57
**$D_{TEMPO}/D_{tethered}$**	-	2.12	2.46	2.01	1.88

[a]Determined in a 1 M Li[TFSI]/diglyme electrolyte.[11] [b]Determined in a 0.5 M Li[TFSI] in diglyme electrolyte.[3] [c]Determined in 0.1 M Li[TFSI] in tetraglyme at an ultramicroelectrode.[12]

Table S5 | Comparison of discharge and charge overpotentials (η_{dchg}, η_{chg}), terminal (dis)charge capacities ($Q_{T,\ (dis)charge}$), maximum discharge capacity ($Q_{max,\ dchg}$) and Li_2O_2 yield in Li-O_2 cells cycled with P50 carbon paper positive electrodes and either $Li_{1-x}FePO_4$ or Li metal negative electrodes at 0.1 mA cm^{-2}.

RM	[a]η_{dchg} (V vs. Li^+/Li)	[b]η_{chg} (V vs. Li^+/Li)	[c]$Q_{T,\ (dis)charge}$ cycle 10 (mAh cm^{-2})	[c]$Q_{T,\ (dis)charge}$ cycle 20 (mAh cm^{-2})	% change in $Q_{T,\ (dis)charge}$ from cycle 10 to 20	[d]$Q_{max,\ dchg}$ (mAh cm^{-2})	[e]% Li_2O_2 yield
No RM	0.34	N/A	*0.10* / **0.04**	*0.02* / **0.01**	*-80* / **-75**	0.67 ± 0.01	88.9 ± 0.8
TEMPO	0.37	0.75	*0.50* / **0.43**	*0.18* / **0.13**	*-64* / **-70**	0.84 ± 0.04	86.8 ± 0.8
[Im_{TEMPO}][TFSI]	0.35	0.84	*0.50* / **0.41**	*0.50* / **0.39**	*0* / **-5**	0.71 ± 0.03	85.6 ± 1.4
[$Pyrr_{TEMPO}$][TFSI]	0.35	0.85	*0.50* / **0.38**	*0.49* / **0.35**	*-2* / **-8**	0.92 ± 0.06	95.8 ± 0.5
[D_{TEMPO}][TFSI]	0.36	0.76	*0.50* / **0.40**	*0.28* / **0.18**	*-44* / **-55**	0.66 ± 0.02	94.8 ± 1.1
[Im_{TEMPO}][NO_3]	0.33	0.80	*0.50* / **0.42**	*0.50* / **0.43**	*0* / **+2**	1.95 ± 0.03	99.8 ± 0.1

[a]Reported for the first cycle at 50% of the discharge capacity limit (0.5 mAh cm^{-2}) in cells cycled with $Li_{1-x}FePO_4$ negative electrodes. The overpotential is calculated with respect to the thermodynamic formation/decomposition potential of Li_2O_2 (2.96 V *vs.* Li^+/Li).

[b]Reported for the first cycle at 50% of the charge capacity limit (0.5 mAh cm^{-2}) in cells cycled with $Li_{1-x}FePO_4$ negative electrodes. The overpotential is calculated with respect to the thermodynamic formation/decomposition potential of Li_2O_2 (2.96 V *vs.* Li^+/Li), which could not be calculated for the unmediated electrolyte due to a strong degree of polarisation.

[c]Terminal discharge capacities are shown in italics and terminal charge capacities in bold. The discharge and charge capacity limit was 0.5 mAh cm^{-2} and cells were cycled with $Li_{1-x}FePO_4$ negative electrodes.

[d]The maximum discharge capacity with a potential cut-off of 2.4 V *vs.* Li^+/Li in cells cycled with Li metal negative electrodes.

[e]Errors represent absolute uncertainty from duplicate measurements. Yields reported are for a single, capacity-limited discharge to 0.5 mAh cm^{-2} in cells with a Li metal negative electrode.

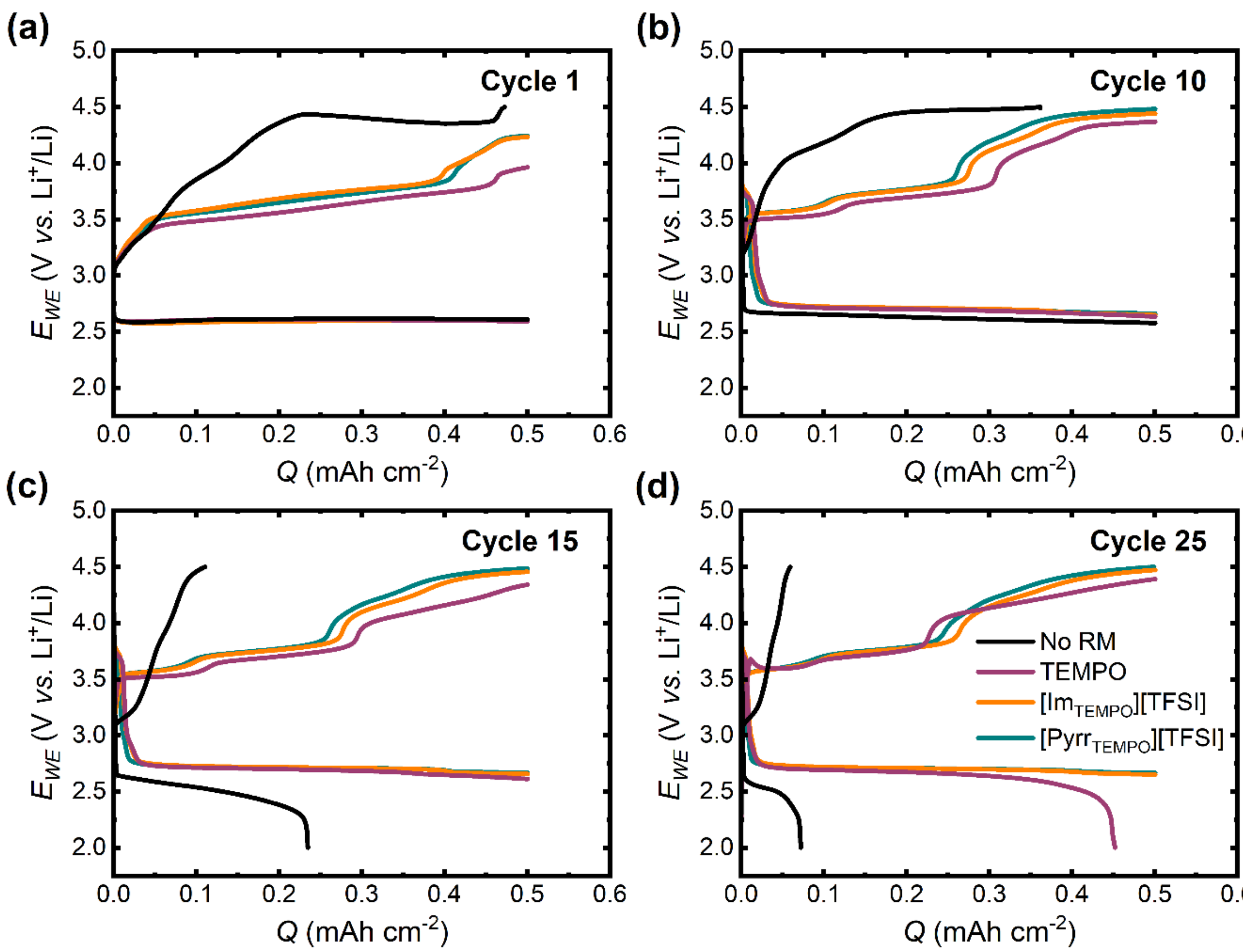

Figure S58 | Galvanostatic cycles **(a)** 1, **(b)** 10, **(c)** 15, **(d)** 25, **(e)** 30, **(f)** 35, **(g)** 40 and **(h)** 50 of standard Li-O_2 cells comprising a carbon paper positive electrode, Li metal negative electrode and an electrolyte without a RM and with either TEMPO, [Im_{TEMPO}][TFSI] or [$Pyrr_{TEMPO}$][TFSI] in 0.8 mol $kg_{solvent}^{-1}$ Li[TFSI] in diglyme. The RM loading was 25 mmol $kg_{solvent}^{-1}$. The capacity was limited to 0.5 mAh cm^{-2} and cells were cycled at a current density of 0.1 mA cm^{-2} with discharge and charge potential cut-offs of 2.0 and 4.5 V *vs*. Li^+/Li, respectively.

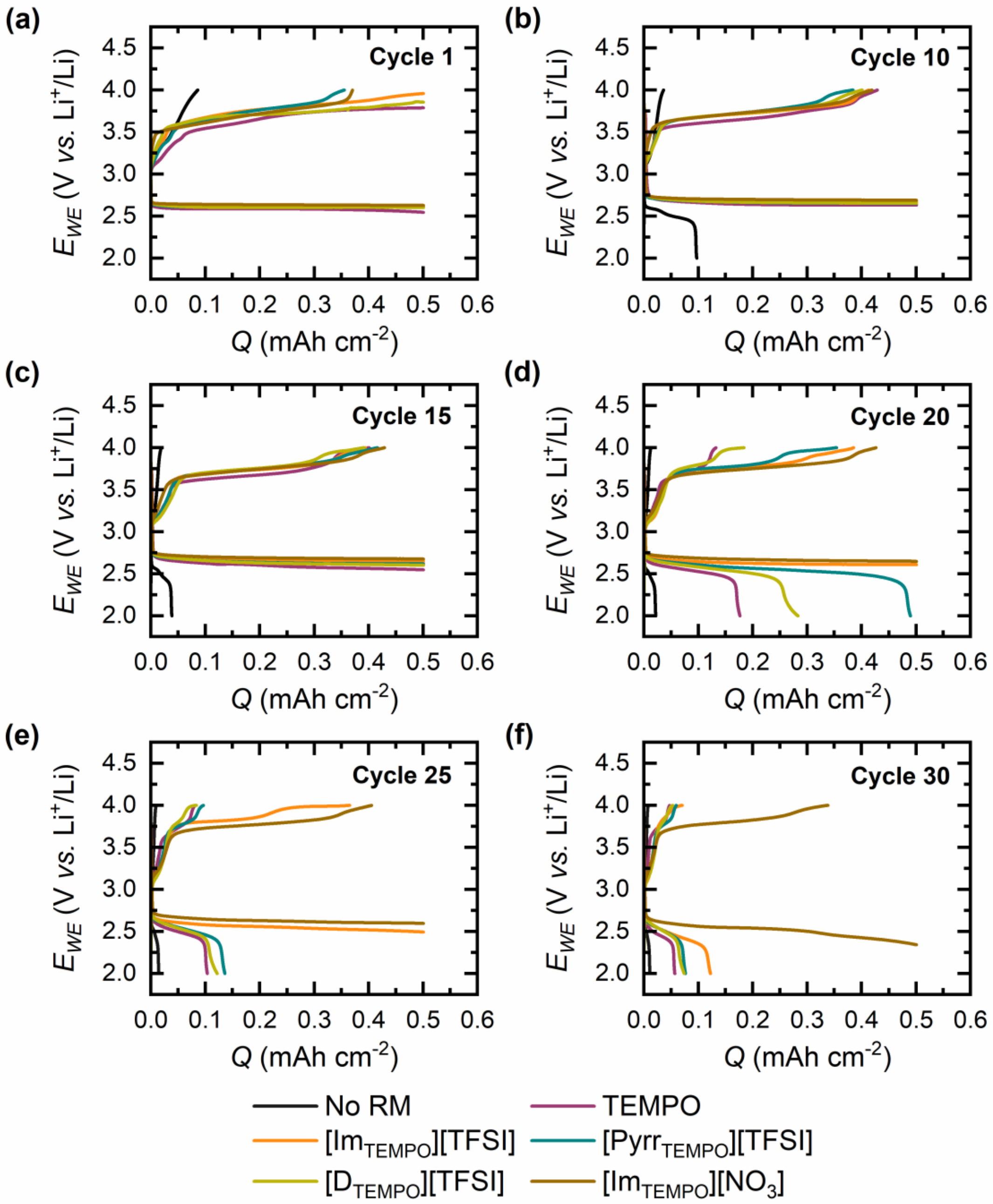

Figure S59 | Galvanostatic cycles **(a)** 1, **(b)** 10, **(c)** 15, **(d)** 20, **(e)** 25 and **(f)** 30 of standard Li-O_2 cells comprising a carbon paper positive electrode, $Li_{1-x}FePO_4$ negative electrode and an electrolyte without a RM and with either TEMPO, $[Im_{TEMPO}]$[TFSI], $[Pyrr_{TEMPO}]$[TFSI], $[D_{TEMPO}]$[TFSI] or $[Im_{TEMPO}][NO_3]$ in 0.8 mol $kg_{solvent}^{-1}$ Li[TFSI] in diglyme. The RM loading was 25 mmol $kg_{solvent}^{-1}$. The capacity was limited to 0.5 mAh cm^{-2} and cells were cycled at a current density of 0.1 mA cm^{-2} with discharge and charge potential cut-offs of 2.0 and 4.0 V *vs*. Li^+/Li, respectively.

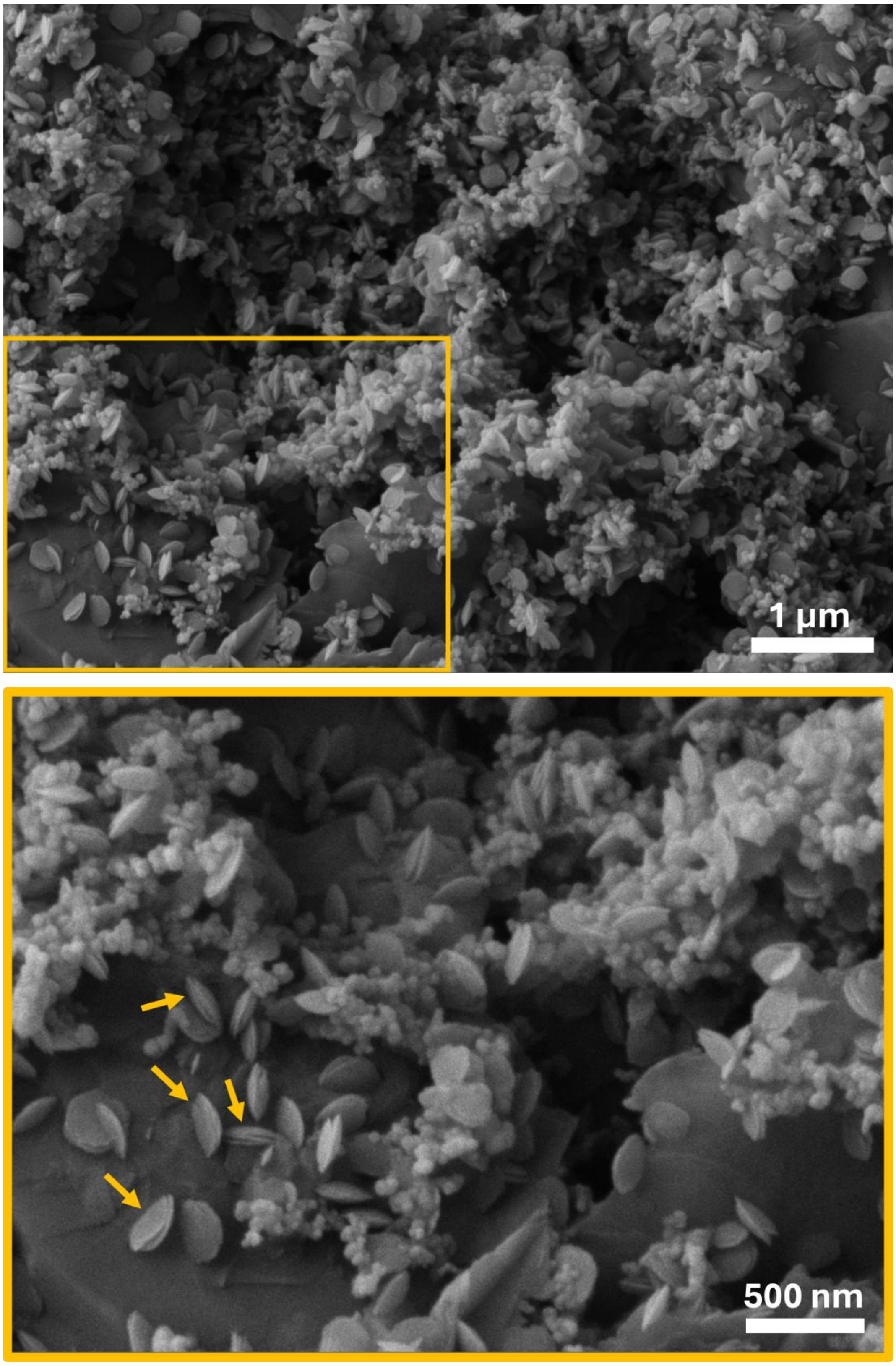

Figure S60 | Enlarged versions of the SEM micrographs shown in **Figure 4c, f** in the main article for carbon paper after a 0.5 mAh discharge at 0.1 mA cm^{-2} in 25 mmol $kg_{solvent}^{-1}$ [Im_{TEMPO}][NO_3] in 0.8 mol $kg_{solvent}^{-1}$ Li[TFSI] in diglyme at two different magnifications. The arrows highlight stacked platelet-like Li_2O_2 formed after discharge.

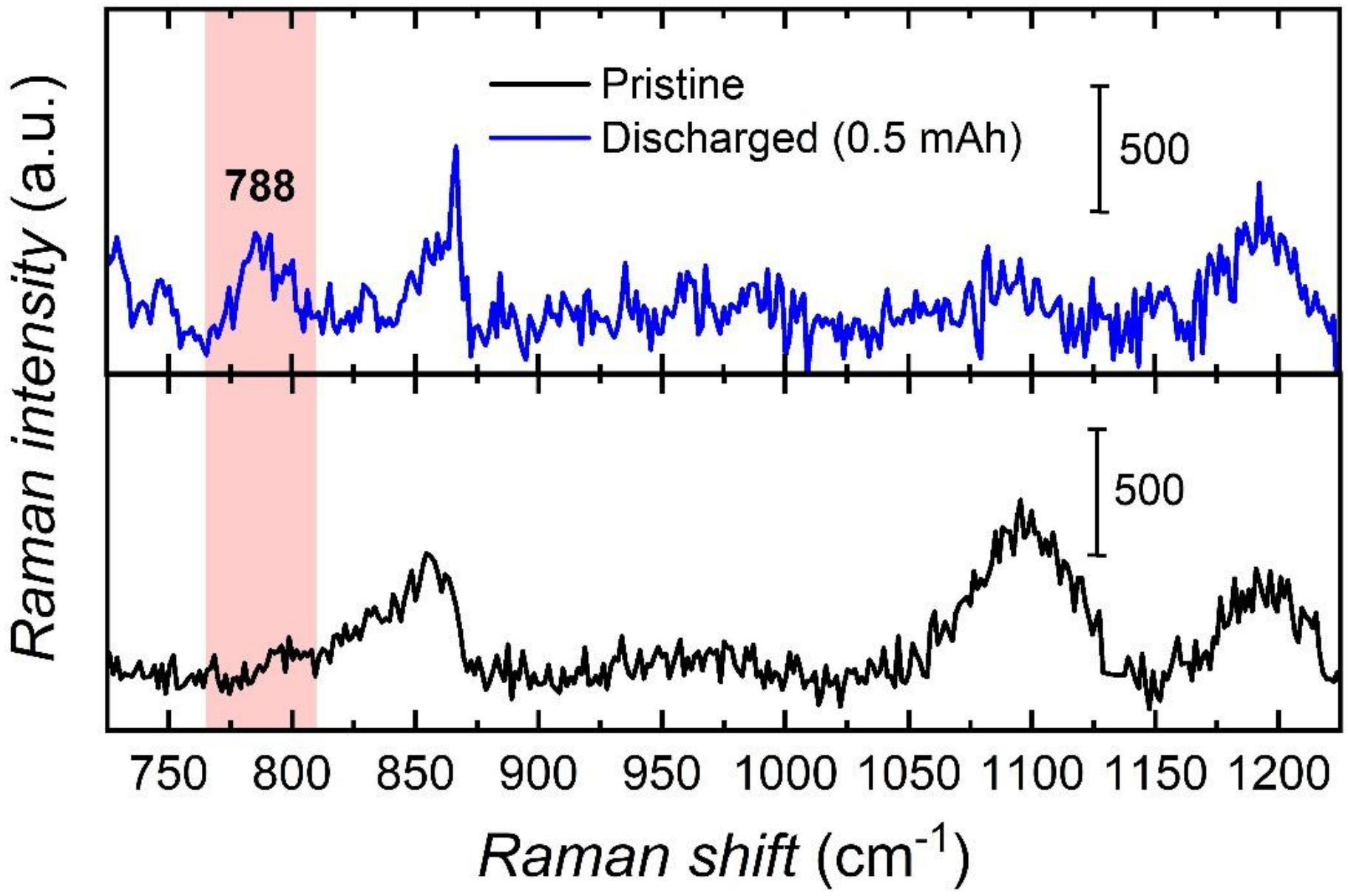

Figure S61 | Raman spectra of pristine and discharged P50 carbon paper electrodes. Discharge was performed in a standard Li-O_2 cell to 0.5 mAh with Li metal as the negative electrode. The electrolyte was 0.8 mol $kg_{solvent}^{-1}$ Li[TFSI] in diglyme with 25 mmol $kg_{solvent}^{-1}$ [Im_{TEMPO}][NO_3]. The red-shaded region highlights the Raman band of Li_2O_2 corresponding to the O-O stretching mode.[25]

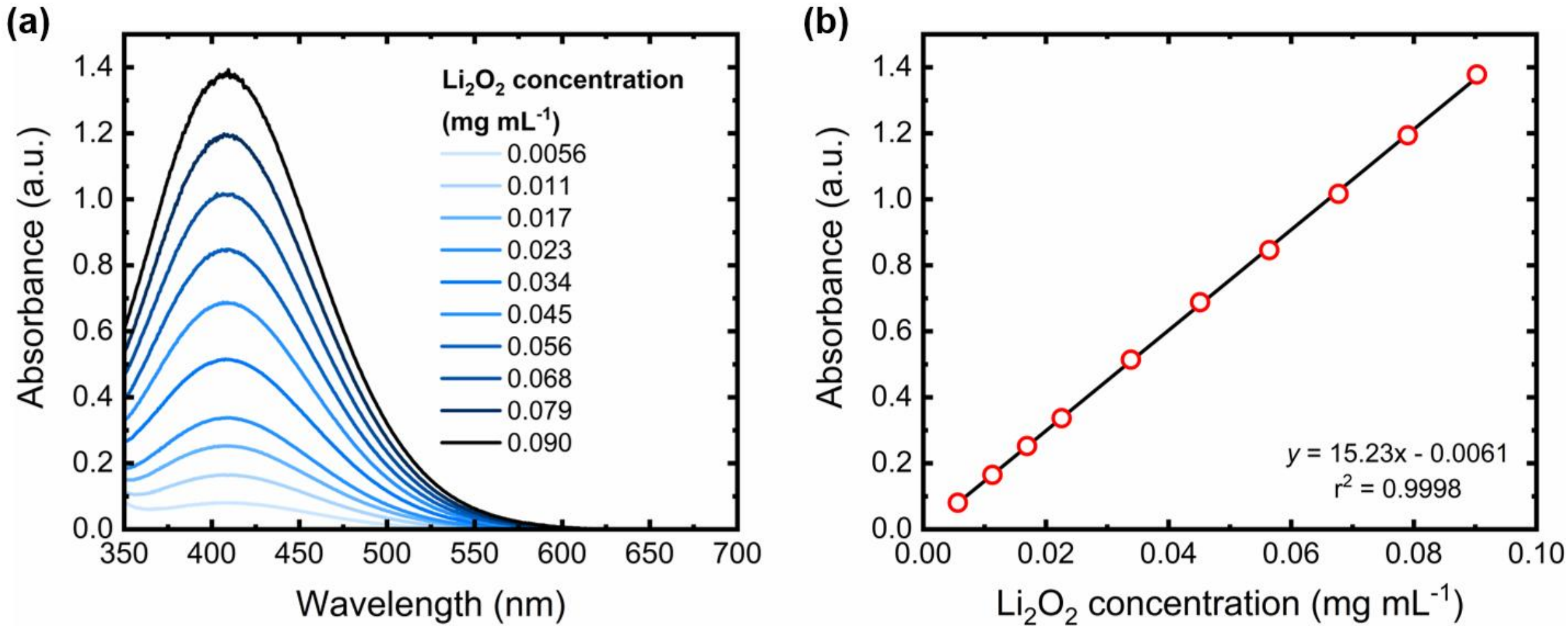

Figure S62 | (a) UV-Vis spectra of the Li_2O_2 calibration standards and **(b)** the corresponding calibration curve used for Li_2O_2 yield determination *via* UV-Vis spectroscopy. The calibration standards were prepared using known amounts of commercial Li_2O_2 (purity: 95%).

Table S6 | Comparison of discharge and charge overpotentials (η_{dchg}, η_{chg}), cycle life, Li_2O_2 yield, discharge capacity and RM diffusion coefficients for Li-O_2 cells cycled with tethered RMs and untethered nitroxide-based RMs.

RM	[a]η_{dchg} (V vs. Li^+/Li)	[a]η_{chg} (V vs. Li^+/Li)	Number of cycles	[b]% Li_2O_2 yield	[c]% change in discharge Q relative to unmediated electrolyte	Diffusion coefficient (x 10^{-6} cm^2 s^{-1})	Reference	Comments
TEMPO	0.37	0.64	8	86.8 ± 0.8	+25	2.95	This work	**+ve electrode:** Unmodified P50 carbon paper (Ø 10 mm)<br>**-ve electrode:** Untreated, bare Li metal<br>**Charge potential cut-off:** 4.0 V *vs* Li^+/Li<br>**Capacity limit:** 0.5 mAh cm^{-2} (based on geometric area of carbon paper electrode)<br>**Current density:** 0.1 mA cm^{-2} (based on geometric area of carbon paper electrode)<br>**RM concentration:** 25 mmol $kg_{solvent}^{-1}$ (*ca.* 20-25 mM)<br>**Base electrolyte:** 0.8 mol $kg_{solvent}^{-1}$ Li[TFSI] in diglyme (*ca.* 0.7 M)
[Im_{TEMPO}] [NO_3]	0.37	0.64	11	99.8 ± 0.1	+191	1.57		
[Im_{TEMPO}] [TFSI]	0.35	0.65	7	85.6 ± 1.4	+6	1.39		
TEMPO	0.28	0.58	50	N/A	-28	14[$]	10	**+ve electrode** Ketjenblack EC600JD/PTFE 75/25 wt% (Ø 10 mm, loading: 0.3 – 0.5 mg carbon)<br>**-ve electrode:** Li metal pre-treated in propylene carbonate<br>**Charge potential cut-off:** 4.5 V *vs.* Li^+/Li<br>**Capacity limit:** 1000 mAh g_c^{-1} (*ca.* 0.4 – 0.6 mAh cm^{-2})<br>**Current density:** 0.1 mA cm^{-2} (based on geometric area of carbon electrode, true areal current density (based on specific surface area (SSA)) ≪ geometric current density)<br>**RM concentration:** 10 mM<br>**Base electrolyte:** 0.1 M Li[TFSI] in diglyme
TEMPO	0.27	0.68	N/A	N/A	-30	3.9	11	**+ve electrode:** Ketjenblack/PTFE 87/13 wt% (Ø 10 mm, loading: 0.4 – 0.8 mg cm^{-2} carbon)<br>**-ve electrode:** Untreated, bare Li metal<br>**Charge potential cut-off:** 4.2 V *vs.* Li^+/Li<br>**Capacity limit:** N/A
4-methoxy-TEMPO	0.36	0.75	N/A	N/A	-28	3.3		
1-Me-AZADO	0.28	0.64	N/A	N/A	-2	3.9		

TMAO	0.28	0.68	N/A	N/A	-11	2.5		**Current density:** 0.1 mA cm^{-2} (based on geometric area of carbon electrode, true areal current density ≪ geometric current density)<br>**RM concentration:** 10 mM<br>**Base electrolyte:** 1 M Li[TFSI] in diglyme
[Im$_{TEMPO}$][TFSI]	0.30	0.58	20	92.7 ± 1.7	+3300	N/A	3	**+ve electrode** Stack of three carbon paper (H2315) electrodes (Ø 10 mm)<br>**-ve electrode** Untreated, bare Li metal<br>**Charge potential cut-off:** 4.6 V *vs.* Li$^+$/Li<br>**Capacity limit:** 0.25 mAh cm^{-2} (based on geometric area of carbon paper electrode)<br>**Current density:** 0.1 mA cm^{-2} (based on geometric area of carbon paper electrode)<br>**RM concentration:** 1% by vol. (*ca.* 25 mM)<br>**Base electrolyte:** 0.5 M Li[TFSI] in diglyme
[Im$_{TEMPO}$][TFSI]	0.49*	0.47*	15	N/A	N/A	N/A	1	**+ve electrode:** Carbon nanotube/PTFE 90/10 wt% (Ø 12 mm, loading: 0.5 mg cm^{-2})<br>**-ve electrode:** Untreated, bare Li metal<br>**Charge potential cut-off:** 4.2 V *vs.* Li$^+$/Li<br>**Capacity limit:** 0.13 mAh cm^{-2} (based on geometric area of carbon electrode)<br>**Current density:** 0.26 mA cm^{-2} (based on geometric area of carbon electrode, true areal current density ≪ geometric current density)<br>**RM concentration:** N/A<br>**Base electrolyte:** 0.5 M Li[TFSI] in [Im$_{TEMPO}$][TFSI] (RM used as solvent, cycling at 73 °C)
IL-PTZ	0.27	0.78	25	N/A	N/A	N/A	13	**+ve electrode:** Ketjenblack-based (loading: 4 mg cm^{-2})<br>**-ve electrode:** Untreated, bare Li metal<br>**Charge potential cut-off:** 4.3 V *vs.* Li$^+$/Li<br>**Capacity limit:** 1000 mAh g_c^{-1} (conversion to mAh cm^{-2} not possible as electrode area was not reported)<br>**Current density:** 0.1 mA cm^{-2} (based on geometric area of carbon electrode, true areal current density ≪ geometric current density)

								**RM concentration:** 100 mM<br>**Base electrolyte:** 1 M Li[TFSI] in tetraglyme
PDI-TEMPO	0.37	0.68	50	N/A	+4530	N/A	14	**+ve electrode:** Stack of three carbon paper electrodes (Ø 10 mm)<br>**-ve electrode** Li metal pre-treated in propylene carbonate with 5% fluoroethylene carbonate<br>**Charge potential cut-off:** 4.6 V *vs.* Li^+/Li<br>**Capacity limit:** 0.25 mAh cm^{-2} (based on geometric area carbon electrode)<br>**Current density:** 0.1 mA cm^{-2} (based on geometric area carbon electrode)<br>**RM concentration:** 5 mM<br>**Base electrolyte:** 0.5 M Li[TFSI] in diglyme

[a]Reported for the first cycle at 50% of the total (dis)charge capacity unless noted otherwise. The overpotential is calculated with respect to the thermodynamic formation/decomposition potential of Li_2O_2 (2.96 V *vs.* Li^+/Li).

[b]Errors represent absolute uncertainty from at least duplicate measurements. Yields reported in this work are for a single, capacity-limited discharge to 0.5 mAh cm^{-2} in cells with a Li metal negative electrode. Yields quoted in other works did not report the capacity cells were discharged to.

[c]Percent change in discharge capacity with a redox mediator compared to the unmediated case in the identical cell configuration *within* each study. *Between* studies, various positive electrodes (carbon paper, carbon nanotubes, carbon black) have been used and do not always correspond to the cell configurations and conditions detailed in the "Comments" column (*e.g.*, some studies used multiple types of positive electrodes, but, in the interest of as fair a comparison as possible, all other metrics (η_{dchg}, η_{chg}, number of cycles and Li_2O_2 yield) have been extracted for the configuration that is most similar to the cell configuration reported in this work).

*Terminal capacities as discharge-charge cycling profiles were not provided.

[$]The diffusion coefficient was determined by chronoamperometry using the Cottrell equation in a 0.1 M Li[TFSI]/diglyme electrolyte. This is different to the Randles-Sevcik analysis used to determine diffusion coefficients of RMs in this work, notably in a 1 M Li[TFSI]/diglyme electrolyte. We attribute the discrepancy between the values to these differences between the two studies.

N/A denotes that the metric is either not reported or cannot be calculated as the required data has not been reported.

Abbreviations: 1-methyl-2-azaadamantane-N-oxyl (1-Me-AZADO), 1,1,3,3-tetramethyl-2,3-dihydro-2-azaphenalene-2-yloxyl (TMAO), ammonium-based ionic liquid (IL) with phenothiazine (PTZ) tethered to the ammonium cation as the redox moiety (IL-PTZ) and N,N′-di-(2,2,6,6-tetramethyl-1-oxyl-4-piperidinyl) perylene-3,4,9,10-tetracarboxylic diimide (PDI-TEMPO).

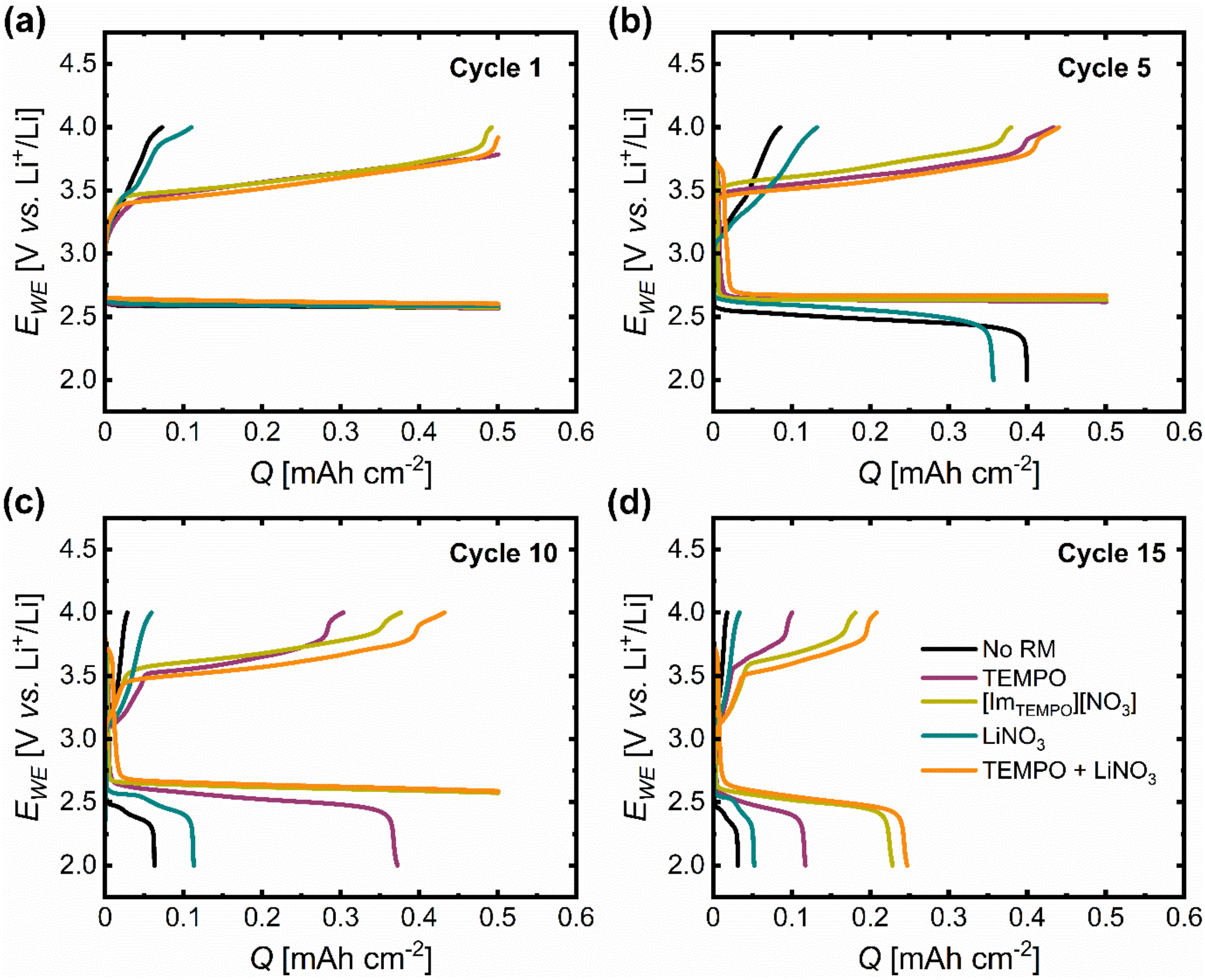

Figure S63 | Galvanostatic cycles **(a)** 1, **(b)** 5, **(c)** 10 and **(d)** 15 of standard Li-O_2 cells comprising a carbon paper positive electrode, Li metal negative electrode and an electrolyte without a RM and with either TEMPO, [Im_{TEMPO}][NO_3], Li[NO_3] or TEMPO + Li[NO_3] in 0.8 mol $kg_{solvent}^{-1}$ Li[TFSI] in diglyme. The RM loading was 25 mmol $kg_{solvent}^{-1}$ (where TEMPO and Li[NO_3] were used in the same electrolyte, the electrolyte comprised of 25 mmol $kg_{solvent}^{-1}$ of each additive). The capacity was limited to 0.5 mAh cm^{-2} and cells were cycled at a current density of 0.1 mA cm^{-2} with discharge and charge potential cut-offs of 2.0 and 4.0 V *vs*. Li^+/Li, respectively.

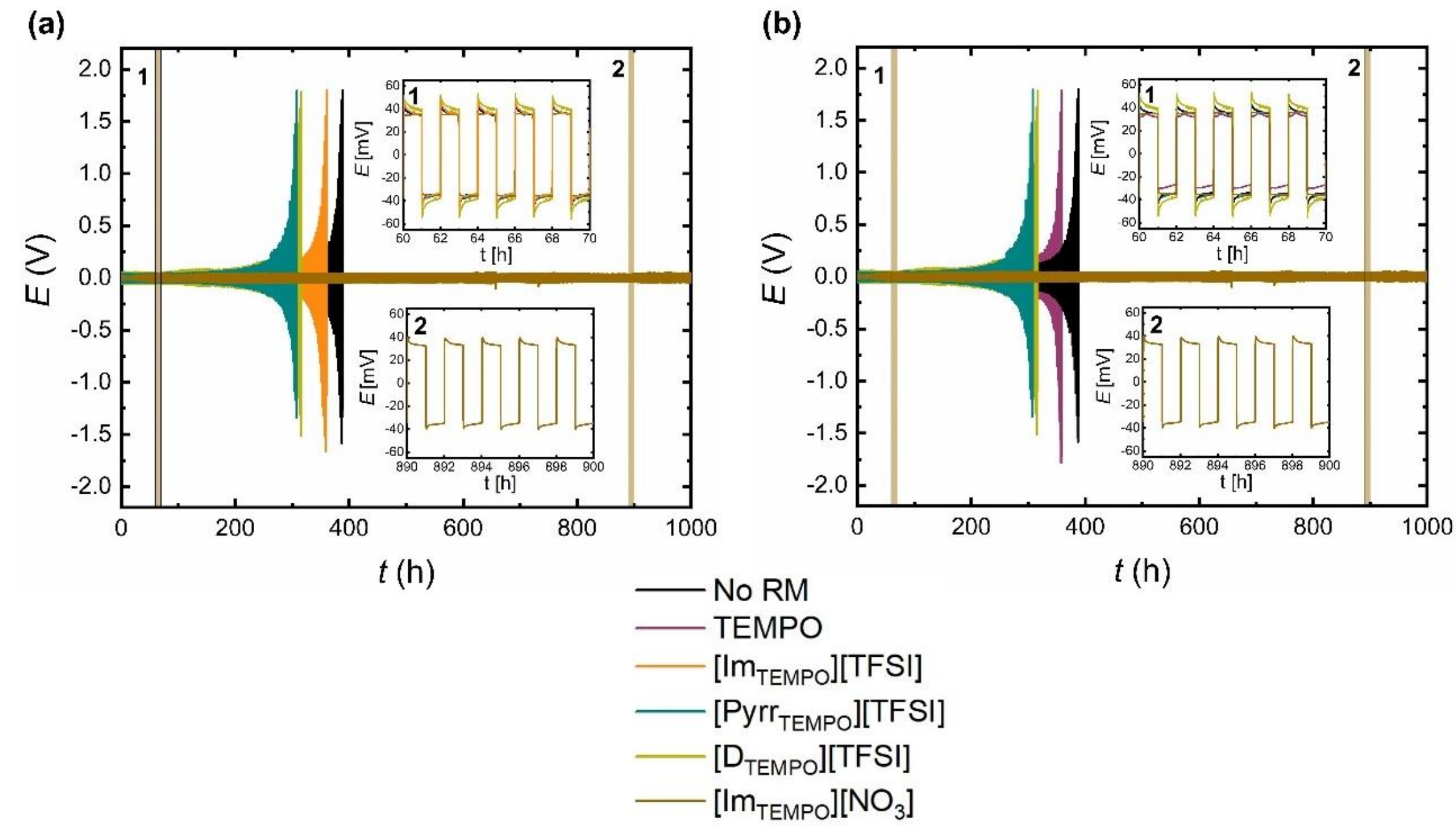

Figure S64 | Li|Li symmetric coin cell cycling with 0.8 mol kg^{-1} Li[TFSI] (black) containing TEMPO (purple), [Im$_{TEMPO}$][TFSI] (orange), [Pyrr$_{TEMPO}$][TFSI] (teal), [D$_{TEMPO}$][TFSI] (olive) and [Im$_{TEMPO}$][NO_3] (brown). Cells were cycled at 0.1 mA cm^{-2} and each discharge/charge half-cycle was 1 h. Note that cells with [Im$_{TEMPO}$][TFSI] in **(a)** and TEMPO in **(b)** fail at nearly identical times, so the corresponding cycling profiles have been plotted on separate panels for clarity.

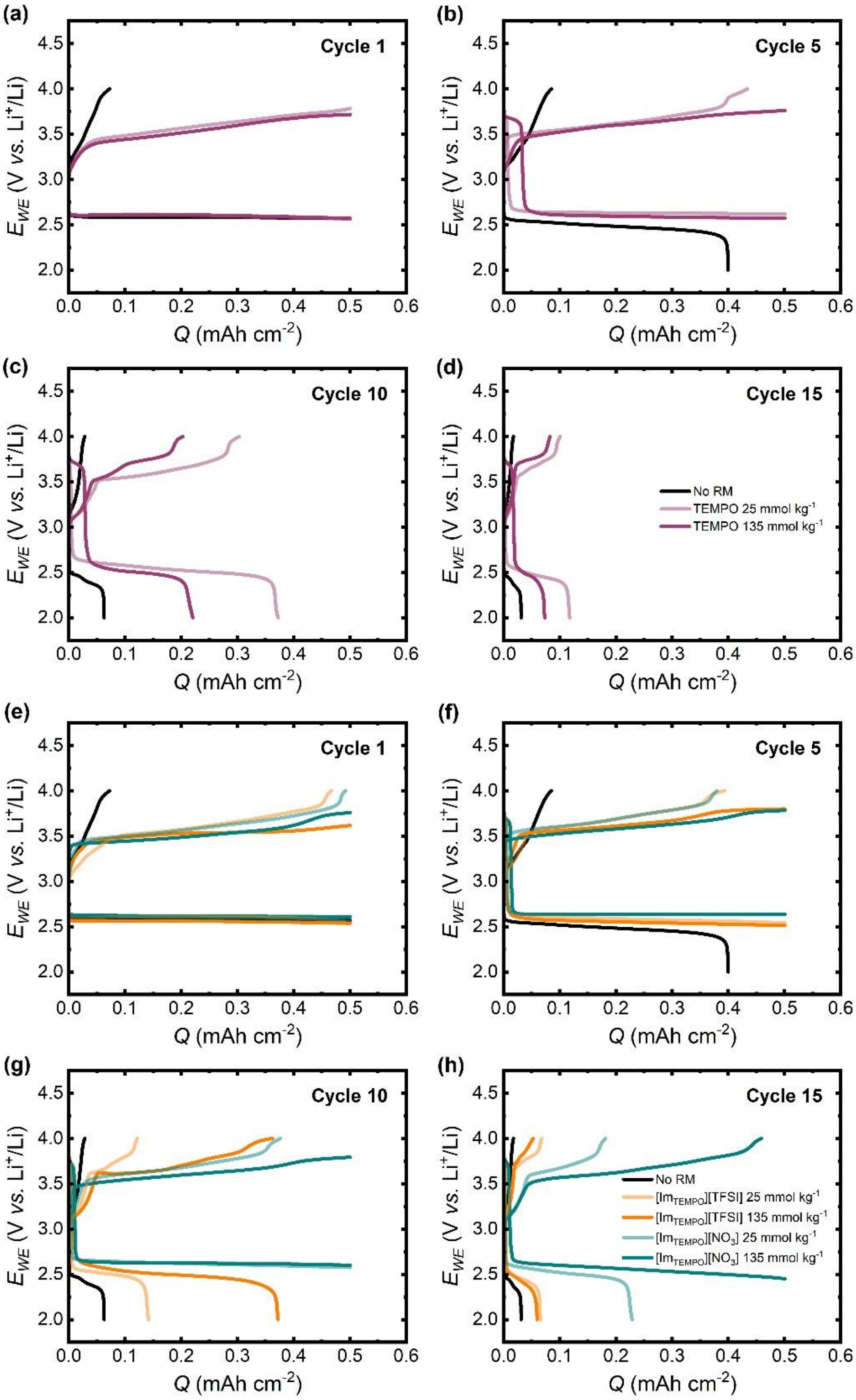

Figure S65 | Galvanostatic cycles **(a, e)** 1, **(b, f)** 5, **(c, g)** 10 and **(d, h)** 15 of standard Li-O_2 cells comprising a carbon paper positive electrode, Li metal negative electrode and an electrolyte without a RM and with either **(a-d)** TEMPO or **(e-h)** the tethered RMs [Im_{TEMPO}][TFSI] or [Im_{TEMPO}][NO_3] in 0.8 mol $kg_{solvent}^{-1}$ Li[TFSI] in diglyme. The RM loading was either 25 or 135 mmol $kg_{solvent}^{-1}$. The capacity was limited to 0.5 mAh cm^{-2} and cells were cycled at a current density of 0.1 mA cm^{-2} with discharge and charge potential cut-offs of 2.0 and 4.0 V *vs*. Li^+/Li, respectively.

Table S7 | Comparison of cell components and cycling parameters for Li-O_2 cells cycled with various multifunctional RMs reported in the literature and those reported in this work.

Reference	RM	[RM] (mM)	+ve electrode	+ve electrode **diameter** (mm) /*loading* (mg)	-ve electrode	Capacity limit		Current density		Charge potential limit (V *vs.* Li^+/Li)	Base electrolyte
						mAh g^{-1}	[a]mAh cm^{-2}	mA g^{-1}	[a]mA cm^{-2}		
This work	TEMPO/ Tethered TEMPO	~25	P50 carbon paper	**10** / *(N/A)*	Li metal or $Li_{1-x}FePO_4$	N/A	0.50	N/A	0.10	4.0	~0.7 M Li[TFSI] in diglyme
15	DMII	100	CB/PVDF (90/10 wt%)	**16** / *0.1*	Li metal	1000	0.05	500	0.02	4.5	1 M Li[TFSI] in diglyme
16	DMPII	100	SP/PVDF (90/10 wt%)	**12** / *0.5*	Li metal	500	0.22	200	0.09	4.0	1 M Li[TFSI] in tetraglyme
17	ATCI	50	SP or graphene/ PVDF (90/10 wt%)	**10** / *0.2-0.4*	Li metal	500	0.13-0.25	500	0.13-0.25	N/A	1 M Li[ClO_4] in DMSO
18	InI_3	16.7	MWCNTs/ PTFE (90/10 wt%)	**7** / *0.2*	Li metal	1000	0.50	500	0.26	N/A	0.5 M Li[ClO_4] in DMSO
19	MPPBr	200	SP/PTFE (80/20 wt%)	**12** / *0.3-0.5*	Li metal	1000	0.3-0.4	500	0.13-0.22	N/A	1 M Li[TFSI] in tetraglyme
20	$PP_{13}Br$	100	MWCNTs/ single layer graphene/ PVDF (80/10/10 wt%)	**12** / *0.1*	Li metal	1000	0.09	1000	0.09	N/A	1 M Li[TFSI] in tetraglyme
21	ABTS	N/A	SP/PVDF (90/10 wt%)	**10** / *(N/A)*	Li metal	500	N/A	500	N/A	N/A	1 M Li[ClO_4] in DMSO

22	BHT	N/A	CNT film	**12** / *0.75*	Li metal	500	0.33	151	0.1	4.5	1 M Li[OTf] in tetraglyme
23	DBDMB	5	SP/PVDF (90/10 wt%)	**N/A** / *(N/A)*	Li metal pre-treated in propylene carbonate	1000	N/A	1000	N/A	N/A	1 M Li[OTf] in tetraglyme
24	$Ir(acac)_3$	10	KB/PVDF (90/10 wt%)	**N/A** / *(N/A)*	Li metal	500	N/A	100	N/A	N/A	1 M Li[TFSI] in tetraglyme

[a]Normalised by geometric area of positive electrode.

N/A denotes "not applicable" or that the parameter information was not provided.

Abbreviations: carbon black (CB, used when specific type of carbon black was not specified), Super P (SP), multi-walled carbon nanotubes (MWCNTs), carbon nanotubes (CNTs), Ketjenblack (KB), polyvinylidene fluoride (PVDF), polytetrafluoroethylene (PTFE), 1,3-dimethylimidazolium iodide (DMII), 1,2-dimethyl-3-propylimidazolium iodide (DMPII), acetylcholine iodide (ATCI), N-Methyl-N-propylpyrrolidinebromide (MPPBr), 1-methyl-1-propylpiperidinium bromide ($PP_{13}Br$), 2,2'-azino bis(3-ethylbenzothiazoline-6-sulfonic acid) diammonium salt (ABTS), butylated hydroxytoluene (BHT), 2,5-di-tert-butyl-1,4-dimethoxybenzene (DBDMB) and iridium acetylacetonate ($Ir(acac)_3$).

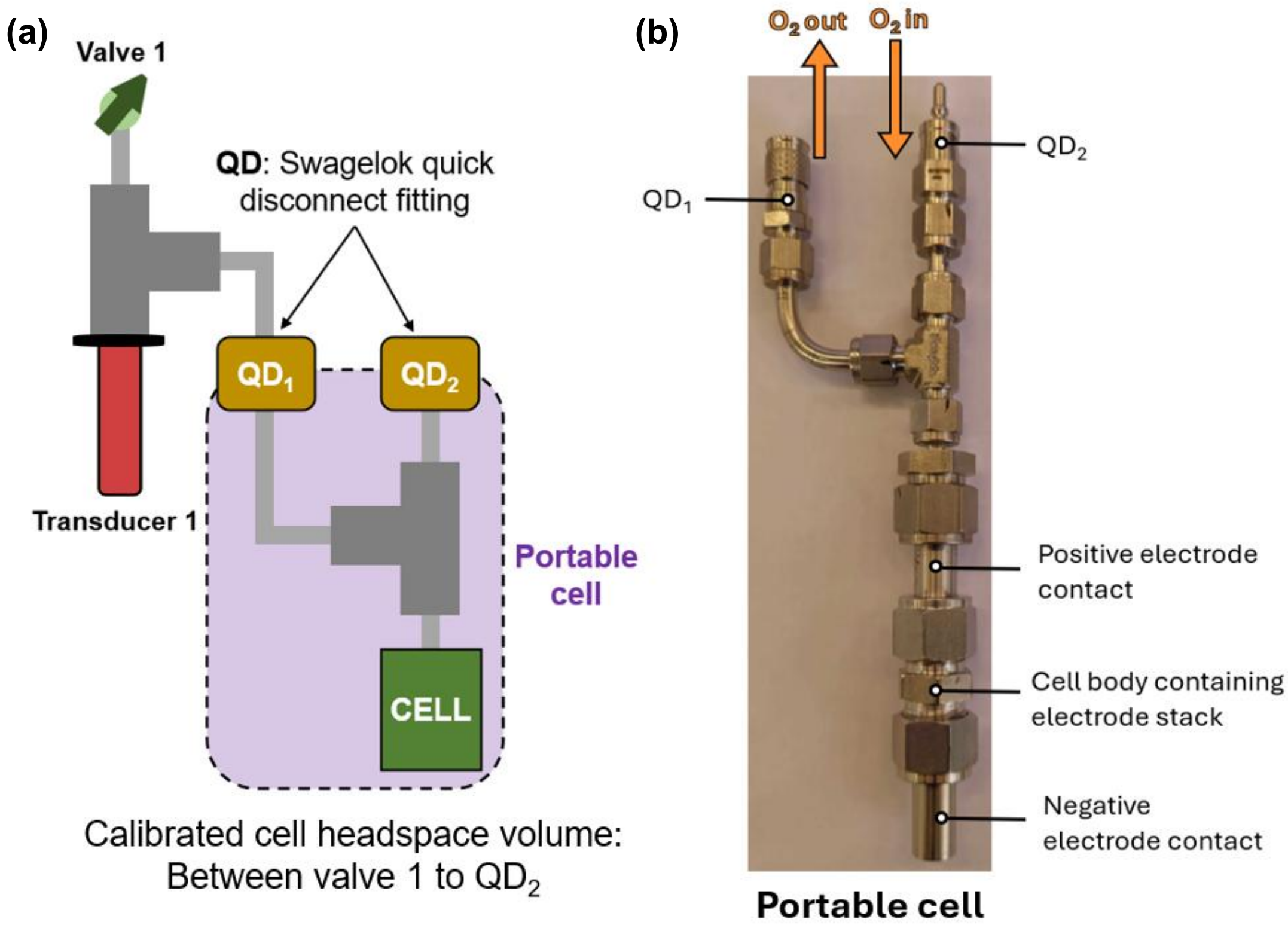

Figure S66 | (a) Schematic of the operando pressure electrochemical cell connected to a pressure transducer *via* a quick disconnect fitting (QD_1) highlighting the portable cell portion. **(b)** An image of the portable cell showing the direction of O_2 flow into and out of the cell. The cell body containing the electrode stack has an internal lining made of polyether ether ketone (PEEK) to prevent short circuiting. The cell headspace is the volume between QD_2 and valve 1. Details regarding calibration of the cell volume and setup have been described in a previous publication.[26]

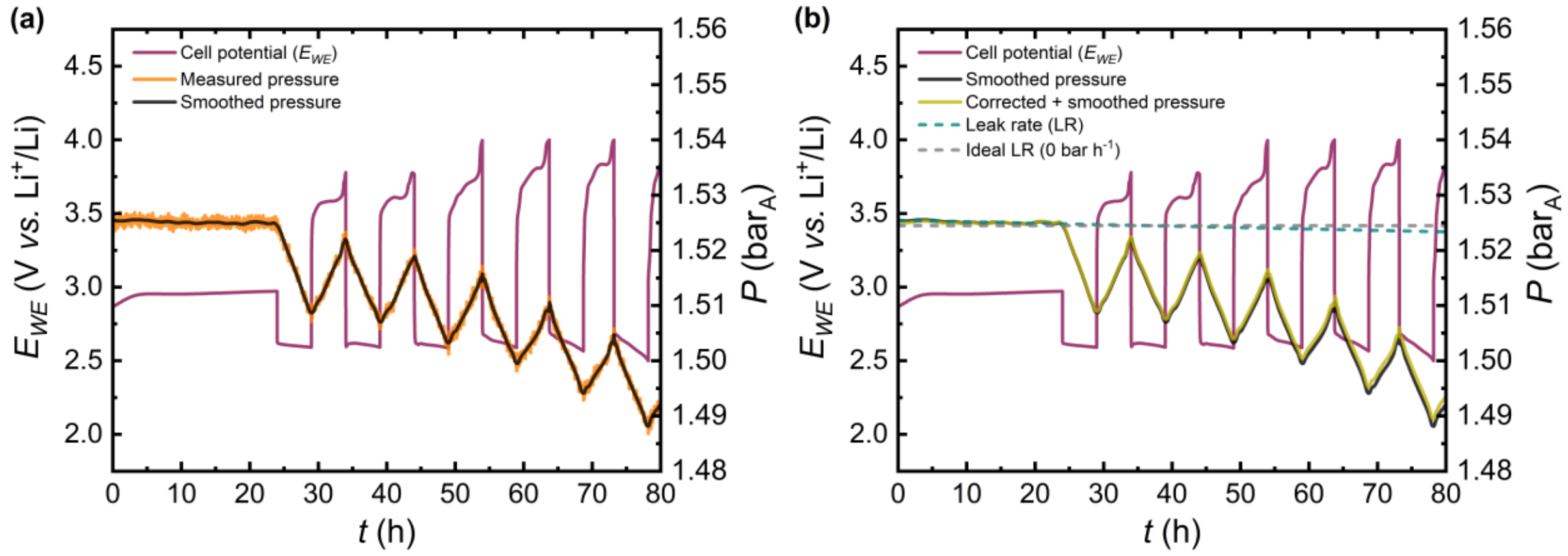

Figure S67 | Example graphs to illustrate the typical data processing procedure for pressure cell data (electrolyte: 25 mmol $kg_{solvent}^{-1}$ TEMPO in 0.8 mol $kg_{solvent}^{-1}$ Li[TFSI] in diglyme). The cell potential (purple) and smoothed pressure (black) are shown as a function of time with **(a)** the measured (raw) pressure reading (orange) and **(b)** the corrected and smoothed pressure (olive), leak rate (cyan, dotted) and ideal leak rate (grey, dotted). The ideal leak rate (i.e. 0 bar h^{-1}) assumes no leaking and is fixed at the pressure at the start of discharge. The leak rate measured over the initial *ca.* 24 h rest period, typically within (3.4 ± 2.3) x 10^{-5} bar h^{-1}, is used to correct the pressure response to account only for pressure changes arising from electrochemical processes. Within this example, the leak rate, defined by the pressure drop over the 24 h open circuit (rest) period, was 2.6 x 10^{-5} bar h^{-1}.

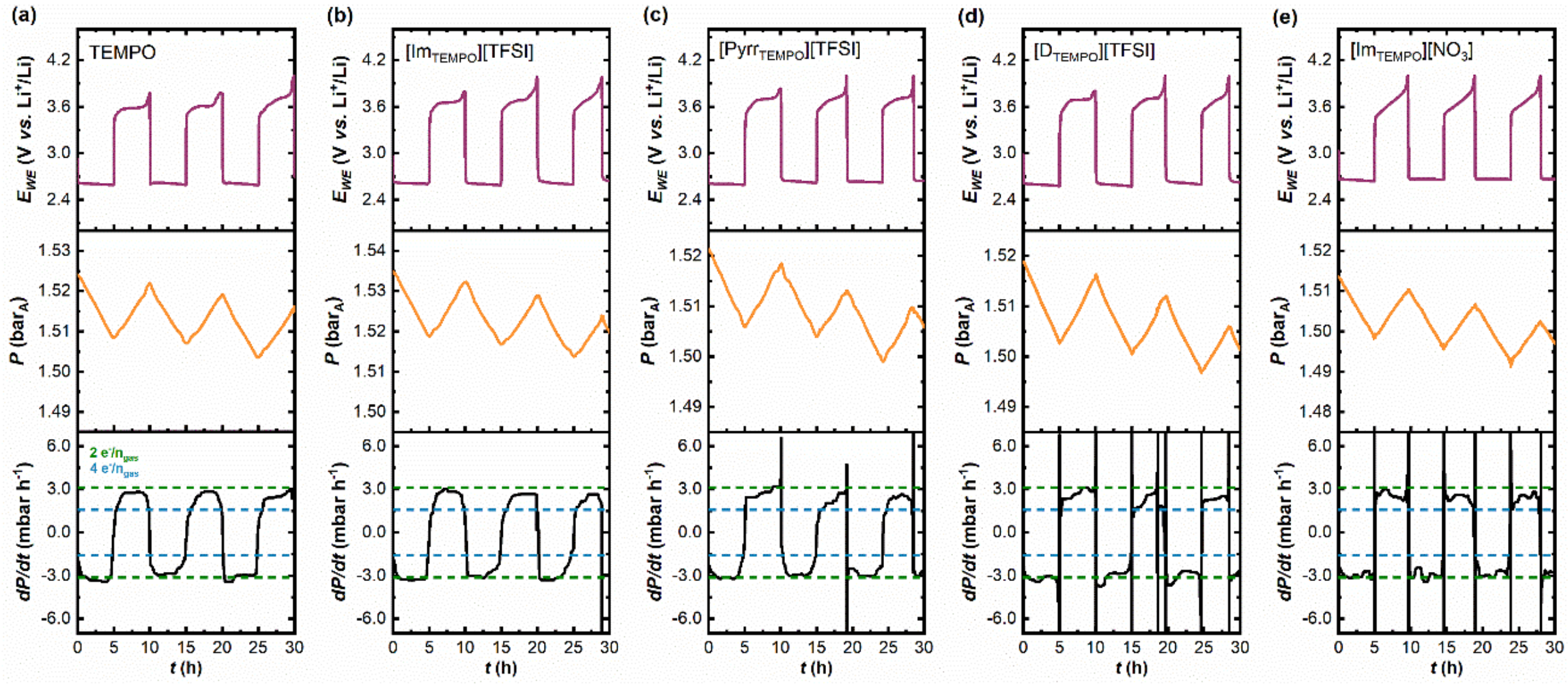

Figure S68 | Cycles 1-3 in pressure cells cycled under a capacity-limited (0.5 mAh cm^{-2}) regime at 0.1 mA cm^{-2} in 0.8 mol $kg_{solvent}^{-1}$ Li[TFSI] in diglyme electrolytes with **(a)** TEMPO, **(b)** [Im_{TEMPO}][TFSI], **(c)** [$Pyrr_{TEMPO}$][TFSI], **(d)** [D_{TEMPO}][TFSI] and **(e)** [Im_{TEMPO}][NO_3]. The RM loading was 25 mmol $kg_{solvent}^{-1}$. The cell potential (purple), pressure response (orange) and 1st derivative of the pressure response (black) are shown as a function of time. The dotted green and blue lines indicate the theoretical gas consumption/evolution rates for $2e^-/n_{gas}$ and $4e^-/n_{gas}$ processes, respectively. The average gas leak rate over a *ca.* 24 h rest period at open circuit potential was (3.4 ± 2.3) x 10^{-5} bar h^{-1}. The leak rate for each cell assembled was determined and used to correct the measured pressure response, as described in **Figure S67**.

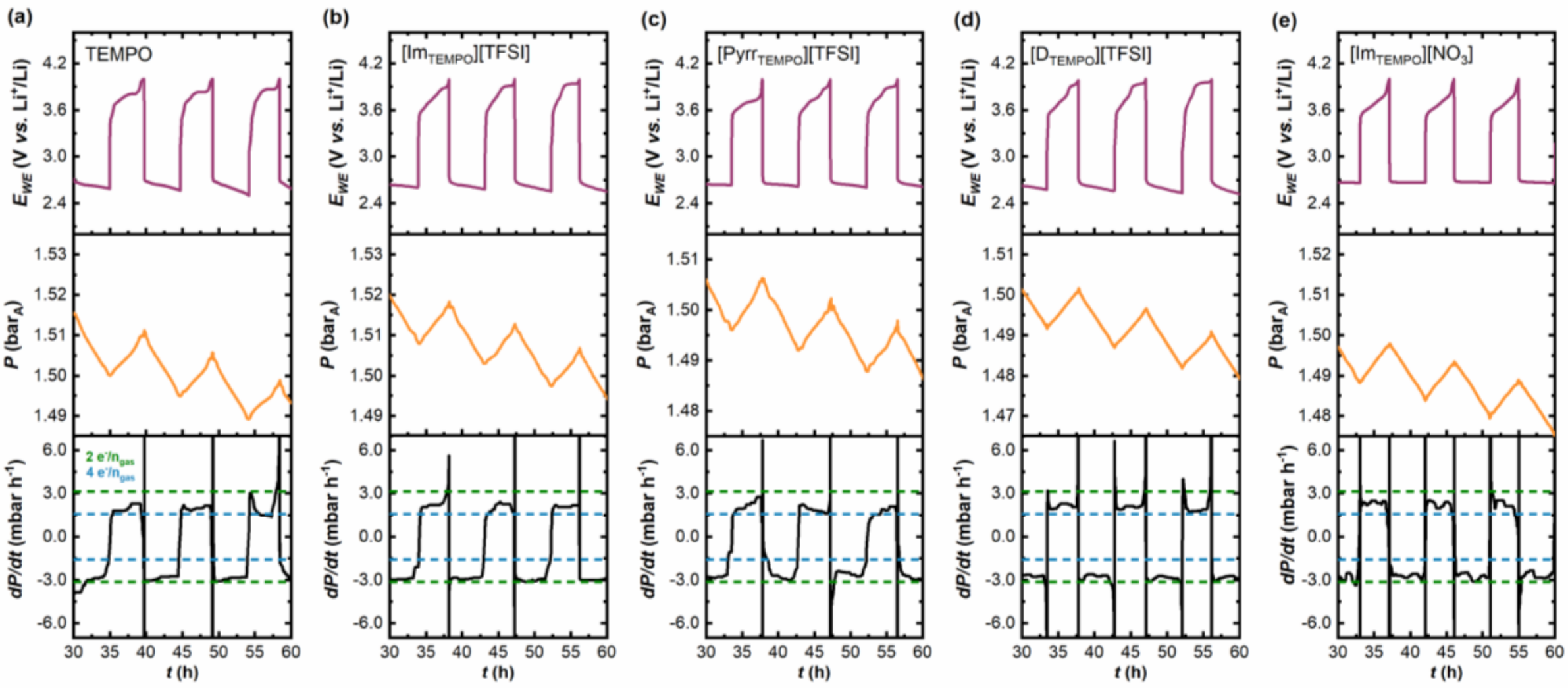

Figure S69 | Cycles 4-6 in pressure cells cycled under a capacity-limited (0.5 mAh cm^{-2}) regime at 0.1 mA cm^{-2} in 0.8 mol kg$_{solvent}^{-1}$ Li[TFSI] in diglyme electrolytes with **(a)** TEMPO, **(b)** [Im$_{TEMPO}$][TFSI], **(c)** [Pyrr$_{TEMPO}$][TFSI], **(d)** [D$_{TEMPO}$][TFSI] and **(e)** [Im$_{TEMPO}$][NO$_3$]. The RM loading was 25 mmol kg$_{solvent}^{-1}$. The cell potential (purple), pressure response (orange) and 1st derivative of the pressure response (black) are shown as a function of time. The dotted green and blue lines indicate the theoretical gas consumption/evolution rates for 2e$^-$/n$_{gas}$ and 4e$^-$/n$_{gas}$ processes, respectively. The average gas leak rate over a *ca.* 24 h rest period at open circuit potential was (3.4 ± 2.3) x 10^{-5} bar h^{-1}. The leak rate for each cell assembled was determined and used to correct the measured pressure response, as described in **Figure S67**.

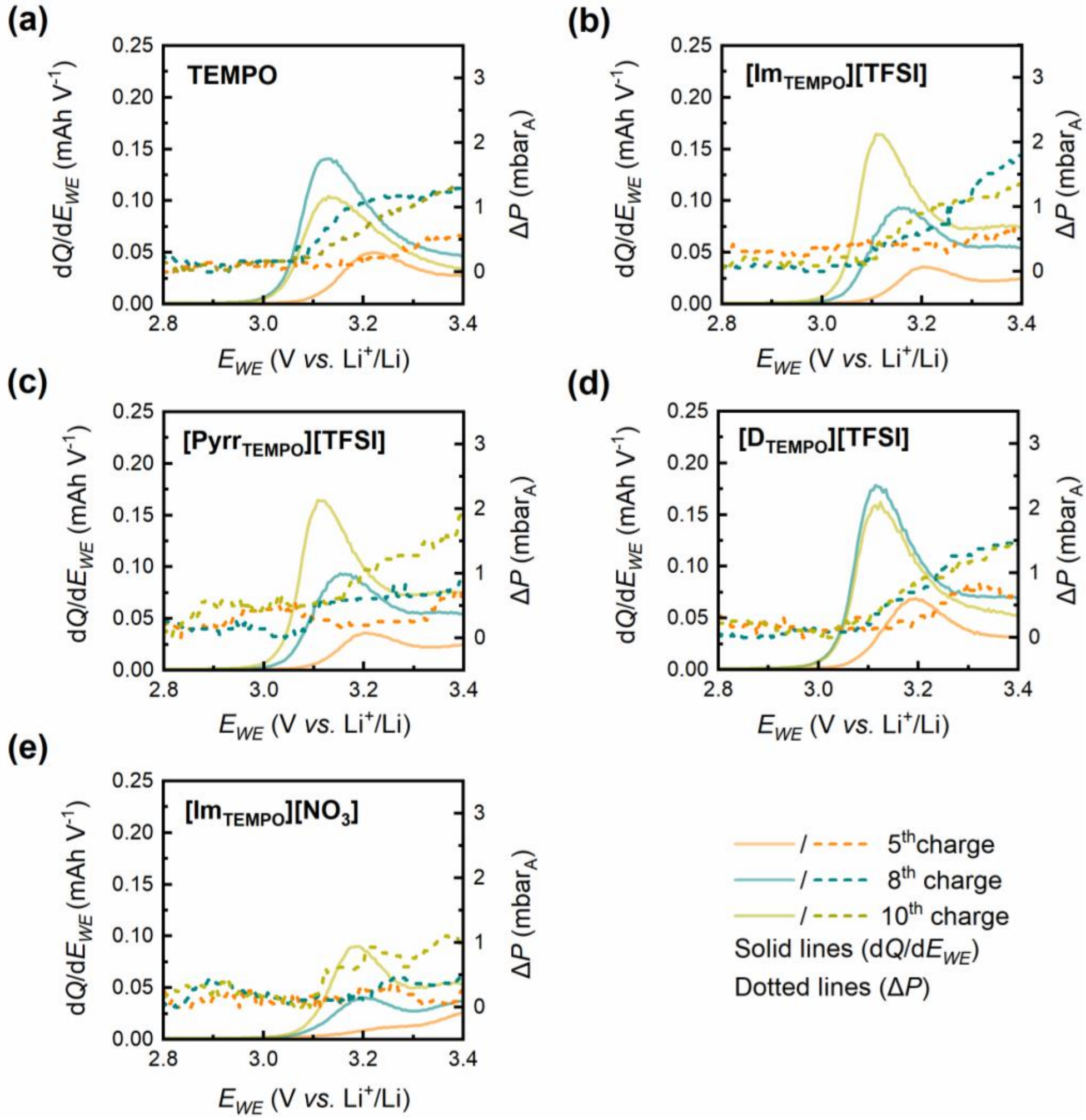

Figure S70 | Differential capacity (dQ/dE_{WE}, solid lines) and change in pressure (ΔP, dotted lines) as a function of cell potential during the 5th, 8th and 10th charge in operando pressure cells in the potential window of 2.8 to 3.4 V *vs.* Li^+/Li, highlighting the growth of a dQ/dE_{WE} peak at *ca.* 3.2 V *vs.* Li^+/Li. The electrolytes comprised 0.8 mol $kg_{solvent}^{-1}$ Li[TFSI] in diglyme with **(a)** TEMPO, **(b)** [Im_{TEMPO}][TFSI], **(c)** [$Pyrr_{TEMPO}$][TFSI], **(d)** [D_{TEMPO}][TFSI] and **(e)** [Im_{TEMPO}][NO_3]. The RM loading was 25 mmol $kg_{solvent}^{-1}$. The pressure at the start of each charge half-cycle is defined as $\Delta P = 0$ and ΔP values have been normalised by the lowest value between 2.8-3.4 V *vs*. Li^+/Li to highlight changes in ΔP within this potential window. The average gas leak rate over a *ca*. 24 h rest period at open circuit potential was $(3.4 \pm 2.3) \times 10^{-5}$ bar h^{-1}. The leak rate for each cell assembled was determined and used to correct the measured pressure response, as described in **Figure S67**.

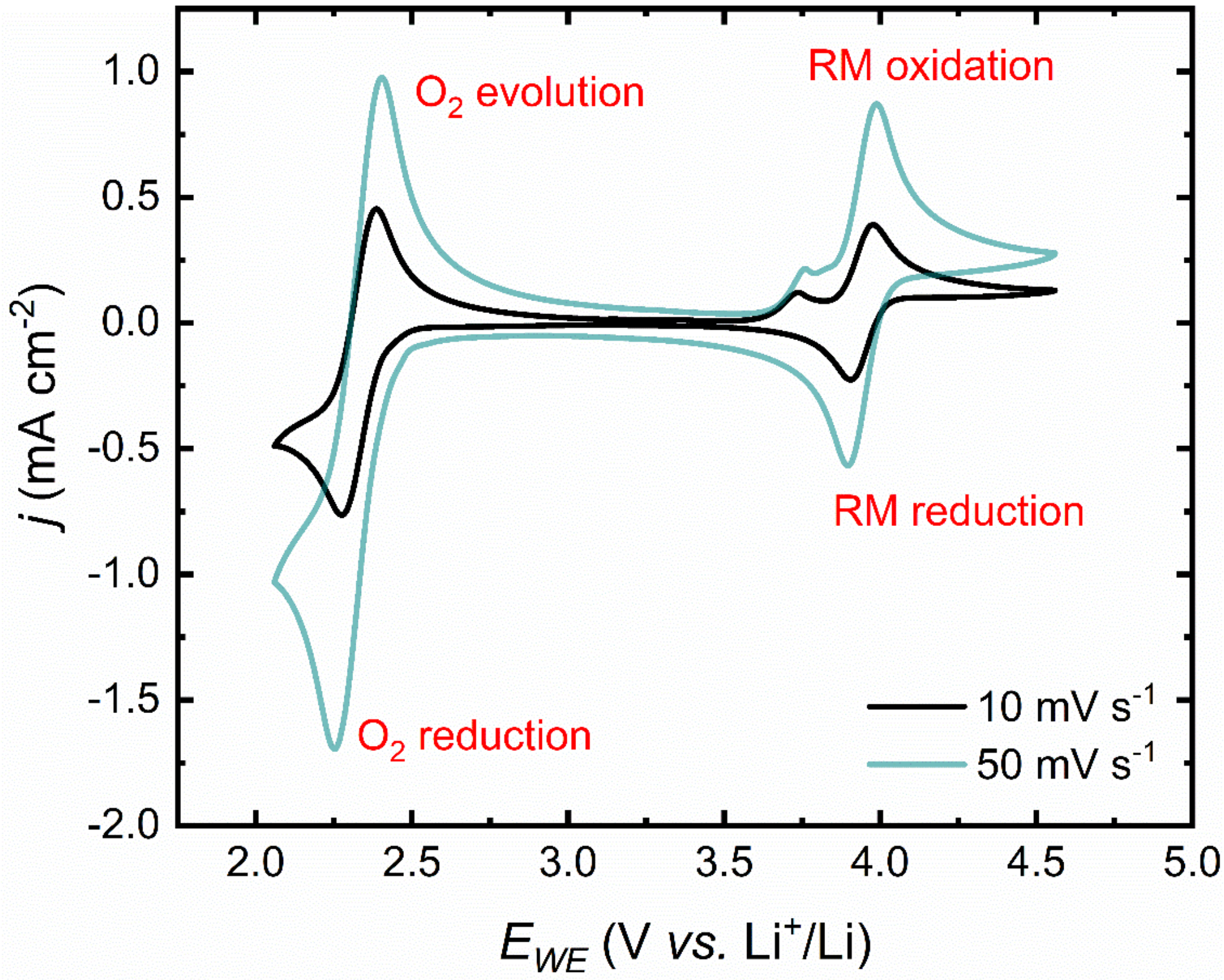

Figure S71 | CVs under O_2 in 1 M [$Pyrr_{13}$][TFSI] in diglyme with 10 mM [D_{TEMPO}][TFSI] at a scan rate of 10 mV s^{-1} (black) with comparison to the CV conducted in the same electrolyte at 50 mV s^{-1} (teal). The CVs were conducted in a three-electrode glass cell comprising a glassy carbon WE, Pt CE and Ag[OTf]/Ag RE. Cell assembly and conversion of potentials from the Ag[OTf]/Ag scale to the Li^+/Li scale are described in the ''Cyclic Voltammetry'' section of the supplementary information.

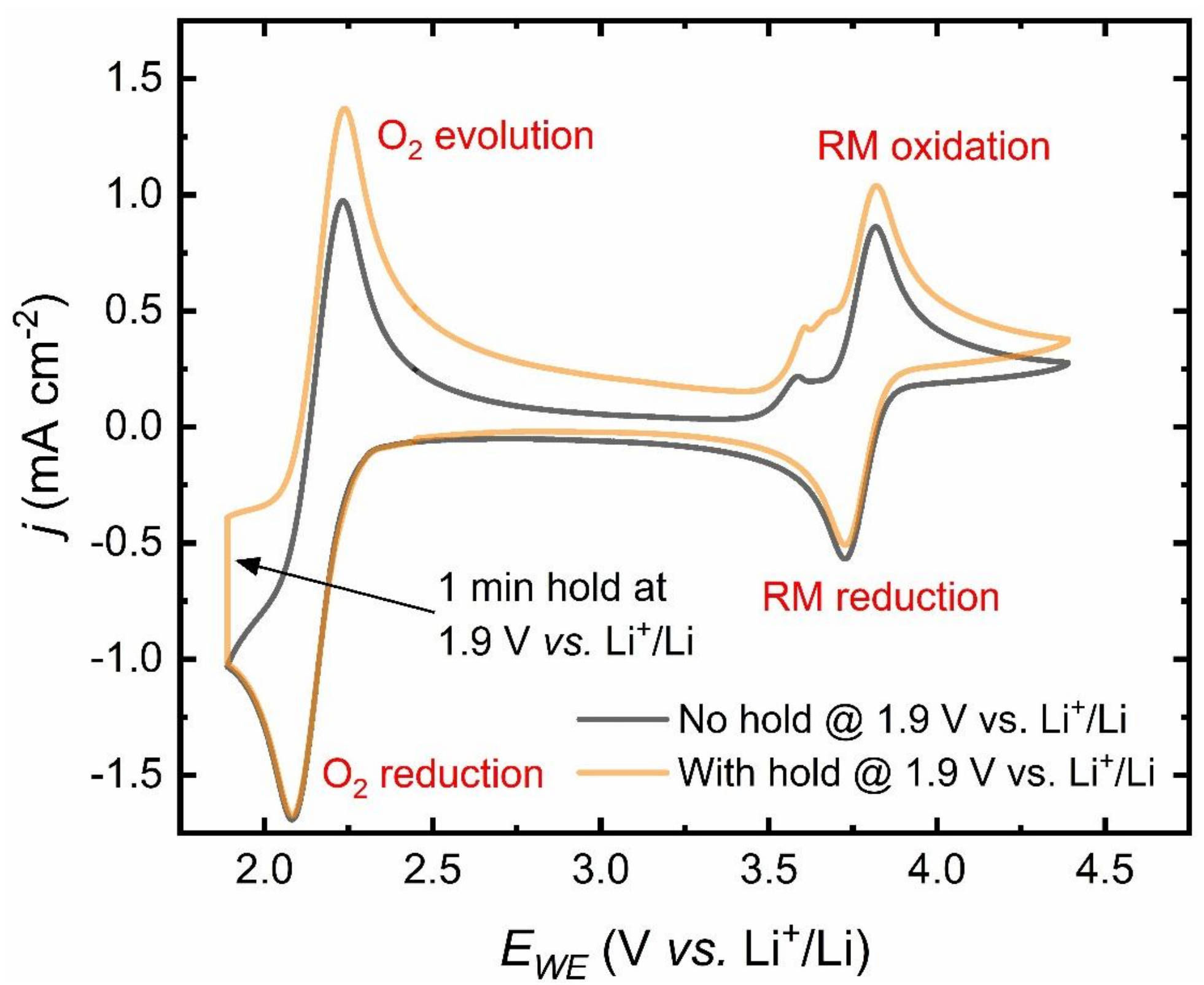

Figure S72 | CVs under O_2 in 1 M [$Pyrr_{13}$][TFSI] in diglyme with 10 mM [D_{TEMPO}][TFSI] at a scan rate of 50 mV s^{-1} with (orange) and without (black) a hold step at 1.9 V *vs.* Li^+/Li. The CVs were conducted in a three-electrode glass cell comprising a glassy carbon WE, Pt CE and Ag[OTf]/Ag RE. Cell assembly and conversion of potentials from the Ag[OTf]/Ag scale to the Li^+/Li scale are described in the ‘‘Cyclic Voltammetry’’ section of the supplementary information.

^{1}H NMR analysis of cycled electrolytes

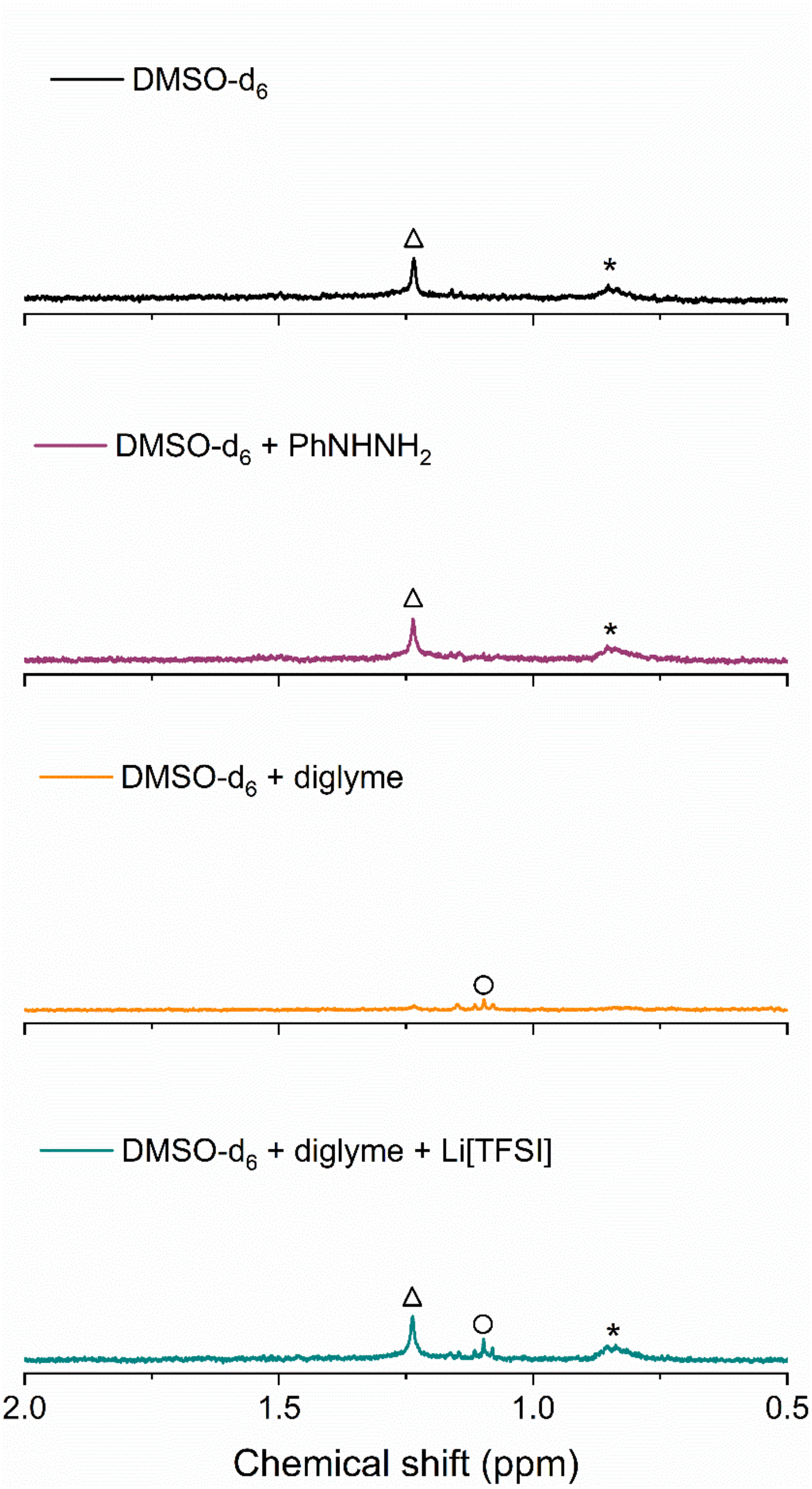

Figure S73 | ^{1}H NMR control spectra within a 0.5 to 2.0 ppm chemical shift window of DMSO-d_6 (black), DMSO-d_6 + phenylhydrazine ($PhNHNH_2$) (purple), DMSO-d_6 + diglyme (orange), DMSO-d_6 + diglyme + Li[TFSI] (teal). $PhNHNH_2$ was used to reduce nitroxyl-radical containing samples prior to the NMR experiment, which is why it is one of the control samples. The spectra have been normalised by the DMSO-d_6 solvent reference peak at 2.5 ppm. Peaks △, ○ and * are referred to in the main paper.

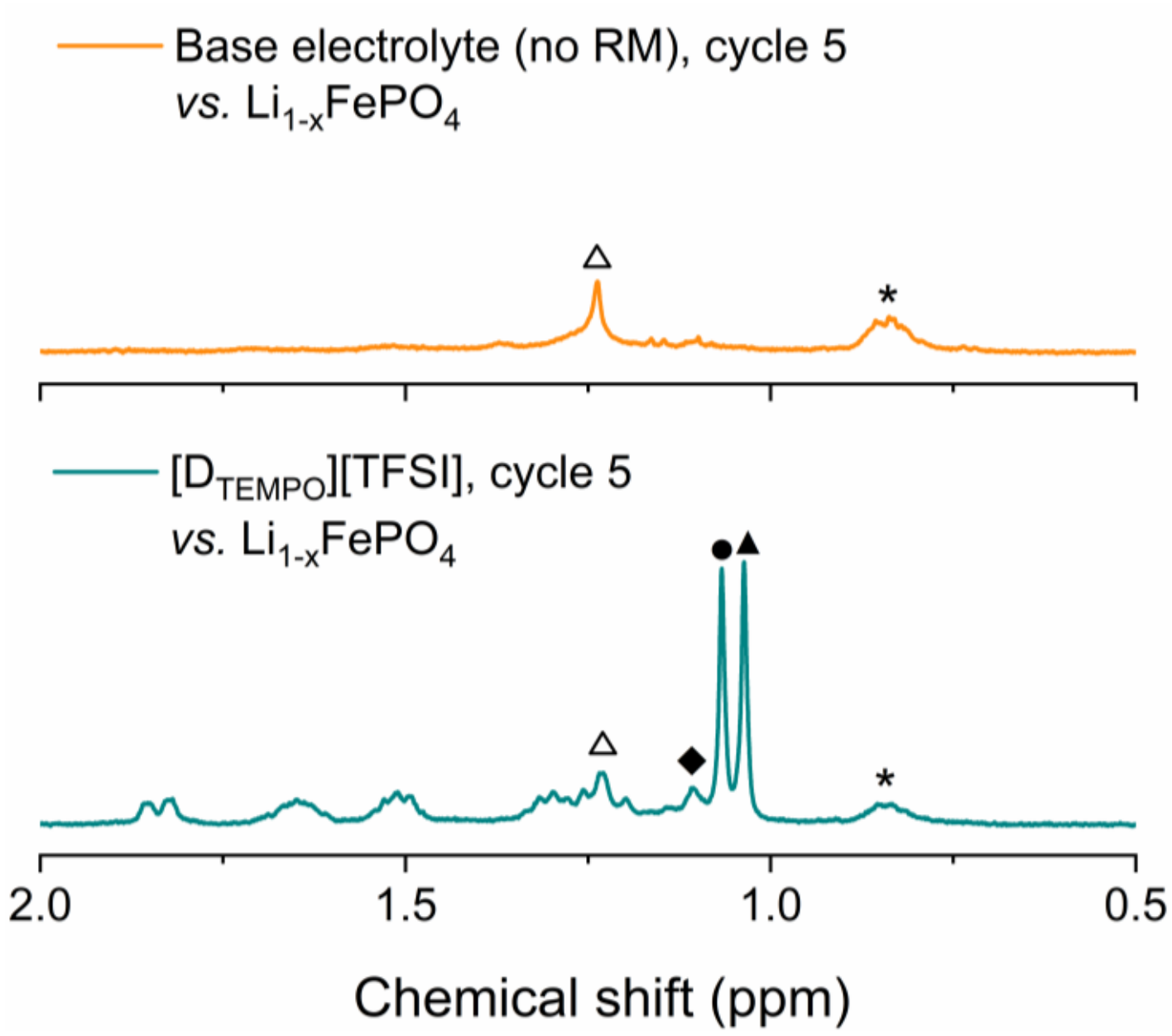

Figure S74 | ^{1}H NMR spectra of the unmediated (orange) and [D_{TEMPO}][TFSI]-containing (teal) electrolyte after 5 cycles in Li-O_2 cells with a carbon paper positive electrode and $Li_{1-x}FePO_4$ (orange, teal) as the counter electrode. Li-O_2 cells were cycled at 0.1 mA cm^{-2} with discharge and charge potential cutoffs of 2.0 and 4.0 (for [D_{TEMPO}][TFSI])/4.5 (with no RM) V *vs.* Li^+/Li, respectively. The base electrolyte was 0.8 mol $kg_{solvent}^{-1}$ Li[TFSI] in diglyme and the mediated system contained 25 mmol $kg_{solvent}^{-1}$ [D_{TEMPO}][TFSI]. Spectra are shown within a 0.5 to 2.0 ppm chemical shift window, highlighting peaks within [D_{TEMPO}][TFSI] corresponding to the alkyl chain (peaks between 1.2 and 2 ppm) and the tethered TEMPO moiety (●, ▲). The peak at 1.10 ppm (◆) grows on cycling. $PhNHNH_2$ was used to reduce nitroxyl-radical containing samples prior to the NMR experiment. The broad peak (*) was present in all electrolytes and based on the ^{1}H NMR control spectra (**Figure S73**), this peak originates from the DMSO-d_6 solvent used to extract electrolyte from cells. Peak △, which also originates from DMSO-d_6, overlaps with peaks corresponding to the alkyl chain in [D_{TEMPO}][TFSI] (see control spectra (**Figure S73**) and the ^{1}H NMR spectrum of the pure RM (**Figure S29**)). Peaks are normalised by the intensity of the DMSO-d_6 solvent reference peak at 2.5 ppm.

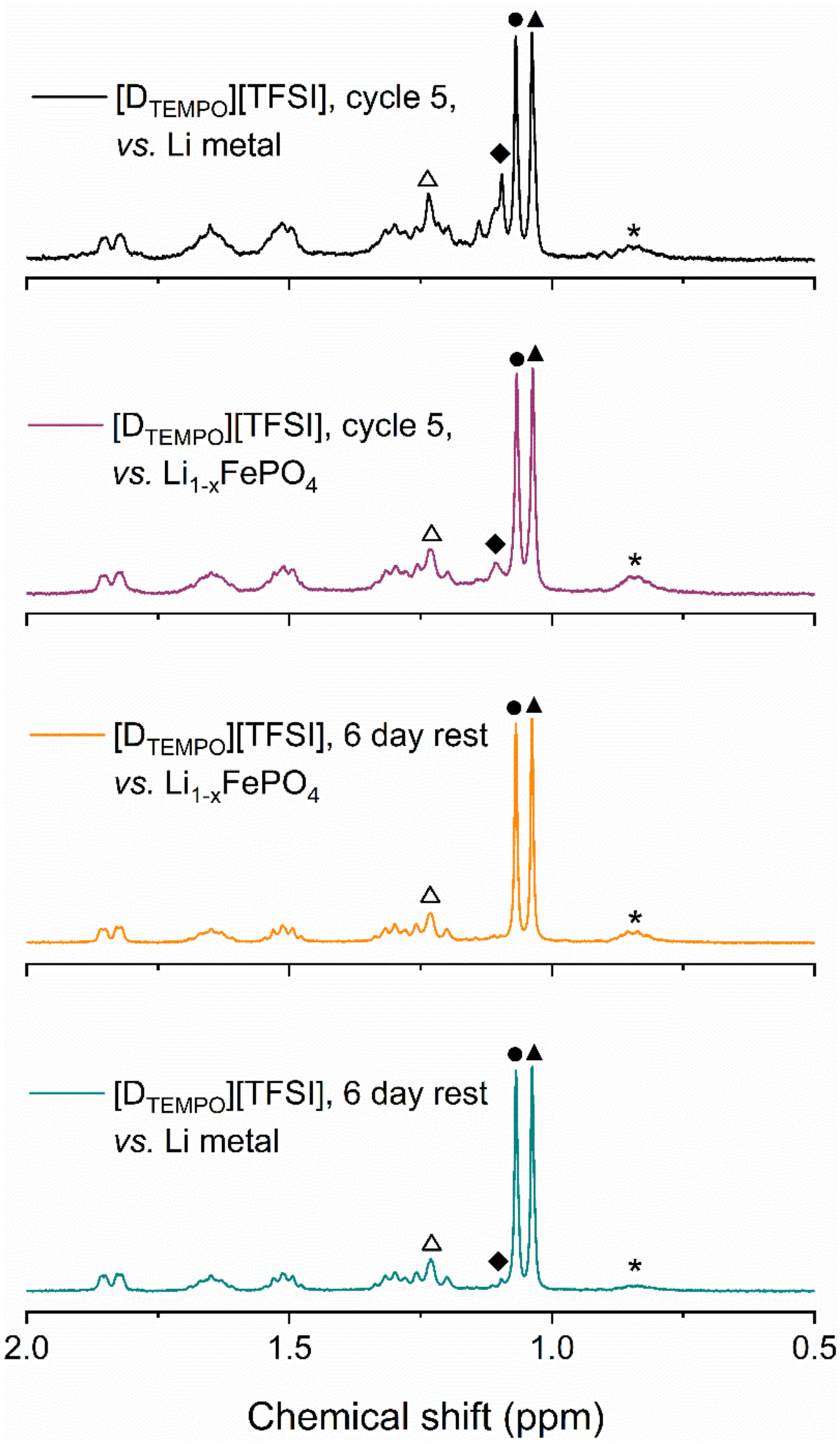

Figure S75 | ^{1}H NMR spectra of a [D_{TEMPO}][TFSI]-containing electrolyte after 5 cycles in Li-O_2 cells with Li metal (black) and $Li_{1-x}FePO_4$ (purple) as the negative electrode, and after 6 days of resting at open circuit potential under an O_2 atmosphere in Li-O_2 cells *vs.* $Li_{1-x}FePO_4$ (orange) and Li metal (teal). The electrolyte was 25 mmol $kg_{solvent}^{-1}$ [D_{TEMPO}][TFSI] in 0.8 mol kg^{-1} Li[TFSI] in diglyme. Spectra are shown within a 0.5 to 2.0 ppm chemical shift window, highlighting peaks within [D_{TEMPO}][TFSI] corresponding to the alkyl chain (peaks between 1.2 and 2 ppm) and the tethered TEMPO moiety (●, ▲). The peak at 1.10 ppm (◆) grows on cycling. $PhNHNH_2$ was used to reduce nitroxyl-radical containing samples prior to the NMR experiment. The broad peak (*) was present in all electrolytes and based on the ^{1}H NMR control spectra (**Figure S73**), this peak originates from the DMSO-d_6 solvent used to extract electrolyte from cells. Peak △, also originates from DMSO-d_6 and overlaps with peaks corresponding to the alkyl chain in [D_{TEMPO}][TFSI] (see control spectra (**Figure S73**) and the ^{1}H NMR spectrum of the pure RM (**Figure S29**)). Peaks are normalised by the intensity of peak ▲.

References

1 A. Tkacheva, J. Zhang, B. Sun, D. Zhou, G. Wang and A. M. McDonagh, *J. Phys. Chem. C*, 2020, **124**, 5087–5092.

2 G. H. Coleman, *Org. Synth.*, 1922, **2**, 71.

3 J. Zhang, B. Sun, Y. Zhao, A. Tkacheva, Z. Liu, K. Yan, X. Guo, A. M. McDonagh, D. Shanmukaraj, C. Wang, T. Rojo, M. Armand, Z. Peng and G. Wang, *Nat. Commun.*, 2019, **10**, 602.

4 O. B. Babushkina, *Z. Naturforsch.*, 2008, **63a**, 66–72.

5 V. I. Kovalenko, A. A. Akhmadiyarov, A. E. Vandyukov and A. R. Khamatgalimov, *J. Mol. Struct.*, 2012, **1028**, 134–140.

6 A. Zulkifli, N. Mazuki and A. S. Samsudin, *Makara J. Technol.*, 2021, **25**, 55.

7 Z. B. Alfassi, R. E. Huie, B. L. Milman and P. Neta, *Anal. Bioanal. Chem.*, 2003, **377**, 159–164.

8 R. Chen, M. Yang, Y. Shi, H. Wang, H. Guo, Y. Zeng, J. Qi, Q. Shi, Z. Liao and T. Lu, *Int. J. Hydrog. Energy*, 2019, **44**, 20249–20256.

9 G. A. Snook, A. S. Best, A. G. Pandolfo and A. F. Hollenkamp, *Electrochem. Commun.*, 2006, **8**, 1405–1411.

10 B. J. Bergner, A. Schürmann, K. Peppler, A. Garsuch and J. Janek, *J. Am. Chem. Soc.*, 2014, **136**, 15054–15064.

11 B. J. Bergner, C. Hofmann, A. Schürmann, D. Schröder, K. Peppler, P. R. Schreiner and J. Janek, *Phys. Chem. Chem. Phys.*, 2015, **17**, 31769–31779.

12 Y. Chen, X. Gao, L. R. Johnson and P. G. Bruce, *Nat. Commun.*, 2018, **9**, 767.

13 Y. Yoon, S. Shin and M. W. Shin, *ACS Appl. Mater. Interfaces*, 2022, **14**, 4220–4229.

14 J. Zhang, Y. Zhao, B. Sun, Y. Xie, A. Tkacheva, F. Qiu, P. He, H. Zhou, K. Yan, X. Guo, S. Wang, A. M. McDonagh, Z. Peng, J. Lu and G. Wang, *Sci. Adv.*, 2022, **8**, eabm1899.

15 J. Liu, Y. Li, Y. Ding, L. Wu, J. Qin, T. Chen, C. Meng, F. Zhou, X. Ma and Z. Wu, *Angew. Chem. Int. Ed.*, 2025, e202421107.

16 D. Li, C. Zhu, M. Zhang, Y. Wang, Z. Kang, Y. Liu, J. Liu, J. Liu and H. Xie, *Chem. Eng. J.*, 2021, **408**, 127335.

17 Z. Sun, Y. Tan, Y. Zhan, K. Li, W. Dou, C. Wang, X. Lin, R. Yuan, J. Yan, M. Zheng and Q. Dong, *Small Methods*, 2023, **7**, 2201289.

18 T. Zhang, K. Liao, P. He and H. Zhou, *Energy Environ. Sci.*, 2016, **9**, 1024–1030.

19 C. Zheng, W. Ding and C. Wang, *J. Mater. Chem. A*, 2019, **7**, 6180–6186.

20 M.-L. Gao, Q.-Y. Wang, Y.-L. Liao, W.-R. Chen, Z.-Y. Huang, X.-X. Zhuang and X.-P. Zhang, *Chem. Commun.*, 2024, **60**, 9058–9061.

21 Z. Sun, X. Lin, W. Dou, Y. Tan, A. Hu, Q. Hou, R. Yuan, M. Zheng and Q. Dong, *Adv. Energy Mater.*, 2022, **12**, 2102764.

22 W. Yu, W. Yang, R. Liu, L. Qin, Y. Lei, L. Liu, D. Zhai, B. Li and F. Kang, *Electrochem. Commun.*, 2017, **79**, 68–72.

23 Q. Xiong, G. Huang and X. Zhang, *Angew. Chem.*, 2020, **132**, 19473–19481.

24 S. Xing, Z. Zhang, Y. Dou, M. Li, J. Wu, Z. Zhang and Z. Zhou, *CCS Chem.*, 2024, **6**, 1810–1820.

25 F. S. Gittleson, K. P. C. Yao, D. G. Kwabi, S. Y. Sayed, W. Ryu, Y. Shao-Horn and A. D. Taylor, *ChemElectroChem*, 2015, **2**, 1446–1457.

26 T. Samarakoon, B. Wood, A. R. Neale, E. Coulbeck, D. J. Saccomando and L. J. Hardwick, *Chem. Sci.*, 2025, **16**, 11359–11374.
